# Intermolecular selective carboacylation of alkenes via nickel-catalyzed reductive radical relay

**DOI:** 10.1038/s41467-018-05951-6

**Published:** 2018-08-28

**Authors:** Xian Zhao, Hai-Yong Tu, Lei Guo, Shengqing Zhu, Feng-Ling Qing, Lingling Chu

**Affiliations:** 1 State Key Laboratory for Modification of Chemical Fibers and Polymer Materials, College of Chemistry, Chemical Engineering and Biotechnology, Center for Advanced Low-Dimension Materials, Donghua University, Shanghai, 201620 China; 2 Key Laboratory of Organofluorine Chemistry, Shanghai Institute of Organic Chemistry, Chinese Academy of Science, Shanghai, 200032 China

## Abstract

The development of catalytic carboacylation of simple olefins, which would enable the rapid construction of ketones with high levels of complexity and diversity, is very challenging. To date, the vast majority of alkene carboacylation reactions are typically restricted to single- and two-component methodologies. Here we describe a three-component carboacylation of alkenes via the merger of radical chemistry with nickel catalysis. This reaction manifold utilizes a radical relay strategy involving radical addition to an alkene followed by alkyl radical capture by an acyl-nickel complex to forge two vicinal C−C bonds under mild conditions. Excellent chemoselectivity and regioselectivity have been achieved by utilizing a pendant weakly chelating group. This versatile protocol allows for facile access to a wide range of important β-fluoroalkyl ketones from simple starting materials.

## Abstract (web summary)

To date the carboacylation of alkenes has been reported only in single- and two-component methodologies. Here, the authors report a three-component nickel-catalyzed carboacylation of olefins which enables the rapid construction of ketones with high levels of complexity and diversity.

## Introduction

Ketones are important structural motifs prevalent in pharmaceuticals, agrochemicals and natural products^1,2^, as well as versatile intermediates for numerous valuable transformations. Transition metal-catalyzed cross coupling with acyl electrophiles has emerged as an efficient platform for ketones synthesis^3–6^, providing an attractive alternative to classical nucleophilic carbonyl addition and Friedel−Crafts acylation^7^. Particularly, catalytic carboacylation of alkenes in the presence of transition metal catalysts has attracted considerable attention in synthetic chemistry^8–25^, due to the fact that: (i) alkenes are abundant and ubiquitous building blocks in chemical and material industries; (ii) this protocol installs multiple C−C bonds across an olefin in one step, allowing for rapid access to complex ketones from simple starting materials.

To date, the vast majority of alkene carboacylation reactions proceed via intramolecular insertion of an acyl-metal intermediate into an alkene. As a result, this reaction is typically restricted to single-component variants, with very few exceptions of two-component reactions^12,18^. The development of fully intermolecular, three-component carboacylation of simple olefins, which would enable the rapid construction of ketone products with high levels of complexity and diversity, is highly desired and remains elusive. One main challenge to this approach is the propensity for decarbonylation from an acyl-metal species^26–28^, which must intercept the alkene. Another challenge associated with a three-component variant is achieving regioselectivity and chemoselectivity. A number of elegant methods have been reported to control regioselectivity in transition metal-catalyzed three-component dicarbofunctionalization of alkenes. For instance, previous studies have demonstrated that employment of activated substrates can facilitate regiocontrol^29–40^. More recently, utilization of directing groups to achieve highly regioselective dicarbofunctionalization of alkenes has also been reported^41–49^. Nevertheless, the capability to achieve selective alkene functionalization in the presence of multiple double bonds remains a longstanding challenge.

**Fig. 1:**
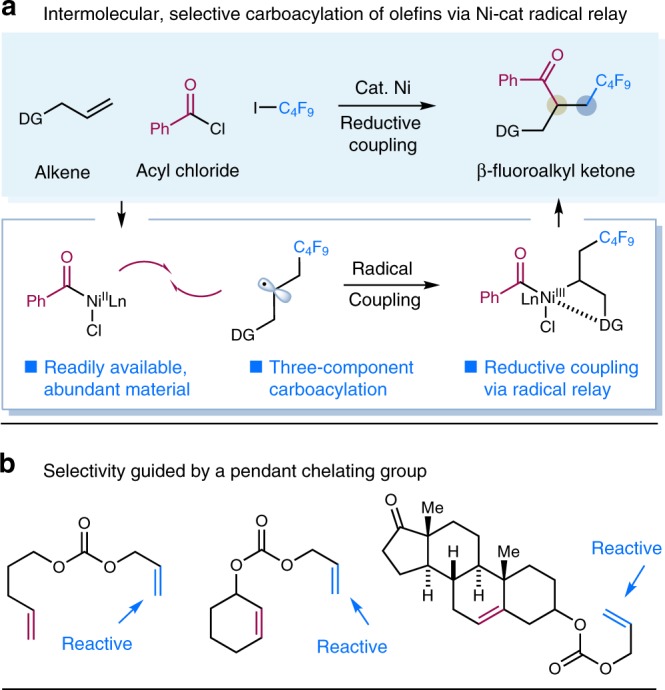
Design of an intermolecular, selective carboacylation of alkenes. **a** Three-component carboacylation of olefins via nickel-catalyzed reductive radical relay. **b** Selectivity guided by a chelating group

Over the last decade, nickel-catalyzed cross-coupling has emerged as a powerful tool to forge C−C bonds in chemical synthesis^50–52^. Particularly, the merger of radical chemistry with nickel catalysis has enabled the invention of numerous useful transformations^51,52^. We recently questioned whether radical-based nickel catalysis might offer an alternative pathway to facilitate the three-component carboacylation of alkenes. Specifically, we envisioned that a radical relay strategy, involving radical addition to an alkene followed by alkyl radical capture by an acyl-nickel species^53–55^ would bypass the challenging acyl-metal/alkene capture required in a conventional two-electron mode. Furthermore, we anticipated that a pendant chelating group could facilitate the capture of the alkyl radical by the nickel species, while also controlling chemoselectivity and regioselectivity via coordination. This strategy described was also validated in seminal work by Nevado and coworkers in which an allylic acetate could serve to stabilize a putative alkylnickel species after radical recombination^44^. Given the increasing importance of fluoroalkyl moieties in the areas of medicinal, agrochemical, and material chemistry^56,57^ as well as elegant progress in radical fluoroalkylation of alkenes^58–62^, we sought to explore the carboacylation of alkenes with fluoroalkyl precursors. Herein, we report selective, three-component carboacylation of alkenes with acyl chlorides via Ni-catalyzed radical relay (Fig. 1). This method takes advantage of Ni-catalyzed reductive coupling of electrophiles^3,63–66^ under mild conditions, delivering valuable β-fluoroalkyl carbonyls that are not easily accessible by other methods^66,67^.

## Results

### Optimization study

We first sought to employ carbonyls, ubiquitous intermediates in organic synthesis, as chelating groups^68^. Evaluation of this nickel-catalyzed intermolecular reductive carboacylation strategy was first examined with allyl hexanoate, 4-*tert*-butylbenzoyl chloride, and perfluorobutyl iodide in the presence of nickel catalysts and reductants. We were delighted to find that 93% yield of the desired carboacylation product could be obtained with Mn dust as stoichiometric reductant in the presence of catalytic amounts of NiCl_2_•glyme and 4,4′-di-*tert*-butyl-2,2′-dipyridyl (dtbbpy) at 25 °C (See Supplementary Table 1). Control experiments have demonstrated that both the nickel catalyst and Mn dust are essential for the desired transformation to proceed, while moderate yield of **3** can be obtained in the absence of ligand, indicating the chelating ability of the pendant ester group (See Supplementary Table 1).

### Substrate scope

**Fig. 2:**
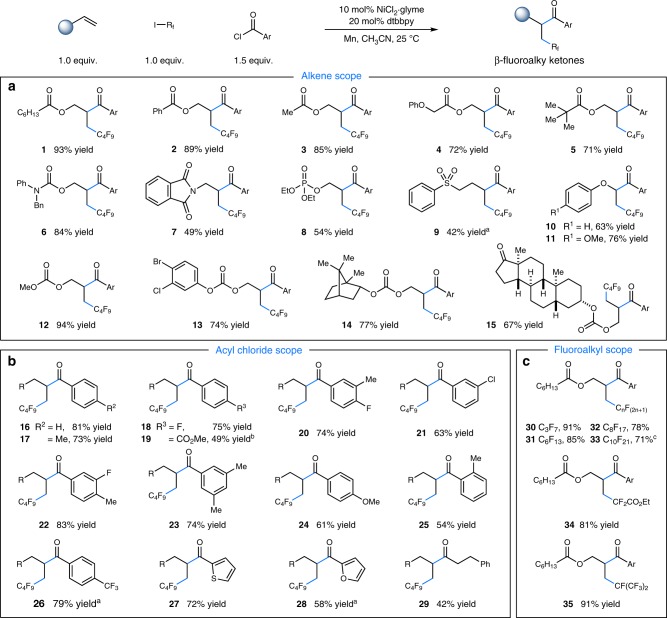
Substrate scope. **a** Scope of alkenes. **b** Scope of acyl chlorides. **c** Scope of fluoroalkyl iodides. Reaction conditions: NiCl_2_•glyme (10 mol%), dtbbpy (20 mol%), alkene (1.0 equiv.), R_f_I (1.0 equiv.), acyl chloride (1.5 equiv.), Mn (3.0 equiv.), CH_3_CN [0.1 M], 25 ^o^C, 20 h, see Supplementary Methods. All cited yields are isolated yields. Ar = 4-*tert*-butylphenyl, R = C_6_H_13_CO_2_. ^a^ Performed with 20 mol% NiCl_2_•glyme. ^b^ Reaction concentration is 0.05M CH_3_CN. ^c^Performed in CH_3_CN/DME (4:1) [0.1 M]

With optimal reaction conditions in hand, we explored the generality of this carboacylation with respect to the alkene fragment and found that a variety of readily available alkenes are viable partners for this transformation (Fig. 2a). Alkenes tethered with different chelating groups, including esters, carbonates, carbamates, sulfonates, and phosphates, undergo the desired reductive coupling with moderate to excellent efficiency (products **1**−**9**, 42−93% yields). Interestingly, electron-rich aryl rings were capable of guiding the desired transformations^69^, exemplified by the reaction of aryl vinyl ethers to furnish α-oxy-β-fluoroalkyl ketones in high yields (products **10** and **11**, 63% and 76% yields, respectively). The mild reaction conditions allow for good compatibility with a wide range of important functional groups including aryl bromides and chlorides, providing a versatile platform for further synthetic manipulations (products **13**, 74% yield). Gratifyingly, more complex partners derived from naturally occurring molecules were also successfully employed, demonstrating the potential applicability of this methodology in late-stage functionalization. For example, derivatives of borneol and estrone functioned as competent alkene partners, furnishing each of the desired coupling products with high efficiency (products **14** and **15**, 77% and 67% yields, respectively). Notably, acyclic internal alkenes, such as (*E*)-but-2-en-1-yl benzoate, also participated in this Ni-catalyzed difunctionalization manifold with moderate efficiency (see Supplementary Fig. 5).

Next, we evaluated the scope of the acyl chloride component in this protocol. As revealed in Fig. 2b, aromatic acyl chlorides containing electron-neutral, -donating, and -withdrawing groups proceeded smoothly under the optimal conditions, delivering the product ketones with moderate to excellent efficiency (products **16**−**26**, 49−83% yields). Moreover, *ortho* substitution on the aryl ring was tolerated, albeit with somewhat diminished efficiency (product **25**, 54% yield). Notably, heteroaromatic acyl chlorides such as thiophene and furan were also viable substrates, furnishing the corresponding heteroaryl ketones in moderate yields (products **27** and **28**, 72% and 58% yields, respectively). Additionally, aliphatic acyl chlorides demonstrated promising levels of efficiency, as exemplified by dialkyl ketone product **29** (42% yield).

Finally, we examined this three-component functionalization protocol with varied fluoroalkyl precursors (Fig. 2c). A series of perfluoroalkyl iodides, including ethyl iododifluoroacetate, can be readily employed with excellent levels of efficiency (products **30**−**35**, 53−91% yields), providing a simple and efficient way to incorporate perfluoroalkyl substituents into complex molecules. Importantly, this efficient alkene carboacylation strategy employs a 1:1 ratio of alkene and fluoroalkyl iodide at room temperature, without the need for excess amounts of perfluoroalkyl iodides in all cases (Fig. 2). Gratifyingly, trifluoroiodomethane (CF_3_I) was also reactive, affording the desired trifluoromethylacylation product in promising levels of efficiency (29% yield) (see Supplementary Fig. 6). Additionally, electron-deficient tertiary alkyl bromides were also suitable coupling partners under slightly modified conditions. For example, the reaction of ethyl 2-bromo-2-methylpropanoate gave the desired carboacylated product in 36% yield (see Supplementary Fig. 7).

**Fig. 3:**
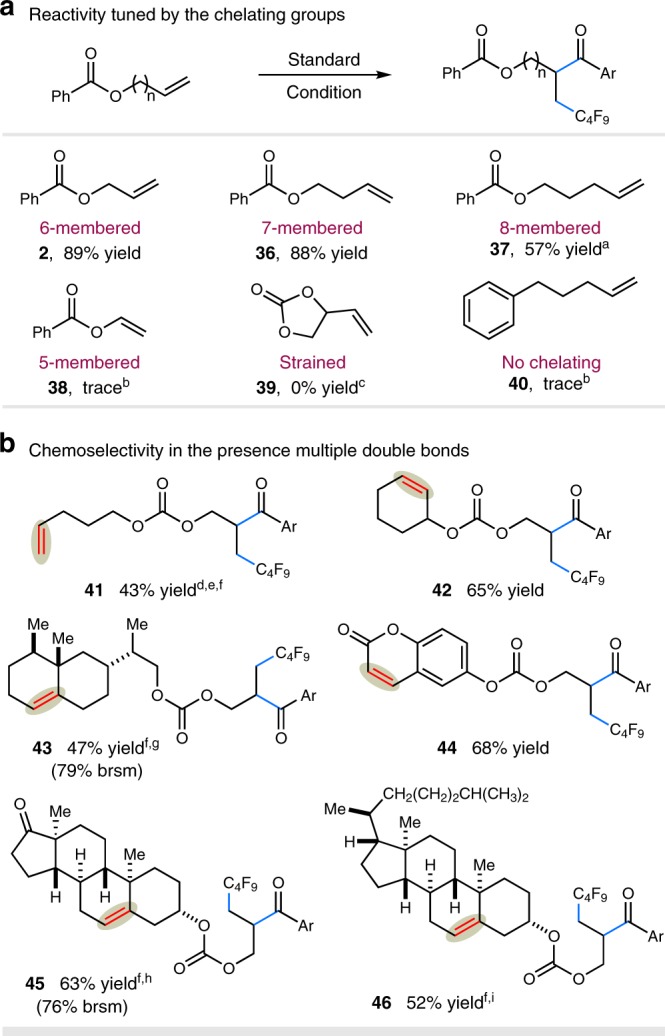
Chemoselectivity guided by pendant chelating groups. **a** Reactivity tuned by the chelating group. **b** Chemoselectivity in the presence of multiple double bonds. Reaction conditions: NiCl_2_•glyme (10 mol%), dtbbpy (20 mol%), alkene (1.0 equiv.), C_4_F_9_I (1.0 equiv.), acyl chloride (1.5 equiv.), Mn (3.0 equiv.), CH_3_CN [0.1 M], 25 ^o^C, 20 h, see Supplementary Methods. All cited yields are isolated yields. Ar = 4-*tert*-butylphenyl. ^a^36% of C_4_F_9_-alkene byproduct isolated. ^b^Alkene consumed. ^c^Alkene remained. ^d^Performed with 2 equiv. of alkene. ^e^140% of alkene recovered. ^f^Performed in 4:1 CH_3_CN/DME [0.05 M]. ^g^41% of alkene recovered. ^h^17% of alkene recovered. ^i^48 h

The effect of the weakly coordinating group in chelation was evaluated by testing a set of different substrates shown in Fig. 3a. In cases of substrates which form six- or seven-membered chelate rings, the reactions proceeded with excellent efficiency (**2** and **36**, 89% and 88% yields, respectively). A slightly decreased efficiency was observed in the case of the substrate proceeding via an eight-membered chelate ring (**37**, 57% yield). It is worth noting, however, that the reaction of vinyl benzoate **38**, bearing a potential five-membered chelate ring, is also hampered by starting material decomposition under the standard conditions. Moreover, alkenes bearing strained or non-chelating groups were ineffective, further highlighting the importance of the directing groups (**39** and **40**).

Intrigued by these results, we examined the chemoselectivity and regioselectivity with substrates bearing multiple double bonds. As shown in Fig. 3b, our reductive protocol can be used for selective functionalization of alkenes through the directing effect. Exclusive site selectivity was observed for carboacylation of terminal alkenes that could form six-membered chelate rings, leaving the non-chelating alkenes untouched (products **41**−**46**, 43−68% yields). Notably, for substrates in which two terminal alkenes are present, excellent site selectivity was observed at olefins guided by the tethered chelating group with a more favorable chelate geometry (products **41**, 43% yield), further highlighting the powerful selectivity feature of this catalytic system. Additionally, olefins derived from naturally occurring molecules, including valencene, coumarin, dehydroisoandrosterone, and cholesterone, could also be employed to furnish the desired products with moderate efficiency and excellent selectivity (products **43**−**46**, 47−68% yields), demonstrating the inherent value of our carboacylation protocol in late-stage functionalization.

**Fig. 4:**
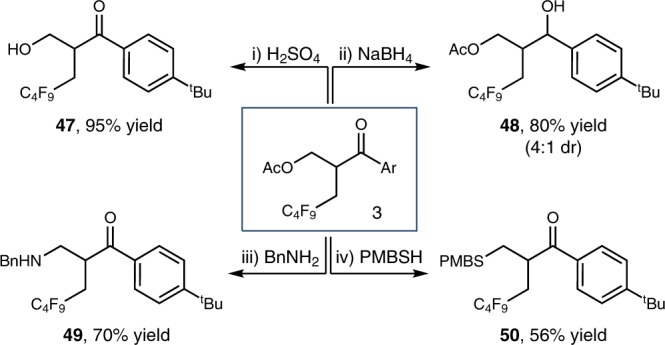
Derivations of compound **3**. See Supplementary Methods. Ar = 4-*tert*-butylphenyl. PMB = 4-methoxybenzyl

To further demonstrate the synthetic benefit of our nickel-catalyzed reductive carboacylation strategy, the difunctionalized products were converted to several useful synthetic functionalities (Fig. 4). The acetyl group of compound **3** can be readily removed under acidic conditions to give alcohol **47** in 95% yield. Selective reduction of aryl ketones with NaBH_4_ led to the formation of secondary alcohol **48** in 80% yield. Furthermore, nucleophilic substitution of **3** with benzylamine afforded γ-fluoroalkylated amine **49** in 70% yield. γ-Fluoroalkylated thioether **50** was obtained in 56% yields through a β-elimination/Michael addition reaction with 4-methoxy-α-toluenethiol.

### Proposed mechanism and mechanistic studies

**Fig. 5:**
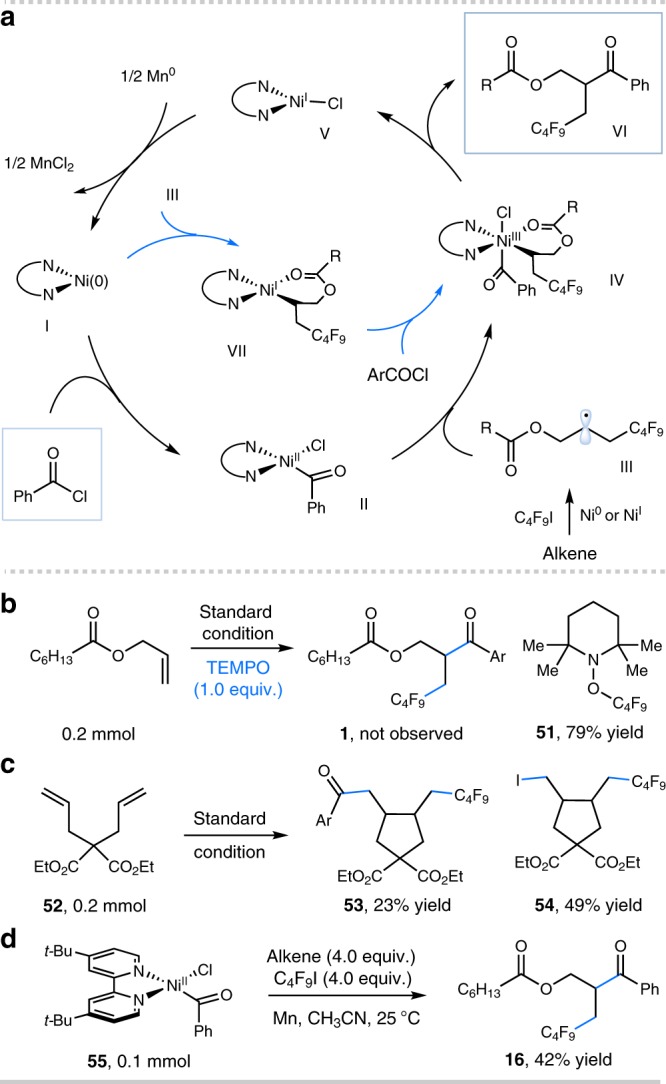
Mechanistic studies. **a** Proposed mechanism. **b** Radical inhibition experiment. **c** Radical clock experiment. **d** Stoichiometric reaction of isolated Ni(II) complex. See Supplementary Discussion. Ar = 4-*tert*-butylphenyl. alkene = allyl heptanoate

Our proposed mechanism for this Ni-catalyzed reductive coupling is outlined in Fig. 5a. Oxidative addition of the active Ni(0) species **I** to acyl chloride affords Ni^II^ complex **II**^70^. Concurrently, Ni(0)- or Ni^I^-mediated single-electron reduction of C_4_F_9_I generates the electrophilic C_4_F_9_ radical, and subsequent radical addition to the alkene coupling partner would deliver alkyl radical species **III**. At this juncture, we expected that Ni^II^ complex **II** could be intercepted by nucleophilic alkyl radical **III** to yield Ni^III^ adduct **IV**^51,52^, which would undergo reductive elimination to deliver the final product and Ni^I^ species **V**. Single-electron reduction of Ni^I^ **V** (*E*_red_ [Ni^II^/Ni^0^] = –1.2 V vs SCE in DMF)^71^ by Mn dust (*E*_red_ = −1.4 V vs SCE in MeCN) would regenerate Ni(0) species **I** and complete the catalytic cycle. We expected that the chelating group would affect the feasibility and stability of the Ni^III^ complex, thus influencing the reactivity of this reaction. Alternatively, alkyl radical **III** could be captured by Ni(0) to form Ni^I^ complex **VII**^72^, followed by oxidative addition of acyl chloride to deliver the crucial Ni^III^ adduct **IV**.

We have conducted several preliminary mechanistic experiments to elucidate the proposed mechanism (Fig. 5b–d). Addition of TEMPO (1.0 equiv.) completely shuts down the desired transformation, and TEMPO-C_4_F_9_ adduct **51** was observed in 79% ^19^F NMR yield (Fig. 5b). Moreover, diene **52** underwent radical addition and cyclization, furnishing the expected coupling product **53** in 23% yield as well as alkyl iodide **54** in 49% yield (Fig. 5c). These results indicate that radical intermediates are involved in this system. Importantly, the stoichiometric reaction of Ni-complex **55** with alkene and C_4_F_9_I in the presence of Mn dust gave the desired coupling product **16** in 42% yield (Fig. 5d), suggesting that the catalytic pathway proceeding via oxidative addition of Ni(0) species with acyl chloride could be operative (Fig. 5a).

## Discussion

In conclusion, we have developed a robust strategy for intermolecular, three-component carboacylation of alkenes with acyl chlorides and fluoroalkyl iodides via a Ni-catalyzed radical relay. This versatile protocol enables facile access to β-fluoroalkyl ketones through the regioselective, sequential formation of two C−C bonds in one step under mild conditions. We expect that the generality of this methodology and readily availability of the starting materials will allow it to enjoy extensive application in the area of organic chemistry.

## Methods

### General procedure for the carboacylation reaction

To a flame-dried 8 mL reaction vial was charged with NiCl_2_•glyme (0.02 mmol, 10 mol%), 4,4′-di-*tert*-butyl-2,2′-dipyridyl (0.04 mmol, 20 mol%), and Mn (0.6 mmol, 3.0 equiv.). The vial was capped. After it was evacuated and backfilled nitrogen three times, CH_3_CN [0.1 M] was added via a syringe, followed by the addition of acyl chloride (0.3 mmol, 1.5 equiv.). The reaction mixture was allowed to stir for approximately 1 min before fluoroalkyl iodide (0.2 mmol, 1.0 equiv., if liquid) and alkene (0.2 mmol, 1.0 equiv. if liquid) were added. The reaction mixture was allowed to stir at 1500 rpm for 20 h at 25 °C. The reaction was quenched with 1 N HCl, extracted with ethyl acetate (EA) three times. The combined organic layers were dried with MgSO_4_, filtered, and concentrated in vacuo. The crude material was purified by flash chromatography to afford the product. See Supplementary Methods for further experimental details.

### Data availability

The authors declare that all the data supporting the findings of this work are available within the article and its Supplementary Information files or from the corresponding author upon request.

## Electronic supplementary material

Supplementary Information
