# Supplementary material for: Intermolecular selective carboacylation of alkenes via nickel-catalyzed reductive radical relay
Source: Nat Commun. 2018 Aug 28;9:3488. doi: 10.1038/s41467-018-05951-6 (PMC6113317; doi:10.1038/s41467-018-05951-6)
Supplement: Supplementary file 1 — Supplementary Information [file 41467_2018_5951_MOESM1_ESM.pdf]

Supplementary Materials for

Intermolecular Selective Carboacylation of Alkenes via Nickel-Catalyzed Reductive Radical Relay

Chu *et al*

correspondence to: lingling.chu1@dhu.edu.cn

This PDF files includes:

Supplementary Methods

Supplementary Discussion

Supplementary Table 1 – 2

Supplementary Figure 1 – 87

Supplementary References

1. Supplementary Methods

General Considerations: Commercial reagents were purchased from Aldrich, TCI, Energy Chemical and J&K chemical, and were used as received. All reactions were carried out in oven-dried glassware under an atmosphere of nitrogen unless otherwise noted. Chromatographic purification of products was accomplished by flash chromatography using silica gel. Thin-layer chromatography (TLC) was performed on Silicycle 250 mm silica gel F-254 plates. ^{1}H, ^{19}F NMR, and ^{13}C NMR spectra were recorded on Bruker 400 (400, 376, and 100 MHz) and Bruker 600 (600, 564, and 150 MHz), and are internally referenced to residual solvent signals (for $CDCl_3$, δ 7.26 and 77.0 ppm). Data for ^{1}H NMR and ^{19}F NMR are reported as follows: chemical shift (δ ppm), multiplicity (s = singlet, d = doublet, t = triplet, q = quartet, m = multiplet, br = broad), integration, coupling constant (Hz). ^{13}C spectra were reported as chemical shifts in ppm and multiplicity where appropriate. High resolution mass spectra were obtained at Shanghai Institute of Organic Chemistry mass spectrometry facilities. All alkenes were used from commercial suppliers or prepared using standard literature procedures or the preparation procedures descried in this supporting information. All acyl chlorides were used from commercial suppliers or prepared using standard literature procedures.

General procedure for the preparation of alkenes: The alkenes were prepared according to a literature procedure from the corresponding alcohol.[1] To a solution of alcohol (5 mmol, 1.0 equiv.) in THF was added pyridine (7 mmol, 1.4 equiv). The reaction mixture was cooled to 0 ^{o}C, and allyl chloroformate (6 mmol, 1.2 equiv) was added slowly. The reaction mixture was allowed to warm to r.t. and stirred for overnight. After addition of water, the reaction mixture was extracted with ethyl acetate. The combined organic layers were washed with 1 N HCl, saturated $NaHCO_3$,

and brine, dried over $MgSO_4$, and concentrated. The residue was purified by column chromatograph to afford the product.

General procedure for the Ni-catalyzed reductive carboacylation reaction: To a flame-dried 8 mL reaction vial was charged with $NiCl_2$•glyme (0.02 mmol, 10 mol%), 4,4'-di-*tert*-butyl-2,2'-dipyridyl (dtbbpy) (0.04 mmol, 20 mol%), and Mn (0.6 mmol, 3.0 equiv.). The vial was capped. After it was evacuated and backfilled nitrogen three times, CH_3CN [0.1 M] was added via a syringe, followed by the addition of acyl chloride (0.3 mmol, 1.5 equiv.). The reaction mixture was allowed to stir for approximately 1 minute before fluoroalkyl iodide (0.2 mmol, 1.0 equiv., if liquid) and alkene (0.2 mmol, 1.0 equiv. if liquid) were added. The reaction mixture was allowed to stir at 1500 rpm for 20 h at 25 ^{o}C. The reaction was quenched with 1 N HCl, extracted with ethyl acetate (EA) three times. The combined organic layers were dried with $MgSO_4$, filtered, and concentrated in vacuo. The crude material was purified by flash chromatography to afford the product.

Note: keeping a stirring speed at 1500 rpm and reaction temperature around 25 ^{o}C is necessary for reproducibility.

General procedure for the optimization studies: To a flame-dried 8 mL reaction vial was charged with $NiCl_2$•glyme (2.2 mg, 0.01 mmol, 10 mol%), dtbbpy (5.4 mg, 0.02 mmol, 20 mol%), and Mn (16.5 mg, 0.3 mmol, 3.0 equiv.). The vial was capped. After it was evacuated and backfilled nitrogen three times, CH_3CN (1 mL) was added via a syringe, followed by the addition of 4-(*tert*-butyl)benzoyl chloride (29.5 μL, 0.15 mmol, 1.5 equiv.). The reaction mixture was allowed to stir for approximately 1 minute before C_4F_9I (17.2 μL, 0.1 mmol, 1.0 equiv.) and allyl heptanoate (19.2 μL, 0.1 mmol, 1.0 equiv.) were added. The reaction mixture was allowed to stir at 1500

rpm for 20 h at 25 °C. The reaction mixtures were analyzed by ^{1}H NMR with an internal standard.

Supplementary Table 1. Optimization studies.

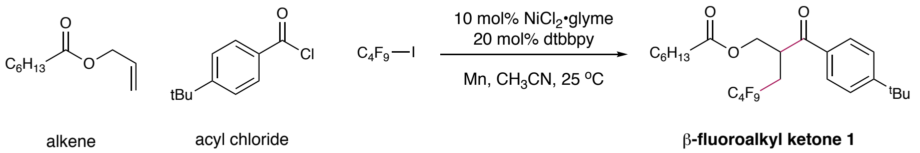

Entry	variations from standard condition	Yield of **1**
1	none	93%
2	Zn, instead of Mn	36%
3	TDAE, instead of Mn	0
4	Ni(acac)$_2$, instead of NiCl$_2$•glyme	60%
5	NiCl$_2$(PPh$_3$)$_2$, instead of NiCl$_2$•glyme	65%
6	NiCl$_2$(Py)$_4$, instead of NiCl$_2$•glyme	45%
7	DME, instead of CH$_3$CN	30%
8	dioxane, instead of CH$_3$CN	65%
9	THF, instead of CH$_3$CN	79%
10	DMA, instead of CH$_3$CN	23%
11	toluene, instead of CH$_3$CN	0
12	no NiCl$_2$•glyme	0
13	no dtbbpy	51%
14	no Mn	0

[a] Conditions: 10 mol% NiCl$_2$•DME, 20% dtbbpy, alkene (1.0 equiv.), C_4F_9I (1.0 equiv.), acyl chloride (1.5 equiv.), Mn (3.0 equiv.), CH_3CN [0.1 M], 25 °C. [b] yields were determined by ^{1}H NMR using an internal standard. dtbbpy = 4,4'-di-*tert*-butyl-2,2'-bipyridine. TDAE = tetrakis(dimethylamino)ethylene

Analytical Data of Compounds

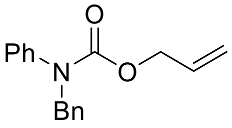

Allyl benzyl(phenyl)carbamate (S1): According to the general procedure, *N*-benzylaniline (916.3 mg, 5 mmol, 1.0 equiv.), pyridine (0.56 mL, 7 mmol, 1.40 equiv), allyl carbonochloridate (0.64 mL, 6 mmol, 1.2 equiv.) in THF (5 mL) were used. After overnight, the product was isolated by flash chromatography (PE: EA= 20:1) as a colorless oil (1.13 g, 85%); ^{1}H NMR (600 MHz, $CDCl_3$) δ 7.28 (t, *J* = 7.7

Hz, 4H), 7.26 – 7.21 (m, 3H), 7.20 (t, *J* = 7.4 Hz, 1H), 7.12 (s, 2H), 5.87 (ddd, *J* = 15.9, 10.3, 5.1 Hz, 1H), 5.14 (t, *J* = 16.4 Hz, 2H), 4.87 (s, 2H), 4.63 (d, *J* = 4.8 Hz, 2H); ^{13}C NMR (150 MHz, $CDCl_3$) δ 155.49, 137.83, 132.62, 128.82, 128.41, 127.87, 127.28, 127.04, 126.95, 126.56, 117.11, 66.25, 54.30. HRMS (ESI$^+$): calcd for $C_{17}H_{18}NO_2^+$ (M+H) 268.1332, found 268.1333.

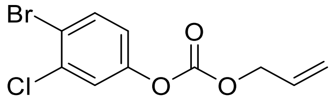

Allyl (4-bromo-3-chlorophenyl) carbonate (S2): According to the general procedure, 4-bromo-3-chlorophenol (1.04 g, 5 mmol, 1.0 equiv.), pyridine (0.56 mL, 7 mmol, 1.40 equiv), allyl carbonochloridate (0.64 mL, 6 mmol, 1.2 equiv.) in THF (5 mL) were used. After overnight, the product was isolated by flash chromatography (PE: EA= 30:1) as a pale-yellow solid (1.37 g, 94%); ^{1}H NMR (600 MHz, $CDCl_3$) δ 7.61 (d, *J* = 8.8 Hz, 1H), 7.35 (d, *J* = 2.7 Hz, 1H), 7.00 (dd, *J* = 8.8, 2.7 Hz, 1H), 5.99 (ddt, *J* = 16.4, 10.6, 5.9 Hz, 1H), 5.43 (dd, *J* = 17.2, 1.2 Hz, 1H), 5.35 (d, *J* = 10.4 Hz, 1H), 4.74 (d, *J* = 5.9 Hz, 2H); ^{13}C NMR (150 MHz, $CDCl_3$) δ 152.68, 150.35, 135.01, 134.01, 130.71, 123.29, 120.89, 119.93, 119.46, 69.53. HRMS (ESI$^+$): calcd for $C_{10}H_9BrClO_3^+$ (M+H) 290.9418, found 290.9420.

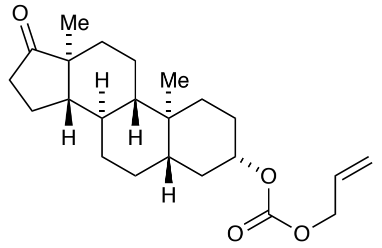

Allyl ((3R,8R,9R,10R,13R,14R)-10,13-dimethyl-17-oxohexadecahydro-1H-cyclo penta[a]phenanthren-3-yl) carbonate (S3): According to the general procedure, (3R,8R,9R,10R,13R,14R)-3-hydroxy-10,13-dimethylhexadecahydro-17H-cyclopenta[a]phenanthren-17-one (1.45 g, 5 mmol, 1.0 equiv.), pyridine (0.56 mL, 7 mmol, 1.40

equiv), allyl carbonochloridate (0.64 mL, 6 mmol, 1.2 equiv.) in THF (5 mL) were used. After overnight, the product was isolated by flash chromatography (PE: EA= 5:1) as a white solid (1.20 g, 64%); ^{1}H NMR (600 MHz, $CDCl_3$) δ 5.93 (dq, *J* = 11.0, 5.8 Hz, 1H), 5.35 (dd, *J* = 17.2, 1.1 Hz, 1H), 5.26 (d, *J* = 10.4 Hz, 1H), 4.61 (d, *J* = 5.7 Hz, 2H), 4.59 – 4.53 (m, 1H), 2.43 (dd, *J* = 19.3, 8.8 Hz, 1H), 2.11 – 2.02 (m, 1H), 1.96 – 1.88 (m, 2H), 1.84 – 1.77 (m, 3H), 1.72 (ddd, *J* = 12.0, 4.7, 2.6 Hz, 1H), 1.68 – 1.63 (m, 1H), 1.55 (ddd, *J* = 17.4, 16.0, 7.5 Hz, 3H), 1.47 (ddd, *J* = 28.4, 16.6, 7.7 Hz, 2H), 1.36 – 1.27 (m, 4H), 1.21 (dd, *J* = 12.6, 3.2 Hz, 1H), 1.07 – 0.97 (m, 2H), 0.85 (d, *J* = 8.3 Hz, 6H), 0.76 – 0.68 (m, 1H). ^{13}C NMR (150 MHz, $CDCl_3$) δ 221.13, 154.43, 131.68, 118.70, 77.51, 68.12, 54.25, 51.33, 47.73, 44.58, 36.59, 35.80, 35.57, 34.98, 33.78, 31.49, 30.76, 28.22, 27.30, 21.73, 20.44, 13.79, 12.14. HRMS (ESI+): calcd for $C_{23}H_{35}O_4^+$ (M+H) 375.2530, found 375.2517.

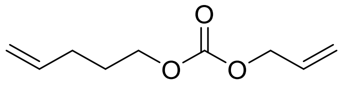

Allyl pent-4-en-1-yl carbonate (S4): According to the general procedure, pent-4-en-1-ol (0.52 mL, 5 mmol, 1.0 equiv.), pyridine (0.56 mL, 7 mmol, 1.40 equiv), allyl carbonochloridate (0.64 mL, 6 mmol, 1.2 equiv.) in THF (5 mL) were used. After overnight, the product was isolated by flash chromatography (PE: EA= 60:1) as a colorless oil (842.5 mg, 99%); ^{1}H NMR (600 MHz, $CDCl_3$) δ 5.94 (ddt, *J* = 16.2, 10.5, 5.8 Hz, 1H), 5.80 (ddt, *J* = 16.9, 10.2, 6.6 Hz, 1H), 5.37 (ddd, *J* = 17.2, 2.9, 1.5 Hz, 1H), 5.27 (ddd, *J* = 10.4, 2.4, 1.2 Hz, 1H), 5.03 (dddd, *J* = 26.4, 10.2, 3.2, 1.4 Hz, 2H), 4.63 (dt, *J* = 5.8, 1.4 Hz, 2H), 4.16 (t, *J* = 6.6 Hz, 2H), 2.15 (dd, *J* = 14.8, 6.8 Hz, 2H), 1.86 – 1.72 (m, 2H); ^{13}C NMR (150 MHz, $CDCl_3$) δ 154.99, 137.18, 131.61, 118.78, 115.41, 68.29, 67.39, 29.73, 27.78. HRMS (ESI+): calcd for $C_9H_{15}O_3^+$ (M+H) 171.1016, found 171.1009.

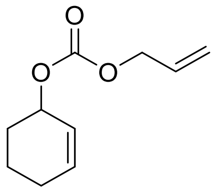

Allyl cyclohex-2-en-1-yl carbonate (S5): According to the general procedure, cyclohex-2-en-1-ol (1.04 g, 5 mmol, 1.0 equiv.), pyridine (0.56 mL, 7 mmol, 1.40 equiv), allyl carbonochloridate (0.64 mL, 6 mmol, 1.2 equiv.) in THF (5 mL) were used. After overnight, the product was isolated by flash chromatography (PE: EA= 60:1) as a colorless oil liquid (930.1 mg, 99%); ^{1}H NMR (600 MHz, $CDCl_3$) δ 5.96 (ddt, *J* = 16.4, 10.9, 4.7 Hz, 2H), 5.80 – 5.75 (m, 1H), 5.36 (d, *J* = 17.4 Hz, 1H), 5.26 (d, *J* = 10.4 Hz, 1H), 5.13 (d, *J* = 3.0 Hz, 1H), 4.63 (t, *J* = 5.3 Hz, 2H), 2.13 – 2.06 (m, 1H), 2.03 – 1.95 (m, 1H), 1.93 – 1.87 (m, 1H), 1.86 – 1.80 (m, 1H), 1.80 – 1.72 (m, 1H), 1.68 – 1.59 (m, 1H). ^{13}C NMR (150 MHz, $CDCl_3$) δ 154.69, 133.35, 131.75, 124.91, 118.71, 71.91, 68.21, 28.19, 24.83, 18.56. HRMS (EI): calcd for $C_{10}H_{14}O_3$ 182.0943, found 182.0945.

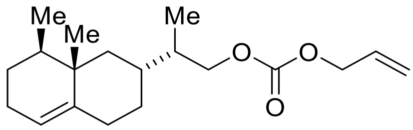

Allyl ((S)-2-((2R,8R,8aS)-8,8a-dimethyl-1,2,3,4,6,7,8,8a-octahydronaphthalen-2-yl)propyl) carbonate (S6): According to the general procedure, (S)-2-((2R,8R,8aS)-8,8a-dimethyl-1,2,3,4,6,7,8,8a-octahydronaphthalen-2-yl)propan-1-ol (200.1 mg, 0.90 mmol, 1.0 equiv.), pyridine (0.56 mL, 7 mmol, 1.40 equiv), allyl carbonochloridate (0.64 mL, 6 mmol, 1.2 equiv.) in THF (5 mL) were used. After overnight, the product was isolated by flash chromatography (PE: EA= 80:1) as a colorless oil (222.0 mg, 80%); ^{1}H NMR (600 MHz, $CDCl_3$) δ 5.99 – 5.90 (m, 1H), 5.38 – 5.34 (m, 1H), 5.33 – 5.30 (m, 1H), 5.29 – 5.25 (m, 1H), 4.64 – 4.61 (m, 2H), 4.18 – 4.12 (m, 1H), 4.01 – 3.95 (m, 1H), 2.31 – 2.20 (m, 1H), 2.11 – 1.98 (m, 2H),

1.96 – 1.88 (m, 1H), 1.82 – 1.74 (m, 1H), 1.73 – 1.64 (m, 3H), 1.44 – 1.36 (m, 3H), 1.34 – 1.23 (m, 1H), 1.07 – 0.98 (m, 1H), 0.93 – 0.85 (m, 9H). ^{13}C NMR (150 MHz, $CDCl_3$) δ 155.21, 143.24 (d, *J* = 2.8 Hz), 131.70, 119.98 (d, *J* = 4.8 Hz), 118.82 (d, *J* = 4.2 Hz), 71.43 (d, *J* = 29.3 Hz), 68.31 (d, *J* = 2.5 Hz), 42.87 (d, *J* = 273.3 Hz), 41.03 (d, *J* = 1.8 Hz), 37.72 (d, *J* = 25.1 Hz), 37.45 (d, *J* = 5.6 Hz), 34.81 (d, *J* = 29.6 Hz), 32.57 (d, *J* = 21.3 Hz), 30.79 (d, *J* = 291.9 Hz), 27.12 (d, *J* = 3.6 Hz), 25.84 (s), 18.45 (d, *J* = 1.2 Hz), 15.66 (d, *J* = 3.2 Hz), 13.49 (d, *J* = 56.5 Hz). HRMS (ESI+): calcd for $C_{19}H_{31}O_3^+$ (M+H) 307.2268, found 307.2255.

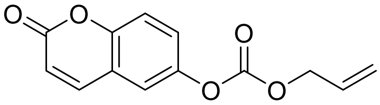

Allyl (2-oxo-2H-chromen-6-yl) carbonate (S7): According to the general procedure, 6-hydroxy-2H-chromen-2-one (810.7 mg, 5 mmol, 1.0 equiv.), pyridine (0.56 mL, 7 mmol, 1.40 equiv), allyl carbonochloridate (0.64 mL, 6 mmol, 1.2 equiv.) in THF (5 mL) were used. After overnight, the product was isolated by flash chromatography (PE: EA= 60:1) as a white solid (923.0 mg, 75%); ^{1}H NMR (600 MHz, $CDCl_3$) δ 7.70 (d, *J* = 9.6 Hz, 1H), 7.50 (d, *J* = 8.5 Hz, 1H), 7.23 (d, *J* = 2.2 Hz, 1H), 7.15 (dd, *J* = 8.5, 2.3 Hz, 1H), 6.41 (d, *J* = 9.6 Hz, 1H), 6.01 (ddt, *J* = 16.4, 10.5, 5.9 Hz, 1H), 5.46 (dd, *J* = 17.2, 1.3 Hz, 1H), 5.37 (dd, *J* = 10.4, 1.0 Hz, 1H), 4.79 – 4.73 (m, 2H); ^{13}C NMR (150 MHz, $CDCl_3$) δ 160.16, 154.61, 153.30, 152.55, 142.69, 130.67, 128.63, 119.99, 117.66, 116.77, 116.21, 109.88, 69.59. HRMS (ESI+): calcd for $C_{13}H_{11}O_5^+$ (M+H) 247.0601, found 247.0595.

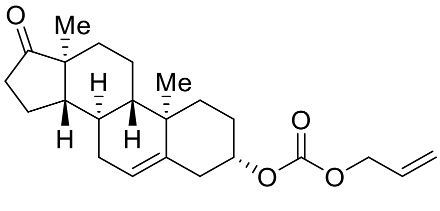

Allyl ((3S,8R,9S,10R,13S,14S)-10,13-dimethyl-17-oxo-2,3,4,7,8,9,10,11,12,13,14,

15,16,17-tetradecahydro-1H-cyclopenta[a]phenanthren-3-yl) carbonate (S8): According to the general procedure, (3S,8R,9S,10R,13S,14S)-3-hydroxy-10,13-dimethyl-1,2,3,4,7,8,9,10,11,12,13,14,15,16-tetradecahydro-17H-cyclopenta[a]phenanthren-17-one (810.7 mg, 5 mmol, 1.0 equiv.), pyridine (0.56 mL, 7 mmol, 1.40 equiv), allyl carbonochloridate (0.64 mL, 6 mmol, 1.2 equiv.) in THF (5 mL) were used. After overnight, the product was isolated by recrystallization as white solid (965.0 mg, 65%); ^{1}H NMR (600 MHz, $CDCl_3$) 5.98 – 5.90 (m, 1H), 5.43 (d, J = 5.2 Hz, 1H), 5.36 (d, J = 17.1 Hz, 1H), 5.27 (d, J = 10.2 Hz, 1H), 4.62 (d, J = 5.8 Hz, 2H), 4.52 – 4.45 (m, 1H), 2.50 – 2.43 (m, 2H), 2.42 – 2.37 (m, 1H), 2.15 – 2.04 (m, 2H), 2.00 – 1.93 (m, 2H), 1.92 – 1.83 (m, 2H), 1.72 – 1.64 (m, 4H), 1.59 – 1.47 (m, 2H), 1.32 – 1.27 (m, 2H), 1.15 (t, J = 13.8 Hz, 1H), 1.04 (s, 3H), 1.04 – 1.00 (m, 1H), 0.89 (s, 3H). ^{13}C NMR (150 MHz, $CDCl_3$) δ 220.95, 154.33, 139.62, 131.67, 122.17, 118.81, 77.65, 68.22, 51.71, 50.12, 47.52, 38.00, 36.81, 36.69, 35.84, 31.46, 31.41, 30.79, 27.67, 21.88, 20.34, 19.31, 13.56; HRMS (ESI+): calcd for $C_{23}H_{33}O_4^+$ (M+H) 373.2373, found 373.2351.

2-(4-(*tert*-Butyl)benzoyl)-4,4,5,5,6,6,7,7,7-nonafluoroheptyl heptanoate (1)

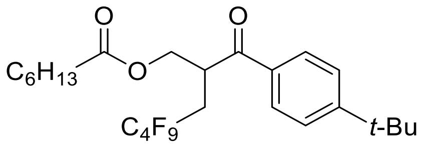

According to the general procedure, allyl heptanoate (38.5 µL, 0.20 mmol, 1.0 equiv.), C_4F_9I (34.4 µL, 0.20 mmol, 1.0 equiv.), 4-(*tert*-butyl)benzoyl chloride (58.6 µL, 0.30 mmol, 1.5 equiv.), $NiCl_2$•glyme (4.4 mg, 0.02 mmol, 0.1 eqiuv), dtbbpy (10.7 mg, 0.04 mmol, 0.2 equiv), and Mn power (33.0 mg, 0.3 mmol, 3.0 equiv) in MeCN (2 mL) were used. After 20 h, the product was isolated by flash chromatography (PE: EA= 60:1) as a pale-yellow oil (102.4 mg, 93%). ^{1}H NMR (400 MHz, $CDCl_3$) δ 7.96 (d, J = 8.6 Hz, 2H), 7.52 (d, J = 8.6 Hz, 2H), 4.42 (dd, J = 10.8, 6.1 Hz, 1H), 4.29 –

4.20 (m, 1H), 4.15-4.17 (m, 1H), 3.10 – 2.90 (m, 1H), 2.41 – 2.26 (m, 1H), 2.22 (t, *J* = 7.6 Hz, 2H), 1.57 – 1.47 (m, 2H), 1.35 (s, 9H), 1.29 – 1.21 (m, 6H), 0.87 (t, *J* = 6.9 Hz, 3H). ^{19}F NMR (376 MHz, $CDCl_3$) δ -81.05 (t, *J* = 9.5 Hz, 3F), -112.25 – -112.71 (m, 2F), -124.16 – -124.57 (m, 2F), -125.80 – -126.13 (m, 2F). ^{13}C NMR (100 MHz, $CDCl_3$) δ 197.41, 173.43, 157.86, 133.02, 128.55, 125.93, 64.41, 38.25, 35.22, 34.01, 31.38, 31.02, 29.41 (t, *J* = 21.4 Hz), 28.70, 24.69, 22.42, 13.97. HRMS (ESI+): calcd for $C_{25}H_{32}F_9O_3^+$ (M+H) 551.2202, found 551.2199.

2-(4-(*tert*-Butyl)benzoyl)-4,4,5,5,6,6,7,7,7-nonafluoroheptyl benzoate (2)

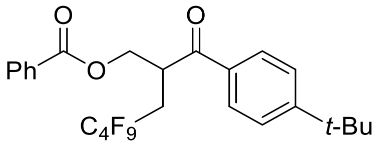

According to the general procedure, allyl benzoate (30.9 μL, 0.20 mmol, 1.0 equiv.), C_4F_9I (34.4 μL, 0.20 mmol, 1.0 equiv.), 4-(*tert*-butyl)benzoyl chloride (58.6 μL, 0.30 mmol, 1.5 equiv.), $NiCl_2$•glyme (4.4 mg, 0.02 mmol, 0.1 eqiuv), dtbbpy (10.7 mg, 0.04 mmol, 0.2 equiv), and Mn power (33.0 mg, 0.3 mmol, 3.0 equiv) in MeCN (2 mL) were used. After 20 h, the product was isolated by flash chromatography (PE: EA= 60:1) as a White solid (96.5 mg, 89%). ^{1}H NMR (600 MHz, $CDCl_3$) δ 8.04 (d, *J* = 8.4 Hz, 2H), 7.86 (d, *J* = 7.5 Hz, 2H), 7.56 – 7.54 (m, 3H), 7.40 – 7.37 (m, 2H), 4.64 – 4.62 (m, 1H), 4.49 – 4.44 (m, 2H), 3.19 – 3.09 (m, 1H), 2.49 – 2.40 (m, 1H), 1.36 (s, 9H); ^{19}F NMR (564 MHz, $CDCl_3$) δ -81.07 (t, *J* = 8.3 Hz, 3F), -109.51 – -115.82 (m, 2F), -124.29 (s, 2F), -125.95 (d, *J* = 10.2 Hz, 2F); ^{13}C NMR (150 MHz, $CDCl_3$) δ 197.55, 165.96, 157.84, 133.28, 133.15, 129.54, 129.22, 128.56, 128.35, 125.91, 65.31, 38.17, 35.16, 30.97, 29.51 (t, *J* = 21.4 Hz). HRMS (ESI+): calcd for $C_{25}H_{24}F_9O_3^+$ (M+H) 543.1576, found 543.1578.

2-(4-(*tert*-Butyl)benzoyl)-4,4,5,5,6,6,7,7,7-nonafluoroheptyl acetate (3)

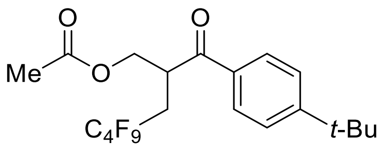

According to the general procedure, allyl acetate (21.6 μL, 0.20 mmol, 1.0 equiv.), C_4F_9I (34.4 μL, 0.20 mmol, 1.0 equiv.), 4-(*tert*-butyl)benzoyl chloride (58.6 μL, 0.30 mmol, 1.5 equiv.), $NiCl_2$•glyme (4.4 mg, 0.02 mmol, 0.1 eqiuv), dtbbpy (10.7 mg, 0.04 mmol, 0.2 equiv), and Mn power (33.0 mg, 0.3 mmol, 3.0 equiv) in MeCN (2 mL) were used. After 23 h, the product was isolated by flash chromatography (PE: EA= 60:1) as a White solid (81.4 mg, 85%); ^{1}H NMR (600 MHz, $CDCl_3$) δ 7.98 (d, *J* = 8.4 Hz, 2H), 7.55 (d, *J* = 8.4 Hz, 2H), 4.46-4.40 (m, 1H), 4.29 – 4.22 (m, 1H), 4.18-4.15 (m, 1H), 3.09 – 2.93 (m, 1H), 2.43 – 2.28 (m, 1H), 2.01 (s, 3H), 1.37 (s, 9H). ^{19}F NMR (376 MHz, $CDCl_3$) δ -81.07 (t, *J* = 9.5 Hz, 3F), -112.28 – -112.73 (m, 2F), -124.32 (d, *J* = 5.6 Hz, 2F), -125.81 – -126.08 (m, 2F). ^{13}C NMR (150 MHz, $CDCl_3$) δ 197.32, 170.58, 157.88, 132.96, 128.51, 125.93, 64.54, 38.22, 35.21, 30.99, 29.44 (t, *J* = 21.4 Hz), 20.56. HRMS (ESI+): calcd for $C_{20}H_{22}F_9O_3^+$ (M+H) 481.1420, found 481.1423.

2-(4-(*tert*-Butyl)benzoyl)-4,4,5,5,6,6,7,7,7-nonafluoroheptyl 2-phenoxyacetate (4)

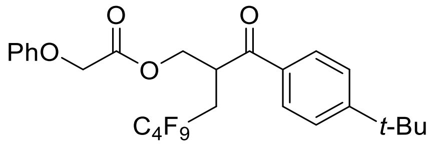

According to the general procedure, allyl 2-phenoxyacetate (34.9 μL, 0.20 mmol, 1.0 equiv.), C_4F_9I (34.4 μL, 0.20 mmol, 1.0 equiv.), 4-(*tert*-butyl)benzoyl chloride (58.6 μL, 0.30 mmol, 1.5 equiv.), $NiCl_2$•glyme (4.4 mg, 0.02 mmol, 0.1 eqiuv), dtbbpy (10.7 mg, 0.04 mmol, 0.2 equiv), and Mn power (33.0 mg, 0.3 mmol, 3.0 equiv) in MeCN (4 mL) were used. After 36 h, the product was isolated by flash chromatography (PE: EA= 20:1) as a pale-yellow oil liquid (82.1 mg, 72%); ^{1}H NMR (400 MHz, $CDCl_3$) δ 7.94 (d, *J* = 8.4 Hz, 2H), 7.52 (d, *J* = 8.4 Hz, 2H), 7.27 (t, *J* = 7.9

Hz, 2H), 6.99 (t, J = 7.3 Hz, 1H), 6.83 (d, J = 8.4 Hz, 2H), 4.55 (d, J = 4.1 Hz, 2H), 4.54 – 4.50 (m, 1H), 4.31 – 4.22 (m, 2H), 3.06 – 2.80 (m, 1H), 2.38 – 2.20 (m, 1H), 1.34 (s, 9H). ^{19}F NMR (376 MHz, $CDCl_3$) δ -81.00 (t, J = 9.6 Hz, 3F), -110.41 – -113.54 (m, 2F), -124.22 (s, 2F), -125.80 – -126.12 (m, 2F). ^{13}C NMR (101 MHz, $CDCl_3$) δ 196.95, 168.69, 158.04, 157.58, 132.84, 129.60, 128.55, 126.01, 121.91, 114.49, 65.13, 64.96, 38.15, 35.23, 30.99, 29.48 (t, J = 21.7 Hz). HRMS (ESI+): calcd for $C_{26}H_{26}F_9O_4^+$ (M+H) 573.1682, found 573.1688.

2-(4-(*tert*-Butyl)benzoyl)-4,4,5,5,6,6,7,7,7-nonafluoroheptyl pivalate (5)

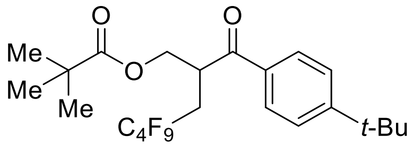

According to the general procedure, allyl pivalate (34.2 μL, 0.20 mmol, 1.0 equiv.), C_4F_9I (34.4 μL, 0.20 mmol, 1.0 equiv.), 4-(*tert*-butyl)benzoyl chloride (58.6 μL, 0.30 mmol, 1.5 equiv.), $NiCl_2$•glyme (4.4 mg, 0.02 mmol, 0.1 eqiuv), dtbbpy (10.7 mg, 0.04 mmol, 0.2 equiv), and Mn power (33.0 mg, 0.3 mmol, 3.0 equiv) in MeCN (2 mL) were used. After 20 h, the product was isolated by flash chromatography (PE: EA= 40:1) as a pale-yellow liquid (74.1 mg, 71%); ^{1}H NMR (600 MHz, $CDCl_3$) δ 7.98 (d, J = 8.4 Hz, 2H), 7.53 (d, J = 8.4 Hz, 2H), 4.42 – 4.39 (m, 1H), 4.29 – 4.26 (m, 1H), 4.10 - 4.07 (m, 1H), 3.09 – 2.98 (m, 1H), 2.36 – 2.27 (m, 1H), 1.35 (s, 9H), 1.09 (s, 9H); ^{19}F NMR (564 MHz, $CDCl_3$) δ -81.08 (t, J = 9.6 Hz, 3F), -112.42 – -113.06 (m, 2F), -124.46 (d, J = 3.0 Hz, 2F), -125.84 – -126.16 (m, 2F). ^{13}C NMR (100 MHz, $CDCl_3$) δ 197.43, 178.08, 157.86, 133.15, 128.56, 125.92, 64.62, 38.74, 38.26, 35.20, 31.00, 29.31 (t, J = 21.3 Hz), 26.88. HRMS (ESI+): calcd for $C_{23}H_{28}F_9O_3^+$ (M+H) 523.1889, found 523.1891.

2-(4-(*tert*-Butyl)benzoyl)-4,4,5,5,6,6,7,7,7-nonafluoroheptyl benzyl(phenyl)carba

mate (6)

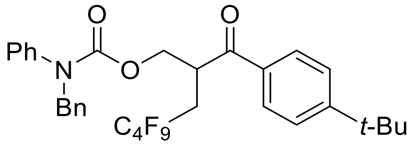

According to the general procedure, **S1** (46.5 μL, 0.20 mmol, 1.0 equiv.), C_4F_9I (34.4 μL, 0.20 mmol, 1.0 equiv.), 4-(*tert*-butyl)benzoyl chloride (58.6 μL, 0.30 mmol, 1.5 equiv.), $NiCl_2$•glyme (4.4 mg, 0.02 mmol, 0.1 eqiuv), dtbbpy (10.7 mg, 0.04 mmol, 0.2 equiv), and Mn power (33.0 mg, 0.3 mmol, 3.0 equiv) in MeCN (2 mL) were used. After 20 h, the product was isolated by flash chromatography (PE: EA= 20:1) as a pale-yellow liquid (108.9 mg, 84%); ^{1}H NMR (600 MHz, $CDCl_3$) δ 7.95 (d, *J* = 8.5 Hz, 2H), 7.50 (d, *J* = 8.5 Hz, 2H), 7.33 – 7.25 (m, 4H), 7.20 – 7.11 (m, 5H), 6.85 – 6.84 (m, 1H), 4.82 – 4.77 (m, 2H), 4.49 – 4.47 (m, 1H), 4.34 – 4.29 (m, 1H), 4.17 – 4.13 (m, 1H), 2.92 – 2.90 (m, 1H), 2.25 – 2.10 (m, 1H), 1.38 (s, 9H);); ^{19}F NMR (564 MHz, $CDCl_3$) δ -81.05 (t, *J* = 9.4 Hz, 3F), -111.83 – -113.22 (m, 2F), -124.42 (s, 2F), -125.86 – -126.08 (m, 2F); ^{13}C NMR (100 MHz, $CDCl_3$) δ 197.56, 157.60, 154.98, 137.30, 133.08, 128.86, 128.51, 128.43, 128.24, 128.20, 127.42, 127.20, 126.97, 125.85, 66.04, 54.39, 38.39, 35.14, 30.98, 29.22 (t, *J* = 21.3 Hz). HRMS (ESI+): calcd for $C_{32}H_{31}F_9NO_3^+$ (M+H) 648.2155, found 648.2151.

2-(2-(4-(*tert*-Butyl)benzoyl)-4,4,5,5,6,6,7,7,7-nonafluoroheptyl)isoindoline-1,3-dione (7)

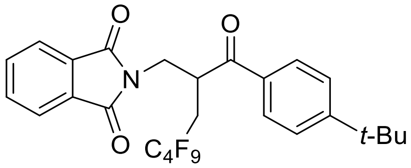

According to the general procedure, 2-allylisoindoline-1,3-dione (37.5 mg, 0.20 mmol, 1.0 equiv.), C_4F_9I (34.4 μL, 0.20 mmol, 1.0 equiv.), 4-(*tert*-butyl)benzoyl chloride (58.6 μL, 0.30 mmol, 1.5 equiv.), $NiCl_2$•glyme (4.4 mg, 0.02 mmol, 0.1 eqiuv), dtbbpy (10.7 mg, 0.4 mmol, 0.02 equiv), and Mn power (33.0 mg, 0.3 mmol, 3.0

equiv) in MeCN (2 mL) were used. After 20 h, the product was isolated by flash chromatography (PE: EA= 6:1) as a pale-yellow solid (54.4 mg, 49%); ^{1}H NMR (600 MHz, $CDCl_3$) δ 8.03 (d, *J* = 8.4 Hz, 2H), 7.82 – 7.81 (m, 2H), 7.72 – 7.71 (m, 2H), 7.47 (d, *J* = 8.4 Hz, 2H), 4.44 – 4.41 (m, 1H), 3.99 – 3.96 (m, 1H), 3.90 – 3.86 (m, 1H), 3.24 – 3.14 (m, 1H), 2.33 – 2.24 (m, 1H), 1.29 (s, 9H); ^{19}F NMR (564 MHz, $CDCl_3$) δ -81.09 (t, *J* = 9.4 Hz, 3F), -111.70 – -113.53 (m, 2F), -124.28 – -124.35 (m, 2F), -126.01 (t, *J* = 12.7 Hz, 2F); ^{13}C NMR (100 MHz, $CDCl_3$) δ 197.53, 167.97, 157.71, 134.21, 132.92, 131.56, 128.60, 125.81, 123.43, 40.15, 37.88, 35.10, 30.93, 30.03 (t, *J* = 21.6 Hz). HRMS (ESI+): calcd for $C_{26}H_{23}F_9NO_3^+$ (M+H) 568.1529, found 568.1527.

2-(4-(*tert*-Butyl)benzoyl)-4,4,5,5,6,6,7,7,7-nonafluoroheptyl diethyl phosphate (8)

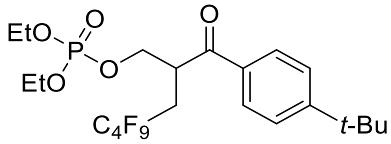

According to the general procedure, allyl diethyl phosphate (35.6 µL, 0.20 mmol, 1.0 equiv.), C_4F_9I (34.4 µL, 0.20 mmol, 1.0 equiv.), 4-(*tert*-butyl)benzoyl chloride (58.6 µL, 0.30 mmol, 1.5 equiv.), $NiCl_2$•glyme (4.4 mg, 0.02 mmol, 0.1 eqiuv), dtbbpy (10.7 mg, 0.04 mmol, 0.2 equiv), and Mn power (33.0 mg, 0.3 mmol, 3.0 equiv) in MeCN (4 mL) were used. After 36 h, the product was isolated by flash chromatography (PE: EA= 5:1) as a brown liquid (61.2 mg, 54%); ^{1}H NMR (600 MHz, $CDCl_3$) δ 7.97 (d, *J* = 8.4 Hz, 2H), 7.53 (d, *J* = 8.4 Hz, 2H), 4.36 – 4.27 (m, 2H), 4.13 – 4.07 (m, 3H), 4.04 – 3.98 (m, 2H), 3.01 – 2.90 (m, 1H), 2.47 – 2.38 (m, 1H), 1.35 (s, 9H), 1.30 (t, *J* = 7.2 Hz, 3H), 1.25 (t, *J* = 7.2 Hz, 3H); ^{19}F NMR (564 MHz, $CDCl_3$) δ -81.10 (t, *J* = 9.4 Hz, 3F), -111.59 – -113.30 (m, 2F), -124.36 – -124.49 (m, 2F), -125.99 (s, 2F). ^{13}C NMR (100 MHz, $CDCl_3$) δ 197.07, 157.90, 132.91, 128.54,

125.90, 67.20, 64.06, 39.56, 35.17, 30.94, 29.15 (t, J = 21.3 Hz), 15.92. HRMS (ESI+): calcd for $C_{22}H_{29}F_9O_5P^+$ (M+H) 575.1603, found 575.1601.

1-(4-(*tert*-Butyl)phenyl)-4,4,5,5,6,6,7,7,7-nonafluoro-2-(2-(phenylsulfonyl)ethyl)heptan-1-one (9)

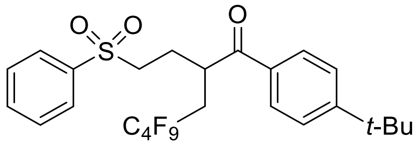

According to the general procedure, (but-3-en-1-ylsulfonyl)benzene (33.5 μL, 0.20 mmol, 1.0 equiv.), C_4F_9I (34.4 μL, 0.20 mmol, 1.0 equiv.), 4-(*tert*-butyl)benzoyl chloride (58.6 μL, 0.30 mmol, 1.5 equiv.), $NiCl_2$•glyme (8.8 mg, 0.04 mmol, 0.2 eqiuv), dtbbpy (21.5 mg, 0.08 mmol, 0.4 equiv), and Mn power (33.0 mg, 0.3 mmol, 3.0 equiv) in MeCN (2 mL) were used. After 20 h, the product was isolated by flash chromatography (PE: EA= 10:1) as a pale-yellow liquid (47.2 mg, 42%); ^{1}H NMR (600 MHz, $CDCl_3$) δ 7.89 (d, J = 8.5 Hz, 2H), 7.85 – 7.81 (m, 2H), 7.66 (t, J = 7.5 Hz, 1H), 7.59 – 7.46 (m, 4H), 4.16 – 4.12 (m, 1H), 3.08 – 2.96 (m, 2H), 2.88 – 2.75 (m, 1H), 2.25 – 2.17 (m, 1H), 2.14 – 2.03 (m, 2H), 1.36 (s, 9H); ^{19}F NMR (564 MHz, $CDCl_3$) δ -81.02 (t, J = 9.6 Hz, 3F), -112.24 – -112.57 (m, 2F), -124.31 (s, 2F), -125.78 – -126.06 (m, 2F). ^{13}C NMR (150 MHz, $CDCl_3$) δ 199.19, 158.15, 138.54, 133.98, 132.59, 129.40, 128.50, 128.00, 126.09, 52.95, 36.13, 35.25, 32.40, 32.26 (t, J = 21.4 Hz), 25.88. HRMS (ESI+): calcd for $C_{25}H_{26}F_9O_3S^+$ (M+H) 577.1453, found 577.1456.

1-(4-(*tert*-Butyl)phenyl)-4,4,5,5,6,6,7,7,7-nonafluoro-2-phenoxyheptan-1-one (10)

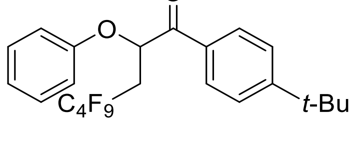

According to the general procedure, (vinyloxy)benzene (24.6 μL, 0.20 mmol, 1.0 equiv.), C_4F_9I (34.4 μL, 0.20 mmol, 1.0 equiv.), 4-(tert-butyl)benzoyl chloride (58.6 μL, 0.30 mmol, 1.5 equiv.), $NiCl_2$•glyme (4.4 mg, 0.02 mmol, 0.1 eqiuv), dtbbpy (10.7 mg, 0.04 mmol, 0.2 equiv), and Mn power (33.0 mg, 0.3 mmol, 3.0 equiv) in MeCN (2 mL) were used. After 20 h, the product was isolated by flash chromatography (PE: EA= 100:1) as a pale-yellow liquid (63.2 mg, 63%); ^{1}H NMR (600 MHz, $CDCl_3$) δ 8.06 – 8.00 (m, 2H), 7.57 – 7.52 (m, 2H), 7.33 – 7.26 (m, 2H), 7.02 (t, *J* = 7.4 Hz, 1H), 6.93 (d, *J* = 8.2 Hz, 2H), 5.84 - 5.82 (m, 1H), 2.98 – 2.77 (m, 2H), 1.37 (s, 9H); ^{19}F NMR (564 MHz, $CDCl_3$) δ -81.00 (t, *J* = 9.5 Hz, 3F), -112.70 (s, 2F), -124.25 (s, 2F), -125.81 – -125.97 (m, 2F); ^{13}C NMR (150 MHz, $CDCl_3$) δ 194.51, 158.39, 156.69, 130.81, 129.73, 128.84, 126.11, 122.22, 115.38, 72.86, 35.29, 33.41 (t, *J* = 21.4 Hz), 30.94. HRMS (ESI+): calcd for $C_{23}H_{22}F_9O_2^+$ (M+H) 501.1471, found 501.1471.

1-(4-(*tert*-Butyl)phenyl)-4,4,5,5,6,6,7,7,7-nonafluoro-2-(4-methoxyphenoxy)heptan-1-one (11)

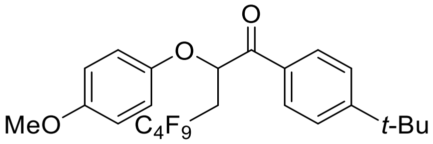

According to the general procedure, 1-methoxy-4-(vinyloxy)benzene (30.6 μL, 0.20 mmol, 1.0 equiv.), C_4F_9I (34.4 μL, 0.20 mmol, 1.0 equiv.), 4-(*tert*-butyl)benzoyl chloride (58.6 μL, 0.30 mmol, 1.5 equiv.), $NiCl_2$•glyme (4.4 mg, 0.02 mmol, 0.1 eqiuv), dtbbpy (10.7 mg, 0.04 mmol, 0.2 equiv), and Mn power (33.0 mg, 0.3 mmol, 3.0 equiv) in MeCN (2 mL) were used. After 20 h, the product was isolated by flash chromatography (PE: EA= 30:1) as a pale-yellow liquid (80.6 mg, 76%); ^{1}H NMR (600 MHz, $CDCl_3$) δ 8.00 (d, *J* = 8.4 Hz, 2H), 7.51 (d, *J* = 8.4 Hz, 2H), 6.86 – 6.78 (m, 4H), 5.70 – 5.68 (m, 1H), 3.73 (s, 3H), 2.89 – 2.74 (m, 2H), 1.34 (s, 9H); ^{19}F NMR

(564 MHz, $CDCl_3$) δ -81.02 (t, J = 9.2 Hz, 3F), -111.93 – -113.49 (m, 2F), -124.27 (s, 2F), -125.90 (s, 2F); ^{13}C NMR (150 MHz, $CDCl_3$) δ 194.88, 158.29, 154.88, 150.84, 130.97, 128.86, 126.05, 116.89, 114.76, 73.96, 55.57, 35.27, 33.42 (t, J = 21.4 Hz), 30.94. HRMS (ESI+): calcd for $C_{24}H_{24}F_9O_3^+$ (M+H) 531.1576, found 531.1579.

2-(4-(*tert*-Butyl)benzoyl)-4,4,5,5,6,6,7,7,7-nonafluoroheptyl methyl carbonate (12)

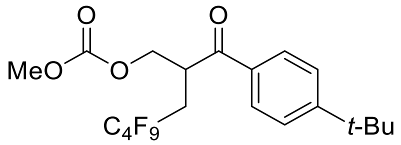

According to the general procedure, allyl methyl carbonate (22.6 μL, 0.20 mmol, 1.0 equiv.), C_4F_9I (34.4 μL, 0.20 mmol, 1.0 equiv.), 4-(*tert*-butyl)benzoyl chloride (58.6 μL, 0.30 mmol, 1.5 equiv.), $NiCl_2$•glyme (4.4 mg, 0.02 mmol, 0.1 eqiuv), dtbbpy (10.7 mg, 0.04 mmol, 0.2 equiv), and Mn power (33.0 mg, 0.3 mmol, 3.0 equiv) in MeCN (2 mL) were used. After 20 h, the product was isolated by flash chromatography (PE: EA= 20:1) as a pale-yellow oil (93.2 mg, 94%); ^{1}H NMR (600 MHz, $CDCl_3$) δ 7.97 (d, J = 8.4 Hz, 2H), 7.53 (d, J = 8.4 Hz, 2H), 4.50 – 4.47 (m, 1H), 4.29 – 4.27 (m, 1H), 4.17 – 4.14 (m, 1H), 3.77 (s, 3H), 3.04 – 2.94 (m, 1H), 2.43 – 2.34 (m, 1H), 1.35 (s, 9H); ^{19}F NMR (564 MHz, $CDCl_3$) δ -81.07 (t, J = 9.6 Hz, 3F), -111.80 – -113.32 (m, 2F), -124.31 (s, 2F), -125.92 – -126.03 (m, 2F). ^{13}C NMR (100 MHz, $CDCl_3$) δ 196.83, 157.97, 155.29, 132.78, 128.55, 125.97, 67.55, 55.08, 38.33, 35.22, 30.99, 29.39 (t, J = 21.4 Hz). HRMS (ESI+): calcd for $C_{20}H_{22}F_9O_4^+$ (M+H) 497.1369, found 497.1374.

4-Bromo-3-chlorophenyl (2-(4-(*tert*-butyl)benzoyl)-4,4,5,5,6,6,7,7,7-nonafluoro-heptyl) carbonate (13)

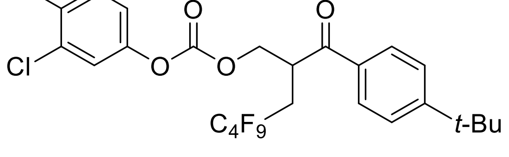

According to the general procedure, **S2** (55.5 mg, 0.20 mmol, 1.0 equiv.), C_4F_9I (34.4 μL, 0.20 mmol, 1.0 equiv.), 4-(*tert*-butyl)benzoyl chloride (58.6 μL, 0.30 mmol, 1.5 equiv.), $NiCl_2$•glyme (4.4 mg, 0.02 mmol, 0.1 eqiuv), dtbbpy (10.7 mg, 0.04 mmol, 0.2 equiv), and Mn power (33.0 mg, 0.3 mmol, 3.0 equiv) in MeCN (2 mL) were used. After 22 h, the product was isolated by flash chromatography (PE: EA= 15:1) as a pale-yellow solid (99.5 mg, 74%); ^{1}H NMR (600 MHz, $CDCl_3$) δ 8.02 (d, *J* = 8.4 Hz, 2H), 7.62 (d, *J* = 8.4 Hz, 1H), 7.57 (d, *J* = 8.4 Hz, 2H), 7.28 (s, 1H), 6.95 – 6.93 (m, 1H), 4.64 – 4.61 (m, 1H), 4.44 – 4.33 (m, 2H), 3.12 – 2.97 (m, 1H), 2.49 – 2.41 (m, 1H), 1.38 (s, 9H); ^{19}F NMR (564 MHz, $CDCl_3$) δ -81.06 (t, *J* = 9.5 Hz, 3F), -112.33 – -112.51 (m, 2F), -124.26 (s, 2F), -125.87 – -126.05 (m, 2F); ^{13}C NMR (150 MHz, $CDCl_3$) δ 194.09, 155.69, 150.00, 147.55, 132.60, 131.56, 130.19, 126.05, 123.54, 120.64, 118.22, 117.27, 66.06, 35.45, 32.70, 28.42, 26.98 (t, *J* = 21.7 Hz). HRMS (ESI+): calcd for $C_{25}H_{22}BrClF_9O_4^+$ (M+H) 671.0241, found 671.0232.

2-(4-(*tert*-Butyl)benzoyl)-4,4,5,5,6,6,7,7,7-nonafluoroheptyl (1,7,7-trimethyl-bicycle[2.2.1]heptan-2-yl) carbonate (14)

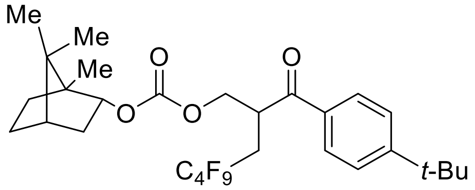

According to the general procedure, allyl ((1S,2R,4S)-1,7,7-trimethylbicyclo[2.2.1]heptan-2-yl) carbonate (38.1 μL, 0.20 mmol, 1.0 equiv.), C_4F_9I (34.4 μL, 0.20 mmol, 1.0 equiv.), 4-(*tert*-butyl)benzoyl chloride (58.6 μL, 0.30 mmol, 1.5 equiv.), $NiCl_2$•glyme (4.4 mg, 0.02 mmol, 0.1 eqiuv), dtbbpy (10.7 mg, 0.04 mmol, 0.2 equiv), and Mn power (33.0 mg, 0.3 mmol, 3.0 equiv) in MeCN (2 mL) were used. After 20 h, the product was isolated by flash chromatography (PE: EA= 30:1) as a pale-yellow liquid (95.3 mg, 77%); ^{1}H NMR (600 MHz, $CDCl_3$) δ 8.00 – 7.98 (m, 2H), 7.56 – 7.49 (m, 2H), 4.81 – 4.76 (m, 1H),

4.48 -4.44 (m, 1H), 4.38 – 4.28 (m, 1H), 4.14 – 4.09 (m, 1H), 3.04 – 2.96 m, 1H), 2.46 – 2.28 (m, 2H), 1.89 – 1.81 (m, 1H), 1.76 – 1.69 (m, 1H), 1.67 (t, *J* = 4.5 Hz, 1H), 1.35 (s, 9H), 1.28 – 1.17 (m, 2H), 1.02 – 0.99 (m, 1H), 0.88 – 0.86 (m, 6H), 0.83 – 0.82 (m, 3H); ^{19}F NMR (564 MHz, $CDCl_3$) δ -81.02 (t, *J* = 9.5 Hz, 3F), -111.58 – -113.05 (m, 2F), -124.27 (s, 2F), -125.87 – -126.01 (m, 2F); ^{13}C NMR (150 MHz, $CDCl_3$) δ 197.06, 157.89, 155.10, 132.92, 128.58, 125.96, 84.56, 67.31, 48.89, 47.94, 44.68, 38.34, 36.28, 35.22, 31.00, 29.44 (t, *J* = 21.6 Hz), 27.84, 26.74, 19.64, 18.77, 13.32. HRMS (ESI+): calcd for $C_{29}H_{39}F_9NO_4^+$ (M+NH_4) 636.2730, found 636.2721.

2-(4-(*tert*-Butyl)benzoyl)-4,4,5,5,6,6,7,7,7-nonafluoroheptyl (10,13-dimethyl-17-oxohexadecahydro-1H-cyclopenta[a]phenanthren-3-yl) carbonate (15)

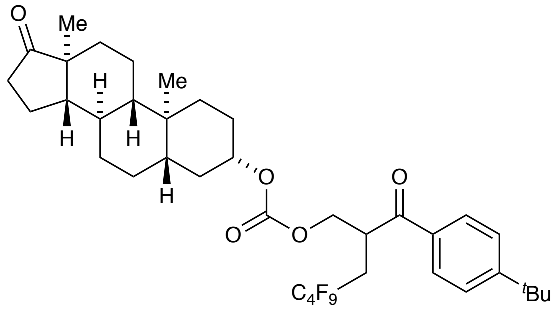

According to the general procedure, **S3** (74.8 mg, 0.20 mmol, 1.0 equiv.), C_4F_9I (34.4 μL, 0.20 mmol, 1.0 equiv.), 4-(*tert*-butyl)benzoyl chloride (58.6 μL, 0.30 mmol, 1.5 equiv.), $NiCl_2$•glyme (4.4 mg, 0.02 mmol, 0.1 eqiuv), dtbbpy (10.7 mg, 0.04 mmol, 0.2 equiv), and Mn power (33.0 mg, 0.3 mmol, 3.0 equiv) in MeCN (2 mL) were used. After 20 h, the product was isolated by flash chromatography (PE: EA= 6:1) as a pale-yellow solid (101.2 mg, 67%); ^{1}H NMR (600 MHz, $CDCl_3$) δ 7.98 (d, *J* = 8.2 Hz, 2H), 7.53 (d, *J* = 8.0 Hz, 2H), 4.58 – 4.50 (m, 1H), 4.49 – 4.43 (m, 1H), 4.34 – 4.27 (m, 1H), 4.17 – 4.09 (m, 1H), 3.08 – 2.94 (m, 1H), 2.47 – 2.33 (m, 2H), 2.11 – 2.03 (m, 1H), 1.96 – 1.73 (m, 6H), 1.69 – 1.63 (m, 2H), 1.56 – 1.48 (m, 3H), 1.41 – 1.37 (m, 1H), 1.35 (s, 9H), 1.32 – 1.24 (m, 4H), 1.21 – 1.14 (m, 1H), 1.05 – 0.97 (m, 2H),

0.86 (s, 3H), 0.83 (s, 3H), 0.74 – 0.67 (m, 1H). ^{19}F NMR (564 MHz, $CDCl_3$) δ -80.97 – -81.17 (m, 3F), -111.55 – -113.36 (m, 2F), -124.28 (s, 2F), -125.87 – -126.10 (m, 3F). ^{13}C NMR (150 MHz, $CDCl_3$) δ 221.07, 196.88, 157.87, 154.18, 132.81, 128.53, 125.93, 78.12, 67.20, 54.23, 51.32, 47.72, 44.55, 38.30, 36.53, 35.78, 35.53, 35.18, 34.97, 33.62, 31.47, 30.98, 30.73, 29.37 (t, *J* = 20.5 Hz), 28.18, 27.14, 21.72, 20.43, 13.76, 12.10. HRMS (ESI+): calcd for $C_{38}H_{48}F_9O_5^+$ (M+H) 755.3353, found 755.3372.

2-Benzoyl-4,4,5,5,6,6,7,7,7-nonafluoroheptyl heptanoate (16)

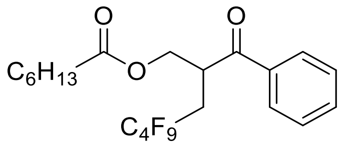

According to the general procedure, allyl heptanoate (38.5 μL, 0.20 mmol, 1.0 equiv.), C_4F_9I (34.4 μL, 0.20 mmol, 1.0 equiv.), benzoyl chloride (34.8 μL, 0.30 mmol, 1.5 equiv.), $NiCl_2$•glyme (4.4 mg, 0.02 mmol, 0.1 eqiuv), dtbbpy (10.7 mg, 0.04 mmol, 0.2 equiv), and Mn power (33.0 mg, 0.3 mmol, 3.0 equiv) in MeCN (2 mL) were used. After 22 h, the product was isolated by flash chromatography (PE: EA= 60:1) as a pale-yellow oil liquid (80.1 mg, 81%); ^{1}H NMR (600 MHz, $CDCl_3$) δ 8.04 (d, *J* = 7.7 Hz, 2H), 7.65 (t, *J* = 7.4 Hz, 1H), 7.54 (t, *J* = 7.2 Hz, 2H), 4.45-4.41 (m, 1H), 4.31-4.25 (m, 1H), 4.19-4.14 (m, 1H), 3.08 – 2.95 (m, 1H), 2.41 – 2.32 (m, 1H), 2.24 (t, *J* = 7.8 Hz, 2H), 1.56 – 1.50 (m, 2H), 1.30 – 1.24 (m, 6H), 0.89 (t, *J* = 7.0 Hz, 3H). ^{19}F NMR (564 MHz, $CDCl_3$) δ -81.03 (t, *J* = 9.5 Hz, 3F), -112.43 (dd, *J* = 26.1, 13.1 Hz, 2F), -124.33 (s, 2F), -125.62 – -126.25 (m, 2F). ^{13}C NMR (150 MHz, $CDCl_3$) δ 198.03, 173.37, 135.68, 133.95, 128.96, 128.53, 64.37, 38.30, 33.99, 31.37, 29.48 (t, *J* = 21.3 Hz), 28.69, 24.68, 22.42, 14.00. HRMS (ESI+): calcd for $C_{21}H_{24}F_9O_3^+$ (M+H) 495.1576, found 495.1581.

4,4,5,5,6,6,7,7,7-Nonafluoro-2-(4-methylbenzoyl)heptyl heptanoate (17)

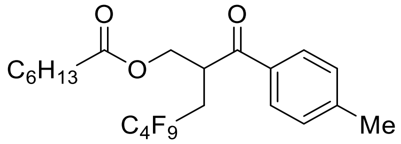

According to the general procedure, allyl heptanoate (38.5 μL, 0.20 mmol, 1.0 equiv.), C_4F_9I (34.4 μL, 0.20 mmol, 1.0 equiv.), 4-methylbenzoyl chloride (39.7 μL, 0.30 mmol, 1.5 equiv.), $NiCl_2$•glyme (4.4 mg, 0.02 mmol, 0.1 eqiuv), dtbbpy (10.7 mg, 0.04 mmol, 0.2 equiv), and Mn power (33.0 mg, 0.3 mmol, 3.0 equiv) in MeCN (2 mL) were used. After 20 h, the product was isolated by flash chromatography (PE: EA= 50:1) as a colorless oil (74.1 mg, 73%); ^{1}H NMR (400 MHz, $CDCl_3$) δ 7.92 (d, *J* = 8.2 Hz, 2H), 7.31 (d, *J* = 8.0 Hz, 2H), 4.43-4.36 (m, 1H), 4.27 – 4.19 (m, 1H), 4.16-4.10 (m, 1H), 3.09 – 2.87 (m, 1H), 2.43 (s, 3H), 2.38 – 2.27 (m, 1H), 2.22 (t, *J* = 7.6 Hz, 2H), 1.56 – 1.45 (m, 2H), 1.28 – 1.20 (m, 6H), 0.87 (t, *J* = 6.9 Hz, 3H). ^{19}F NMR (376 MHz, $CDCl_3$) δ -81.06 (t, *J* = 9.5 Hz, 3F), -112.02 – -112.93 (m, 2F), -124.17 – -124.58 (m, 2F), -125.79 – -126.14 (m, 2F). ^{13}C NMR (150 MHz, $CDCl_3$) δ 197.52, 173.34, 144.95, 133.22, 129.62, 128.66, 64.45, 38.17, 34.00, 31.37, 29.51 (t, *J* = 21.5 Hz), 28.69, 24.68, 22.40, 21.67, 13.95. HRMS (ESI+): calcd for $C_{22}H_{26}F_9O_3^+$ (M+H) 509.1733, found 509.1708.

4,4,5,5,6,6,7,7,7-Nonafluoro-2-(4-fluorobenzoyl)heptyl heptanoate (18)

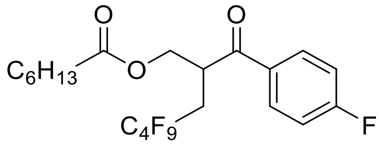

According to the general procedure, allyl heptanoate (38.5 μL, 0.20 mmol, 1.0 equiv.), C_4F_9I (34.4 μL, 0.20 mmol, 1.0 equiv.), 4-fluorobenzoyl chloride (35.5 μL, 0.30 mmol, 1.5 equiv.), $NiCl_2$•glyme (4.4 mg, 0.02 mmol, 0.1 eqiuv), dtbbpy (10.7 mg, 0.04 mmol, 0.2 equiv), and Mn power (33.0 mg, 0.3 mmol, 3.0 equiv) in MeCN (2 mL) were used. After 40 h, the product was isolated by flash chromatography (PE:

EA= 50:1) as a pale-yellow oil liquid (76.8 mg, 75%); ^{1}H NMR (600 MHz, $CDCl_3$) δ 8.06 (dd, *J* = 8.6, 5.4 Hz, 2H), 7.18 (t, *J* = 8.5 Hz, 2H), 4.42-4.36 (m, 1H), 4.27 – 4.21 (m, 1H), 4.23-4.17 (m, 1H), 4.13-4.08 (m, 1H), 3.04 – 2.91 (m, 1H), 2.39 – 2.27 (m, 1H), 2.22 (t, *J* = 7.5 Hz, 2H), 1.54 – 1.46 (m, 2H), 1.27 – 1.21 (m, 6H), 0.86 (t, *J* = 6.9 Hz, 3H). ^{19}F NMR (376 MHz, $CDCl_3$) δ -81.09 (t, *J* = 9.5 Hz, 3F), -103.46 – -103.61 (m, 1F), -112.08 – -112.98 (m, 2F), -124.39 (d, *J* = 9.3 Hz, 2F), -125.89 – -126.21 (m, 2F). ^{13}C NMR (150 MHz, $CDCl_3$) δ 196.61, 173.50, 166.42 (d, *J* = 256.5 Hz), 132.33 (d, *J* = 2.9 Hz), 131.44 (d, *J* = 9.5 Hz), 116.28 (d, *J* = 22.0 Hz), 64.49, 38.46, 34.11, 31.50, 29.75 (t, *J* = 21.4 Hz), 28.82, 24.83, 22.53, 14.06. HRMS (ESI+): calcd for $C_{21}H_{23}F_{10}O_3^+$ (M+) 513.1482, found 513.1487.

Methyl 4-(4,4,5,5,6,6,7,7,7-nonafluoro-2-((heptanoyloxy)methyl)heptanoyl) Benzoate (19)

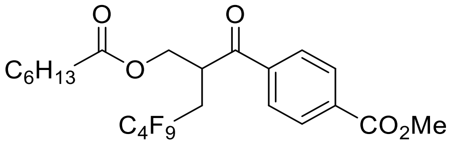

According to the general procedure, allyl heptanoate (38.5 μL, 0.20 mmol, 1.0 equiv.), C_4F_9I (34.4 μL, 0.20 mmol, 1.0 equiv.), methyl 4-(chlorocarbonyl) benzoate (32.1 μL, 0.30 mmol, 1.5 equiv.), $NiCl_2$•glyme (4.4 mg, 0.02 mmol, 0.1 eqiuv), dtbbpy (10.7 mg, 0.04 mmol, 0.2 equiv), and Mn power (33.0 mg, 0.3 mmol, 3.0 equiv) in MeCN (4 mL) were used. After 26 h, the product was isolated by flash chromatography (PE: EA= 40:1) as a pale-yellow solid (54.1 mg, 49%); ^{1}H NMR (600 MHz, $CDCl_3$) δ 8.19 (d, *J* = 8.2 Hz, 2H), 8.08 (d, *J* = 8.2 Hz, 2H), 4.43 – 4.37 (m, 1H), 4.30 – 4.24 (m, 1H), 4.21-4.17 (m, 1H), 3.98 (s, 3H), 3.07 – 2.93 (m, 1H), 2.42 – 2.31 (m, 1H), 2.22 (t, *J* = 7.2 Hz, 2H), 1.53 – 1.48 (m, 2H), 1.27 – 1.22 (m, 6H), 0.88 (t, *J* = 7.0 Hz, 3H). ^{19}F NMR (564 MHz, $CDCl_3$) δ -81.07 (t, *J* = 9.4 Hz, 3F), -112.13 – -112.50 (m, 2F), -124.35 (d, *J* = 6.1 Hz, 2F), -125.97 (s, 2F). ^{13}C NMR (150 MHz, $CDCl_3$) δ 197.84,

173.22, 165.96, 138.97, 134.59, 130.09, 128.40, 64.23, 52.53, 38.69, 33.93, 29.69, 29.60 (t, J = 21.3 Hz), 28.66, 24.65, 22.38, 13.93. HRMS (ESI+): calcd for $C_{23}H_{26}F_9O_5^+$ (M+H) 553.1631, found 553.1650.

4,4,5,5,6,6,7,7,7-Nonafluoro-2-(4-fluoro-3-methylbenzoyl)heptyl heptanoate (20)

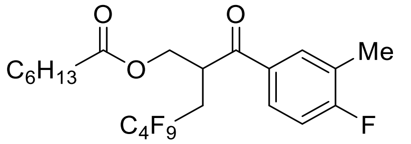

According to the general procedure, allyl heptanoate (38.5 μL, 0.20 mmol, 1.0 equiv.), C_4F_9I (34.4 μL, 0.20 mmol, 1.0 equiv.), 4-fluoro-3-methylbenzoyl chloride (41.3 μL, 0.30 mmol, 1.5 equiv.), $NiCl_2$•glyme (4.4 mg, 0.02 mmol, 0.1 eqiuv), dtbbpy (10.7 mg, 0.04 mmol, 0.2 equiv), and Mn power (33.0 mg, 0.3 mmol, 3.0 equiv) in MeCN (2 mL) were used. After 20 h, the product was isolated by flash chromatography (PE: EA= 70:1) as a colorless oil liquid (77.8 mg, 74%); ^{1}H NMR (600 MHz, $CDCl_3$) δ 7.92 (d, J = 7.2 Hz, 1H), 7.91 – 7.87 (m, 1H), 7.14 (t, J = 8.8 Hz, 1H), 4.43-4.39 (m, 1H), 4.25 – 4.19 (m, 1H), 4.16-4.11 (m, 1H), 3.08 – 2.90 (m, 1H), 2.37 (s, 3H), 2.35 – 2.27 (m, 1H), 2.25 (t, J = 7.5 Hz, 2H), 1.57 – 1.49 (m, 2H), 1.30 – 1.22 (m, 6H), 0.89 (t, J = 7.0 Hz, 3H). ^{19}F NMR (564 MHz, $CDCl_3$) δ -81.07 (s, 3F), -107.53 – -107.78 (m, 1F), -111.87 – -113.23 (m, 2F), -124.40 (s, 2F), -126.01 (s, 2F). ^{13}C NMR (150 MHz, $CDCl_3$) δ 196.65, 173.35, 165.77, 164.08, 132.43 (d, J = 6.8 Hz), 131.82 (d, J = 3.2 Hz), 128.59 (d, J = 9.5 Hz), 125.96 (d, J = 18.0 Hz), 64.38, 38.22, 34.00, 31.38, 29.53 (t, J = 21.2 Hz), 28.69, 24.70, 22.40, 14.61 (d, J = 3.1 Hz), 13.96. HRMS (ESI+): calcd for $C_{22}H_{25}F_{10}O_3^+$ (M+H) 527.1639, found 527.1642.

2-(3-Chlorobenzoyl)-4,4,5,5,6,6,7,7,7-nonafluoroheptyl heptanoate (21)

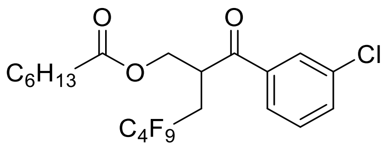

According to the general procedure, allyl heptanoate (38.5 μL, 0.20 mmol, 1.0 equiv.), C_4F_9I (34.4 μL, 0.20 mmol, 1.0 equiv.), 3-chlorobenzoyl chloride (38.4 μL, 0.30 mmol, 1.5 equiv.), $NiCl_2$•glyme (4.4 mg, 0.02 mmol, 0.1 eqiuv), dtbbpy (10.7 mg, 0.04 mmol, 0.2 equiv), and Mn power (33.0 mg, 0.3 mmol, 3.0 equiv) in MeCN (4 mL) were used. After 40 h, the product was isolated by flash chromatography (PE: EA= 60:1) as a pale-yellow oil liquid (66.5 mg, 63%); ^{1}H NMR (600 MHz, $CDCl_3$) δ 8.00 (s, 1H), 7.92 (d, J = 7.8 Hz, 1H), 7.62 (d, J = 8.1 Hz, 1H), 7.49 (t, J = 7.9 Hz, 1H), 4.42 – 4.36 (m, 1H), 4.23 – 4.19 (m, 1H), 4.19 – 4.12 (m, 1H), 3.07 – 2.92 (m, 1H), 2.41 – 2.30 (m, 1H), 2.24 (t, J = 7.2 Hz, 2H), 1.57 – 1.49 (m, 2H), 1.29 – 1.22 (m, 6H), 0.89 (t, J = 7.0 Hz, 3H). ^{19}F NMR (376 MHz, $CDCl_3$) δ -81.11 (t, J = 9.4 Hz, 3F), -112.42 (dt, J = 26.1, 11.9 Hz, 2F), -124.40 (d, J = 9.5 Hz, 2F), -125.87 – -126.15 (m, 2F). ^{13}C NMR (150 MHz, $CDCl_3$) δ 196.96, 173.24, 137.31, 135.39, 133.83, 130.24, 128.58, 126.56, 64.24, 38.55, 33.96, 31.35, 29.58 (t, J = 21.3 Hz), 28.68, 24.67, 22.39, 13.94. HRMS (ESI+): calcd for $C_{21}H_{23}ClF_9O_3^+$ (M+H) 529.1187, found 529.1191.

4,4,5,5,6,6,7,7,7-Nonafluoro-2-(3-fluoro-4-methylbenzoyl)heptyl heptanoate (22)

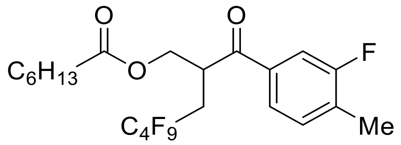

According to the general procedure, allyl heptanoate (38.5 μL, 0.20 mmol, 1.0 equiv.), C_4F_9I (34.4 μL, 0.20 mmol, 1.0 equiv.), 3-fluoro-4-methylbenzoyl chloride (42.7 μL, 0.30 mmol, 1.5 equiv.), $NiCl_2$•glyme (4.4 mg, 0.02 mmol, 0.1 eqiuv), dtbbpy (10.7 mg, 0.04 mmol, 0.2 equiv), and Mn power (33.0 mg, 0.3 mmol, 3.0 equiv) in MeCN (2 mL) were used. After 27 h, the product was isolated by flash chromatography (PE: EA= 60:1) as a colorless oil liquid (87.9 mg, 83%); ^{1}H NMR (600 MHz, $CDCl_3$) δ 7.73 (d, J = 7.9 Hz, 1H), 7.67 (d, J = 10.1 Hz, 1H), 7.35 (t, J = 7.6 Hz, 1H), 4.41-4.37 (m, 1H), 4.22 – 4.18 (m, 1H), 4.18-4.14 (m, 1H), 3.07-2.89 (m, 1H), 2.38 (s, 3H), 2.35

– 2.27 (m, 1H), 2.25 (t, *J* = 7.5 Hz, 2H), 1.57 – 1.50 (m, 2H), 1.30 – 1.22 (m, 6H), 0.89 (t, *J* = 6.9 Hz, 3H). ^{19}F NMR (564 MHz, $CDCl_3$) δ -81.04 (t, *J* = 9.4 Hz, 3F), -111.77 – -113.31 (m, 2F), -115.48 (t, *J* = 8.6 Hz, 1F), -124.34 (d, *J* = 6.7 Hz, 2F), -125.94 (t, *J* = 15.8 Hz, 2F). ^{13}C NMR (150 MHz, $CDCl_3$) δ 196.69 (d, *J* = 1.8 Hz), 173.31, 161.40 (d, *J* = 247.3 Hz), 135.39 (d, *J* = 6.3 Hz), 132.07, 131.92 (d, *J* = 5.0 Hz), 124.11 (d, *J* = 3.2 Hz), 114.89 (d, *J* = 23.5 Hz), 64.34, 38.31, 33.97, 31.37, 29.53 (t, *J* = 21.3 Hz), 28.69, 24.68, 22.40, 14.90 (d, *J* = 3.3 Hz), 13.95. HRMS (ESI+): calcd for $C_{22}H_{25}F_{10}O_3^+$ (M+H) 527.1639, found 527.1641.

2-(3,5-Dimethylbenzoyl)-4,4,5,5,6,6,7,7,7-nonafluoroheptyl heptanoate (23)

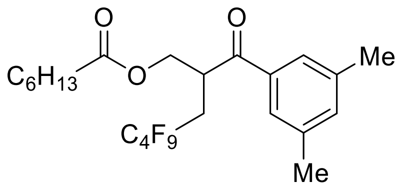

According to the general procedure, allyl heptanoate (38.5 μL, 0.20 mmol, 1.0 equiv.), C_4F_9I (34.4 μL, 0.20 mmol, 1.0 equiv.), 3,5-dimethylbenzoyl chloride (47.9 μL, 0.30 mmol, 1.5 equiv.), $NiCl_2$•glyme (4.4 mg, 0.02 mmol, 0.1 eqiuv), dtbbpy (10.7 mg, 0.04 mmol, 0.2 equiv), and Mn power (33.0 mg, 0.3 mmol, 3.0 equiv) in MeCN (2 mL) were used. After 20 h, the product was isolated by flash chromatography (PE: EA= 60:1) as a colorless oil (77.3 mg, 74%); ^{1}H NMR (600 MHz, $CDCl_3$) δ 7.61 (s, 2H), 7.26 (s, 1H), 4.41-4.37 (m, 1H), 4.26 – 4.20 (m, 1H), 4.18-4.14 (m, 1H), 3.05 – 2.92 (m, 1H), 2.39 (s, 6H), 2.36 – 2.27 (m, 1H), 2.22 (t, *J* = 7.5 Hz, 2H), 1.55 – 1.48 (m, 2H), 1.28 – 1.19 (m, 6H), 0.87 (t, *J* = 7.0 Hz, 3H). ^{19}F NMR (564 MHz, $CDCl_3$) δ -81.09 (t, *J* = 9.6 Hz, 3F), -112.48 – -112.65 (m, 2F), -124.43 (s, 2F), -125.94 – -126.16 (m, 2F). ^{13}C NMR (150 MHz, $CDCl_3$) δ 198.25, 173.34, 138.62, 135.74, 135.63, 126.29, 64.43, 38.36, 34.03, 31.38, 29.39 (t, *J* = 21.4 Hz), 28.71, 24.70, 22.41, 21.24, 13.97. HRMS (ESI+): calcd for $C_{23}H_{28}F_9O_3^+$ (M+H) 523.1889, found 523.1891.

4,4,5,5,6,6,7,7,7-Nonafluoro-2-(4-methoxybenzoyl)heptyl heptanoate (24)

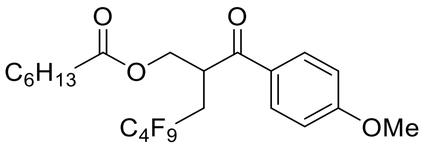

According to the general procedure, allyl heptanoate (38.5 μL, 0.20 mmol, 1.0 equiv.), C_4F_9I (34.4 μL, 0.20 mmol, 1.0 equiv.), 4-methoxybenzoyl chloride (40.6 μL, 0.30 mmol, 1.5 equiv.), $NiCl_2$•glyme (4.4 mg, 0.02 mmol, 0.1 eqiuv), dtbbpy (10.7 mg, 0.04 mmol, 0.2 equiv), and Mn power (33.0 mg, 0.3 mmol, 3.0 equiv) in MeCN (4 mL) were used. After 20 h, the product was isolated by flash chromatography (PE: EA= 60:1) as a pale-yellow oil liquid (64.1 mg, 61%); ^1H NMR (600 MHz, $CDCl_3$) δ 8.04 (d, J = 8.4 Hz, 2H), 7.00 (d, J = 8.4 Hz, 2H), 4.45-4.40 (m, 1H), 4.28 – 4.20 (m, 1H), 4.15-4.10 (m, 1H), 3.91 (s, 3H), 3.07 – 2.93 (m, 1H), 2.40 – 2.29 (m, 1H), 2.25 (t, J = 7.5 Hz, 2H), 1.57 – 1.50 (m, 2H), 1.30 – 1.22 (m, 6H), 0.89 (t, J = 6.9 Hz, 3H). ^{19}F NMR (564 MHz, $CDCl_3$) δ -81.04 (t, J = 9.4 Hz, 3F), -112.43 – -112.61 (m, 2F), -124.34 (s, 2F), -125.89 – -126.06 (m, 2F). ^{13}C NMR (150 MHz, $CDCl_3$) δ 196.25, 173.42, 164.22, 130.95, 128.66, 114.12, 64.56, 55.52, 37.93, 34.02, 31.38, 29.52 (t, J = 21.2 Hz), 28.69, 24.70, 22.40, 13.95. HRMS (ESI+): calcd for $C_{22}H_{26}F_9O_4^+$ (M+H) 525.1682, found 525.1684.

4,4,5,5,6,6,7,7,7-Nonafluoro-2-(2-methylbenzoyl)heptyl heptanoate (25)

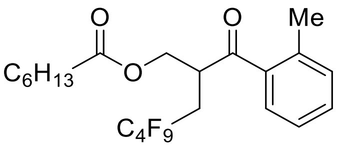

According to the general procedure, allyl heptanoate (38.5 μL, 0.20 mmol, 1.0 equiv.), C_4F_9I (34.4 μL, 0.20 mmol, 1.0 equiv.), 2-methylbenzoyl chloride (39.1 μL, 0.30 mmol, 1.5 equiv.), $NiCl_2$•glyme (4.4 mg, 0.02 mmol, 0.1 eqiuv), dtbbpy (10.7 mg, 0.04 mmol, 0.2 equiv), and Mn power (33.0 mg, 0.3 mmol, 3.0 equiv) in MeCN (2

mL) were used. After 20 h, the product was isolated by flash chromatography (PE: EA= 60:1) as a colorless oil liquid (55.3 mg, 54%); ^{1}H NMR (600 MHz, $CDCl_3$) δ 7.73 (d, *J* = 7.7 Hz, 1H), 7.44 (t, *J* = 7.4 Hz, 1H), 7.33 (t, *J* = 7.6 Hz, 1H), 7.31 (d, *J* = 7.7 Hz, 1H), 4.40-4.35 (m, 1H), 4.2-4.15 (m, 1H), 4.13 – 4.07 (m, 1H), 3.09 – 2.95 (m, 1H), 2.49 (s, 3H), 2.35 – 2.28 (m, 1H), 2.22 (t, *J* = 7.5 Hz, 2H), 1.56 – 1.50 (m, 2H), 1.31 – 1.24 (m, 6H), 0.89 (t, *J* = 7.0 Hz, 3H). ^{19}F NMR (564 MHz, $CDCl_3$) δ -81.08 (t, *J* = 9.5 Hz, 3F), -111.86 – -113.40 (m, 2F), -124.41 (s, 2F), -125.95 (t, *J* = 9.6 Hz, 2F). ^{13}C NMR (150 MHz, $CDCl_3$) δ 201.06, 173.21, 139.07, 136.51, 132.17, 132.03, 128.36, 125.92, 64.34, 41.15, 33.91, 31.36, 29.11 (t, *J* = 21.3 Hz), 28.69, 24.65, 22.40, 20.96, 13.93. HRMS (ESI+): calcd for $C_{22}H_{26}F_9O_3^+$ (M+H) 509.1733, found 509.1707.

4,4,5,5,6,6,7,7,7-Nonafluoro-2-(4-(trifluoromethyl)benzoyl)heptyl heptanoate (26)

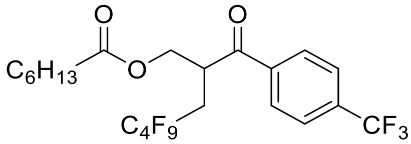

According to the general procedure, allyl heptanoate (38.5 μL, 0.20 mmol, 1.0 equiv.), C_4F_9I (34.4 μL, 0.20 mmol, 1.0 equiv.), 4-(trifluoromethyl)benzoyl chloride (44.6 μL, 0.30 mmol, 1.5 equiv.), $NiCl_2$•glyme (8.8 mg, 0.04 mmol, 0.2 eqiuv), dtbbpy (21.5 mg, 0.08 mmol, 0.4 equiv), and Mn power (33.0 mg, 0.3 mmol, 3.0 equiv) in MeCN (2 mL) were used. After 20 h, the product was isolated by flash chromatography (PE: EA= 80:1) as a colorless oil liquid (88.8 mg, 79%); ^{1}H NMR (600 MHz, $CDCl_3$) δ 8.15 (d, *J* = 8.2 Hz, 2H), 7.81 (d, *J* = 8.2 Hz, 2H), 4.44-4.39 (m, 1H), 4.30 – 4.24 (m, 1H), 4.20-4.15 (m, 1H), 3.10 – 2.94 (m, 1H), 2.44 – 2.32 (m, 1H), 2.23 (t, *J* = 7.5 Hz, 2H), 1.55 – 1.48 (m, 2H), 1.30 – 1.22 (m, 6H), 0.89 (t, *J* = 7.1 Hz, 3H). ^{19}F NMR (564 MHz, $CDCl_3$) δ -63.33 (s, 3F), -81.07 (t, *J* = 9.6 Hz, 3F), -111.65 – -113.01 (m, 2F), -124.34 (d, *J* = 7.0 Hz, 2F), -125.96 (d, *J* = 10.9 Hz, 2F). ^{13}C NMR (150 MHz,

$CDCl_3$) δ 197.38, 173.26, 138.46, 135.15 (q, *J* = 32.9 Hz), 128.85, 126.01 (q, *J* = 3.6 Hz), 123.42 (q, *J* = 272.8 Hz), 64.16, 38.71, 33.93, 31.33, 29.65 (t, *J* = 21.2 Hz), 28.66, 24.66, 22.38, 13.91. HRMS (ESI+): calcd for $C_{22}H_{23}F_{12}O_3^+$ (M+H) 563.1450, found 563.1446.

4,4,5,5,6,6,7,7,7-Nonafluoro-2-(thiophene-2-carbonyl)heptyl heptanoate (27)

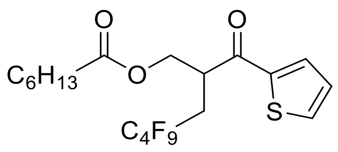

According to the general procedure, allyl heptanoate (38.5 μL, 0.20 mmol, 1.0 equiv.), C_4F_9I (34.4 μL, 0.20 mmol, 1.0 equiv.), thiophene-2-carbonyl chloride (32.1 μL, 0.30 mmol, 1.5 equiv.), $NiCl_2$•glyme (4.4 mg, 0.02 mmol, 0.1 eqiuv), dtbbpy (10.7 mg, 0.04 mmol, 0.2 equiv), and Mn power (33.0 mg, 0.3 mmol, 3.0 equiv) in MeCN (2 mL) were used. After 70 h, the product was isolated by flash chromatography (PE: EA= 60:1) as a yellow oil (72.1 mg, 72%); ^1H NMR (600 MHz, $CDCl_3$) δ 7.90 (d, *J* = 3.5 Hz, 1H), 7.76 (d, *J* = 4.6 Hz, 1H), 7.21 (t, *J* = 4.2 Hz, 1H), 4.47-4.42 (m, 1H), 4.22-4.17 (m, 1H), 4.11 – 4.04 (m, 1H), 3.04 – 2.91 (m, 1H), 2.40 – 2.31 (m, 1H), 2.26 (t, *J* = 7.5 Hz, 2H), 1.58 – 1.51 (m, 2H), 1.30 – 1.25 (m, 6H), 0.89 (t, *J* = 7.0 Hz, 3H). ^{19}F NMR (564 MHz, $CDCl_3$) δ -81.06 (t, *J* = 9.6 Hz, 3F), -111.74 – -113.48 (m, 2F), -124.33 (d, *J* = 5.5 Hz, 2F), -125.89 – -126.04 (m, 2F). ^{13}C NMR (150 MHz, $CDCl_3$) δ 190.36, 173.34, 142.99, 135.44, 133.08, 128.52, 64.52, 40.05, 34.00, 31.37, 29.48 (t, *J* = 21.4 Hz), 28.68, 24.67, 22.40, 13.95. HRMS (ESI+): calcd for $C_{19}H_{22}F_9O_3S^+$ (M+H) 501.1140, found 501.1141.

4,4,5,5,6,6,7,7,7-Nonafluoro-2-(furan-2-carbonyl)heptyl heptanoate (28)

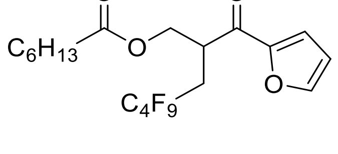

According to the general procedure, allyl heptanoate (38.5 μL, 0.20 mmol, 1.0 equiv.), C_4F_9I (34.4 μL, 0.20 mmol, 1.0 equiv.), furan-2-carbonyl chloride (29.6 μL, 0.30 mmol, 1.5 equiv.), $NiCl_2$•glyme (8.8 mg, 0.04 mmol, 0.2 eqiuv), dtbbpy (21.5 mg, 0.08 mmol, 0.4 equiv), and Mn power (33.0 mg, 0.3 mmol, 3.0 equiv) in MeCN (2 mL) were used. After 24 h, the product was isolated by flash chromatography (PE: EA= 40:1) as a pale-yellow oil (56.6 mg, 58%); ^{1}H NMR (600 MHz, $CDCl_3$) δ 7.67 (s, 1H), 7.37 (d, J = 3.5 Hz, 1H), 6.62 (t, J = 3.3 Hz, 1H), 4.40-4.36 (m, 1H), 4.31-4.26 (m, 1H), 4.08– 4.00 (m, 1H), 3.01 – 2.87 (m, 1H), 2.38 – 2.28 (m, 1H), 2.26 (t, J = 7.5 Hz, 2H), 1.59 – 1.52 (m, 2H), 1.32 – 1.23 (m, 6H), 0.89 (t, J = 6.9 Hz, 3H). ^{19}F NMR (564 MHz, $CDCl_3$) δ -81.09 (t, J = 9.6 Hz, 3F), -112.56 – -113.01 (m, 2F), -124.37 (s, 2F), -125.88 – -126.11 (m, 2F). ^{13}C NMR (150 MHz, $CDCl_3$) δ 186.09, 173.31, 151.66, 147.47, 118.97, 112.82, 64.07, 39.16, 33.99, 31.38, 28.87 (t, J = 21.6 Hz), 28.70, 24.68, 22.42, 13.97. HRMS (ESI+): calcd for $C_{19}H_{22}F_9O_4^+$ (M+H) 485.1369, found 485.1374.

4,4,5,5,6,6,7,7,7-Nonafluoro-2-(3-phenylpropanoyl)heptyl heptanoate (29)

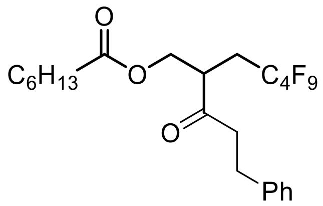

According to the general procedure, allyl heptanoate (38.5 μL, 0.20 mmol, 1.0 equiv.), C_4F_9I (34.4 μL, 0.20 mmol, 1.0 equiv.), 3-phenylpropanoyl chloride (44.6 μL, 0.30 mmol, 1.5 equiv.), $NiCl_2$•glyme (4.4 mg, 0.02 mmol, 0.1 equiv.), dtbbpy (10.7 mg, 0.04 mmol, 0.2 equiv.), and Mn power (33.0 mg, 0.3 mmol, 3.0 equiv.) in MeCN (2 mL) were used. After 20 h, the product was isolated by flash chromatography (PE: EA= 60:1) as a colorless oil liquid (43.8 mg, 42%); ^{1}H NMR (600 MHz, $CDCl_3$) δ 7.30 (t, J = 7.5 Hz, 2H), 7.24 – 7.19 (m, 3H), 4.25 – 4.18 (m, 2H), 3.28 – 3.22 (m, 1H), 3.00 – 2.86 (m, 4H), 2.84 – 2.72 (m, 1H), 2.27 (t, J = 7.6 Hz, 2H), 2.22 – 2.11 (m, 1H),

1.61 – 1.57 (m, 2H), 1.32 – 1.28 (m, 6H), 0.91 (t, J = 7.0 Hz, 3H). ^{19}F NMR (564 MHz, $CDCl_3$) δ -81.02 (t, J = 9.5 Hz, 3F), -112.35 – -112.98 (m, 2F), -124.38 (s, 2F), -125.85 – -126.10 (m, 2F). ^{13}C NMR (150 MHz, $CDCl_3$) δ 207.06, 173.13, 140.55, 128.53, 128.28, 126.26, 63.60, 44.43, 43.45, 33.93, 31.39, 29.33, 28.90 (t, J = 21.8 Hz), 28.72, 24.74, 22.43, 13.94. HRMS (ESI+): calcd for $C_{23}H_{28}F_9O_3^+$ (M+H) 523.1889, found 523.1993.

2-(4-(*tert*-Butyl)benzoyl)-4,4,5,5,6,6,6-heptafluorohexyl heptanoate (30)

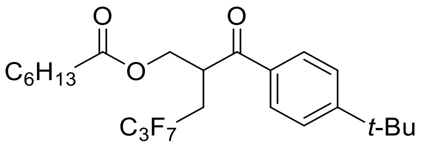

According to the general procedure, allyl heptanoate (38.5 μL, 0.20 mmol, 1.0 equiv.), C_3F_7I (28.9 μL, 0.20 mmol, 1.0 equiv.), 4-(*tert*-butyl)benzoyl chloride (58.6 μL, 0.30 mmol, 1.5 equiv.), $NiCl_2$•glyme (4.4 mg, 0.02 mmol, 0.1 eqiuv), dtbbpy (10.7 mg, 0.04 mmol, 0.2 equiv), and Mn power (33.0 mg, 0.3 mmol, 3.0 equiv) in MeCN (2 mL) were used. After 20 h, the product was isolated by flash chromatography (PE: EA= 90:1) as a yellow oil (92.1 mg, 91%); ^{1}H NMR (600 MHz, $CDCl_3$) δ 7.99 (d, J = 8.1 Hz, 2H), 7.54 (d, J = 8.1 Hz, 2H), 4.47-4.41 (m, 1H), 4.31 – 4.23 (m, 1H), 4.16-4.12 (m, 1H), 3.10 – 2.92 (m, 1H), 2.40-2.28 (m, 1H), 2.24 (t, J = 7.2 Hz, 2H), 1.57 – 1.50 (m, 2H), 1.37 (s, 9H), 1.29 – 1.22 (m, 6H), 0.89 (t, J = 6.9 Hz, 3H). ^{19}F NMR (376 MHz, $CDCl_3$) δ -80.49 (t, J = 9.7 Hz, 3F), -110.93 – -115.79 (m, 2F), -127.65 – -127.76 (m, 2F). ^{13}C NMR (150 MHz, $CDCl_3$) δ 197.43, 173.39, 157.83, 133.04, 128.52, 125.90, 64.42, 38.21, 35.21, 34.00, 31.37, 31.01, 29.27 (t, J = 21.3 Hz), 28.69, 24.68, 22.41, 13.96. HRMS (ESI+): calcd for $C_{24}H_{32}F_7O_3^+$ (M+H) 501.2234, found 501.2236.

2-(4-(*tert*-Butyl)benzoyl)-4,4,5,5,6,6,7,7,8,8,9,9,9-tridecafluorononyl heptanoate (31)

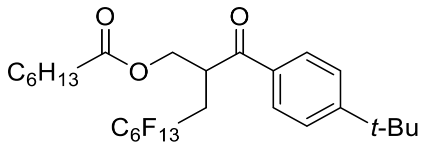

According to the general procedure, allyl heptanoate (38.5 μL, 0.20 mmol, 1.0 equiv.), $C_6F_{13}I$ (43.2 μL, 0.20 mmol, 1.0 equiv.), 4-(*tert*-butyl)benzoyl chloride (58.6 μL, 0.30 mmol, 1.5 equiv.), $NiCl_2$•glyme (4.4 mg, 0.02 mmol, 0.1 eqiuv), dtbbpy (10.7 mg, 0.04 mmol, 0.2 equiv), and Mn power (33.0 mg, 0.3 mmol, 3.0 equiv) in MeCN (2 mL) were used. After 24 h, the product was isolated by flash chromatography (PE: EA= 80:1) as a yellow oil (111.1 mg, 85%); ^{1}H NMR (600 MHz, $CDCl_3$) δ 7.99 (d, *J* = 8.6 Hz, 2H), 7.55 (d, *J* = 8.6 Hz, 2H), 4.47-4.42 (m, 1H), 4.30 – 4.24 (m, 1H), 4.15-4.11 (m, 1H), 3.10 – 2.95 (m, 1H), 2.41 – 2.29 (m, 1H), 2.25 (t, *J* = 7.5 Hz, 2H), 1.57 – 1.51 (m, 2H), 1.37 (s, 9H), 1.30 – 1.23 (m, 6H), 0.89 (t, *J* = 7.0 Hz, 3H). ^{19}F NMR (564 MHz, $CDCl_3$) δ -80.79 (t, *J* = 10.0 Hz, 3F), -112.33 (dd, *J* = 27.9, 14.3 Hz, 2F), -121.86 (s, 2F), -122.90 (s, 2F), -123.44 (s, 2F), -126.18 (d, *J* = 14.6 Hz, 2F). ^{13}C NMR (150 MHz, $CDCl_3$) δ 197.38, 173.40, 157.82, 133.00, 128.54, 125.91, 64.40, 38.24, 35.20, 34.00, 31.38, 30.99, 29.48 (t, *J* = 21.2 Hz), 28.71, 24.69, 22.42, 13.95. HRMS (ESI+): calcd for $C_{27}H_{32}F_{13}O_3^+$ (M+H) 651.2138, found 651.2138.

2-(4-(*tert*-Butyl)benzoyl)-4,4,5,5,6,6,7,7,8,8,9,9,10,10,11,11,11-heptadecafluoroundecyl heptanoate (32)

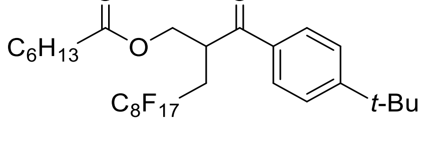

According to the general procedure, allyl heptanoate (38.5 μL, 0.20 mmol, 1.0 equiv.), $C_8F_{17}I$ (52.8 μL, 0.20 mmol, 1.0 equiv.), 4-(*tert*-butyl)benzoyl chloride (58.6 μL, 0.30 mmol, 1.5 equiv.), $NiCl_2$•glyme (4.4 mg, 0.02 mmol, 0.1 eqiuv), dtbbpy (10.7 mg,

0.04 mmol, 0.2 equiv), and Mn power (33.0 mg, 0.3 mmol, 3.0 equiv) in MeCN (2 mL) were used. After 24 h, the product was isolated by flash chromatography (PE: EA= 80:1) as a white solid (117.1 mg, 78%); ^{1}H NMR (600 MHz, $CDCl_3$) δ 7.99 (d, *J* = 8.5 Hz, 2H), 7.55 (d, *J* = 8.5 Hz, 2H), 4.46-4.42 (m, 1H), 4.31 – 4.24 (m, 1H), 4.15-4.11 (m, 1H), 3.10 – 2.95 (m, 1H), 2.41 – 2.28 (m, 1H), 2.25 (t, *J* = 7.5 Hz, 2H), 1.57 – 1.50 (m, 2H), 1.37 (s, 9H), 1.30 – 1.23 (m, 6H), 0.89 (t, *J* = 7.0 Hz, 3H). ^{19}F NMR (564 MHz, $CDCl_3$) δ -80.75 (t, *J* = 9.8 Hz, 3F), -112.15 – -112.45 (m, 2F), -121.63 (s, 2F), -121.95 (s, 4F), -122.75 (s, 2F), -123.38 (s, 2F), -126.15 (d, *J* = 12.7 Hz, 2F); ^{13}C NMR (150 MHz, $CDCl_3$) δ 197.37, 173.39, 157.81, 133.01, 128.53, 125.90, 64.40, 38.24, 35.19, 33.99, 31.38, 30.96, 29.47 (t, *J* = 21.3 Hz), 28.70, 24.69, 22.40, 13.91. HRMS (ESI+): calcd for $C_{29}H_{32}F_{17}O_3^+$ (M+H) 751.2075, found 751.2071.

2-(4-(*tert*-Butyl)benzoyl)-4,4,5,5,6,6,7,7,8,8,9,9,10,10,11,11,12,12,13,13,13-henicosafluorotridecyl heptanoate (33)

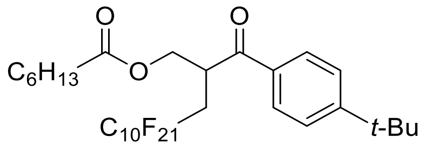

According to the general procedure, allyl heptanoate (38.5 μL, 0.20 mmol, 1.0 equiv.), $C_{10}F_{21}I$ (129.2 mg, 0.20 mmol, 1.0 equiv.), 4-(tert-butyl)benzoyl chloride (58.6 μL, 0.30 mmol, 1.5 equiv.), $NiCl_2$•glyme (4.4 mg, 0.02 mmol, 0.1 eqiuv), dtbbpy (10.7 mg, 0.04 mmol, 0.2 equiv), and Mn power (33.0 mg, 0.3 mmol, 3.0 equiv) in MeCN/DME(4:1) (2 mL) were used. After 48 h, the product was isolated by flash chromatography (PE: EA= 80:1) as a White solid (123.0 mg, 71%); ^{1}H NMR (600 MHz, $CDCl_3$) δ 7.99 (d, *J* = 8.4 Hz, 2H), 7.54 (d, *J* = 8.4 Hz, 2H), 4.47-4.42 (m, 1H), 4.31 – 4.23 (m, 1H), 4.16-4.11 (m, 1H), 3.14 – 2.93 (m, 1H), 2.41 – 2.30 (m, 1H), 2.24 (t, *J* = 7.5 Hz, 2H), 1.57 – 1.49 (m, 2H), 1.37 (s, 9H), 1.30 – 1.23 (m, 6H), 0.88 (t,

J = 7.0 Hz, 3H). ^{19}F NMR (564 MHz, CDCl$_3$) δ -80.95 (t, J = 9.6 Hz, 3F), -111.71 – -113.21 (m, 2F), -121.71 (s, 2F), -121.90 (s, 5F), -122.04 (s, 3F), -122.87 (s, 2F), -123.48 (s, 2F), -126.30 (s, 2F). ^{13}C NMR (150 MHz, CDCl$_3$) δ 197.37, 173.38, 157.80, 133.02, 128.52, 125.89, 64.40, 38.25, 35.18, 33.99, 31.37, 30.96, 29.49 (t, J = 21.6 Hz), 28.70, 24.68, 22.39, 13.89. HRMS (ESI$^+$): calcd for $C_{31}H_{32}F_{21}O_3^+$ (M+H) 851.2011, found 851.2011.

2-(4-(*tert*-Butyl)benzoyl)-5-ethoxy-4,4-difluoro-5-oxopentyl heptanoate (34)

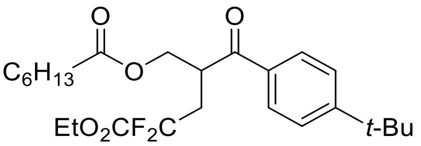

According to the general procedure, allyl heptanoate (38.5 μL, 0.20 mmol, 1.0 equiv.), ICF_2CO_2Et (29.4 μL, 0.20 mmol, 1.0 equiv.), 4-(*tert*-butyl)benzoyl chloride (58.6 μL, 0.30 mmol, 1.5 equiv.), $NiCl_2$•glyme (4.4 mg, 0.02 mmol, 0.1 eqiuv), dtbbpy (10.7 mg, 0.04 mmol, 0.2 equiv), and Mn power (33.0 mg, 0.3 mmol, 3.0 equiv) in MeCN (2 mL) were used. After 20 h, the product was isolated by flash chromatography (PE: EA= 40:1) as a yellow oil (73.3 mg, 81%); ^{1}H NMR (600 MHz, CDCl$_3$) δ 7.96 (d, J = 8.1 Hz, 2H), 7.52 (d, J = 8.1 Hz, 2H), 4.42-4.37 (m, 1H), 4.31 – 4.21 (m, 2H), 4.21 – 4.17 (m, 1H), 4.16-4.12 (m, 1H), 2.96 – 2.83 (m, 1H), 2.39 – 2.28 (m, 1H), 2.22 (t, J = 7.2 Hz, 2H), 1.55 – 1.49 (m, 2H), 1.36 (s, 9H), 1.29 (t, J = 7.1 Hz, 3H), 1.26 – 1.23 (m, 6H), 0.88 (t, J = 7.0 Hz, 3H). ^{19}F NMR (564 MHz, CDCl$_3$) δ -104.39 (ddq, J = 260.5, 20.9, 13.0 Hz, 2F). ^{13}C NMR (150 MHz, CDCl$_3$) δ 198.12, 173.40, 163.68 (t, J = 32.4 Hz), 157.53, 133.40, 128.51, 125.79, 115.21 (t, J = 251.1 Hz), 64.66, 63.09, 39.24, 35.18, 34.02, 33.24 (t, J = 23.5 Hz), 31.38, 31.04, 28.71, 24.67, 22.43, 14.00, 13.79. HRMS (ESI+): calcd for $C_{25}H_{37}F_2O_5^+$ (M+H) 455.2604, found 455.2607.

2-(4-(*tert*-Butyl)benzoyl)-4,5,5,5-tetrafluoro-4-(trifluoromethyl)pentyl heptanoate (35)

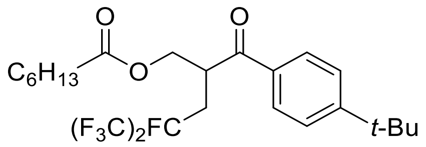

According to the general procedure, allyl heptanoate (38.5 μL, 0.20 mmol, 1.0 equiv.), $ICF(CF_3)_2$ (28.5 μL, 0.20 mmol, 1.0 equiv.), 4-(tert-butyl)benzoyl chloride (58.6 μL, 0.30 mmol, 1.5 equiv.), $NiCl_2$•glyme (4.4 mg, 0.02 mmol, 0.1 eqiuv), dtbbpy (10.7 mg, 0.04 mmol, 0.2 equiv), and Mn power (33.0 mg, 0.3 mmol, 3.0 equiv) in MeCN (2 mL) were used. After 24 h, the product was isolated by flash chromatography (PE: EA= 120:1) as a yellow oil (90.9 mg, 91%); ^{1}H NMR (600 MHz, $CDCl_3$) δ 8.00 (d, *J* = 8.1 Hz, 2H), 7.54 (d, *J* = 7.9 Hz, 2H), 4.48-4.42 (m, 1H), 4.32 – 4.24 (m, 1H), 4.01-3.95 (m, 1H), 3.26 – 3.14 (m, 1H), 2.38 – 2.29 (m, 1H), 2.26 (t, *J* = 7.5 Hz, 2H), 1.59 – 1.53 (m, 2H), 1.37 (s, 9H), 1.30 – 1.24 (m, 6H), 0.90 (t, *J* = 6.8 Hz, 3H). ^{19}F NMR (564 MHz, $CDCl_3$) δ -76.95 (dt, *J* = 16.1, 7.6 Hz, 6F), -183.81 – -183.97 (m, 1F). ^{13}C NMR (150 MHz, $CDCl_3$) δ 196.76, 173.40, 157.80, 132.83, 128.49, 125.91, 64.60, 39.57, 35.19, 33.96, 31.37, 30.99, 28.71, 26.29 (d, *J* = 19.3 Hz), 24.64, 22.42, 13.95. HRMS (ESI$^+$): calcd for $C_{24}H_{32}F_7O_3^+$ (M+H) 501.2234, found 501.2241.

3-(4-(*tert*-Butyl)benzoyl)-5,5,6,6,7,7,8,8,8-nonafluorooctyl benzoate (36)

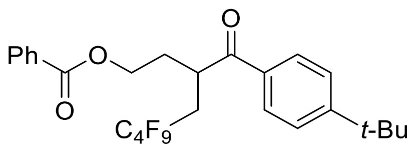

According to the general procedure, but-3-en-1-yl benzoate (35.6 μL, 0.20 mmol, 1.0 equiv.), C_4F_9I (34.4 μL, 0.20 mmol, 1.0 equiv.), 4-(*tert*-butyl)benzoyl chloride (58.6 μL, 0.30 mmol, 1.5 equiv.), $NiCl_2$•glyme (4.4 mg, 0.02 mmol, 0.1 eqiuv), dtbbpy (10.7 mg, 0.04 mmol, 0.2 equiv), and Mn power (33.0 mg, 0.3 mmol, 3.0 equiv) in MeCN (2 mL) were used. After 27 h, the product was isolated by flash

chromatography (PE: EA= 60:1) as a colorless oil (97.9 mg, 88%); ^{1}H NMR (600 MHz, $CDCl_3$) δ 7.98 – 7.92 (m, 4H), 7.59 (t, *J* = 7.4 Hz, 1H), 7.47 – 7.42 (m, 4H), 4.43 – 4.37 (m, 1H), 4.35 – 4.30 (m, 1H), 4.18 – 4.13 (m, 1H), 3.13 – 2.97 (m, 1H), 2.39 – 2.28 (m, 2H), 2.14 – 2.06 (m, 1H), 1.33 (s, 9H). ^{19}F NMR (564 MHz, $CDCl_3$) δ -81.07 (t, *J* = 9.6 Hz, 3F), -111.80 – -113.45 (m, 2F), -124.38 (s, 2F), -125.89 – -126.01 (m, 2F). ^{13}C NMR (150 MHz, $CDCl_3$) δ 199.83, 166.12, 157.59, 133.09, 132.97, 129.74, 129.52, 128.40, 128.35, 125.82, 62.09, 35.24, 35.11, 32.50, 32.39 (t, *J* = 21.1 Hz), 30.97. HRMS (ESI+): calcd for $C_{26}H_{26}F_9O_3^+$ (M+H) 557.1733, found 557.1706.

4-(4-(*tert*-Butyl)benzoyl)-6,6,7,7,8,8,9,9,9-nonafluorononyl benzoate (37)

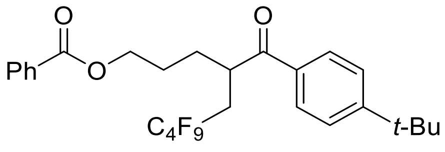

According to the general procedure, pent-4-en-1-yl benzoate (37.7 μL, 0.20 mmol, 1.0 equiv.), C_4F_9I (34.4 μL, 0.20 mmol, 1.0 equiv.), 4-(tert-butyl)benzoyl chloride (58.6 μL, 0.30 mmol, 1.5 equiv.), $NiCl_2$•glyme (4.4 mg, 0.02 mmol, 0.1 eqiuv), dtbbpy (10.7 mg, 0.04 mmol, 0.2 equiv), and Mn power (33.0 mg, 0.3 mmol, 3.0 equiv) in MeCN (2 mL) were used. After 23 h, the product was isolated by flash chromatography (PE: EA= 60:1) as a white solid (65.4 mg, 57%); ^{1}H NMR (600 MHz, $CDCl_3$) δ 8.01 (d, *J* = 8.4 Hz, 2H), 7.95 (d, *J* = 8.6 Hz, 2H), 7.58 (t, *J* = 7.4 Hz, 1H), 7.50 (d, *J* = 8.6 Hz, 2H), 7.44 (t, *J* = 7.8 Hz, 2H), 4.34 – 4.27 (m, 2H), 4.02 – 3.94 (m, 1H), 3.09 – 2.93 (m, 1H), 2.34 – 2.17 (m, 1H), 2.03 – 1.96 (m, 1H), 1.85 – 1.80 (m, 1H), 1.80 – 1.76 (m, 2H), 1.36 (s, 9H). ^{19}F NMR (564 MHz, $CDCl_3$) δ -81.04 (t, *J* = 9.5 Hz, 3F), -111.77 – -113.21 (m, 2F), -124.35 (s, 2F), -125.87 – -126.00 (m, 2F). ^{13}C NMR (150 MHz, $CDCl_3$) δ 200.20, 166.48, 157.53, 133.17, 133.01, 130.05,

129.54, 128.37, 128.36, 125.90, 64.05, 37.46, 35.17, 31.87 (t, *J* = 21.2 Hz), 31.02, 29.94, 25.96. HRMS (ESI$^+$): calcd for $C_{27}H_{28}F_9O_3^+$ (M+H) 571.1889, found 571.1855.

2-(4-(*tert*-Butyl)benzoyl)-4,4,5,5,6,6,7,7,7-nonafluoroheptyl pent-4-en-1-yl carbonate (41)

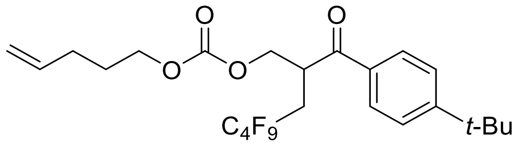

According to the general procedure, **S4** (36.0 μL, 0.20 mmol, 1.0 equiv.), C_4F_9I (34.4 μL, 0.20 mmol, 1.0 equiv.), 4-(*tert*-butyl)benzoyl chloride (58.6 μL, 0.30 mmol, 1.5 equiv.), $NiCl_2$•glyme (4.4 mg, 0.02 mmol, 0.1 eqiuv), dtbbpy (10.7 mg, 0.04 mmol, 0.2 equiv), and Mn power (33.0 mg, 0.3 mmol, 3.0 equiv) in MeCN (2 mL) were used. After 20 h, the product was isolated by flash chromatography (PE: EA= 60:1) as a pale-yellow liquid (47.3 mg, 43%); ^{1}H NMR (600 MHz, $CDCl_3$) δ 7.92 (d, *J* = 8.4 Hz, 2H), 7.51 (d, *J* = 8.4 Hz, 2H), 5.94 – 5.88 (m, 1H), 5.34 (dd, *J* = 17.2, 1.2 Hz, 1H), 5.26 (dd, *J* = 10.4, 0.8 Hz, 1H), 4.60 (d, *J* = 5.8 Hz, 2H), 4.10 (t, *J* = 6.0 Hz, 2H), 3.93 – 3.90 (m, 1H), 3.02 – 2.91 (m, 1H), 2.19 – 2.13 (m, 1H), 1.90 – 1.85 (m, 1H), 1.72 – 1.63 (m, 3H), 1.35 (s, 9H); ^{19}F NMR (564 MHz, $CDCl_3$) δ -81.07 (t, *J* = 9.6 Hz, 3F), -111.78 – -113.31 (m, 2F), -124.40 (s, 2F), -125.92 – -126.04 (m, 2F); ^{13}C NMR (150 MHz, $CDCl_3$) δ 200.22, 157.52, 154.86, 133.23, 131.44, 128.35, 125.89, 118.97, 68.45, 67.24, 37.32, 35.16, 32.05 (t, *J* = 21.1 Hz), 31.01, 29.82, 25.95. HRMS (ESI$^+$): calcd for $C_{24}H_{28}F_9O_4^+$ (M+H) 551.1838, found 551.1833.

2-(4-(*tert*-Butyl)benzoyl)-4,4,5,5,6,6,7,7,7-nonafluoroheptyl cyclohex-2-en-1-yl carbonate (42)

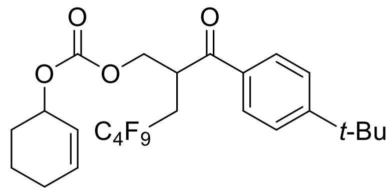

According to the general procedure, **S5** (36.9 μL, 0.20 mmol, 1.0 equiv.), C_4F_9I (34.4 μL, 0.20 mmol, 1.0 equiv.), 4-(*tert*-butyl)benzoyl chloride (58.6 μL, 0.30 mmol, 1.5 equiv.), $NiCl_2$•glyme (4.4 mg, 0.02 mmol, 0.1 eqiuv), dtbbpy (10.7 mg, 0.04 mmol, 0.2 equiv), and Mn power (33.0 mg, 0.3 mmol, 3.0 equiv) in MeCN (2 mL) were used. After 37 h, the product was isolated by flash chromatography (PE: EA= 60:1) as a colorless oil (73.1 mg, 65%); ^{1}H NMR (600 MHz, $CDCl_3$) δ 8.01 (d, *J* = 8.3 Hz, 2H), 7.55 (d, *J* = 8.3 Hz, 2H), 6.03 – 5.97 (m, 1H), 5.77 – 5.71 (m, 1H), 5.17 – 5.07 (m, 1H), 4.52 – 4.46 (m, 1H), 4.37 – 4.29 (m, 1H), 4.22 – 4.07 (m, 1H), 3.12 – 2.96 (m, 1H), 2.51 – 2.35 (m, 1H), 2.13 – 2.07 (m, 1H), 2.03 – 1.97 (m, 1H), 1.91 – 1.85 (m, 1H), 1.83 – 1.77 (m, 1H), 1.76 – 1.70 (m, 1H), 1.67 – 1.59 (m, 1H), 1.37 (s, 9H). ^{19}F NMR (564 MHz, $CDCl_3$) δ -81.07 (t, *J* = 9.4 Hz, 3F), -112.19 – -112.63 (m, 2F), -124.27 (s, 2F), -125.97 (t, *J* = 11.3 Hz, 2F). ^{13}C NMR (150 MHz, $CDCl_3$) δ 196.95 (d, *J* = 5.3 Hz), 157.89 (d, *J* = 1.4 Hz), 154.43, 133.70 (d, *J* = 5.6 Hz), 132.86 (d, *J* = 2.7 Hz), 128.58 (d, *J* = 2.8 Hz), 125.95, 124.48 (d, *J* = 3.6 Hz), 72.56, 67.30 (d, *J* = 3.2 Hz), 38.38, 35.21, 30.99, 29.42 (t, *J* = 21.6 Hz), 28.04 (d, *J* = 4.7 Hz), 24.77 (d, *J* = 2.2 Hz), 18.46 (d, *J* = 6.1 Hz). HRMS (ESI+): calcd for $C_{25}H_{28}F_9O_4^+$ (M+H) 563.1838, found 563.1833.

2-(4-(*tert*-Butyl)benzoyl)-3,3,4,4,5,5,6,6,6-nonafluorohexyl (2-8,8a-dimethyl -1,2,3,4,6,7,8,8a-octahydronaphthalen-2-yl)propyl) carbonate (43)

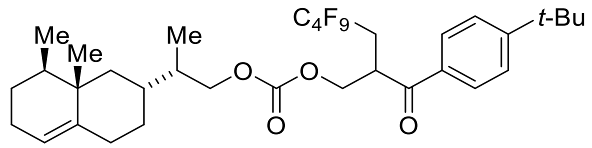

According to the general procedure, **S6** (70.2 μL, 0.20 mmol, 1.0 equiv.), C_4F_9I (34.4 μL, 0.20 mmol, 1.0 equiv.), 4-(*tert*-butyl)benzoyl chloride (58.6 μL, 0.30 mmol, 1.5 equiv.), $NiCl_2$•glyme (4.4 mg, 0.02 mmol, 0.1 eqiuv), dtbbpy (10.7 mg, 0.04 mmol, 0.2 equiv), and Mn power (33.0 mg, 0.3 mmol, 3.0 equiv) in MeCN/DME (4:1) (4 mL) were used. After 48 h, the product was isolated by flash chromatography (hexanes: Et_2O= 60:1) as a colorless oil liquid (64.4 mg, 47%); ^1H NMR (600 MHz, $CDCl_3$) δ 8.01 (d, J = 8.3 Hz, 2H), 7.55 (d, J = 8.4 Hz, 2H), 5.37 – 5.28 (m, 1H), 4.55 – 4.47 (m, 1H), 4.36 – 4.30 (m, 1H), 4.19 – 4.11 (m, 2H), 4.02 – 3.93 (m, 1H), 3.10 – 2.94 (m, 1H), 2.50 – 2.35 (m, 1H), 2.30 – 2.22 (m, 1H), 2.10 – 1.94 (m, 3H), 1.81 – 1.75 (m, 1H), 1.71 – 1.63 (m, 3H), 1.44 – 1.40 (m, 2H), 1.37 (s, 9H), 1.30 – 1.27 (m, 1H), 1.06 – 0.96 (m, 1H), 0.94 – 0.90 (m, 4H), 0.90 – 0.87 (m, 5H), 0.84 – 0.75 (m, 1H). ^{19}F NMR (564 MHz, $CDCl_3$) δ -81.03 (t, J = 9.3 Hz), -112.10 – -112.79 (m), -124.28 (s), -125.84 – -126.06 (m). ^{13}C NMR (150 MHz, $CDCl_3$) δ 196.85, 157.94, 154.97, 143.15, 132.80, 128.58, 125.97, 120.06 (d, J = 4.8 Hz), 72.03 (d, J = 3.7 Hz), 71.85, 67.34, 43.73 (d, J = 3.1 Hz), 41.90 (d, J = 11.1 Hz), 41.05 (d, J = 2.1 Hz), 38.45, 37.38, 35.23, 32.11 (d, J = 111.5 Hz), 31.02, 29.39 (t, J = 21.7 Hz), 27.12 (d, J = 3.3 Hz), 25.85, 18.40, 15.64 (d, J = 4.4 Hz), 13.53 (d, J = 7.8 Hz), 13.25 (d, J = 3.3 Hz). HRMS (ESI+): calcd for $C_{34}H_{44}F_9O_4^+$ (M+H) 687.3090, found 687.3093.

2-(4-(*tert*-Butyl)benzoyl)-4,4,5,5,6,6,7,7,7-nonafluoroheptyl(2-oxo-2H-chromen-6-yl) carbonate (44)

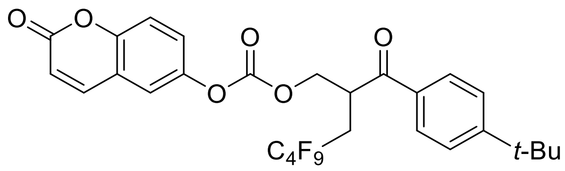

According to the general procedure, **S7** (49.2 mg, 0.20 mmol, 1.0 equiv.), C_4F_9I (34.4 μL, 0.20 mmol, 1.0 equiv.), 4-(*tert*-butyl)benzoyl chloride (58.6 μL, 0.30 mmol, 1.5 equiv.), $NiCl_2$•glyme (4.4 mg, 0.02 mmol, 0.1 equiv.), dtbbpy (10.7 mg, 0.04 mmol,

0.2 equiv), and Mn power (33.0 mg, 0.3 mmol, 3.0 equiv) in MeCN (2 mL) were used. After 20 h, the product was isolated by flash chromatography (PE: EA= 4:1) as a yellow solid (85.1 mg, 68%); ^{1}H NMR (600 MHz, $CDCl_3$) δ 7.99 (d, *J* = 8.2 Hz, 2H), 7.69 (d, *J* = 9.0 Hz, 1H), 7.55 (d, *J* = 8.2 Hz, 2H), 7.48 (d, *J* = 9.0 Hz, 1H), 7.14 (s, 1H), 7.06 (d, *J* = 9.0 Hz, 1H), 6.41 (d, *J* = 9.0 Hz, 1H), 4.64 – 4.60 (m, 1H), 4.40 – 4.35 (m, 2H), 3.07 – 2.97 (m, 1H), 2.48 – 2.39 (m, 1H), 1.36 (s, 9H); ^{19}F NMR (564 MHz, $CDCl_3$) δ -81.07 (t, *J* = 9.6 Hz, 3F), -112.37 – -112.60 (m, 2F), -124.31 (s, 2F), -125.92 – -126.03 (m, 2F). ^{13}C NMR (100 MHz, $CDCl_3$) δ 196.60, 160.02, 158.24, 154.60, 152.96, 152.38, 142.58, 132.65, 126.07, 117.48, 116.98, 116.41, 109.81, 68.56, 37.98, 35.23, 30.95, 29.50 (t, *J* = 21.7 Hz). HRMS (ESI+): calcd for $C_{28}H_{24}F_9O_6^+$ (M+H) 627.1424, found 627.1421.

2-(4-(*tert*-butyl)benzoyl)-4,4,5,5,6,6,7,7,7-nonafluoroheptyl (9,13,14-trimethyl-17-oxo-2,3,4,7,8,9,10,11,12,13,14,15,16,17-tetradecahydro-1H-cyclopenta[a]phenanthren-3-yl) carbonate (45)

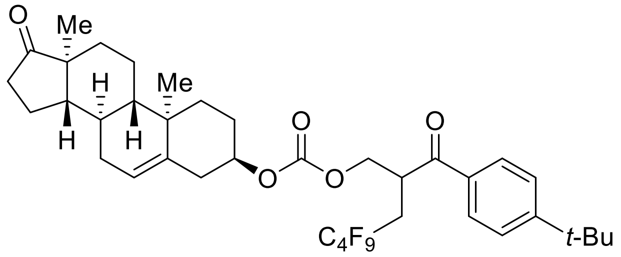

According to the general procedure, **S8** (74.5 mg, 0.20 mmol, 1.0 equiv.), C_4F_9I (34.4 μL, 0.20 mmol, 1.0 equiv.), 4-(*tert*-butyl)benzoyl chloride (58.6 μL, 0.30 mmol, 1.5 equiv.), $NiCl_2$•glyme (4.4 mg, 0.02 mmol, 0.1 eqiuv), dtbbpy (10.7 mg, 0.04 mmol, 0.2 equiv), and Mn power (33.0 mg, 0.3 mmol, 3.0 equiv) in MeCN/DME (4:1) (4 mL) were used. After 48 h, the product was isolated by flash chromatography (CH_2Cl_2: Et_2O= 120:1) as a colorless oil (94.8 mg, 63%); ^{1}H NMR (600 MHz, $CDCl_3$) δ 7.99 (d, *J* = 8.5 Hz, 2H), 7.54 (d, *J* = 8.5 Hz, 2H), 5.44 (d, *J* = 4.6 Hz, 1H), 4.50 – 4.44 (m,

2H), 4.36 – 4.28 (m, 1H), 4.18 – 4.12 (m, 1H), 3.09 – 2.95 (m, 1H), 2.50 – 2.46 (m, 1H), 2.43 – 2.32 (m, 3H), 2.15 – 2.07 (m, 2H), 2.04 – 1.85 (m, 5H), 1.70 – 1.66 (m, 3H), 1.62 – 1.46 (m, 3H), 1.37 (s, 9H), 1.31 – 1.26 (m, 2H), 1.17 – 1.12 (m, 1H), 1.04 (s, 3H), 0.90 (s, 3H). ^{19}F NMR (564 MHz, $CDCl_3$) δ -81.03 (t, *J* = 9.1 Hz), -112.08 – -112.74 (m), -124.25 (s), -125.84 – -126.02 (m). ^{13}C NMR (150 MHz, $CDCl_3$) δ 220.89, 196.89, 157.91, 154.07, 139.39 (d, *J* = 5.6 Hz), 132.83, 128.57, 125.97, 122.34 (d, *J* = 2.6 Hz), 78.21, 67.30, 51.70, 50.10, 47.51, 38.32, 37.81 (d, *J* = 12.2 Hz), 36.75, 36.65, 35.82, 35.23, 31.44, 31.39, 31.01, 30.76, 29.42 (t, *J* = 21.4 Hz), 27.46 (d, *J* = 8.5 Hz), 21.87, 20.33, 19.26, 13.54. HRMS (ESI+): calcd for $C_{38}H_{49}F_9NO_5^+$ (M+NH_4) 770.3462, found 770.3453.

2-(4-(*tert*-butyl)benzoyl)-4,4,5,5,6,6,7,7,7-nonafluoroheptyl (10,13-dimethyl-17-(6-methylheptan-2-yl)-2,3,4,7,8,9,10,11,12,13,14,15,16,17-tetradecahydro-1H-cyclopenta[a]phenanthren-3-yl) carbonate (46)

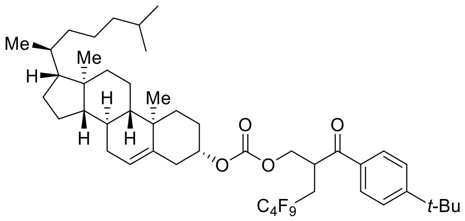

According to the general procedure, allyl ((3S,8S,9S,10R,13R,14S,17R)-10,13-dimethyl-17-((S)-6-methylheptan-2-yl)-2,3,4,7,8,9,10,11,12,13,14,15,16,17-tetradecahydro-1H-cyclopenta[a]phenanthren-3-yl) carbonate (94.1 mg, 0.20 mmol, 1.0 equiv.), C_4F_9I (34.4 μL, 0.20 mmol, 1.0 equiv.), 4-(*tert*-butyl)benzoyl chloride (58.6 μL, 0.30 mmol, 1.5 equiv.), $NiCl_2$•glyme (4.4 mg, 0.02 mmol, 0.1 eqiuv), dtbbpy (10.7 mg, 0.04 mmol, 0.2 equiv), and Mn power (33.0 mg, 0.3 mmol, 3.0 equiv) in MeCN/DME (4:1) (4 mL) were used. After 48 h, the product was isolated by flash chromatography (hexanes: Et_2O= 60:1) as a colorless oil (88.5 mg, 52%); ^{1}H NMR (600 MHz, $CDCl_3$) δ 8.00 (d, *J* = 8.5 Hz, 2H), 7.55 (d, *J* =

8.5 Hz, 2H), 5.44 – 5.39 (m, 1H), 4.51 – 4.43 (m, 2H), 4.36 – 4.30 (m, 1H), 4.19 – 4.12 (m, 1H), 3.12 – 2.94 (m, 1H), 2.47 – 2.31 (m, 3H), 2.07 – 1.99 (m, 2H), 1.93 – 1.83 (m, 3H), 1.66 – 1.42 (m, 9H), 1.37 (s, 9H), 1.32 – 1.25 (m, 2H), 1.23 – 1.06 (m, 8H), 1.02 (s, 3H), 0.94 (d, J = 6.4 Hz, 3H), 0.90 – 0.87 (m, 6H), 0.70 (s, 3H). ^{19}F NMR (564 MHz, $CDCl_3$) δ -81.03 (t, J = 9.3 Hz), -112.08 – -112.69 (m), -124.24 (s), -125.84 – -126.03 (m). ^{13}C NMR (150 MHz, $CDCl_3$) δ 196.95, 157.90 , 154.10, 139.12 (d, J = 5.4 Hz), 132.86, 128.58, 125.97, 123.12 (d, J = 2.6 Hz), 78.53, 67.27, 56.69, 56.15, 50.00, 42.32, 39.72, 39.53, 38.34, 37.87 (d, J = 12.5 Hz), 36.82, 36.51, 36.19, 35.80, 35.23, 31.87 (d, J = 8.9 Hz), 31.02, 29.43 (t, J = 21.4 Hz), 28.23, 28.02, 27.55 (d, J = 8.8 Hz), 24.28, 23.84, 22.69 (d, J = 38.7 Hz), 21.05, 19.24, 18.72, 11.86. HRMS (ESI+): calcd for $C_{46}H_{67}F_9NO_4^+$ (M+NH$_4$) 868.4921, found 868.4911.

Derivations of compound 3.

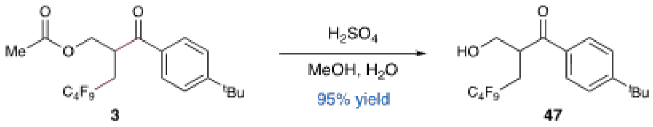

Supplementary Figure 1. Deprotection of the acetyl group

A mixture of **3** (48.0 mg, 0.1 mmol), MeOH (0.12 ml), H_2O (11 μL, 0.6 mmol, 6.0 equiv.), and concentrated sulfuric acid (9 μL, 0.15 mmol, 1.5 equiv.) was stirred at room temperature for 23 h. A saturated aqueous solution of $NaHCO_3$ was cautiously added and the mixture was partitioned between EtOAc and H_2O. The separated organic layer was washed with brine and dried over anhydrous magnesium sulfate. The solvents were removed *in vacuo* and the residual oil was chromatographed on silica gel (PE: EA= 5:1) to afford the product as a colorless oil liquid (41.7 mg, 95%).

1-(4-(tert-Butyl)phenyl)-4,4,5,5,6,6,7,7,7-nonafluoro-2-(hydroxymethyl)heptan-1-one (47). ^{1}H NMR (600 MHz, $CDCl_3$) δ 7.94 (d, *J* = 8.5 Hz, 2H), 7.52 (d, *J* = 8.5 Hz, 2H), 4.10 – 4.04 (m, 1H), 3.96 – 3.90 (m, 1H), 3.89 – 3.83 (m, 1H), 2.91 – 2.76 (m, 1H), 2.57 – 2.42 (m, 1H), 2.10 (s, 1H), 1.35 (s, 9H). ^{19}F NMR (564 MHz, $CDCl_3$) δ -81.04 (t, *J* = 9.6 Hz, 3F), -111.84 – -113.33 (m, 2F), -124.34 (s, 2F), -125.83 – -126.06 (m, 2F). ^{13}C NMR (150 MHz, $CDCl_3$) δ 200.18, 157.93, 132.92, 128.58, 125.95, 63.43, 40.44, 35.23, 31.02, 29.32 (t, *J* = 21.2 Hz). HRMS (ESI+): calcd for $C_{18}H_{20}F_9O_2^+$ (M+H) 439.1314, found 439.1320.

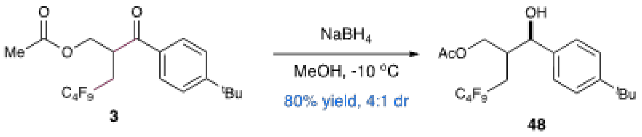

Supplementary Figure 2. Reduction of ketone

In a 10 mL round-bottom flask equipped with a magnetic stirrer bar were placed with **3** (48.0 mg, 0.1 mmol, 1.0 equiv.) in MeOH (1 mL) under an N_2 atmosphere, and the solution was cooled at -10 °C. $NaBH_4$ (18.8 mg, 0.5 mmol, 5.0 equiv.) was added, and the solution was stirred at -10 °C for 4 h. Then, saturated $NaHCO_3$ solution and DCM were added, and the mixture was stirred for 5 min. The organic layer was concentrated under reduced pressure, the product was isolated by flash chromatography (PE : EA = 8:1) as a colorless oil (38.6 mg, 80%, d.r. 4:1);

2-((4-(tert-Butyl)phenyl)(hydroxy)methyl)-4,4,5,5,6,6,7,7,7-nonafluoroheptyl acetate (48) ^{1}H NMR (400 MHz, $CDCl_3$) δ 7.39 (d, J = 8.3 Hz, 2H), 7.25 (d, J = 8.3 Hz, 2H), 4.85 – 4.76 (m, 1H), 4.40 – 4.31 (m, 0.8H), 4.31 – 4.25 (m, 0.2H), 4.14 – 4.09 (m, 0.8H), 3.97 – 3.90 (m, 0.2H), 2.64 – 2.08 (m, 4H), 2.06 (s, 0.8H), 2.01 (s, 2.4H), 1.32 (s, 9H); ^{19}F NMR (375 MHz, $CDCl_3$) δ -80.80 – -81.20 (m), -111.10 –

-114.57 (m), -124.46 (d, J = 8.6 Hz), -125.83 – -126.15 (m); ^{13}C NMR (100 MHz, $CDCl_3$) δ 171.03, 151.21, 138.11, 125.80, 125.73, 125.59, 73.49, 72.93, 63.90, 62.73, 38.61, 38.08, 34.54, 31.25, 28.31 (t, J = 21.2 Hz), 20.71. HRMS (ESI+): calcd for $C_{20}H_{24}F_9O_3^+$ (M+H) 483.1576, found 483.1579.

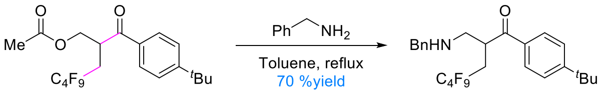

Supplementary Figure 3. Nucleophilic substitution with benzylamine

In a 10 mL round-bottom flask equipped with a magnetic stirrer bar were placed with **3** (48.0 mg, 0.1 mmol, 1.0 equiv.), aniline (36.0 μL, 0.3 mmol, 3.0 equiv.) and 4Å molecular sieves (50 mg) in toluene (1 mL) under an N_2 atmosphere. The reaction mixture was heated to reflux for 4 h. Then the mixture was cooled, and filtered through Celite, washed with EA, and concentrated in vacuum. The product was isolated by flash chromatography (PE: EA = 30:1 with 3% Et_3N) as a pale-yellow oil (36.9 mg, 70%);

2-((Benzylamino)methyl)-1-(4-(tert-butyl)phenyl)-4,4,5,5,6,6,7,7,7-nonafluoroheptan-1-one (49) ^{1}H NMR (400 MHz, $CDCl_3$) δ 7.89 (d, J = 8.5 Hz, 2H), 7.49 (d, J = 8.5 Hz, 2H), 7.31 – 7.19 (m, 5H), 4.07 – 3.97 (m, 1H), 3.75 (s, 2H), 3.03 – 2.97 (m, 1H), 2.97 – 2.82 (m, 1H), 2.81 – 2.72 (m, 1H), 2.58 – 2.40 (m, 1H), 1.56 (brs, 1H), 1.35 (s, 9H); ^{19}F NMR (375 MHz, $CDCl_3$) δ -80.02 (t, J = 9.5Hz, 3F), -111.39 – -113.38 (m, 2F), -124.26 – -124.41 (m, 2F), -125.94 (dd, J = 22.9, 11.0 Hz, 2F); ^{13}C NMR (100 MHz, $CDCl_3$) δ 200.09, 157.39, 139.76, 133.44, 128.39, 127.99, 127.08, 125.78, 53.59, 51.19, 38.97, 35.15, 31.03, 30.33 (t, J = 21.3 Hz). HRMS (ESI+): calcd for $C_{25}H_{27}F_9ON^+$ (M+H) 528.1943, found 528.1942.

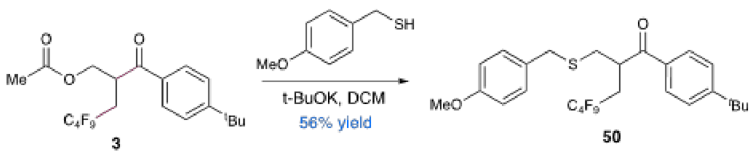

Supplementary Figure 4. Conversion to thioether

To a solution of **3** (48.0 mg, 0.1 mmol) in 2 ml DCM, *t*-BuOK (33.7 mg, 0.3 mmol, 3.0 equiv.) was added in portions at 0 °C under N_2 atmosphere, followed by the addition of (4-methoxyphenyl)methanethiol (27.8 μL, 0.2 mmol, 3.0 equiv.) at 0 °C. The mixture was stirred overnight at room temperature. Then, water was added, the layer was separated and the aqueous layer was extracted three times with DCM. The combined organic layers were dried over $MgSO_4$ and concentrated in vacuo. Purification by flash chromatography (PE: EA= 40:1) yielded the product as a colorless oil liquid (31.0 mg, 56%).

1-(4-(tert-Butyl)phenyl)-4,4,5,5,6,6,7,7,7-nonafluoro-2-(((4-methoxybenzyl)thio)methyl)heptan-1-one (50) ^{1}H NMR (400 MHz, $CDCl_3$) δ 7.74 (d, J = 8.5 Hz, 2H), 7.46 (d, J = 8.5 Hz, 2H), 7.19 (d, J = 8.5 Hz, 2H), 6.84 (d, J = 8.6 Hz, 2H), 3.92 – 3.85 (m, 1H), 3.80 (s, 3H), 3.67 (s, 2H), 2.95 – 2.80 (m, 2H), 2.58 – 2.42 (m, 2H), 1.35 (s, 9H). ^{19}F NMR (375 MHz, $CDCl_3$) δ -81.01 (t, J = 9.5 Hz, 3F), -111.07 – -113.18 (m, 2F), -124.31 (d, J = 7.9 Hz, 2F), -125.77 – -126.26 (m, 2F). ^{13}C NMR (150 MHz, $CDCl_3$) δ 199.02, 158.88, 157.57, 132.89, 129.98, 129.23, 128.44, 125.78, 114.07, 55.24, 38.07, 35.92, 35.17, 33.86, 31.23 (t, J = 20.9 Hz), 31.02. HRMS (ESI+): calcd for $C_{26}H_{28}F_9O_2S^+$ (M+H) 575.1661, found 575.1668.

Supplementary Discussion

Evaluation of the stirring rates:

To a flame-dried 8 mL reaction vial was charged with $NiCl_2$•glyme (2.2 mg, 0.01 mmol, 10 mol%), dtbbpy (5.4 mg, 0.02 mmol, 20 mol%), and Mn (16.5 mg, 0.3 mmol, 3.0 equiv.). The vial was capped. After it was evacuated and backfilled nitrogen three times, CH_3CN (1 mL) was added via a syringe, followed by the addition of 4-(*tert*-butyl)benzoyl chloride (29.5 µL, 0.15 mmol, 1.5 equiv.). The reaction mixture was allowed to stir for approximately 1 minute before C_4F_9I (17.2 µL, 0.1 mmol, 1.0 equiv.) and allyl heptanoate (19.2 µL, 0.1 mmol, 1.0 equiv.) were added. The reaction mixture was allowed to stir at 1500 rpm for 20 h at 25 ^{o}C. The reaction mixture was analyzed by ^{1}H NMR with an internal standard.

Supplementary Table 2. Evaluation of the stirring rates

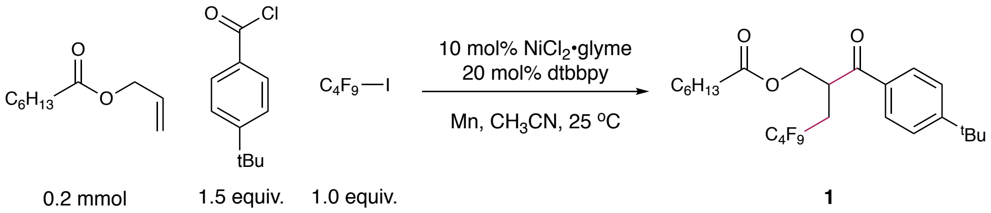

Entry	stirring rate	Yield of **1**
1	1500 rpm (standard)	93%
2	800 rpm	76%
3	500 rpm	69%

Conclusion: A stirring rate of 1500 rpm is necessary for high reaction efficiency and reproducibility. We also sometimes observed that the reaction mixtures remained clear with low stirring rates, which led to no formation of products.

Other successful and unsuccessful substrates:

Acyclic internal alkenes:

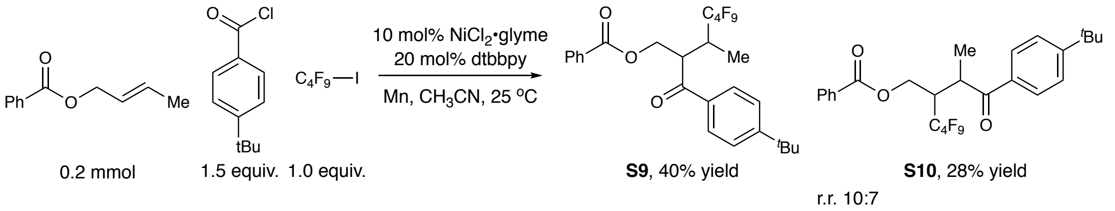

Supplementary Figure 5. Reaction of acyclic internal alkenes

The reaction of (*E*)-but-2-en-1-yl benzoate under the standard condition afforded the desired product **S9** in 40% yield as well as the regioisomeric product **S10** in 28% yield (r.r. 10:7).

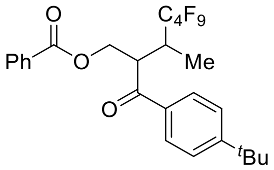

2-(4-(tert-Butyl)benzoyl)-4,4,5,5,6,6,7,7,7-nonafluoro-3-methylheptyl benzoate (S9). According to the general procedure, (Z/E)-but-2-en-1-yl benzoate (34.0 μL, 0.20 mmol, 1.0 equiv.), C_4F_9I (34.4 μL, 0.20 mmol, 1.0 equiv.), 4-(tert-butyl)benzoyl chloride (58.6 μL, 0.30 mmol, 1.5 equiv.), $NiCl_2$•glyme (4.4 mg, 0.02 mmol, 0.1 equiv.), dtbbpy (10.7 mg, 0.04 mmol, 0.2 equiv.), and Mn power (33.0 mg, 0.3 mmol, 3.0 equiv.) in MeCN (2 mL) were used. After 20 h, the product was isolated by flash chromatography (PE: EA= 60:1) as a white solid (44.5 mg, 40%); ^{1}H NMR (600 MHz, $CDCl_3$) δ 7.94 (d, *J* = 8.5 Hz, 2H), 7.76 (d, *J* = 8.4 Hz, 2H), 7.52 – 7.47 (m, 3H), 7.31 (t, *J* = 7.8 Hz, 2H), 4.72 – 4.67 (m, 1H), 4.67 – 4.61 (m, 1H), 4.38 – 4.32 (m, 1H), 3.31 – 3.18 (m, 1H), 1.34 (s, 9H), 1.31 – 1.29 (m, 3H). ^{19}F NMR (564 MHz, $CDCl_3$) δ -80.87 (t, *J* = 9.8 Hz, 3F), -108.81 – -119.33 (m, 2F), -120.47 – -122.04 (m, 2F), -124.63 – -127.54 (m, 2F). ^{13}C NMR (150 MHz, $CDCl_3$) δ 197.97, 166.03, 157.61, 133.15, 133.10, 129.55, 129.43, 128.45, 128.25, 125.93, 62.63, 42.07, 35.97 (t, *J* = 20.4 Hz), 35.20, 31.05, 29.71. HRMS (ESI+): calcd for $C_{26}H_{26}F_9O_3^+$ (M+H) 557.1733, found 557.1730.

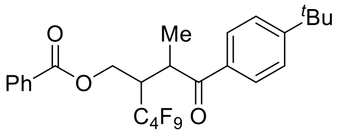

2-(1-(4-(tert-Butyl)phenyl)-1-oxopropan-2-yl)-3,3,4,4,5,5,6,6,6-nonafluorohexyl benzoate (S10). According to the general procedure, (Z/E)-but-2-en-1-yl benzoate (34.0 μL, 0.20 mmol, 1.0 equiv.), C_4F_9I (34.4 μL, 0.20 mmol, 1.0 equiv.), 4-(tert-butyl)benzoyl chloride (58.6 μL, 0.30 mmol, 1.5 equiv.), $NiCl_2$•glyme (4.4 mg, 0.02 mmol, 0.1 equiv.), dtbbpy (10.7 mg, 0.04 mmol, 0.2 equiv.), and Mn power (33.0 mg, 0.3 mmol, 3.0 equiv.) in MeCN (2 mL) were used. After 20 h, the product was isolated by flash chromatography (PE: EA= 60:1) as a white solid (31.2 mg, 28%); ^{1}H NMR (600 MHz, $CDCl_3$) δ 8.01 (d, J = 7.2 Hz, 2H), 7.89 (d, J = 8.5 Hz, 2H), 7.60 (t, J = 7.4 Hz, 1H), 7.51 (d, J = 8.5 Hz, 2H), 7.47 (t, J = 7.8 Hz, 2H), 4.69 (d, J = 3.7 Hz, 2H), 4.16 – 4.09 (m, 1H), 3.60 (d, J = 24.0 Hz, 1H), 1.35 (s, 9H), 1.26 (s, 3H). ^{19}F NMR (564 MHz, $CDCl_3$) δ -80.87 (t, J = 9.8 Hz, 3F), -107.68 – -116.02 (m, 2F), -120.67 – -123.22 (m, 2F), -124.22 – -127.80 (m, 2F). ^{13}C NMR (150 MHz, $CDCl_3$) δ 200.01, 165.89, 157.34, 133.32, 132.34, 129.70, 129.48, 128.49, 128.38, 125.87, 59.50, 41.32 (t, J = 19.8 Hz), 35.17, 31.05, 29.71, 14.40. HRMS (ESI+): calcd for $C_{26}H_{26}F_9O_3^+$ (M+H) 557.1733, found 557.1730.

Trifluoroiodomethane:

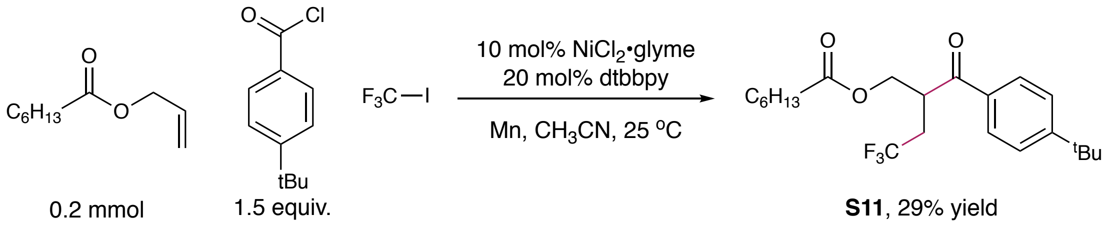

Supplementary Figure 6. Reaction of CF_3I

The reaction of CF_3I under the standard condition afforded the desired trifluoromethylacylated product **S11** in 29% yield (37% ^{19}F NMR yield).

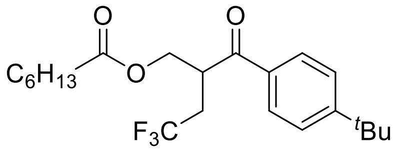

2-(4-(tert-Butyl)benzoyl)-4,4,4-trifluorobutyl heptanoate (S11). According to the general procedure, allyl heptanoate (38.5 μL, 0.20 mmol, 1.0 equiv.), CF_3I balloon, 4-(tert-butyl)benzoyl chloride (58.6 μL, 0.30 mmol, 1.5 equiv.), $NiCl_2$•glyme (4.4 mg, 0.02 mmol, 0.1 equiv.), dtbbpy (10.7 mg, 0.04 mmol, 0.2 equiv.), and Mn power (33.0 mg, 0.3 mmol, 3.0 equiv.) in MeCN (2 mL) were used. After 20 h, the product was isolated by flash chromatography (PE: EA= 80:1) as a yellow oil liquid (23.5 mg, 29%); ^{1}H NMR (600 MHz, $CDCl_3$) δ 7.96 (d, J = 8.5 Hz, 2H), 7.54 (d, J = 8.5 Hz, 2H), 4.43 – 4.39 (m, 1H), 4.19 – 4.11 (m, 2H), 2.97 – 2.86 (m, 1H), 2.45 – 2.35 (m, 1H), 2.24 (td, J = 7.5, 3.1 Hz, 2H), 1.56 – 1.50 (m, 2H), 1.37 (s, 9H), 1.28 – 1.25 (m, 6H), 0.89 (t, J = 7.1 Hz, 3H). ^{19}F NMR (564 MHz, $CDCl_3$) δ -64.72 (t, J = 10.9 Hz, 3F). ^{13}C NMR (150 MHz, $CDCl_3$) δ 197.51, 173.40, 157.80, 133.20, 128.49, 126.35 (q, J = 276.7 Hz), 125.90, 64.17, 39.58, 35.22, 34.01, 32.56 (q, J = 29.3 Hz), 31.38 , 31.03, 28.71, 24.69, 22.44, 14.01. HRMS (ESI+): calcd for $C_{22}H_{32}F_3O_3^+$ (M+H) 401.2298, found 401.2303.

Tertiary alkyl halides:

Simple tertiary alkyl bromides were also viable coupling partners under slightly modified conditions. For example, the reaction of ethyl 2-bromo-2-methylpropanoate gave the desired carboacylated product **S12** in 36% yield.

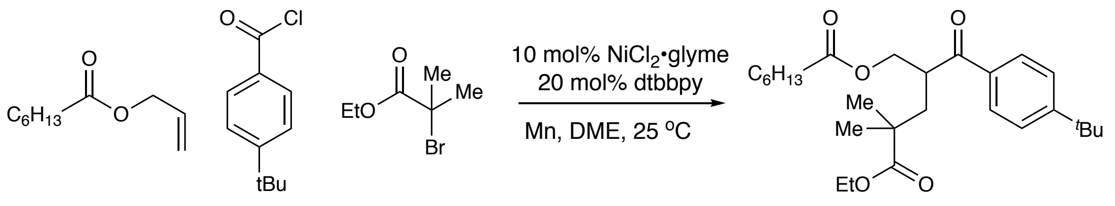

Supplementary Figure 7. Reaction of tertiary alkyl bromides.

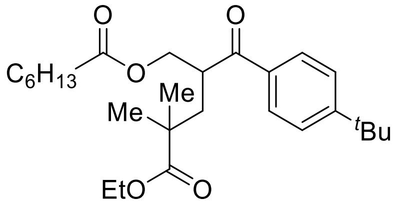

2-(4-(tert-Butyl)benzoyl)-5-ethoxy-4,4-dimethyl-5-oxopentyl heptanoate (S12): According to the general procedure, allyl heptanoate (76.9 μL, 0.40 mmol, 2.0 equiv.), ethyl 2-bromo-2-methylpropanoate (29.4 μL, 0.20 mmol, 1.0 equiv.), 4-(tert-butyl)benzoyl chloride (58.6 μL, 0.30 mmol, 1.5 equiv.), $NiCl_2$•glyme (4.4 mg, 0.02 mmol, 0.1 equiv.), dtbbpy (10.7 mg, 0.04 mmol, 0.2 equiv.), and Mn power (33.0 mg, 0.3 mmol, 3.0 equiv.) in DME (2 mL) were used. After 24 h, the product was isolated by flash chromatography (PE: EA= 40:1) as a colorless oil liquid (32.2 mg, 36%); ^{1}H NMR (600 MHz, $CDCl_3$) δ 7.92 (d, J = 8.5 Hz, 2H), 7.48 (d, J = 8.5 Hz, 2H), 4.21 – 4.17 (m, 1H), 4.11 – 4.06 (m, 1H), 4.04 – 3.97 (m, 2H), 3.92 – 3.86 (m, 1H), 2.31 – 2.25 (m, 1H), 2.16 – 2.05 (m, 2H), 1.78 – 1.73 (m, 1H), 1.46 – 1.40 (m, 2H), 1.33 (s, 9H), 1.27 – 1.21 (m, 6H), 1.18 (s, 3H), 1.17 (t, J = 7.2 Hz, 3H), 1.07 (s, 3H), 0.84 (t, J = 7.2 Hz, 3H). ^{13}C NMR (150 MHz, $CDCl_3$) δ 200.84 (s), 177.21, 173.54, 157.03, 134.44, 128.38, 125.65, 66.27, 60.58, 42.00, 41.98, 38.81, 35.12, 34.07, 31.38, 31.07, 28.73, 26.30, 25.04, 24.61, 22.43, 14.05, 14.00. HRMS (ESI+): calcd for $C_{27}H_{43}O_5^+$ (M+H) 447.3105, found 447.3111.

Unsuccessful alkenes and acyl electrophiles:

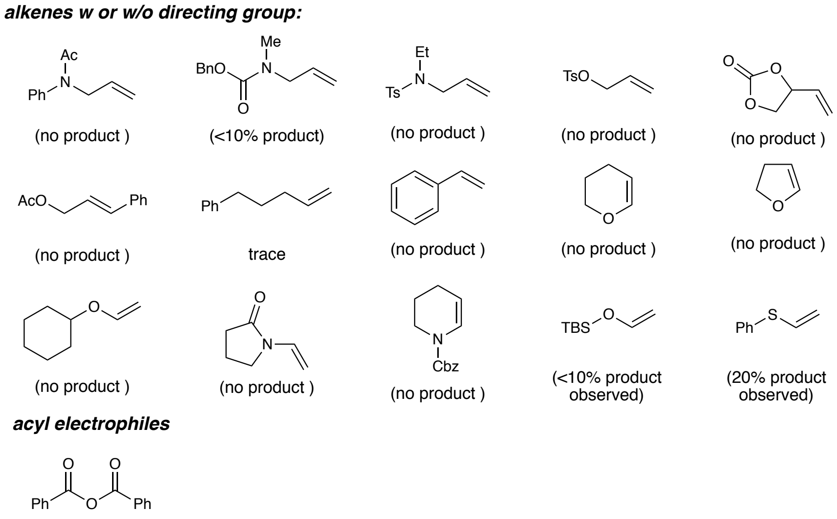

Supplementary Figure 8. Failed substrates.

Evaluation of bis-functionalization of diene with excess amounts of reagents:

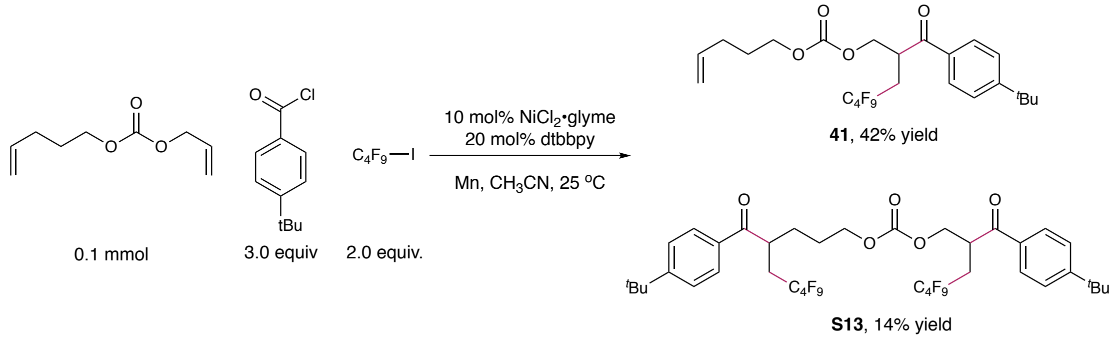

Supplementary Figure 9. Bis-functionalization of diene.

According to the general procedure, allyl pent-4-en-1-yl carbonate (36.6 μL, 0.20 mmol, 1.0 equiv.), C_4F_9I (68.8 μL, 0.40 mmol, 2.0 equiv.), 4-(tert-butyl)benzoyl chloride (117.2 μL, 0.60 mmol, 3.0 equiv.), $NiCl_2$•glyme (4.4 mg, 0.02 mmol, 0.1 equiv.), dtbbpy (10.7 mg, 0.04 mmol, 0.2 equiv.), and Mn power (33.0 mg, 0.3 mmol, 3.0 equiv.) in MeCN (2 mL) were used. After 24 h, the products were isolated by flash chromatography (PE: EA = 20:1). Mono-functionalization product **41** (pale-yellow liquid, 46.0 mg, 42%); bis-functionalization product **S13** (yellow oil, 25.7 mg, 14%).

2-(4-(tert-Butyl)benzoyl)-4,4,5,5,6,6,7,7,7-nonafluoroheptyl (4-(4-(tert-butyl) benzoyl)-6,6,7,7,8,8,9,9,9-nonafluorononyl) carbonate (S13).

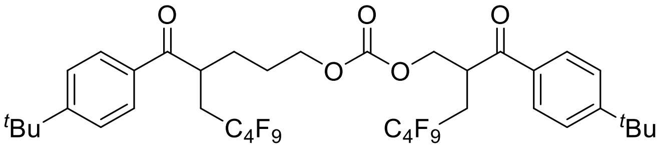

^{1}H NMR (600 MHz, $CDCl_3$) δ 7.97 – 7.93 (m, 2H), 7.91 (d, J = 7.5 Hz, 2H), 7.54 – 7.48 (m, 4H), 4.49 – 4.43 (m, 1H), 4.29 – 4.23 (m, 1H), 4.17 – 4.10 (m, 1H), 4.10 – 4.03 (m, 2H), 3.94 – 3.88 (m, 1H), 3.04 – 2.87 (m, 2H), 2.45 – 2.30 (m, 1H), 2.23 – 2.10 (m, 1H), 1.89 – 1.78 (m, 1H), 1.71 – 1.60 (m, 3H), 1.36 – 1.33 (m, 18H); ^{19}F NMR (564 MHz, $CDCl_3$) δ -81.00-81.14 (m, 6F), -112.32 – -112.78 (m, 4F), -124.17- -124.93 (m, 4F), -125.91- -126.03 (m, 4F). ^{13}C NMR (150 MHz, $CDCl_3$) δ 200.23, 196.69, 158.00, 157.61, 154.62, 133.25, 132.67, 128.53, 128.34, 125.97, 125.92, 67.76, 67.41, 38.35, 37.27, 35.22, 35.17, 31.01, 30.99, 29.75, 29.70, 29.37 (t, J = 20.7 Hz), 25.85. HRMS (ESI+): calcd for $C_{39}H_{41}F_{18}O_5^+$ (M+H) 931.2661, found 931.2669.

Regioselectivity discussions:

Regarding the regioselectivity issue, we have carefully reevaluated all NMR data, GC-MS data, and several crude NMR and GC-MS data, and can conclude that regioselectivity is very high for these terminal alkenes. Some of the relevant analysis has been highlighted bellows:

Crude NMR analysis:

Since the ^{19}F NMR spectra of the regioisomers should be identifiably different, we have carefully reevaluated the ^{19}F NMR spectra of the crude reaction mixtures, especially for the cases of ICF_2CO_2Et and CF_3I.

For the case of ICF_2CO_2Et, the CF_2 group for the desired product should be doublet of triplets (dt), while the CF_2 group for the regioisomer would be doublet of doublets of doublets (ddd).

In the crude ^{19}F NMR of the reaction mixture, only one set of ^{19}F NMR peaks (dt) has been observed, which is the same as the final pure product compound (dt).

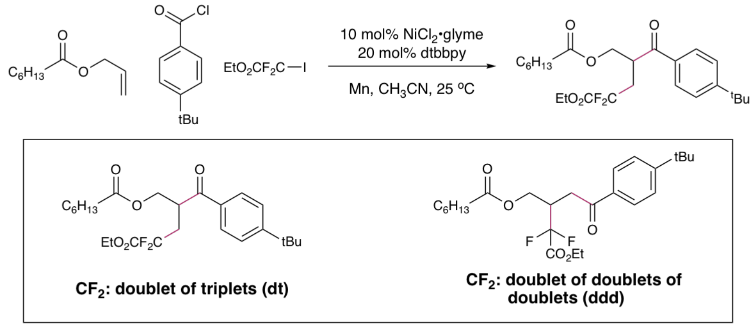

The crude ^{19}F NMR of ICF_2CO_2Et:

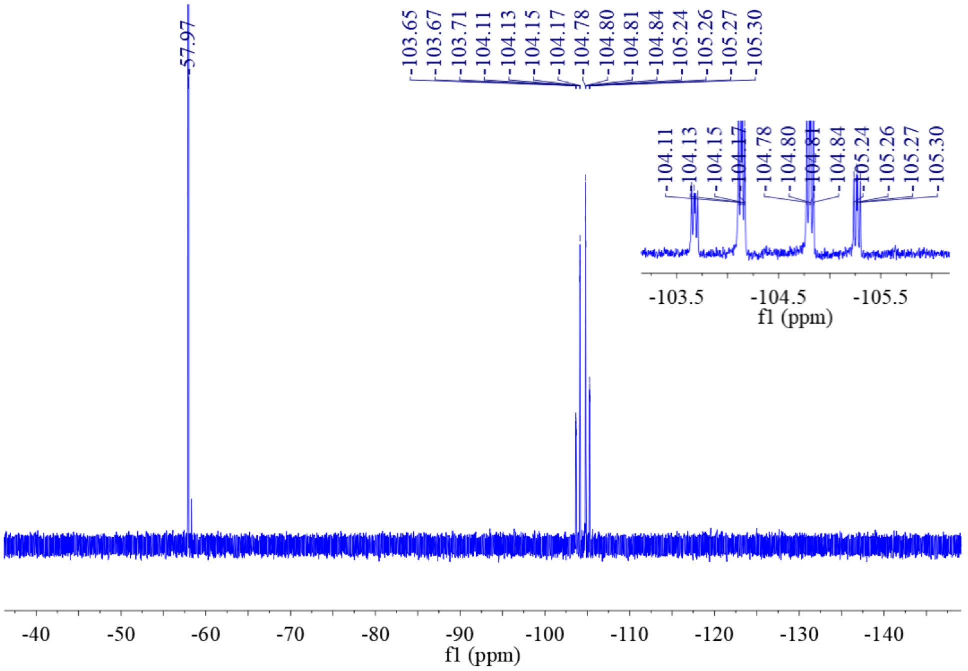

Supplementary Figure 10. The crude ^{19}F NMR of ICF_2CO_2Et

For the case of CF_3I, the CF_3 group for the desired product is triplet (t), while the CF_3 group for the regioisomer should be doublet (d).

In the crude ^{19}F NMR, the desired CF_3-product is present (-64.25 ppm, triplet, (J = 11.3 Hz)). There are some unidentified byproducts which appear as singlet peaks in the crude ^{19}F NMR, which does not suggest the presence of the regioisomer.

Besides CF_3H (-78.41 ppm, doublet, J = 39.5 Hz), only one double peak (-68.97 ppm, doublet, J = 2.8 Hz) has been observed in low yield (1% yield in ^{19}F NMR). This doublet peak was inseparable to the desired product, and the ratio of these two compounds is 1:150 in the final spectrum of the isolated product. We couldn't rule out the possibility that this minor peak is the regioisomeric product. Nevertheless, the regioselectivity for this transformation is still high (grear than the limit of precision by NMR analysis).

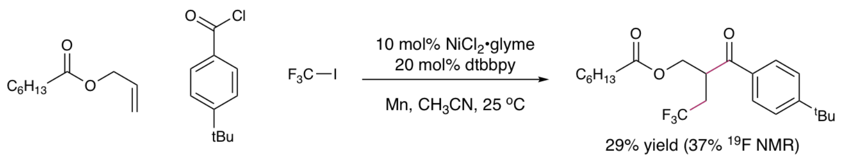

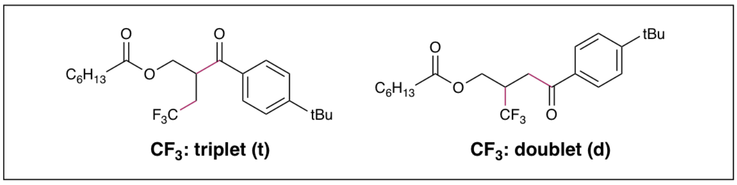

The crude ^{19}F NMR of CF_3I:

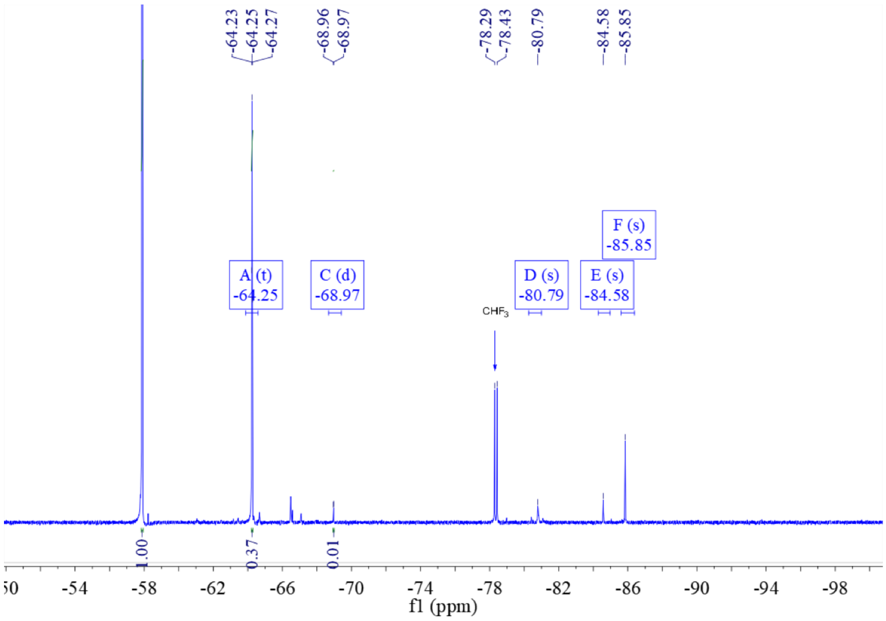

Supplementary Figure 11. The crude ^{19}F NMR of CF_3I

For the case of C_4F_9I, only one set of ^{19}F NMR peaks, which is the same as the final product, has been observed in the crude ^{19}F NMR of the reaction mixture.

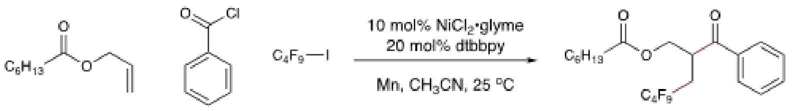

The crude ^{19}F NMR of C_4F_9I:

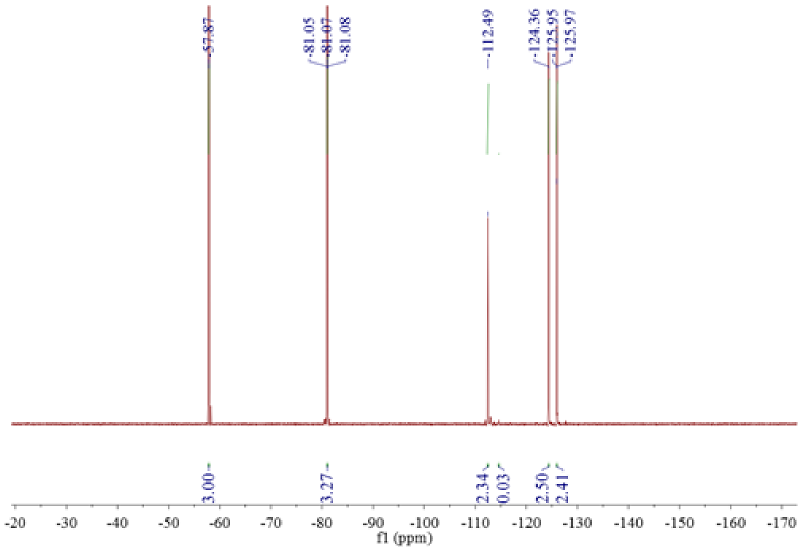

Supplementary Figure 12. The crude ^{19}F NMR of C_4F_9I

Characterization of impurities

One reviewer questioned whether the minor impurities showed in the spectra of several products are the regioisomeric products or not.

For the cases of **19** and **25**, we have successfully isolated the primary impurities appeared in ^{1}H NMR spectra. The primary impurities are the β-eliminated byproducts of the final products, not the regioisomeric products.

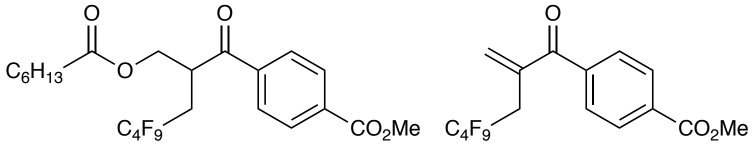

19 impurity

Supplementary Figure 13. The primary impurity of 19 in ^{1}H NMR spectra

Methyl 4-(4,4,5,5,6,6,7,7,7-nonafluoro-2-methyleneheptanoyl)benzoate (S14).

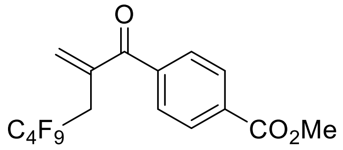

^{1}H NMR (600 MHz, $CDCl_3$) δ 8.13 (d, J = 8.3 Hz, 2H), 7.81 (d, J = 8.3 Hz, 2H), 6.26 (s, 1H), 6.00 (s, 1H), 3.96 (s, 3H), 3.39 (t, J = 18.7 Hz, 2H); ^{19}F NMR (564 MHz, $CDCl_3$) δ -80.86 – -81.44 (m, J = 9.4, 2.8 Hz, 3F), -112.44 – -112.84 (m, 2F), -123.73 – -124.05 (m, 2F), -125.71 – -126.07 (m, 2F); ^{13}C NMR (150 MHz, $CDCl_3$) δ 195.08, 166.15, 140.16, 136.77, 133.57, 133.35, 129.60, 129.40, 52.48, 32.55 (t, J = 21.9 Hz); GC-MS: 422 (6), 391 (14), 363 (2),203 (4), 163 (100), 135 (18), 120 (6).

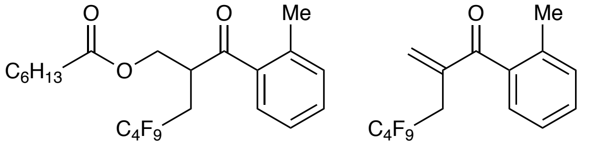

25 impurity

Supplementary Figure 14. The primary impurity of 25 in ^{1}H NMR spectra

4,4,5,5,6,6,7,7,7-Nonafluoro-2-methylene-1-(o-tolyl)heptan-1-one (S15).

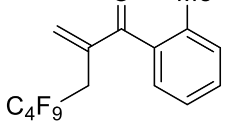

^{1}H NMR (600 MHz, $CDCl_3$) δ 7.36 (t, *J* = 7.5 Hz, 1H), 7.28 (d, *J* = 6.9 Hz, 1H), 7.25 (d, *J* = 7.4 Hz, 1H), 7.23 (t, *J* = 7.5 Hz, 1H), 6.27 (s, 1H), 5.98 (s, 1H), 3.37 (t, *J* = 19.1 Hz, 2H), 2.33 (s, 3H). ^{19}F NMR (564 MHz, $CDCl_3$) δ -81.04 (t, *J* = 9.6 Hz, 3F), -112.80 – -113.22 (m, 2F), -123.87 – -124.15 (m, 2F), -125.67 – -126.18 (m, 2F). ^{13}C NMR (150 MHz, $CDCl_3$) δ 197.80, 138.21, 137.36, 136.74, 135.34, 131.03, 130.41, 128.32, 125.17, 30.72 (t, *J* = 22.0 Hz), 19.66. GC-MS: 378.1.

Mechanistic investigations.

Plausible mechanisms involving radical intermediates

Two plausible pathways involving radical intermediates have been outlined in Supplementary Figure 15 and 16.

Pathway I:

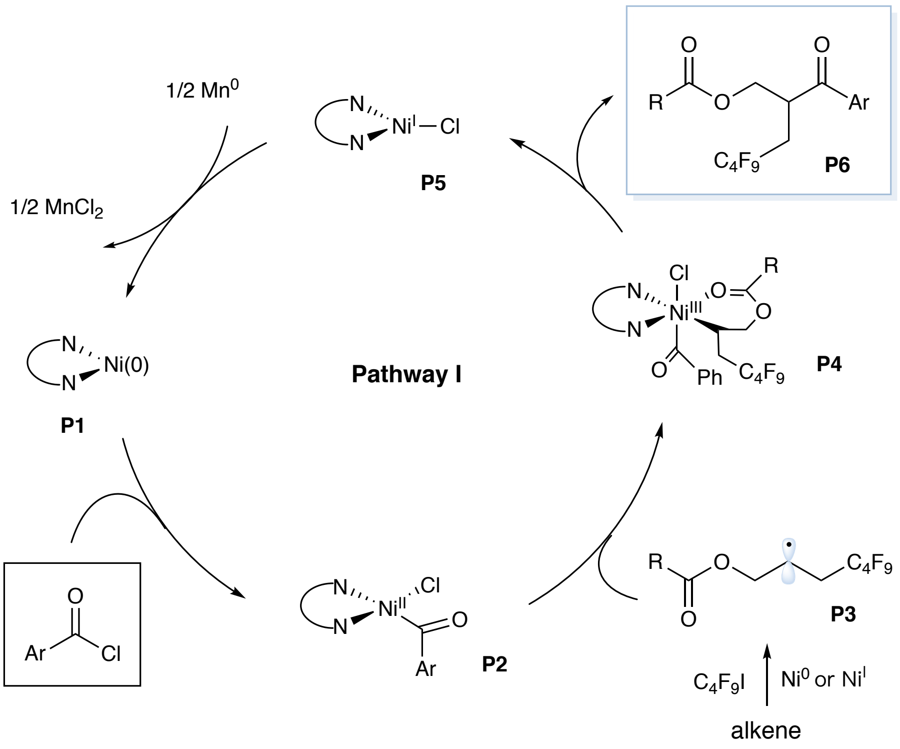

Supplementary Figure 15. Plausible mechanisms of pathway I

Oxidative addition of the active Ni(0) species **P1** to acyl chloride affords Ni^{II} complex **P2**. Concurrently, Ni(0)- or Ni^{I}-mediated single-electron reduction of C_4F_9I generates the electrophilic C_4F_9 radical, and subsequent radical addition to the alkene coupling partner would deliver the alkyl radical species **P3**. At this juncture, we expected that Ni^{II} complex **P2** could be intercepted by the nucleophilic alkyl radical **P3** to yield Ni^{III} adduct **P4**, which would undergo reductive elimination to deliver the final product **P6** and Ni^{I} species **P5**. Single-electron reduction of Ni^{I} **P5** (E_{red} [Ni^{II}/Ni^{0}] = –1.2 V vs SCE in DMF)[2] by Mn dust (E_{red} = –1.4 V vs SCE in MeCN) would regenerate Ni(0) species and complete the catalytic cycle.

Pathway II:

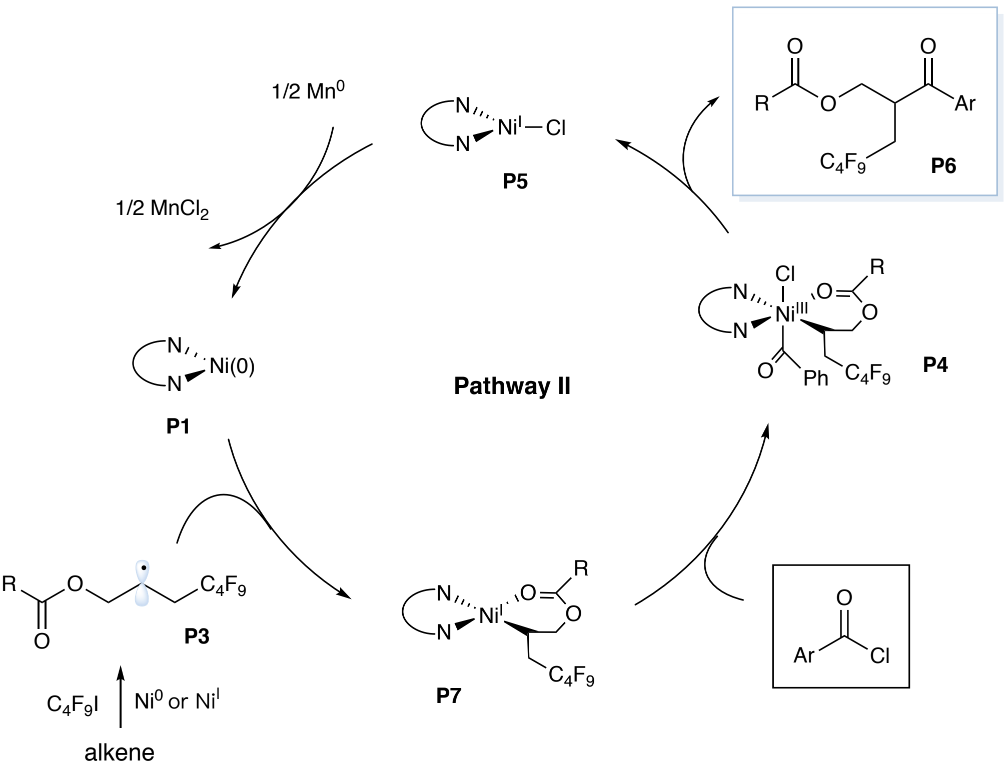

Supplementary Figure 16. Plausible mechanisms of pathway II

Being different from pathway **I**, in pathway **II**, alkyl radical **P3** could be captured by Ni(0) to form Ni^{I} complex **P7**, followed by oxidative addition of acyl chloride to deliver the crucial Ni^{III} adduct **P4**.

Preliminary mechanistic studies

Stoichiometric reaction of isolated Ac-Ni^{II} complex

The stoichiometric reaction of Ac-Ni^{II} complex **55** (prepared according to the previously reported procedure[3]) with alkene and C_4F_9I in the presence of Mn dust gave the desired coupling product **16** in 42% yield.

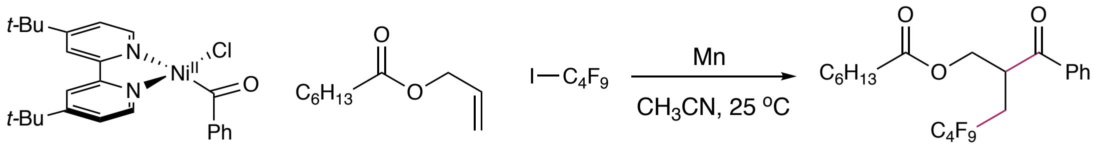

55, 0.1 mmol   4.0 equiv   4.0 equiv   **16**, 42% yield

Supplementary Figure 17. The stoichiometric reaction of Ac-Ni^{II} complex 55.

In a nitrogen-filled glovebox, to a flame-dried 8 mL reaction vial was charged with Mn (8.2 mg, 0.15 mmol, 3.0 equiv.), **55** (23.4 mg, 0.05 mmol), and CH_3CN (0.5 mL). Then C_4F_9I (34.4 µL, 0.2 mmol, 4.0 equiv.) and allyl heptanoate (38.5 µL, 0.2 mmol, 4.0 equiv.) were added sequentially. The reaction mixture was allowed to stir at 1500 rpm for 20 h at 25 °C. The reaction was quenched with 1 N HCl, extracted with ethyl acetate (EA) three times. The combined organic layers were dried with $MgSO_4$, filtered, and concentrated in vacuo. The crude material was purified by flash chromatography to afford the product.

Radical inhibition experiment

Addition of TEMPO (1.0 equiv.) completely shut down the desired transformation, and TEMPO-C_4F_9 adduct was detected in 79% ^{19}F NMR yield.

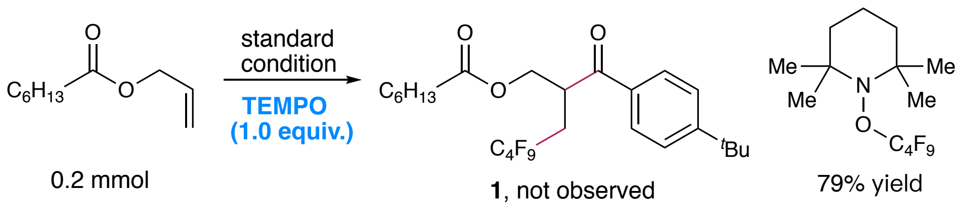

Supplementary Figure 18. Radical inhibition experiment with TEMPO

To a flame-dried 8 mL reaction vial was charged with $NiCl_2$•glyme (2.2 mg, 0.01 mmol, 10 mol%), dtbbpy (5.4 mg, 0.02 mmol, 20 mol%), and Mn (16.5 mg, 0.3 mmol, 3.0 equiv.) and TEMPO (1.0 equiv.). The vial was capped. After evacuated and backfilled nitrogen three times, CH_3CN (1 mL) was added via a syringe, followed by the addition of 4-(*tert*-butyl)benzoyl chloride (29.5 μL, 0.15 mmol, 1.5 equiv.). The reaction mixture was allowed to stir for approximately 1 minute before C_4F_9I (17.2 μL, 0.1 mmol, 1.0 equiv.) and allyl heptanoate (19.2 μL, 0.1 mmol, 1.0 equiv.) were added. The reaction mixture was allowed to stir at 1500 rpm for 20 h at 25 ^{o}C. The reaction mixtures were analyzed by ^{1}H NMR and ^{19}F NMR with internal standards.

Radical clock experiment

Diene **52** underwent radical addition and cyclization, furnishing the expected coupling product **53** in 23% yield as well as alkyl iodide **54** in 49% yield.

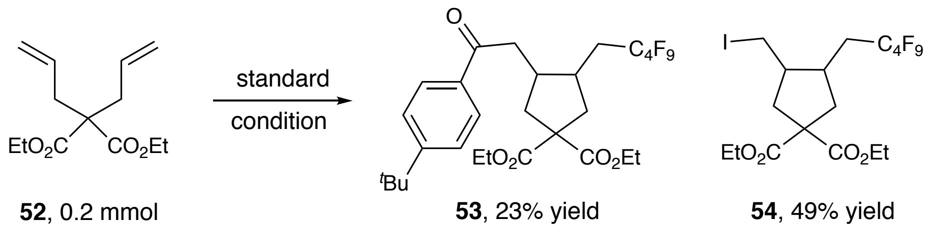

Supplementary Figure 19. Radical clock experiment

According to the general procedure, diethyl 2,2-diallylmalonate (48.3 μL, 0.20 mmol, 1.0 equiv.), C_4F_9I (34.4 μL, 0.20 mmol, 1.0 equiv.), 4-(*tert*-butyl)benzoyl

chloride (58.6 μL, 0.30 mmol, 1.5 equiv.), $NiCl_2$•glyme (4.4 mg, 0.02 mmol, 0.1 equiv.), dtbbpy (10.7 mg, 0.04 mmol, 0.2 equiv.), and Mn power (33.0 mg, 0.3 mmol, 3.0 equiv.) in MeCN (2 mL) were used. Products were obtained by flash chromatography.

Diethyl 3-(2-(4-(tert-butyl)phenyl)-2-oxoethyl)-4-(2,2,3,3,4,4,5,5,5-nonafluoropentyl)cyclopentane-1,1-dicarboxylate (53)

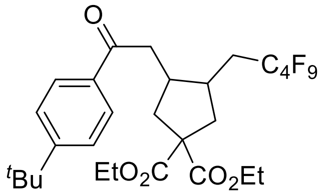

A pale-yellow oil (28.6 mg, 23%); ^{1}H NMR (400 MHz, $CDCl_3$) δ 7.87 (d, J = 8.5 Hz, 2H), 7.48 (d, J = 8.5 Hz, 2H), 4.23 – 4.18 (m, 2H), 4.17 – 4.12 (m, 2H), 3.02 – 2.80 (m, 3H), 2.65 – 2.53 (m, 3H), 2.26 – 2.10 (m, 3H), 2.07 – 1.95 (m, 1H), 1.34 (s, 9H), 1.24 (t, J = 7.1 Hz, 3H), 1.19 (t, J = 7.1 Hz, 3H); ^{19}F NMR (564 MHz, $CDCl_3$) δ -81.03 (t, J = 9.3 Hz), -111.93 – -114.82 (m), -124.42 (d, J = 6.7 Hz), -125.95 (dt, J = 26.7, 13.5 Hz); ^{13}C NMR (100 MHz, $CDCl_3$) δ 198.32, 172.69, 172.16, 157.01, 134.40, 127.96, 125.60, 61.71, 61.66, 58.45, 39.13, 38.94, 38.02, 37.67, 35.10, 34.70, 31.07, 30.54 (t, J = 21.4 Hz), 13.96, 13.89. HRMS (ESI+): calcd for $C_{28}H_{34}F_9O_5^+$ (M+H) 621.2257, found 621.2263.

Diethyl 3-(iodomethyl)-4-(2,2,3,3,4,4,5,5,5-nonafluoropentyl)cyclopentane-1,1-Dicarboxylate (54)

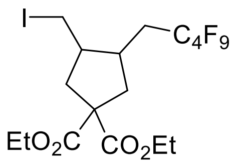

A pale-yellow oil (PE:EA = 15:1) (57.8 mg, 49%); ^{1}H NMR (600 MHz, $CDCl_3$) δ 4.25 – 4.20 (m, 4H), 3.20 – 3.14 (m, 1H), 3.07 (t, J = 9.8 Hz, 1H), 2.66 – 2.48 (m, 4H),

2.36 – 2.31 (m, 1H), 2.31 – 2.18 (m, 2H), 2.12 – 2.04 (m, 1H), 1.30 – 1.26 (m, 6H); ^{19}F NMR (375 MHz, $CDCl_3$) δ -80.71 – -81.25 (m), -112.10 – -115.04 (m), -124.39 (d, J = 8.1 Hz), -125.77 – -126.11 (m); ^{13}C NMR (150 MHz, $CDCl_3$) δ 172.27, 171.95, 61.91, 61.82, 58.26, 45.36, 39.70, 38.33, 35.33, 29.58(t, J = 21.5 Hz), 13.99, 13.97, 5.52. HRMS (ESI+): calcd for $C_{17}H_{21}F_9IO_5^+$ (M+H) 587.0335, found 587.0040.

Evaluation of the generation of C_4F_9 radical in the absence of Ni

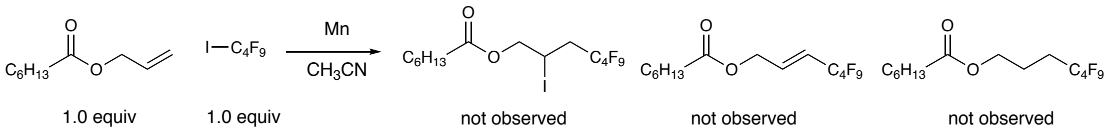

Supplementary Figure 20. Control experiment without nickel catalyst.

In the absence of nickel catalyst, alkene remained untouched. Mn(0) couldn't promote the generation of C_4F_9 radical. We assumed that Ni(0) is responsible for the generation of C_4F_9 radical in this transformation.

Evaluation of the chelating group effect

Six-membered and seven-membered chelating substrates gave the optimal yields. For the case of the eight-membered chelating substrate, the formation of C_4F_9-alkene byproduct (36% isolated yield) as well as C_4F_9-alkane byproduct (detected by GC-MS) has also been observed.

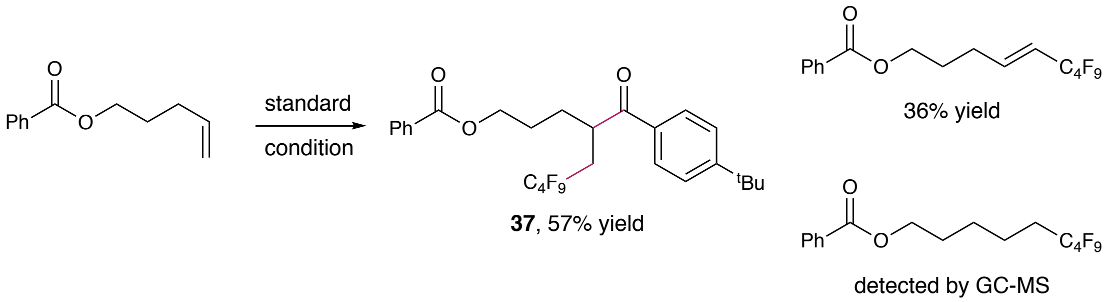

Supplementary Figure 21. Reaction of pent-4-en-1-yl benzoate

For the substrate without directing group, only a trace amount of the desired product was observed, major byproducts were C_4F_9-alkene and C_4F_9-alkane (detected by GC-MS).

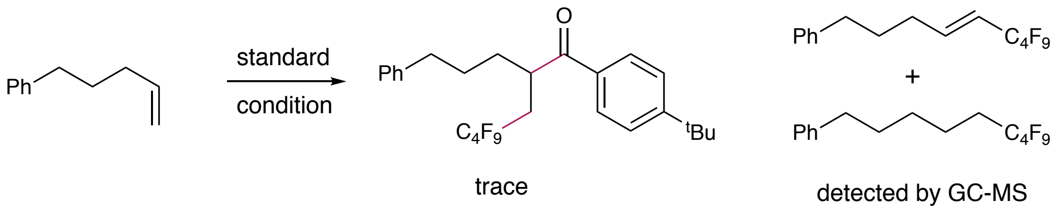

Supplementary Figure 22. Reaction of pent-4-en-1-ylbenzene

These results indicate that the chelating effect does not promote the generation of C_4F_9 radical. We envision that the chelating effect would facilitate the capture of alkyl radical to Ni complex.

On the basis of these experimental results, we reasoned that pathway **I** proceeding via oxidative addition of Ni(0) species with acyl chloride could be operative.

Discussion on another possible pathway:

Pathway III:

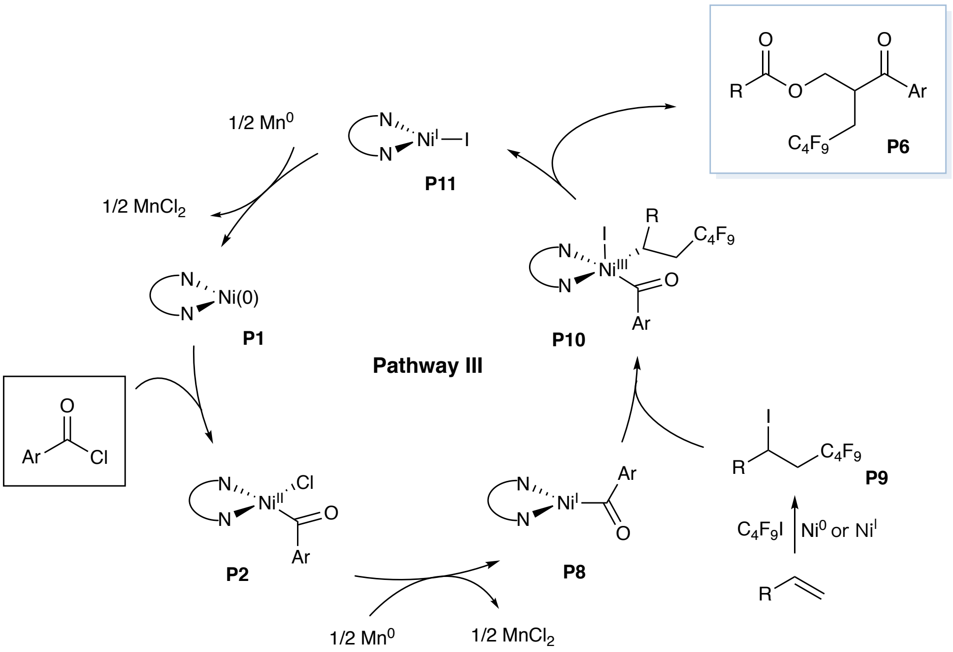

Supplementary Figure 23. Plausible mechanisms of pathway III

Similar to the previous Ni-catalyzed reductive cross-coupling of acyl chlorides and alkyl iodides works developed by the Gong[4] and Reisman[5] group, a third alternative involves a sequential oxidative addition pathway. Oxidative addition of Ni(0) with acyl chloride would afford Ni^{II} complex **P2**, which would be reduced by Mn dust to yield Ni^{I} species **P8**. At the same time, ATRA reaction of alkene with C_4F_9I mediated by Ni(0) would generate alkyl iodide **P9**. Subsequent oxidative addition of Ni^{I} **P8** with alkyl iodide would generate Ni^{III} complex **P10**, which would undergo facile reductive elimination to furnish the final product as well as Ni^{I} intermediate **P11**. Single-electron reduction of Ni^{I} **P11** by Mn dust would regenerate Ni(0) species **PI** and complete the catalytic cycle. Based on Gong and Reisman's work, alkene substrates with or without directing groups should afford similar results, which were not true in our cases. In addition, GC-MS analysis of the crude reaction mixtures (standard alkene substrates bearing directing groups) indicated that no formation of alkyl iodides in our system. We believe that pathway **II** involving radical species could give a better elucidation for our experimental results.

Spectral Data.

^{1}H NMR (600 MHz, $CDCl_3$)

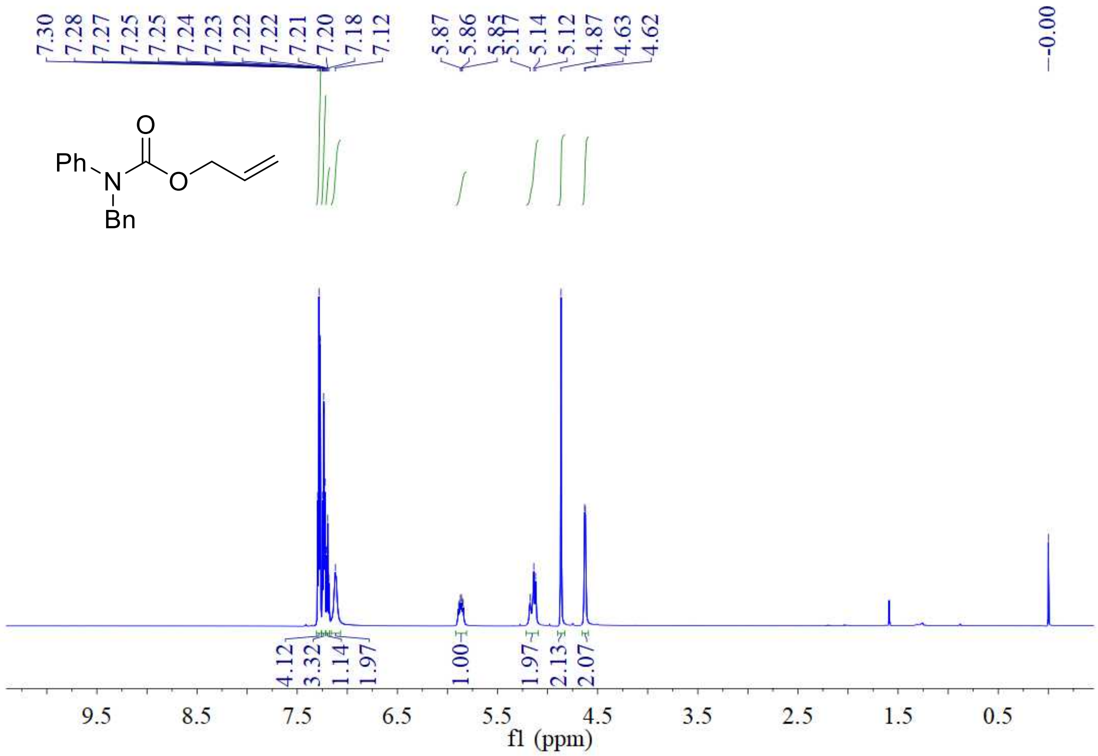

^{13}C NMR (150 MHz, $CDCl_3$)

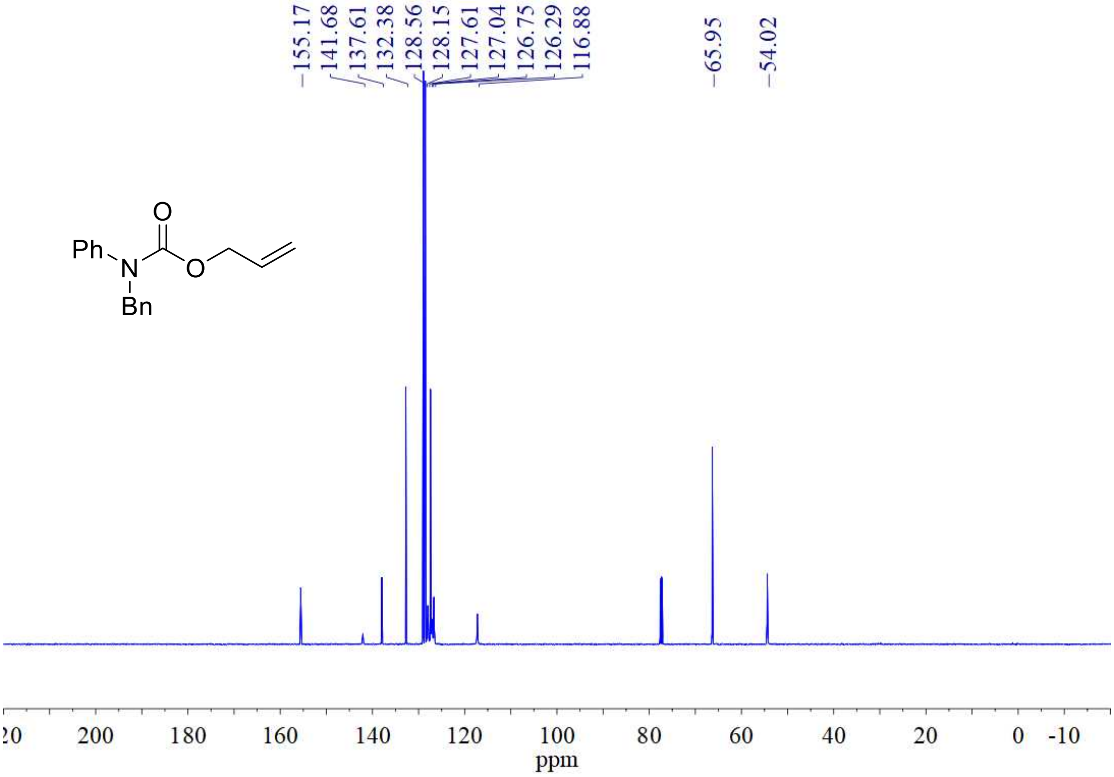

Supplementary Figure 24: NMR Spectra of Allyl benzyl(phenyl)carbamate (S1)

^{1}H NMR (600 MHz, $CDCl_3$)

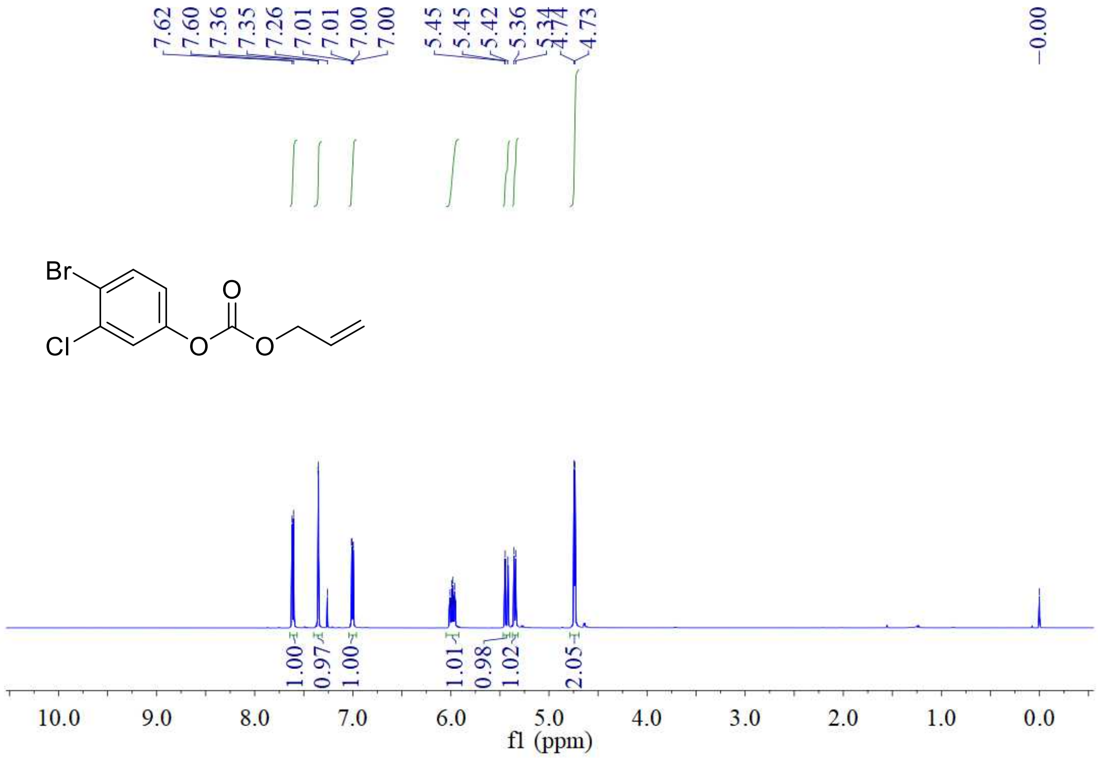

^{13}C NMR (150 MHz, $CDCl_3$)

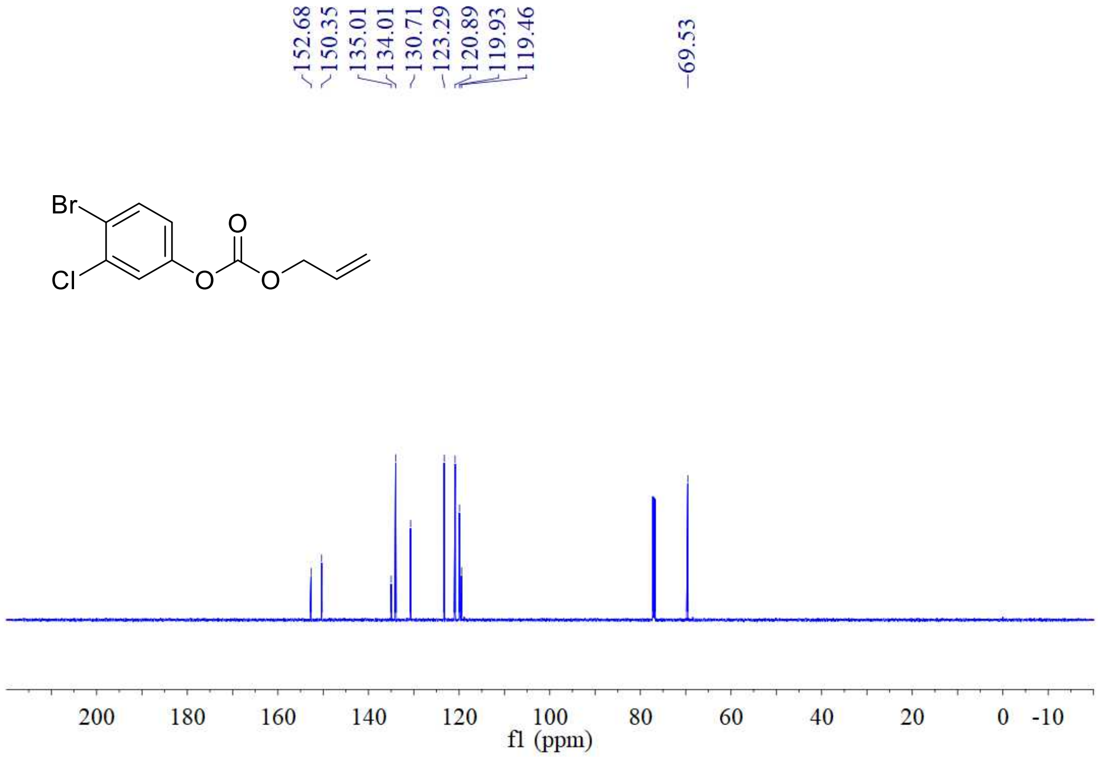

Supplementary Figure 25: NMR Spectra of Allyl (4-bromo-3-chlorophenyl) carbonate (S2)

^{1}H NMR (600 MHz, $CDCl_3$)

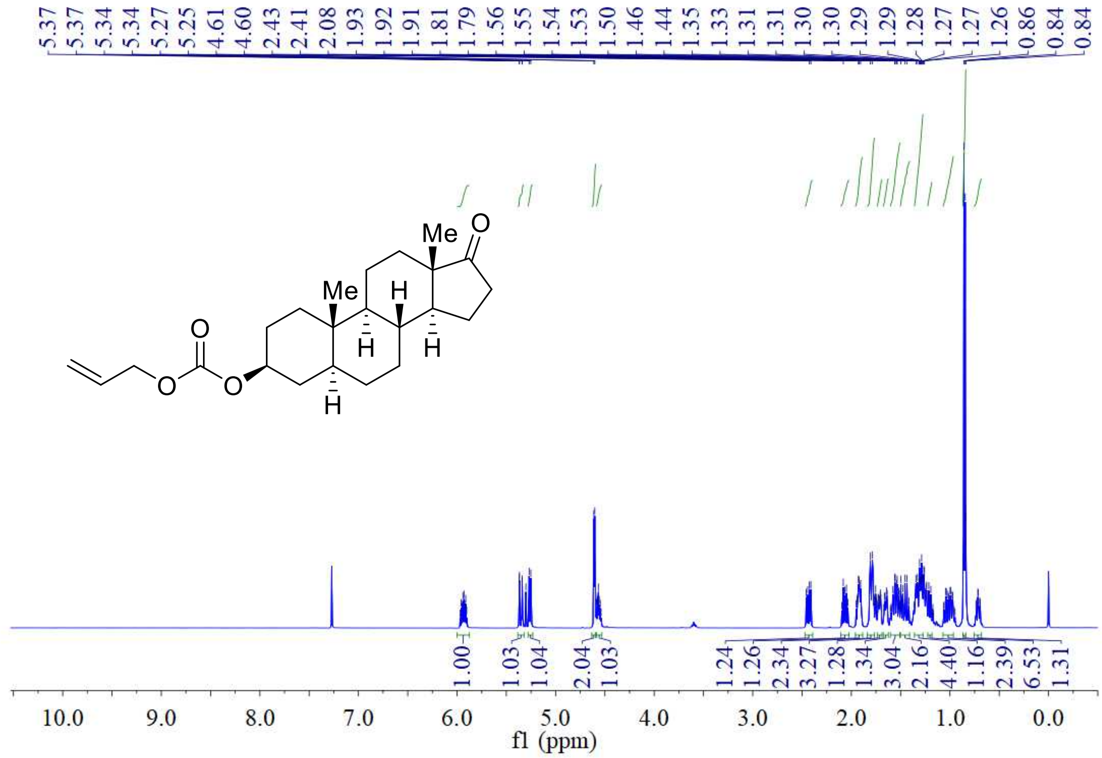

^{13}C NMR (150 MHz, $CDCl_3$)

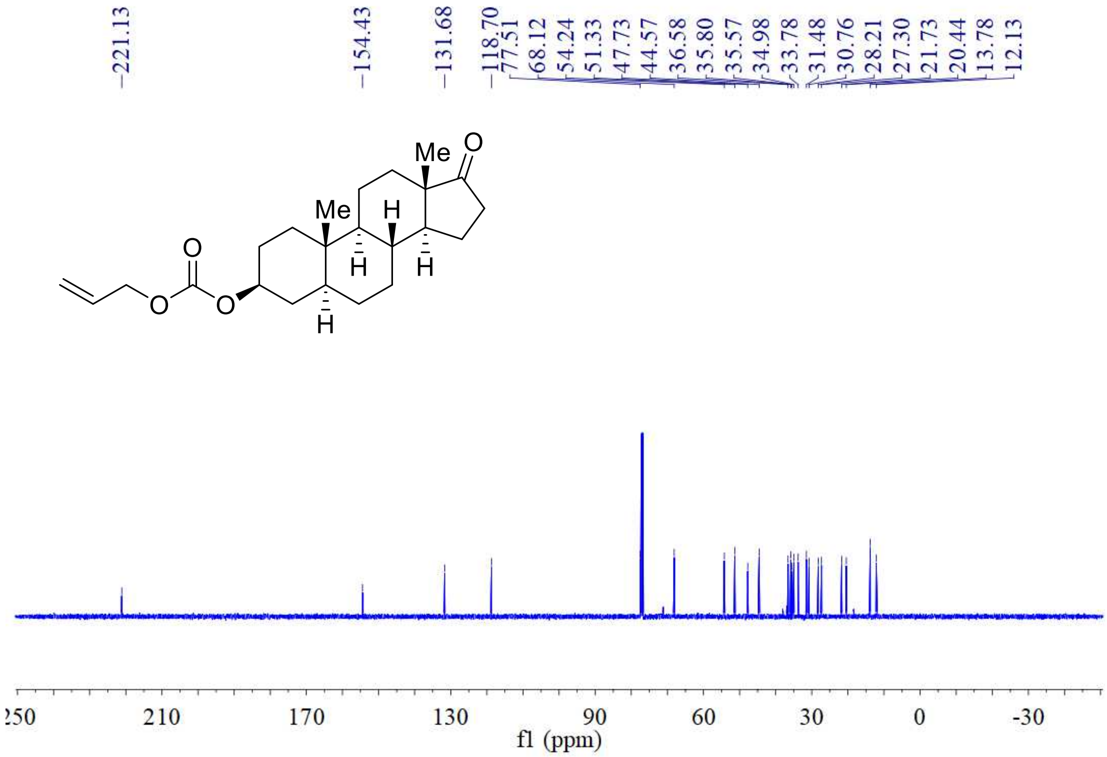

Supplementary Figure 26: NMR Spectra of Allyl ((3R,8R,9R,10R,13R,14R)-10,13-dimethyl-17-oxohexadecahydro-1H-cyclopenta[a]phenanthren-3-yl) carbonate (S3)

^{1}H NMR (600 MHz, $CDCl_3$)

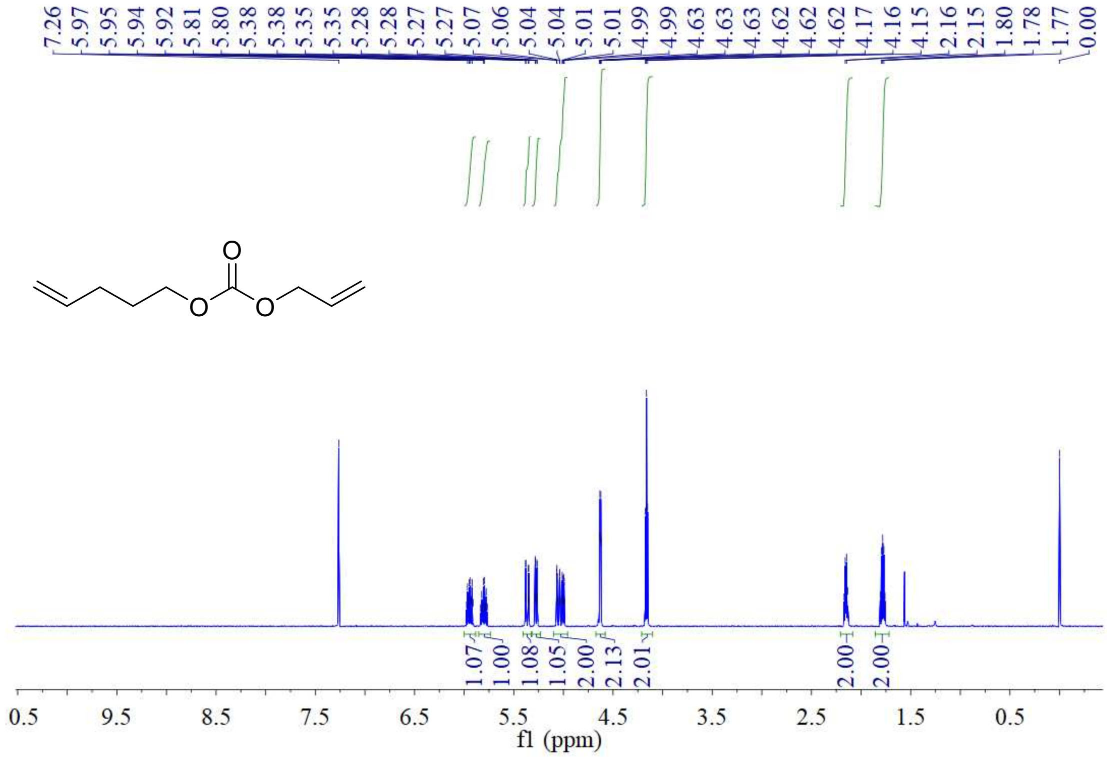

^{13}C NMR (150 MHz, $CDCl_3$)

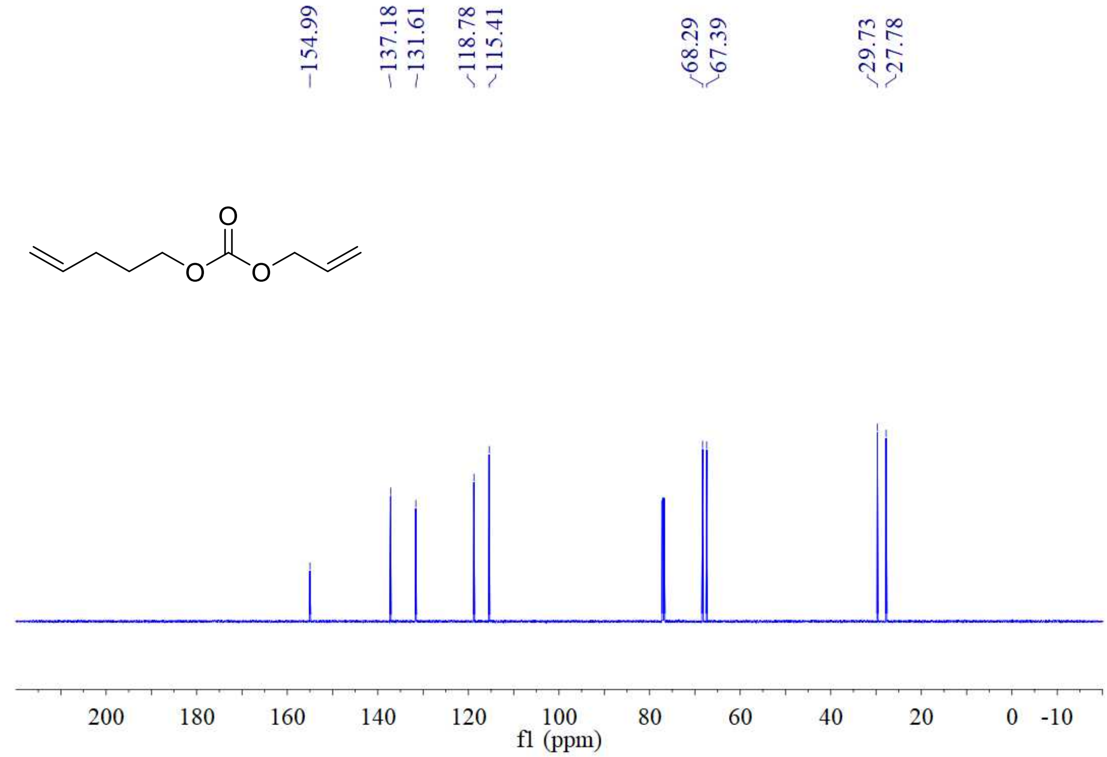

Supplementary Figure 27: NMR Spectra of Allyl pent-4-en-1-yl carbonate (S4)

^{1}H NMR (600 MHz, $CDCl_3$)

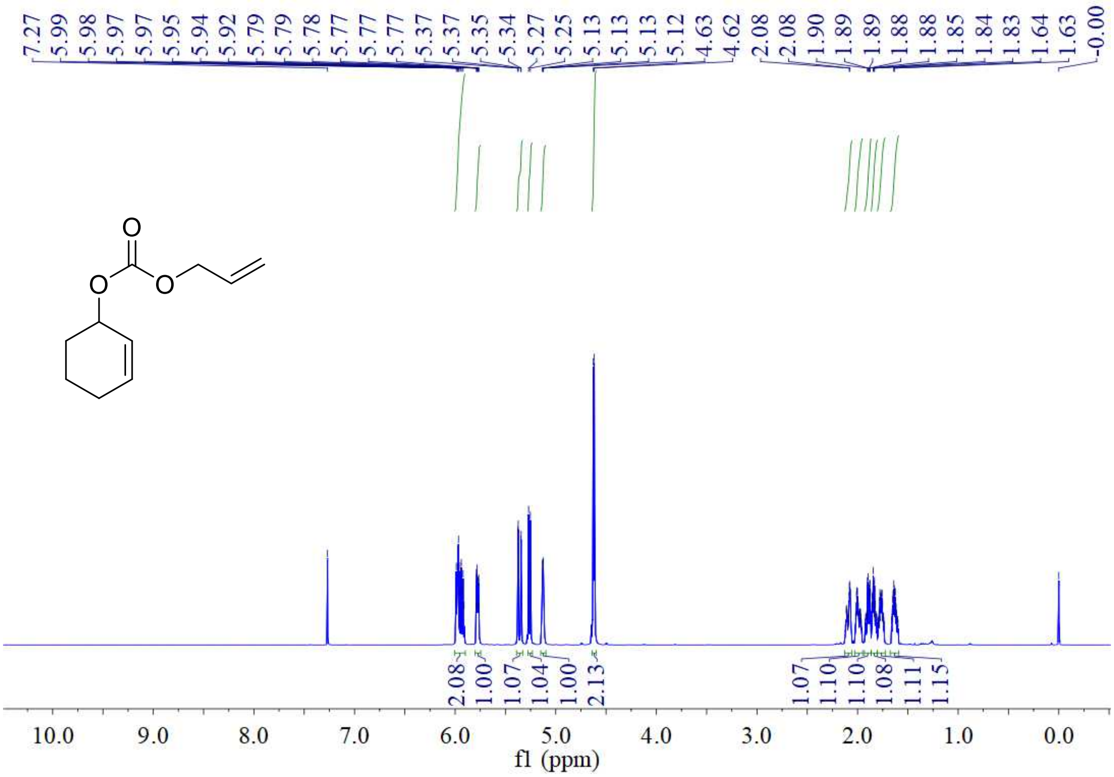

^{13}C NMR (150 MHz, $CDCl_3$)

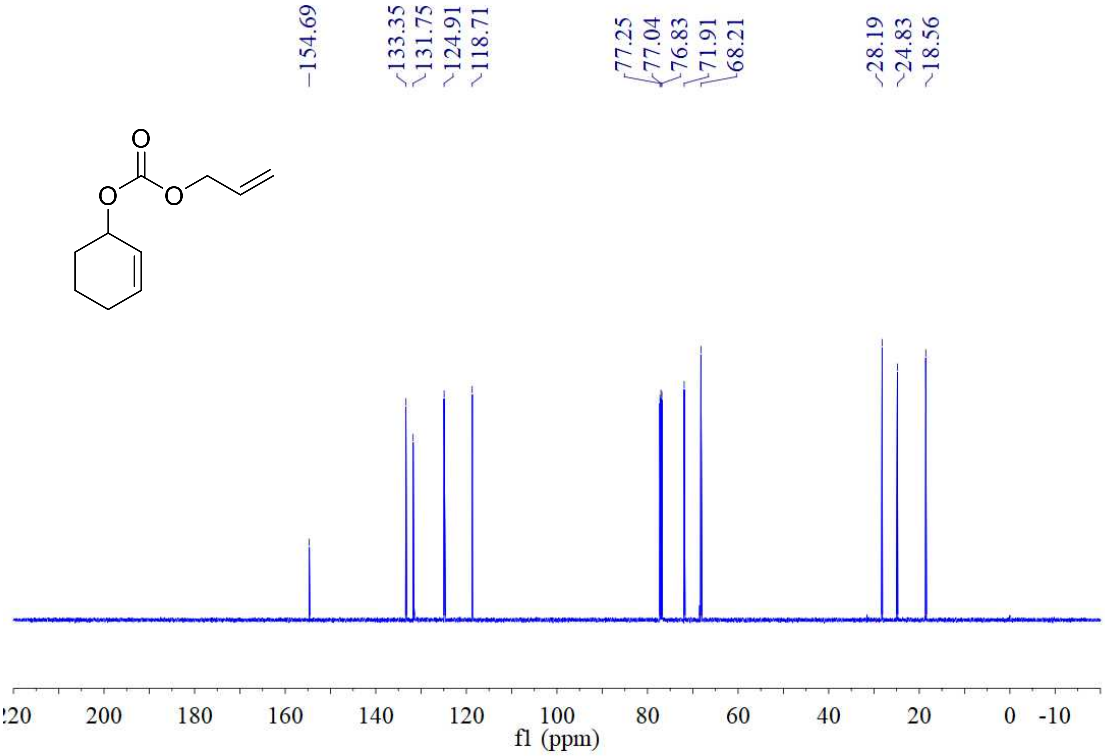

Supplementary Figure 28: NMR Spectra of Allyl cyclohex-2-en-1-yl carbonate (S5)

[1] H NMR (600 MHz, $CDCl_3$)

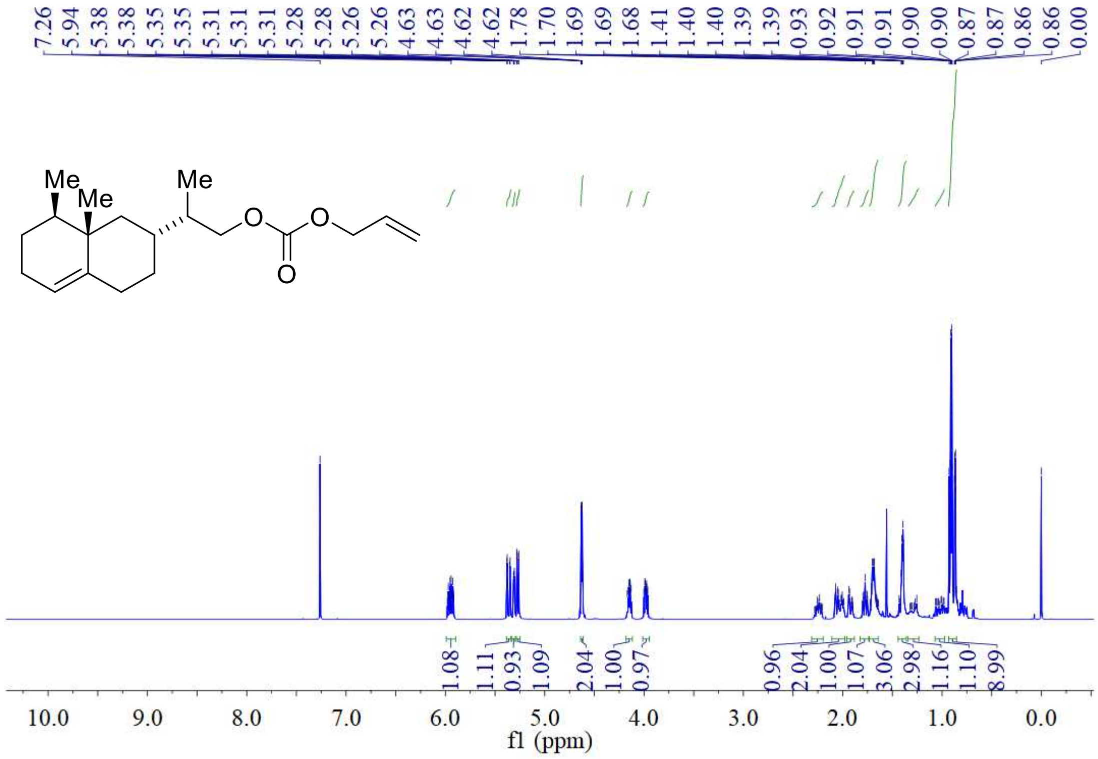

[13] C NMR (150 MHz, $CDCl_3$)

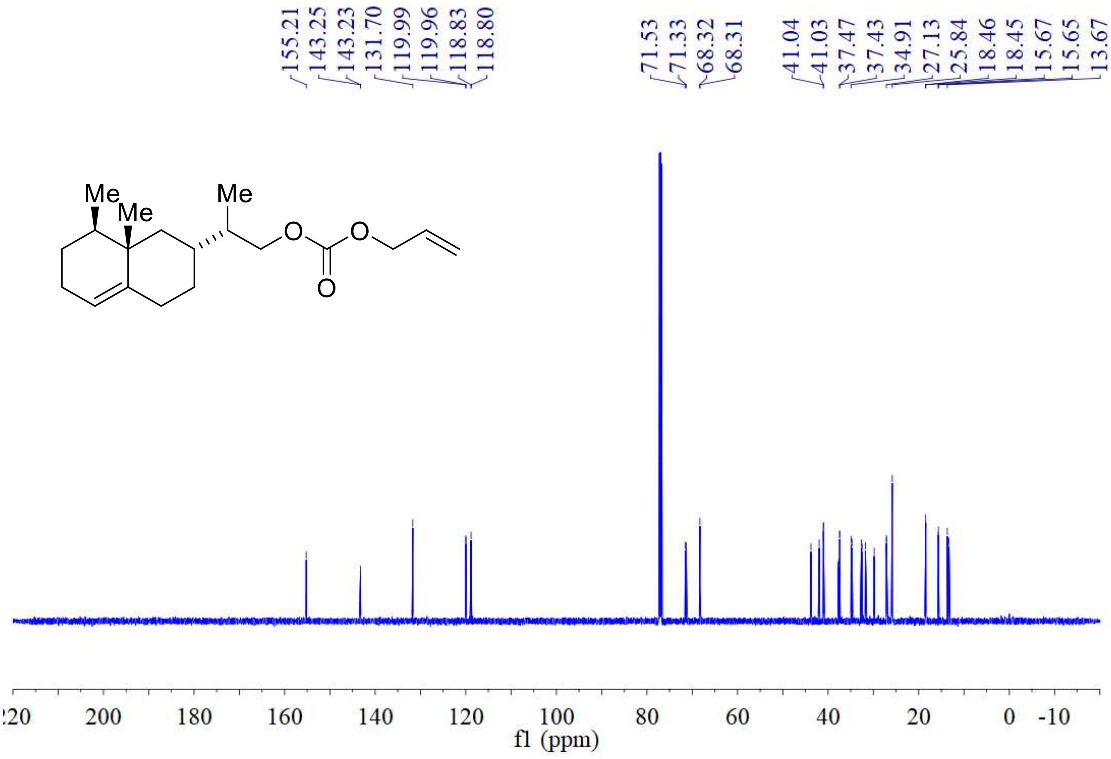

Supplementary Figure 29: NMR Spectra of Allyl ((S)-2-((2R,8R,8aS)-8,8a-dimethyl-1,2,3,4,6,7,8,8a-octahydronaphthalen-2-yl)propyl) carbonate (S6)

^{1}H NMR (600 MHz, $CDCl_3$)

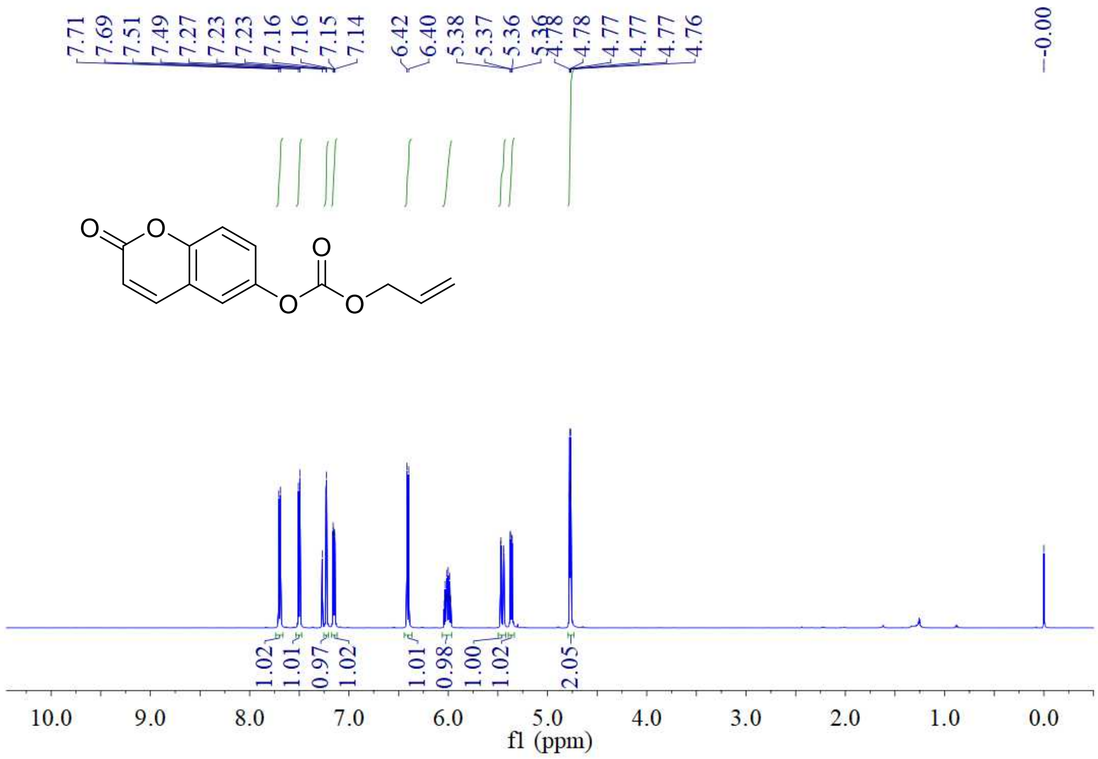

^{13}C NMR (150 MHz, $CDCl_3$)

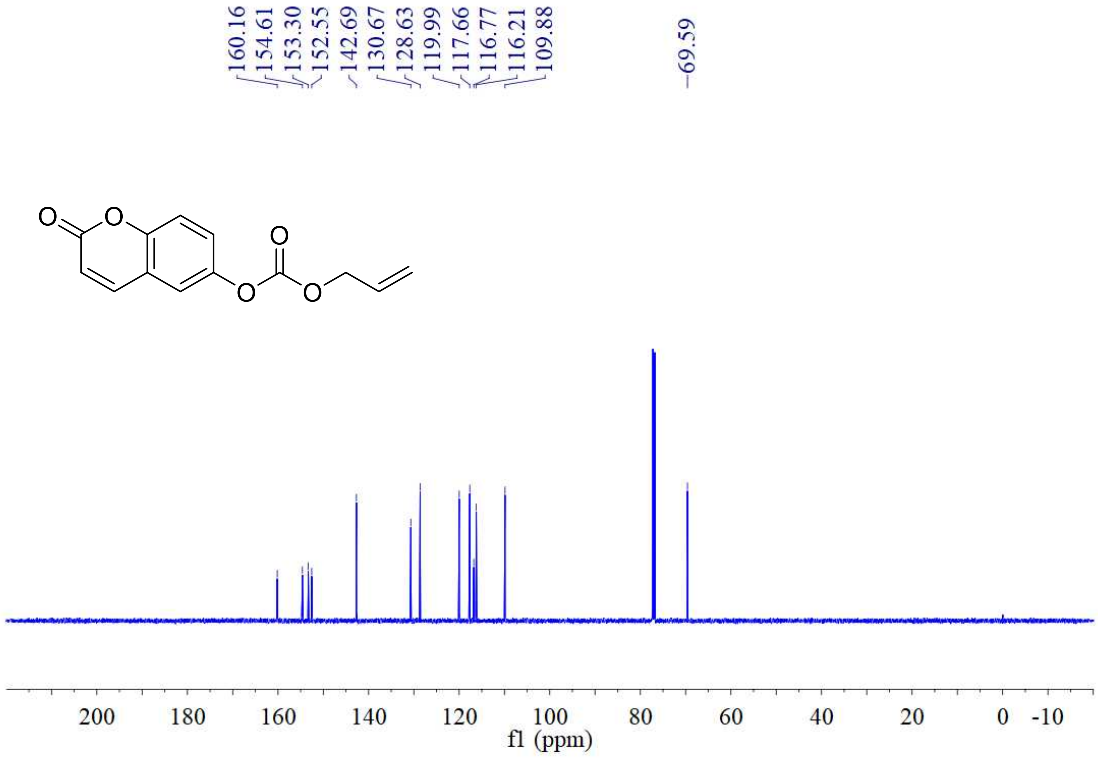

Supplementary Figure 30: NMR Spectra of Allyl (2-oxo-2H-chromen-6-yl) carbonate (S7)

^{1}H NMR (600 MHz, $CDCl_3$)

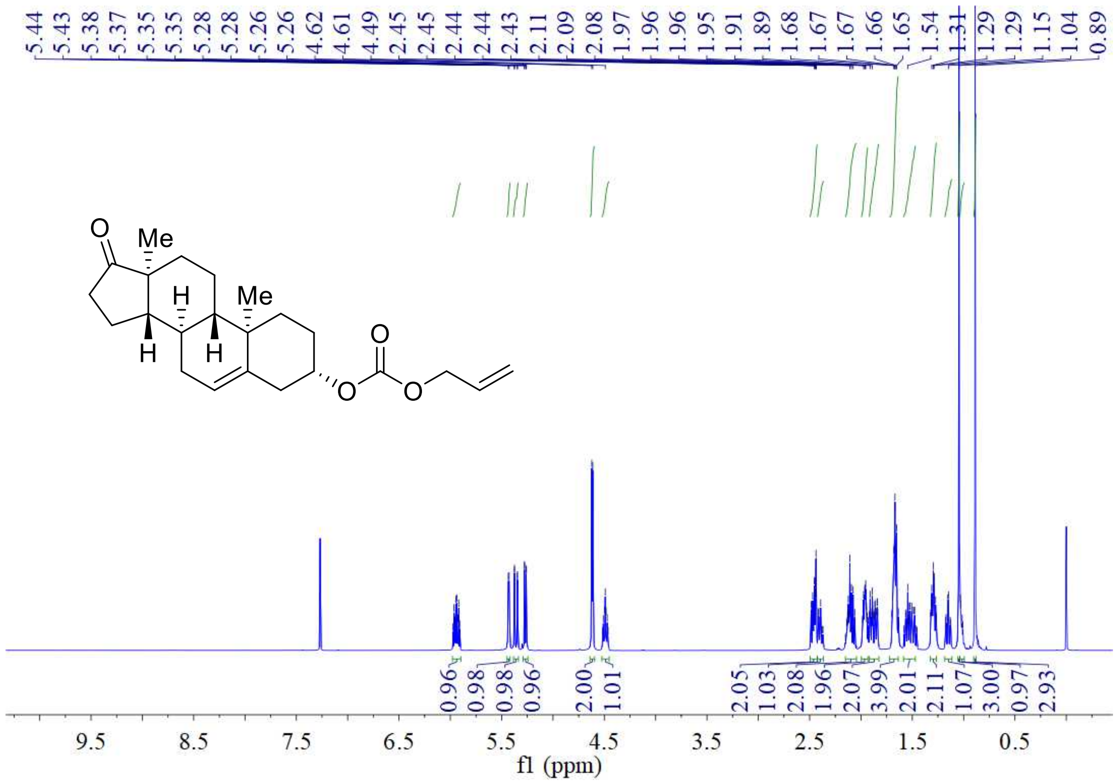

^{13}C NMR (150 MHz, $CDCl_3$)

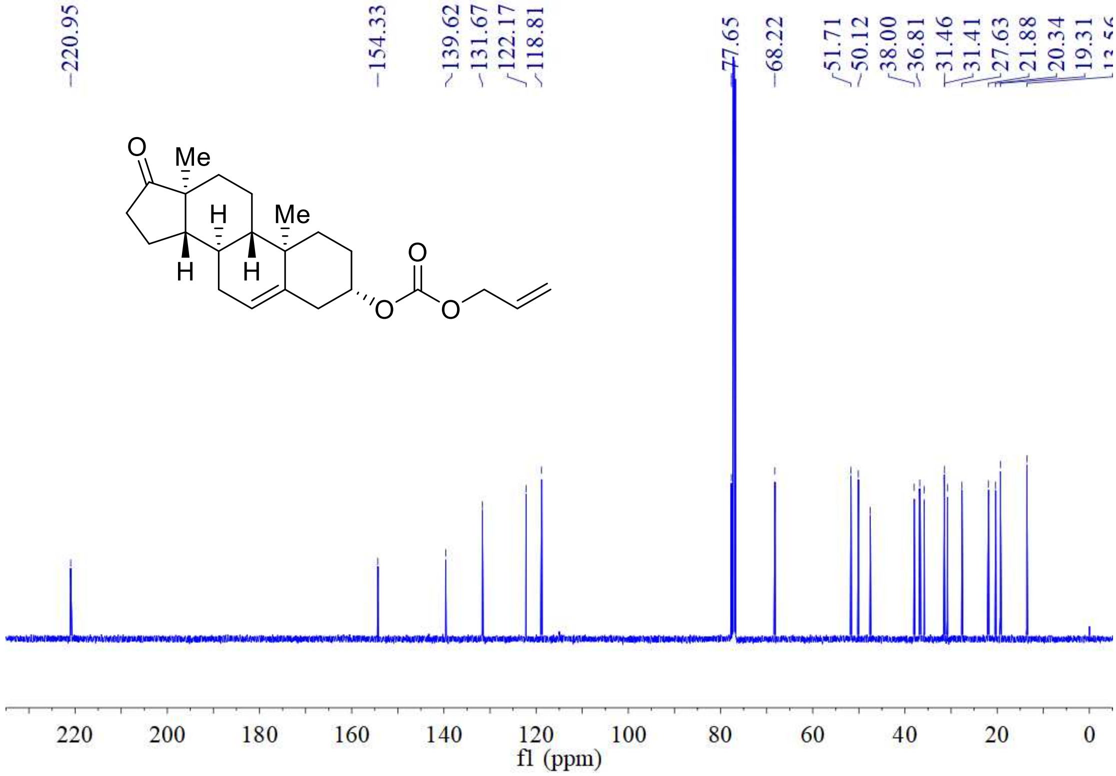

Supplementary Figure 31: NMR Spectra of Allyl ((3S,8R,9S,10R,13S,14S)-10,13-dimethyl-17-oxo-2,3,4,7,8,9,10,11,12,13,14,15,16,17-tetradecahydro-1H-cyclopenta[a]phenanthren-3-yl) carbonate (S8)

^{1}H NMR (400 MHz, $CDCl_3$)

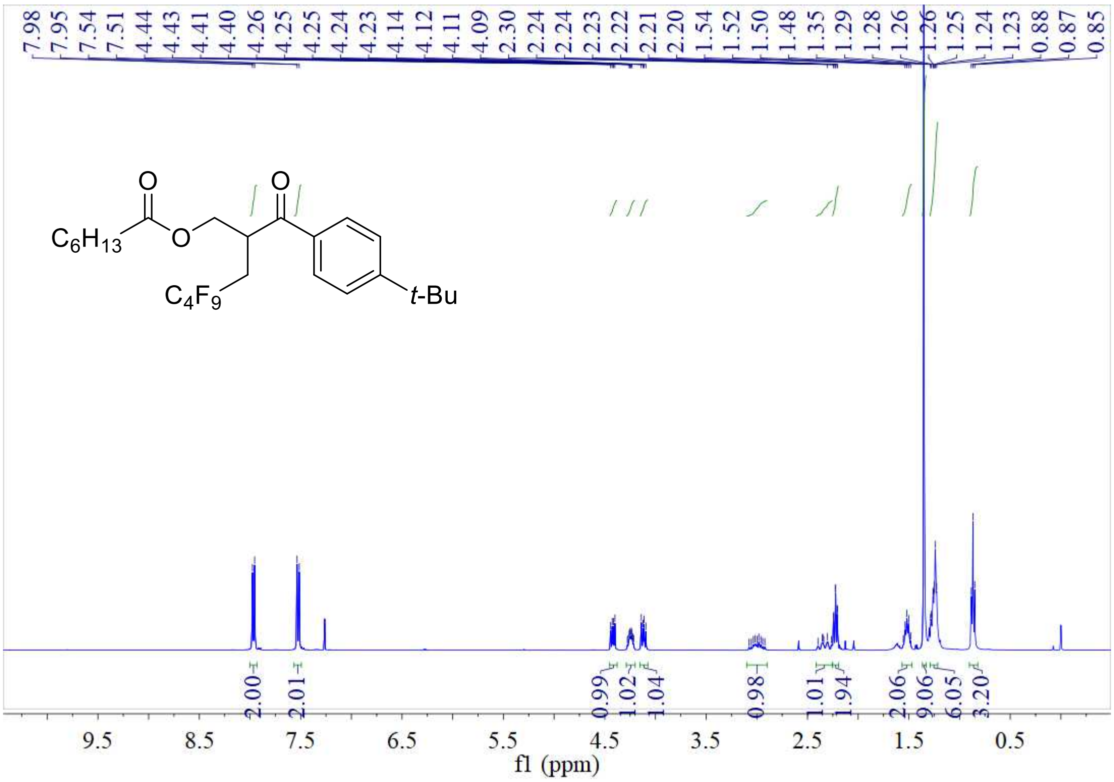

^{19}F NMR (376 MHz, $CDCl_3$)

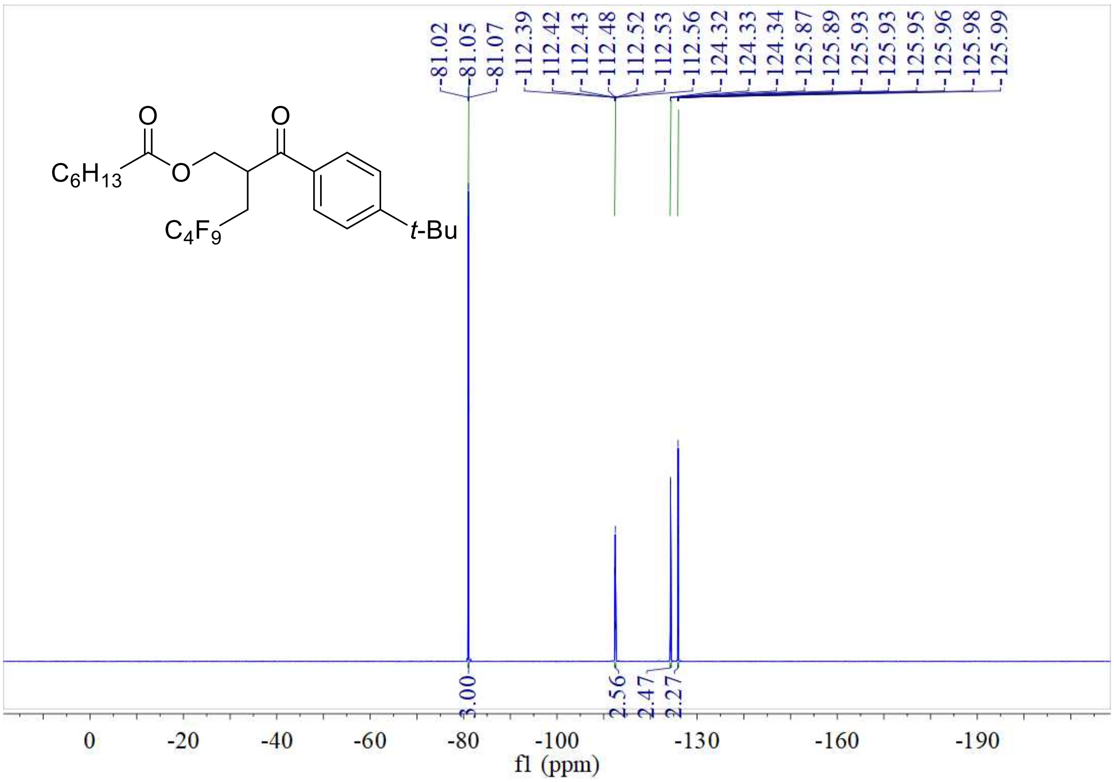

^{13}C NMR (100 MHz, $CDCl_3$)

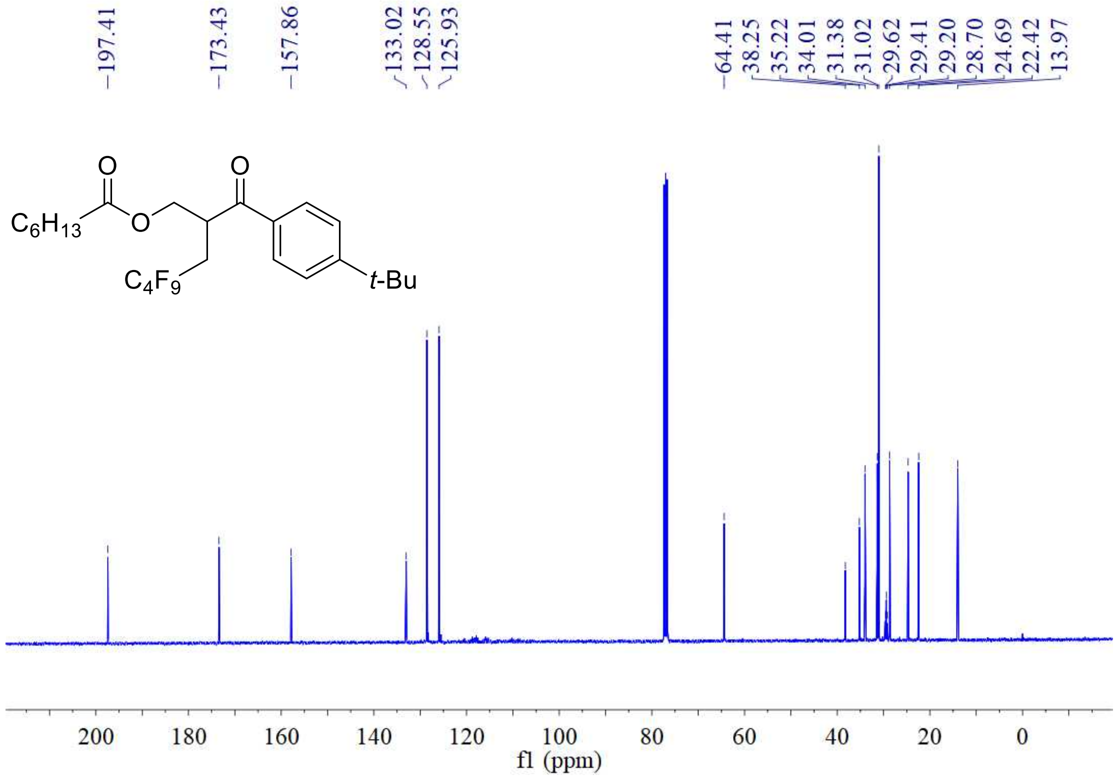

Supplementary Figure 32: NMR Spectra of 2-(4-(*tert*-Butyl)benzoyl)-4,4,5,5,6,6,7,7,7-nonafluoroheptyl heptanoate (1)

^{1}H NMR (600 MHz, $CDCl_3$)

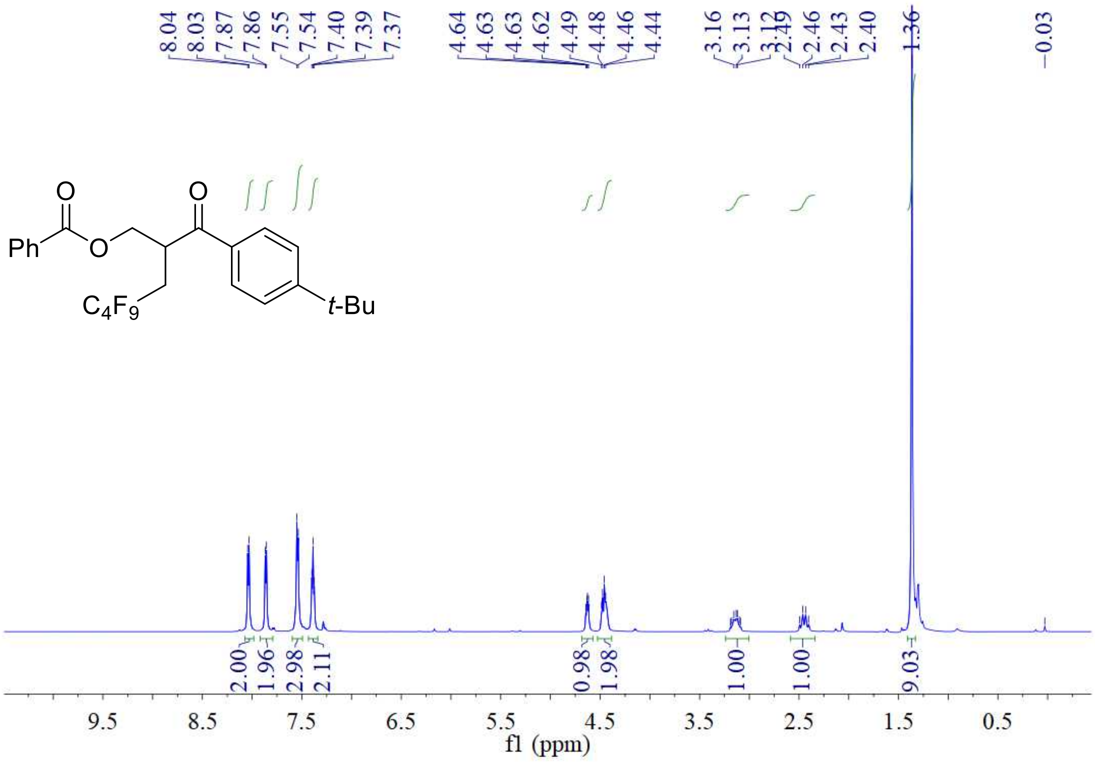

^{19}F NMR (564 MHz, $CDCl_3$)

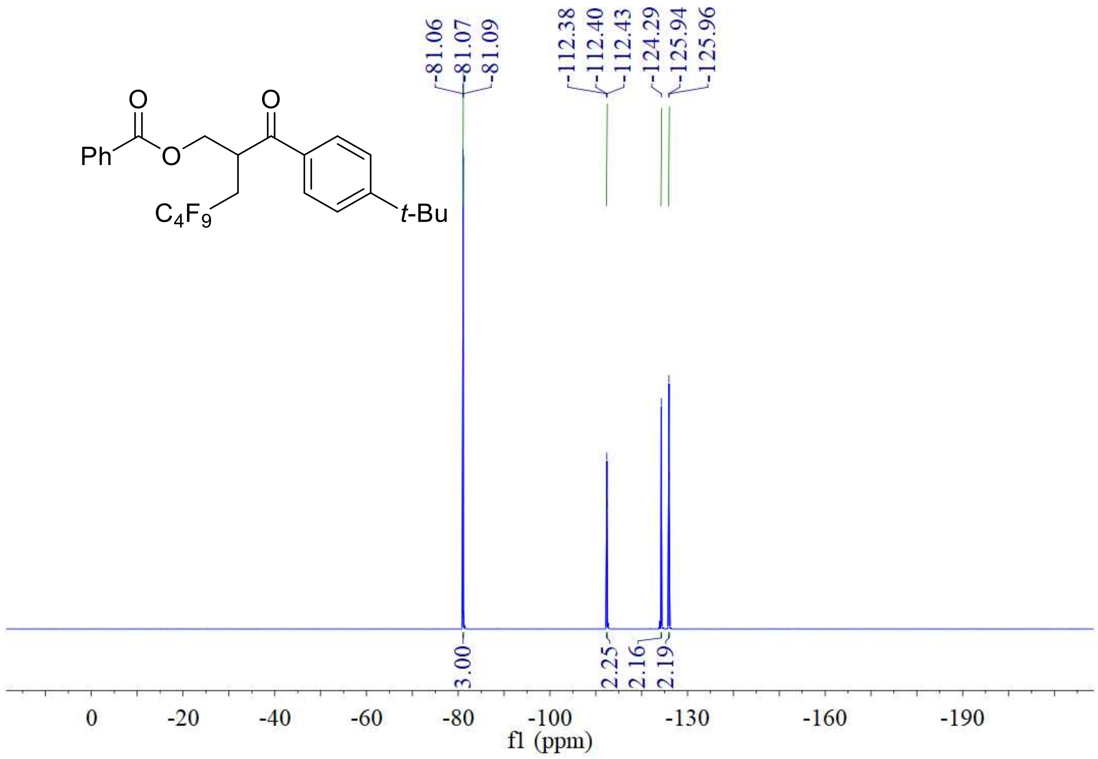

^{13}C NMR (150 MHz, $CDCl_3$)

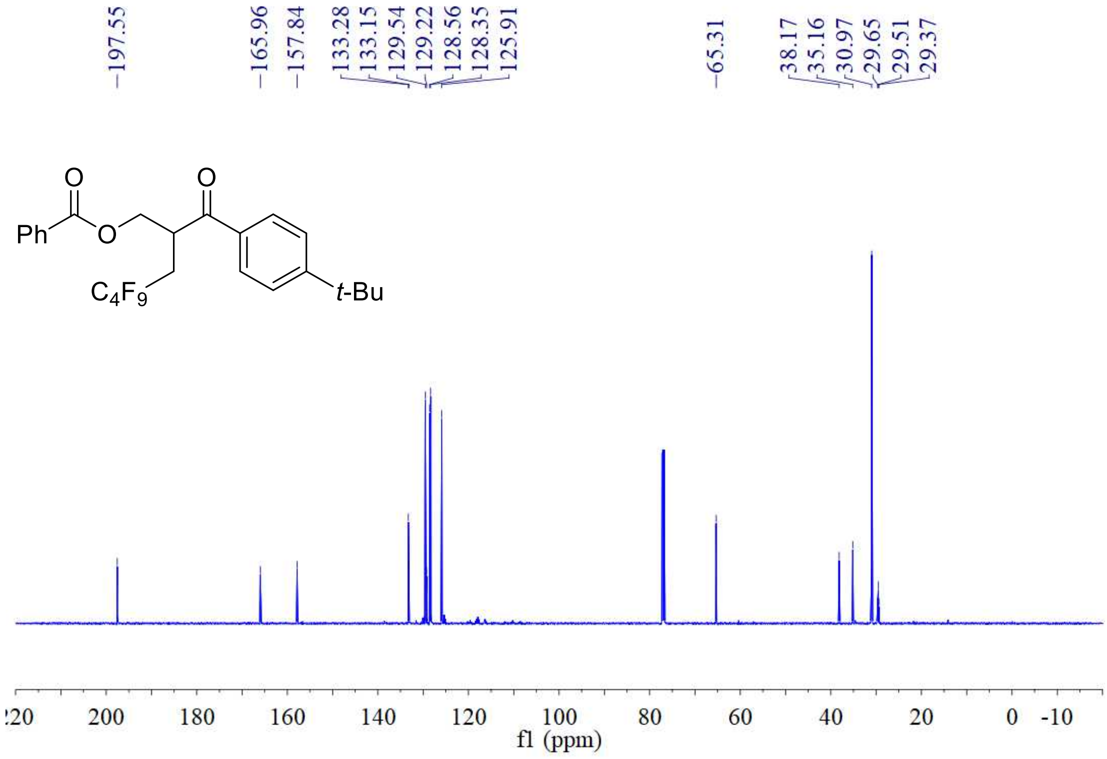

Supplementary Figure 33: NMR Spectra of 2-(4-(*tert*-Butyl)benzoyl)-4,4,5,5,6,6,7,7,7-nonafluoroheptyl benzoate (2)

^{1}H NMR (600 MHz, $CDCl_3$)

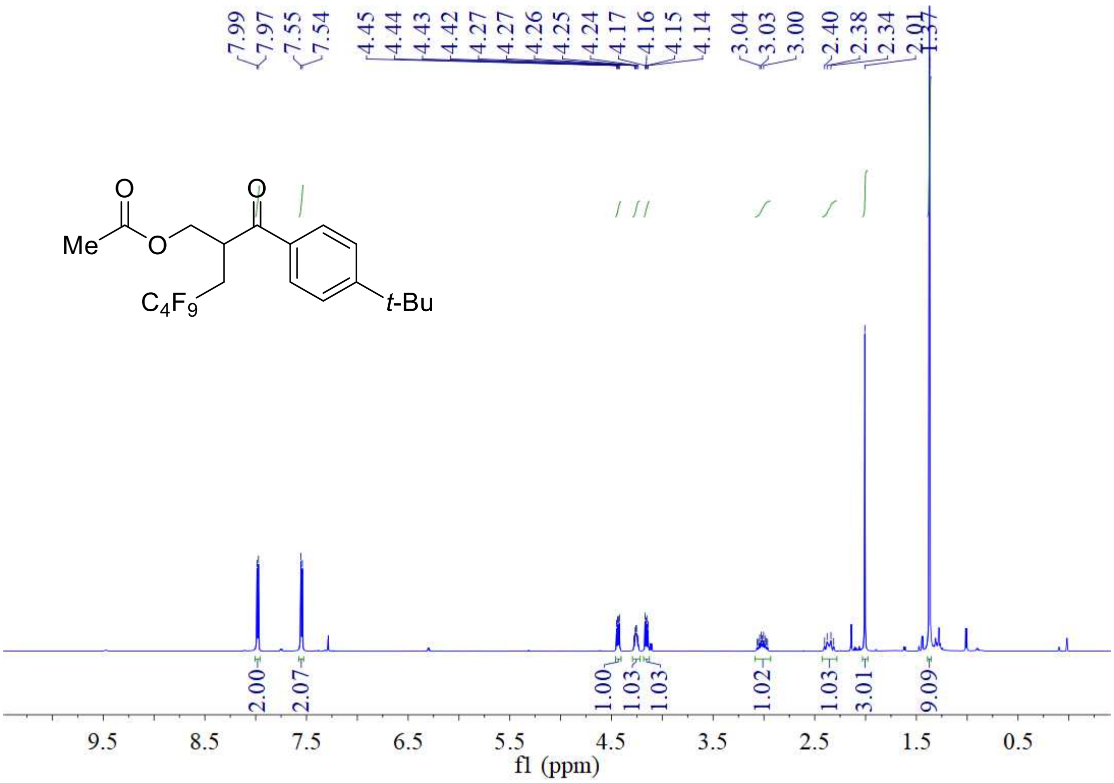

^{19}F NMR (376 MHz, $CDCl_3$)

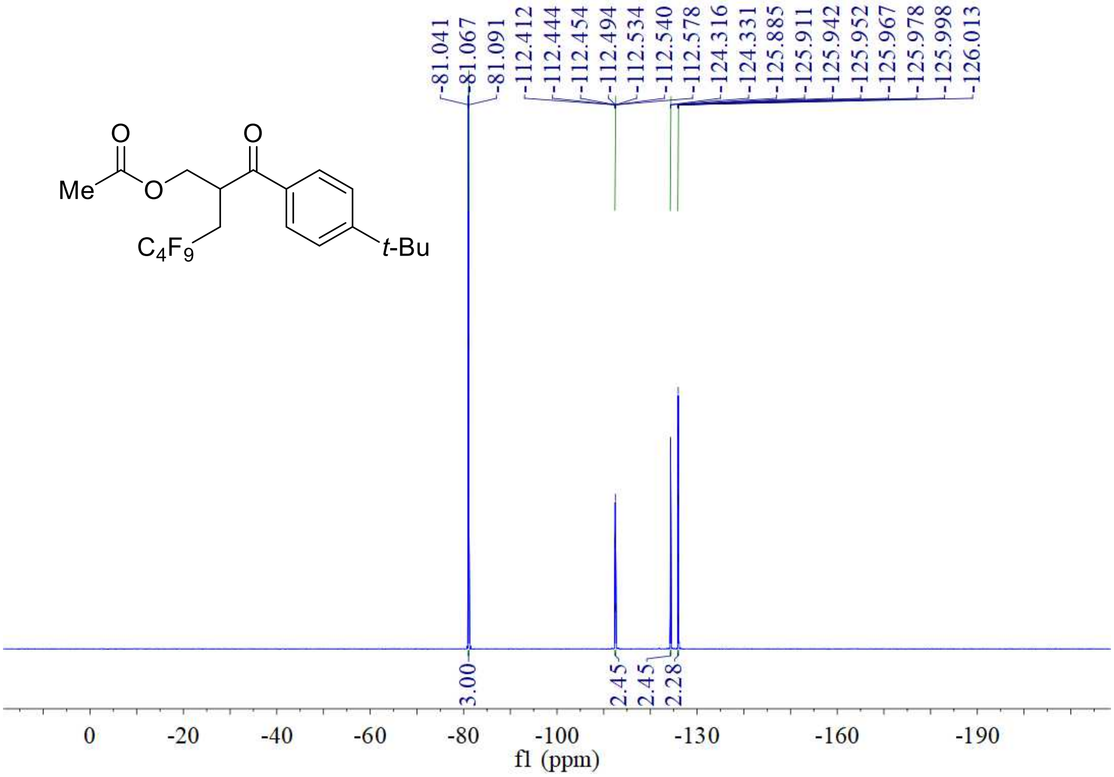

^{13}C NMR (150 MHz, $CDCl_3$)

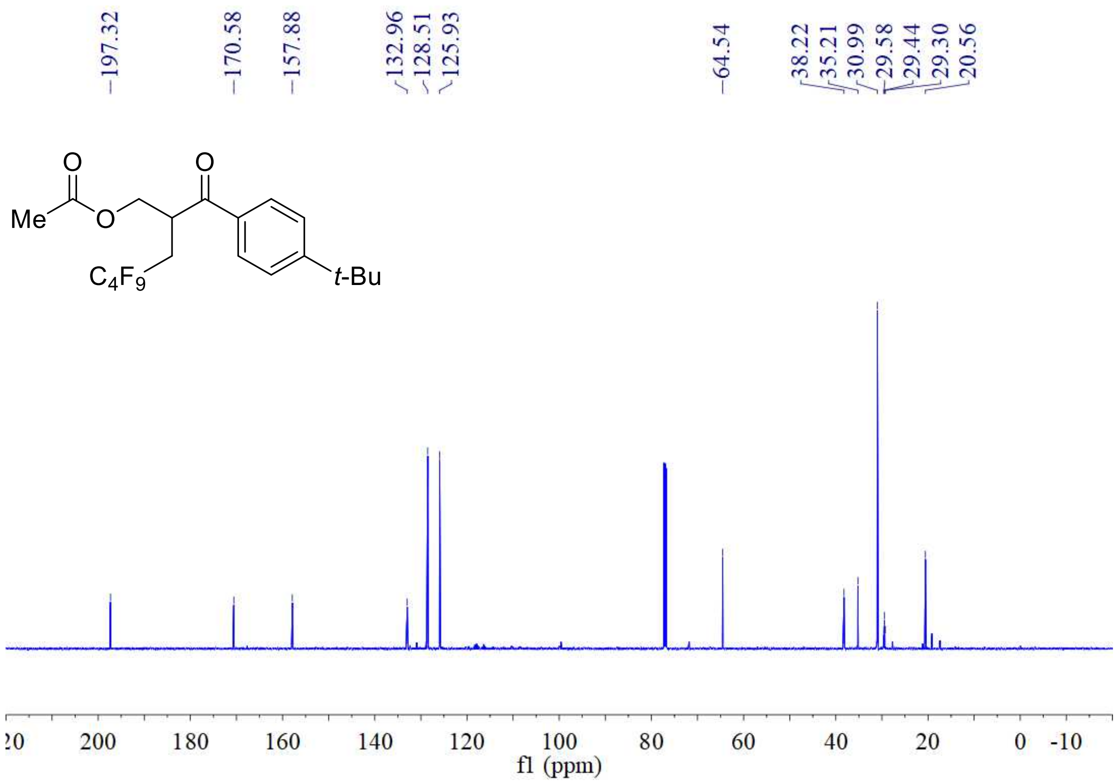

Supplementary Figure 34: NMR Spectra of 2-(4-(*tert*-Butyl)benzoyl)-4,4,5,5,6,6,7,7,7-nonafluoroheptyl acetate (3)

^{1}H NMR (400 MHz, $CDCl_3$)

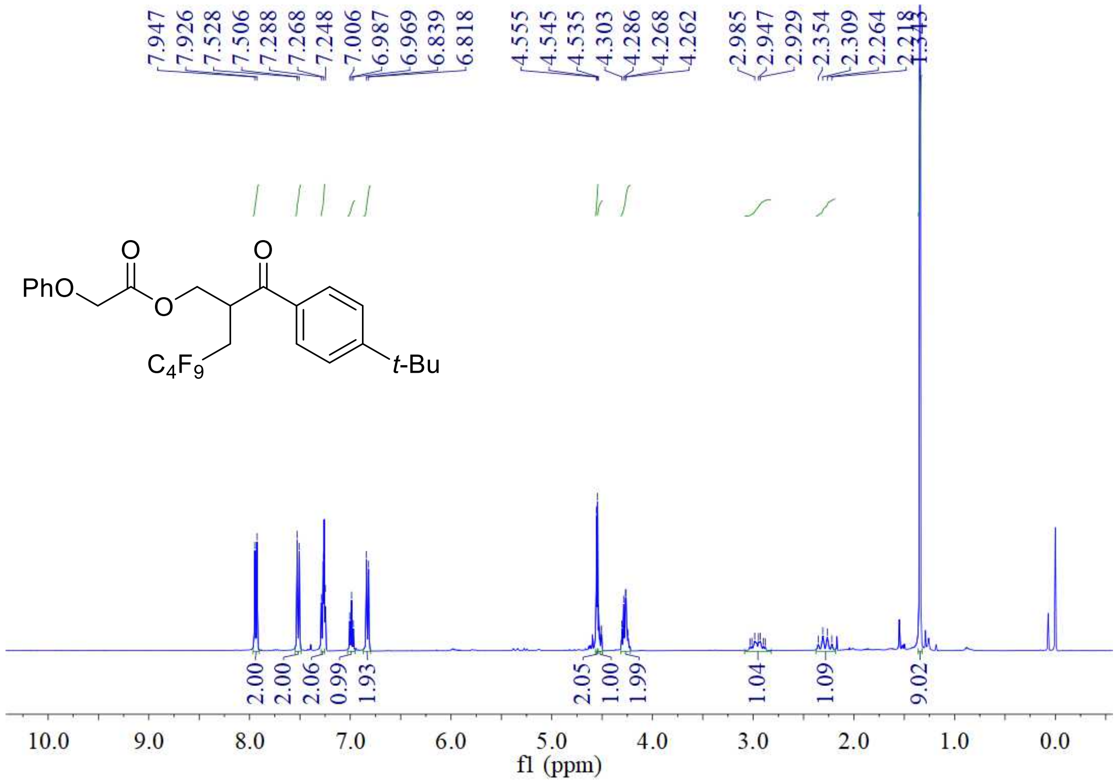

^{19}F NMR (376 MHz, $CDCl_3$)

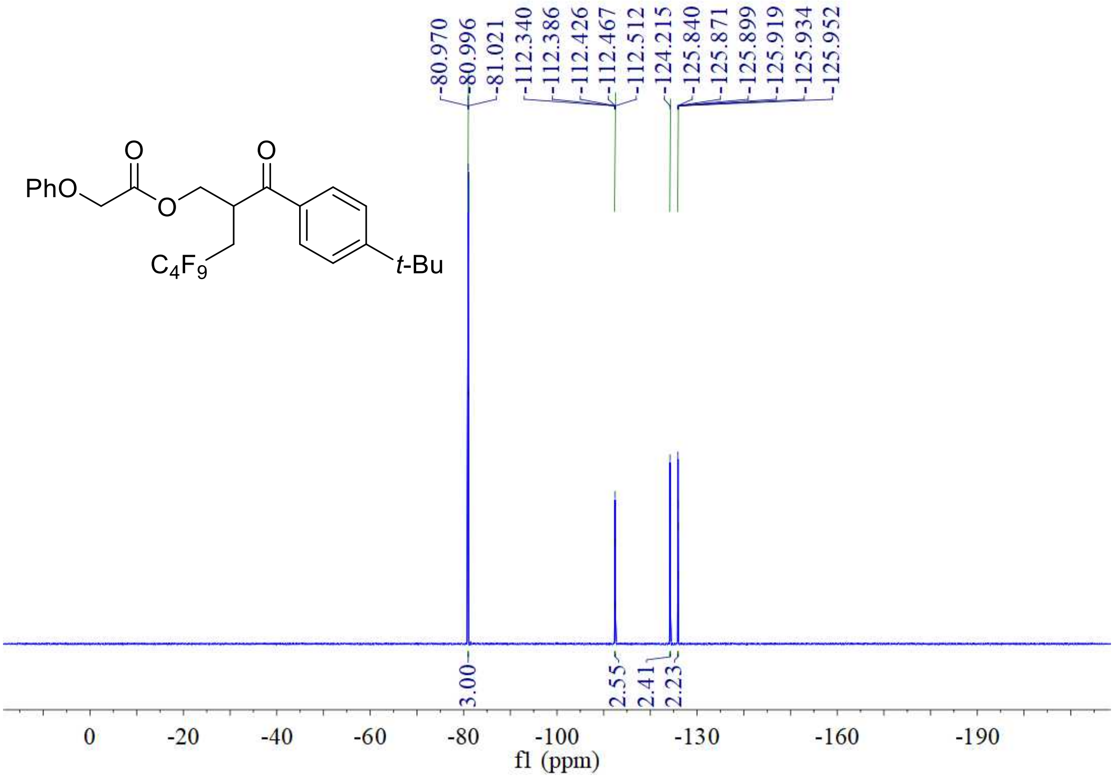

^{13}C NMR (101 MHz, $CDCl_3$)

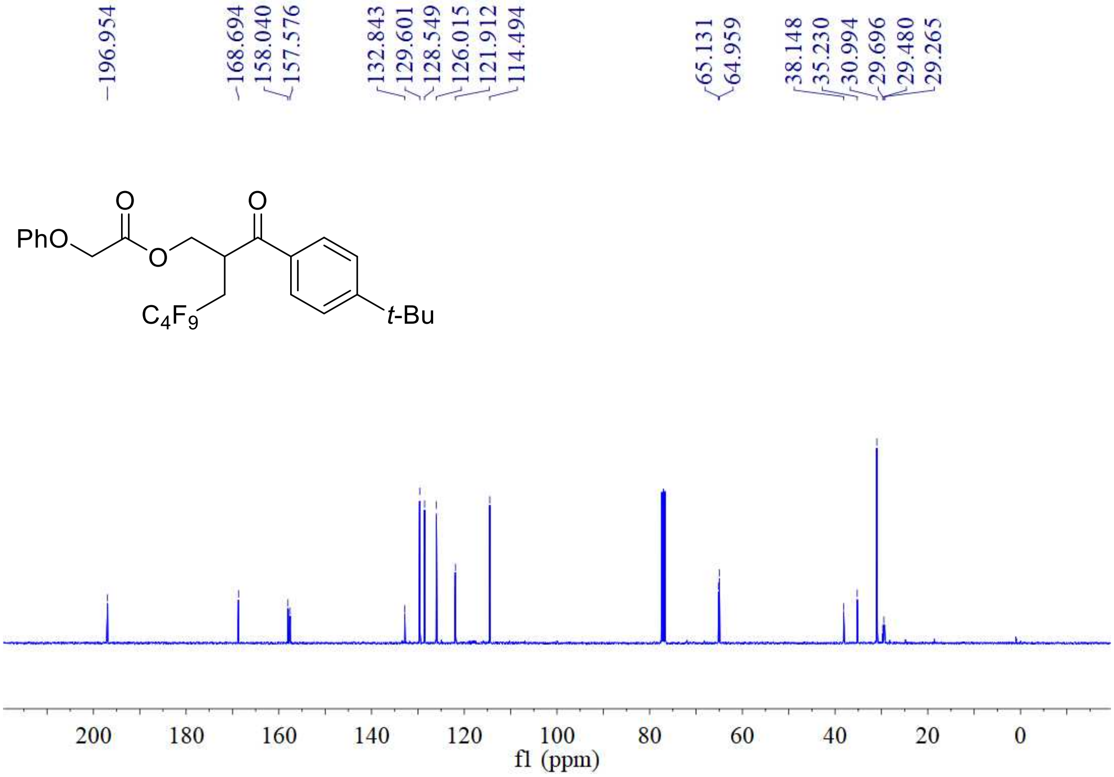

Supplementary Figure 35: NMR Spectra of 2-(4-(*tert*-Butyl)benzoyl)-4,4,5,5,6,6,7,7,7-nonafluoroheptyl 2-phenoxyacetate (4)

^{1}H NMR (600 MHz, $CDCl_3$)

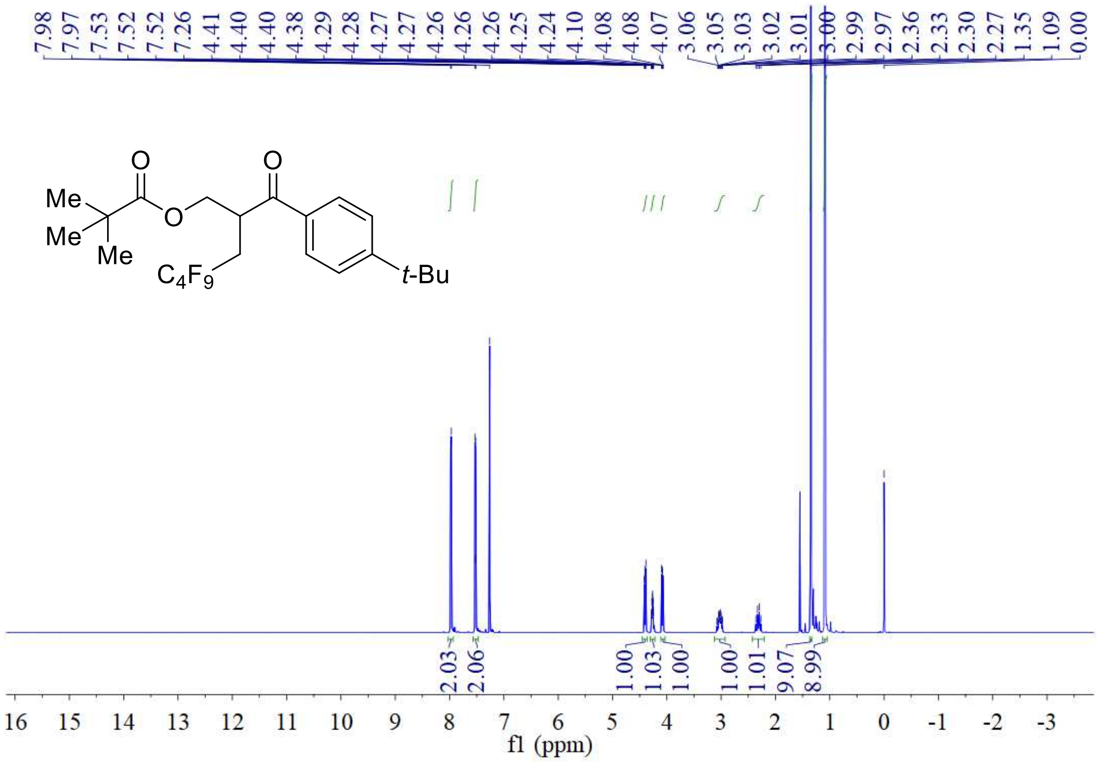

^{19}F NMR (564 MHz, $CDCl_3$)

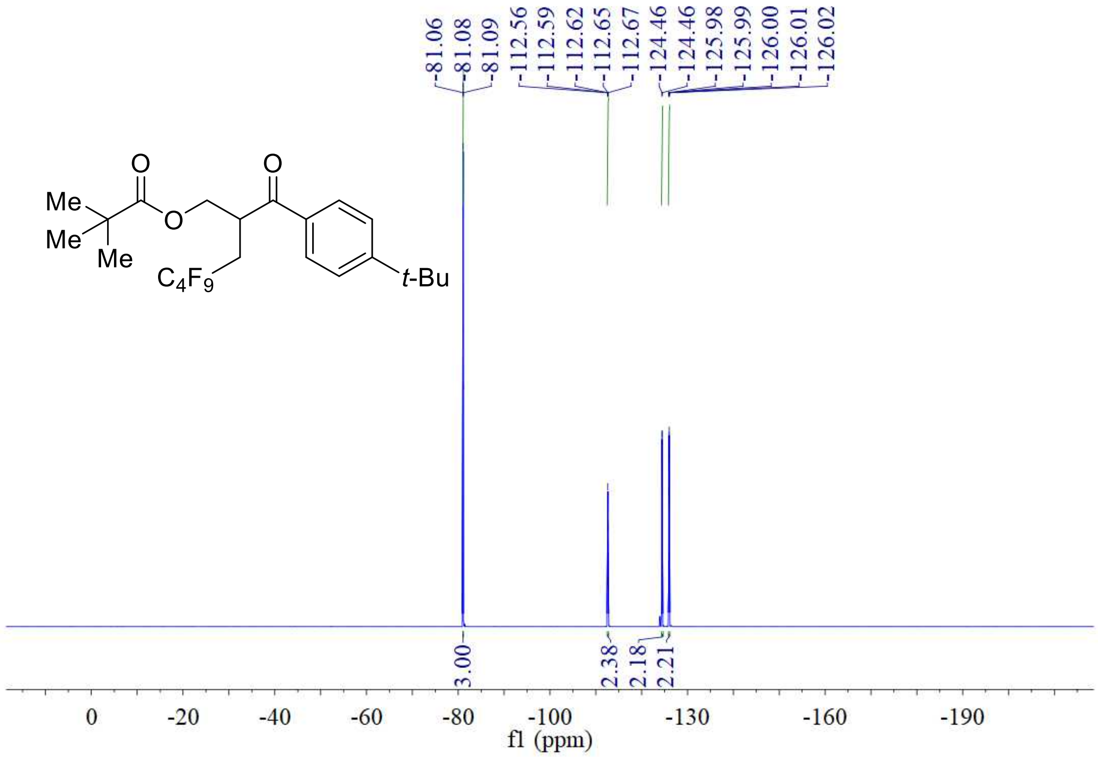

^{13}C NMR (100 MHz, $CDCl_3$)

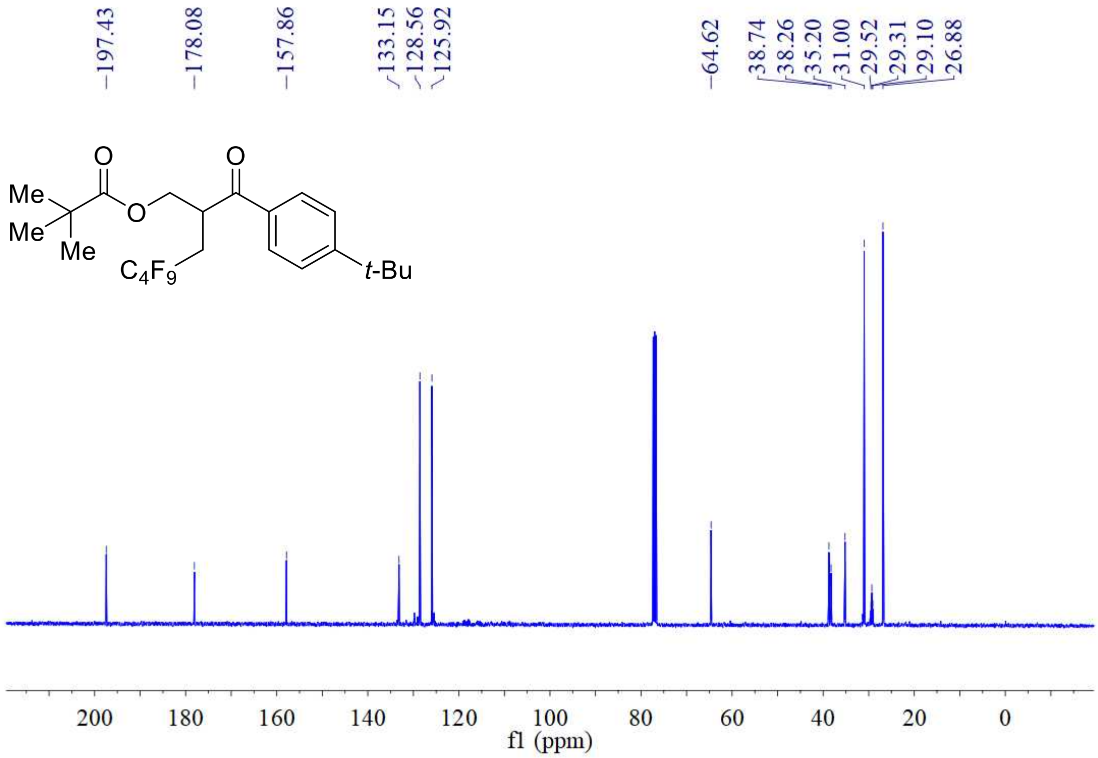

Supplementary Figure 36: NMR Spectra of 2-(4-(*tert*-Butyl)benzoyl)-4,4,5,5,6,6,7,7,7-nonafluoroheptyl pivalate (5)

^{1}H NMR (600 MHz, $CDCl_3$)

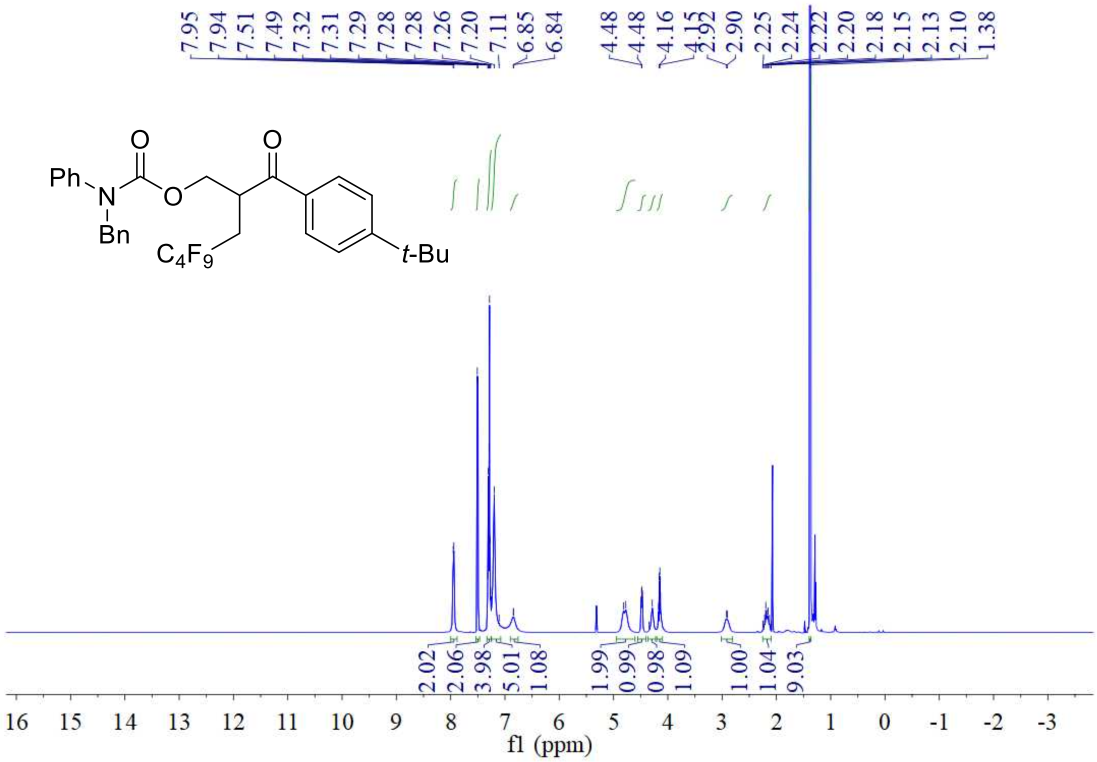

^{19}F NMR (564 MHz, $CDCl_3$)

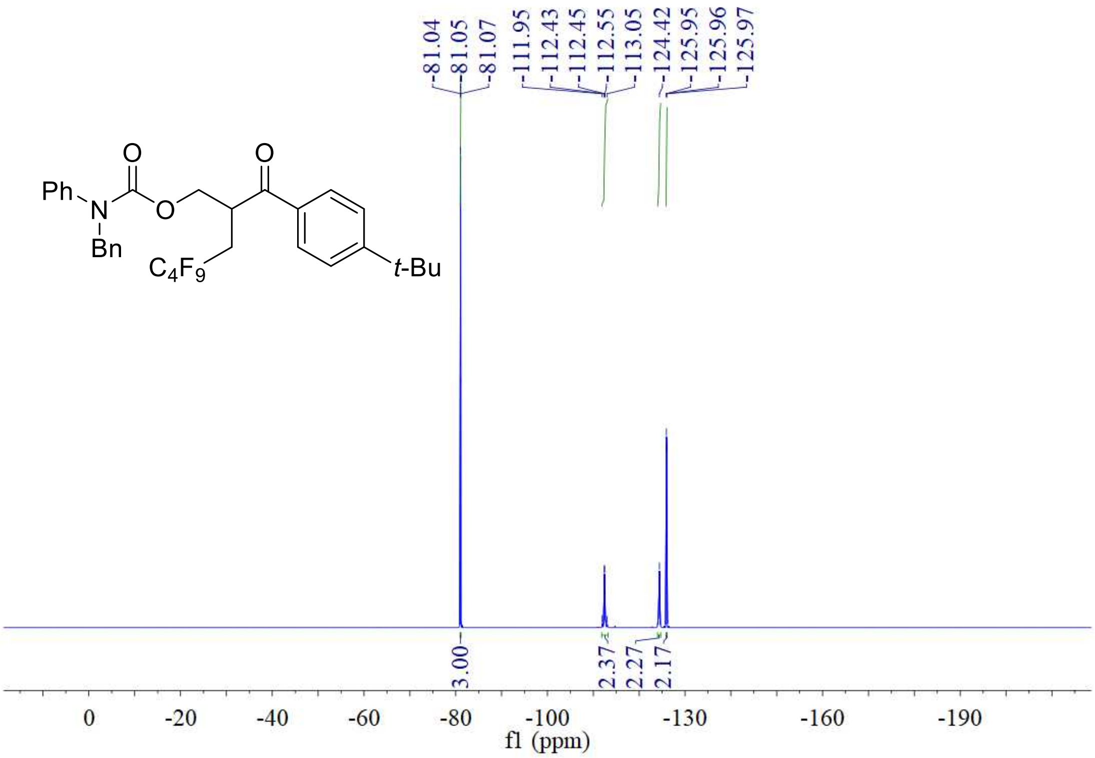

^{13}C NMR (100 MHz, $CDCl_3$)

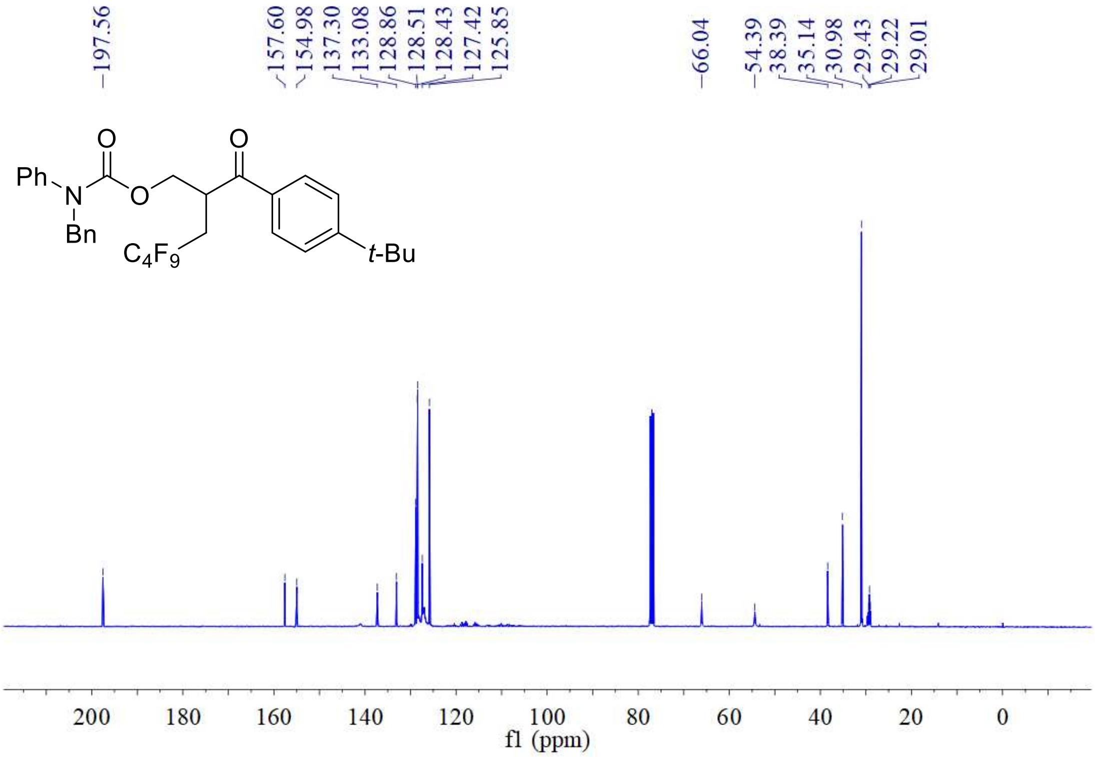

Supplementary Figure 37: NMR Spectra of 2-(4-(*tert*-Butyl)benzoyl)-4,4,5,5,6,6,7,7,7-nonafluoroheptyl benzyl(phenyl)carbamate (6)

^{1}H NMR (600 MHz, $CDCl_3$)

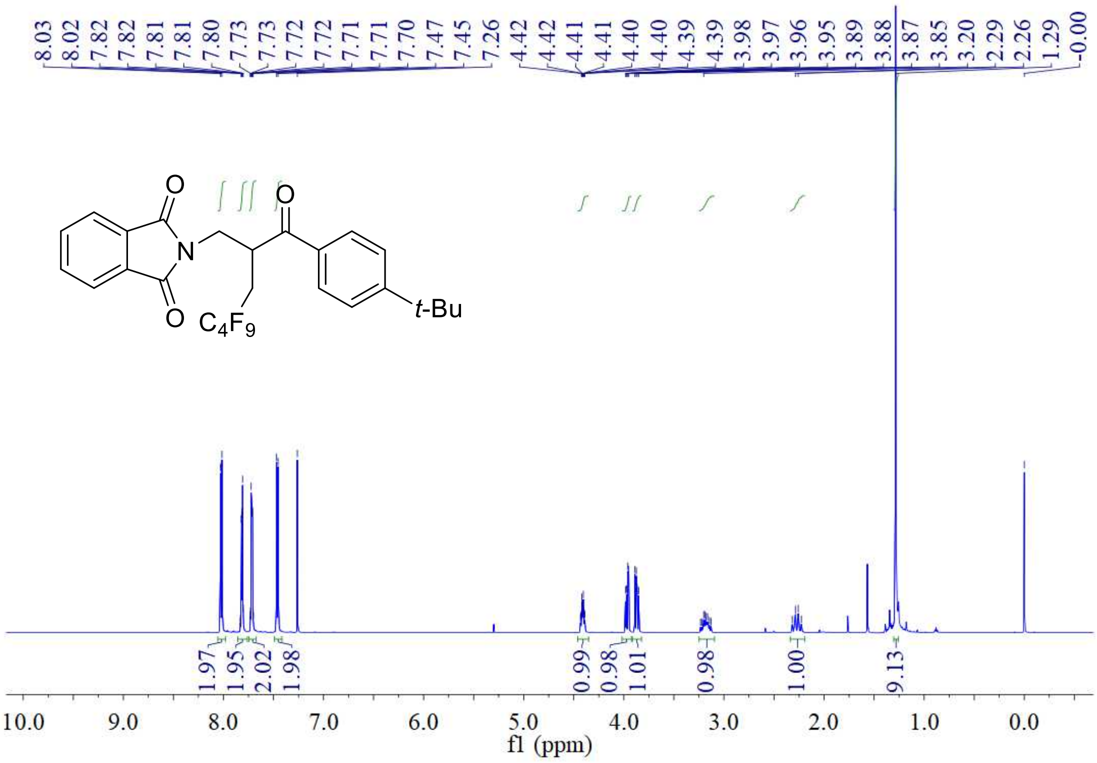

^{19}F NMR (564 MHz, $CDCl_3$)

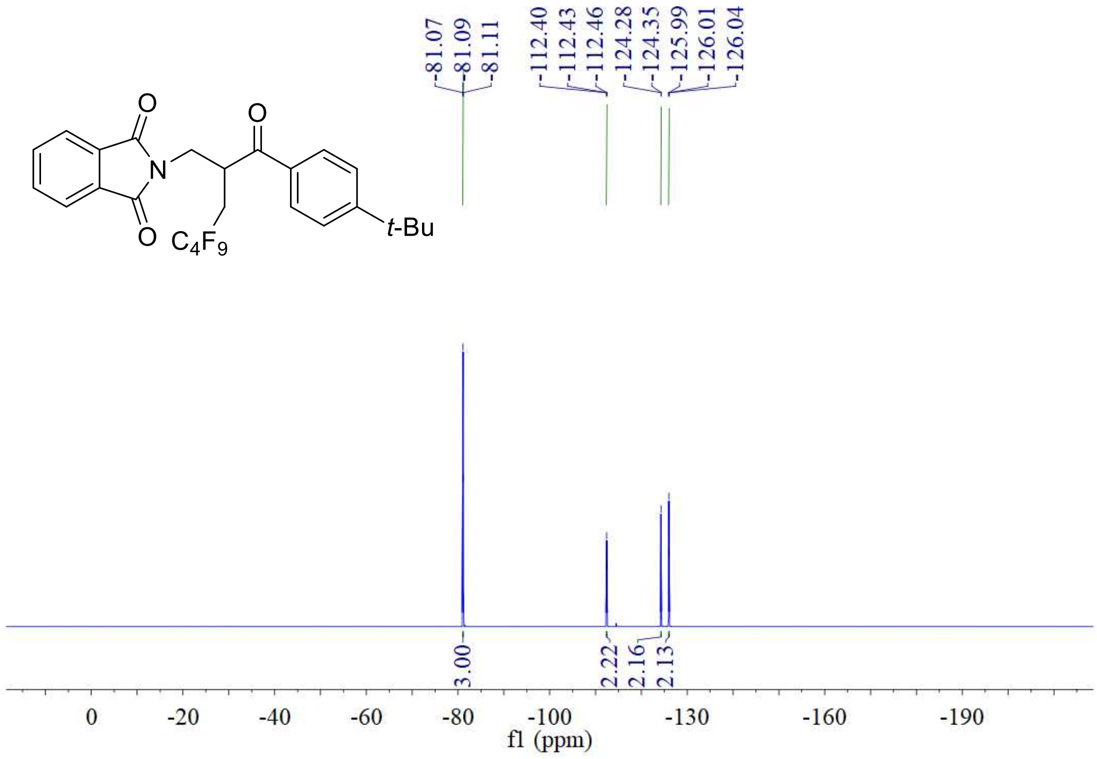

^{13}C NMR (100 MHz, $CDCl_3$)

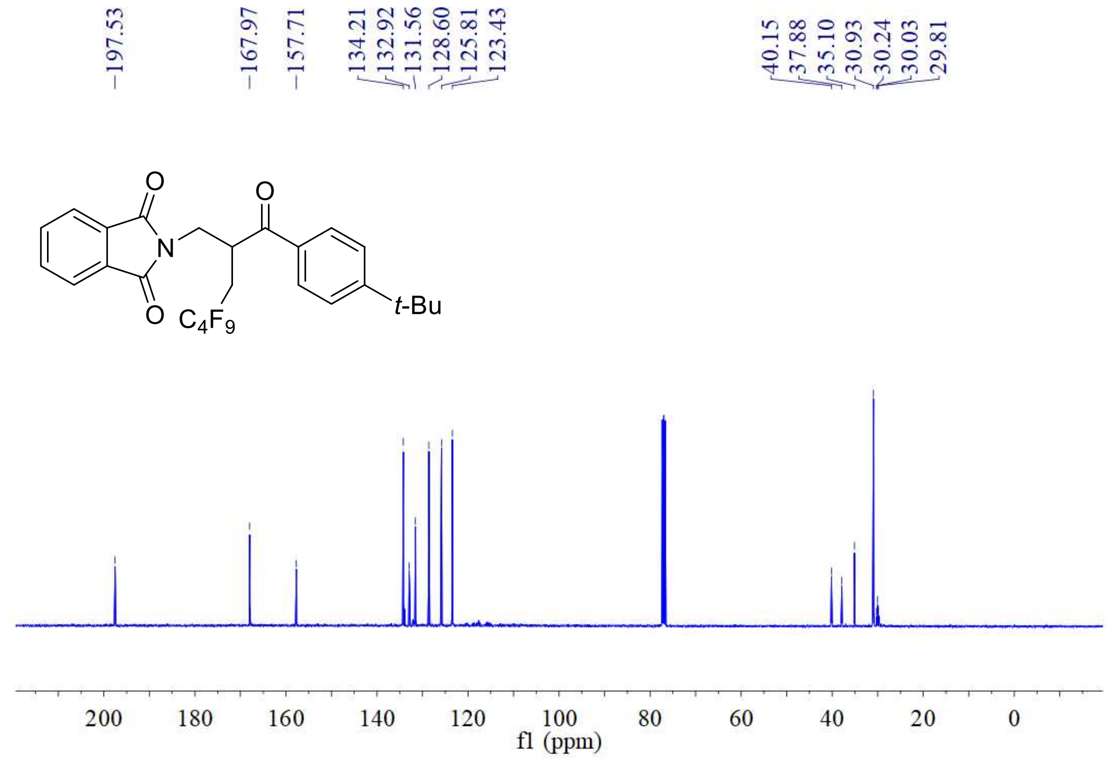

Supplementary Figure 38: NMR Spectra of 2-(2-(4-(*tert*-Butyl)benzoyl)-4,4,5,5,6,6,7,7,7-nonafluoroheptyl)isoindoline-1,3-dione (7)

^{1}H NMR (600 MHz, $CDCl_3$)

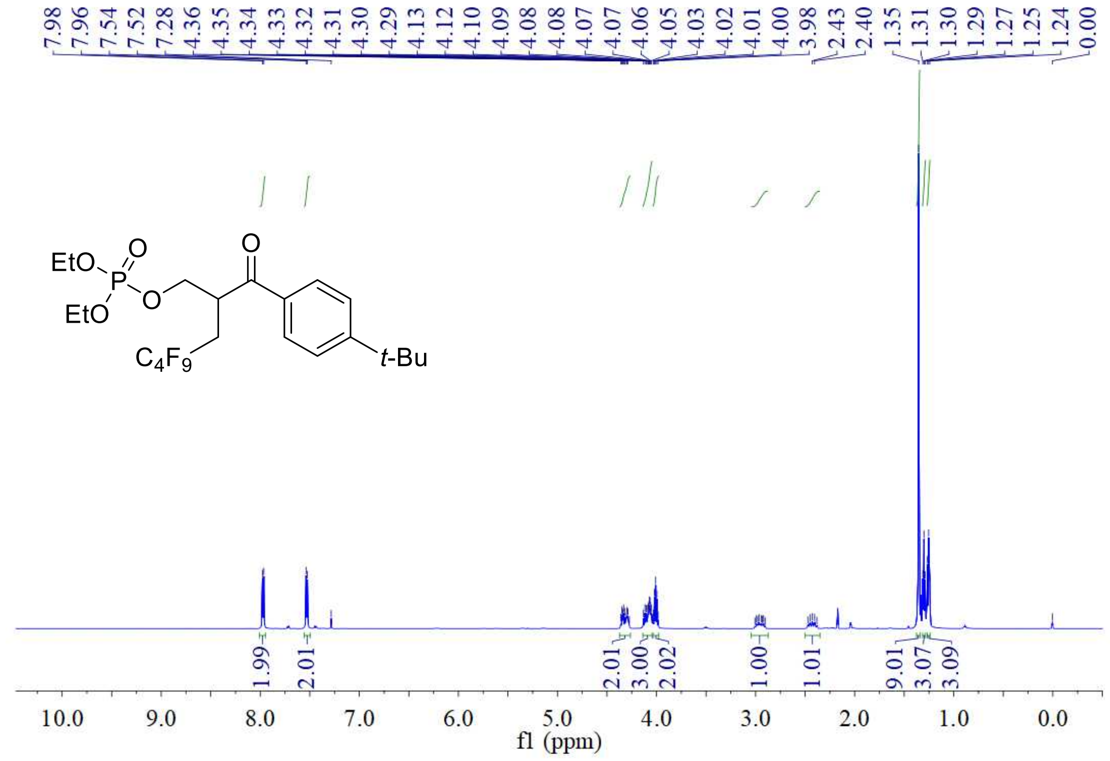

^{19}F NMR (564 MHz, $CDCl_3$)

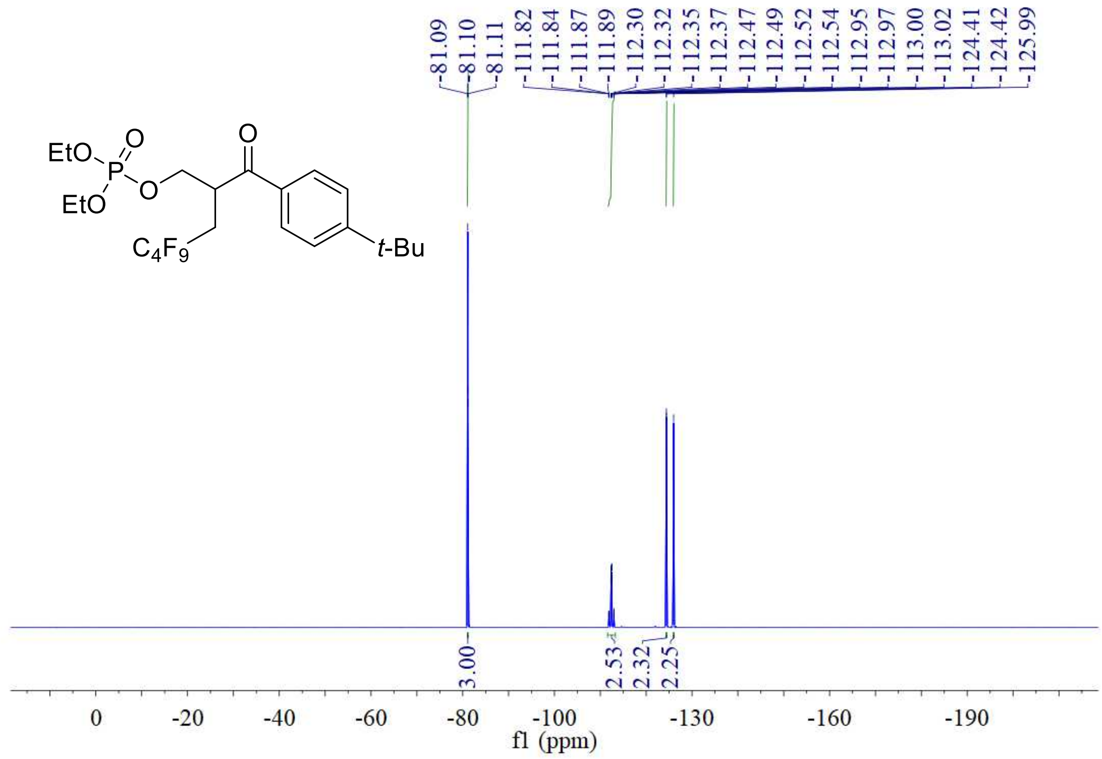

^{13}C NMR (100 MHz, $CDCl_3$)

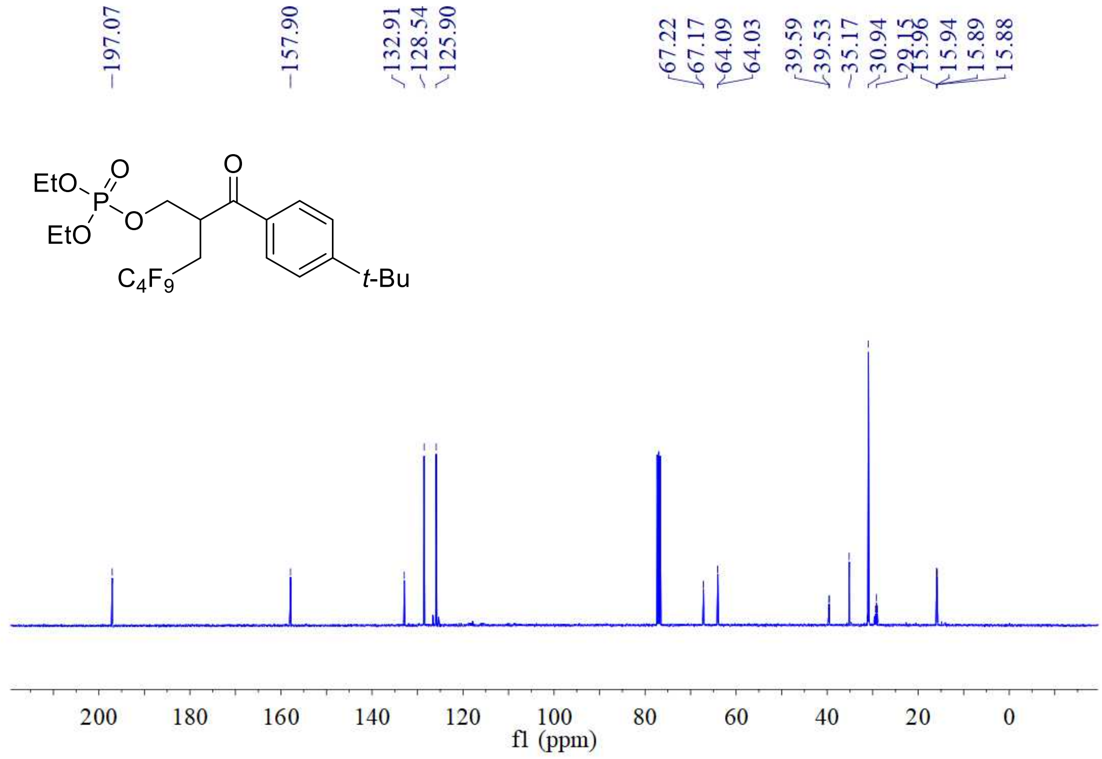

Supplementary Figure 39: NMR Spectra of 2-(4-(*tert*-Butyl)benzoyl)-4,4,5,5,6,6,7,7,7-nonafluoroheptyl diethyl phosphate (8)

^{1}H NMR (600 MHz, $CDCl_3$)

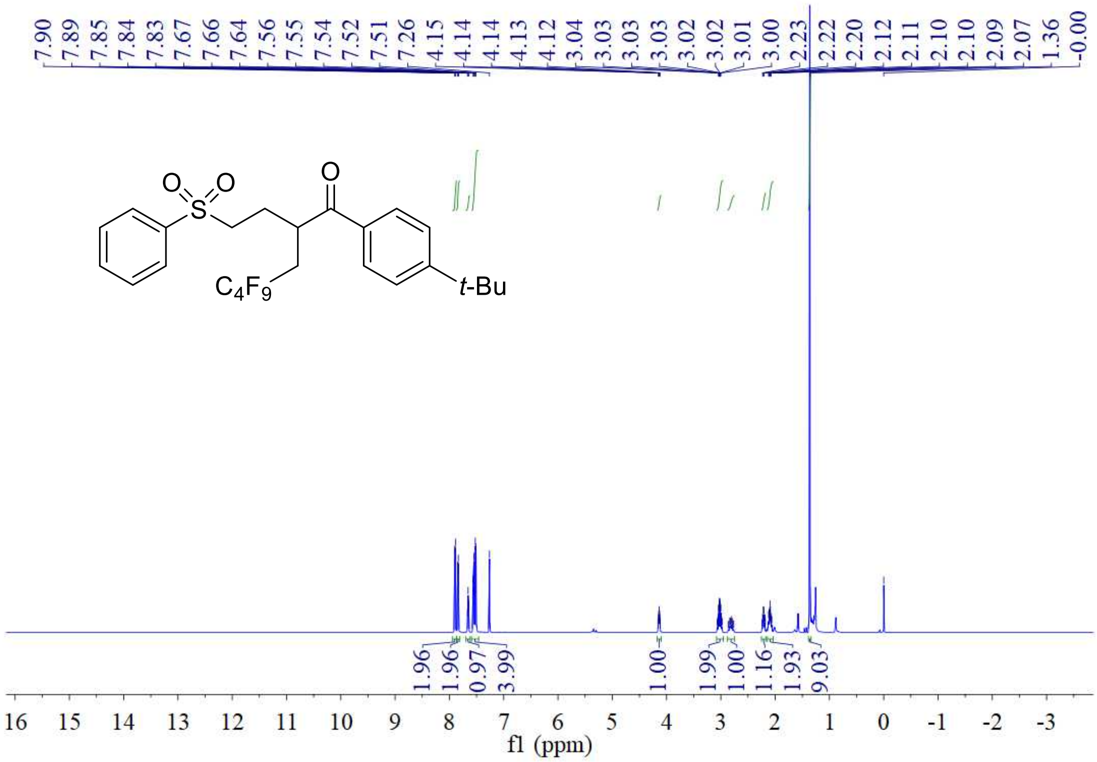

^{19}F NMR (564 MHz, $CDCl_3$)

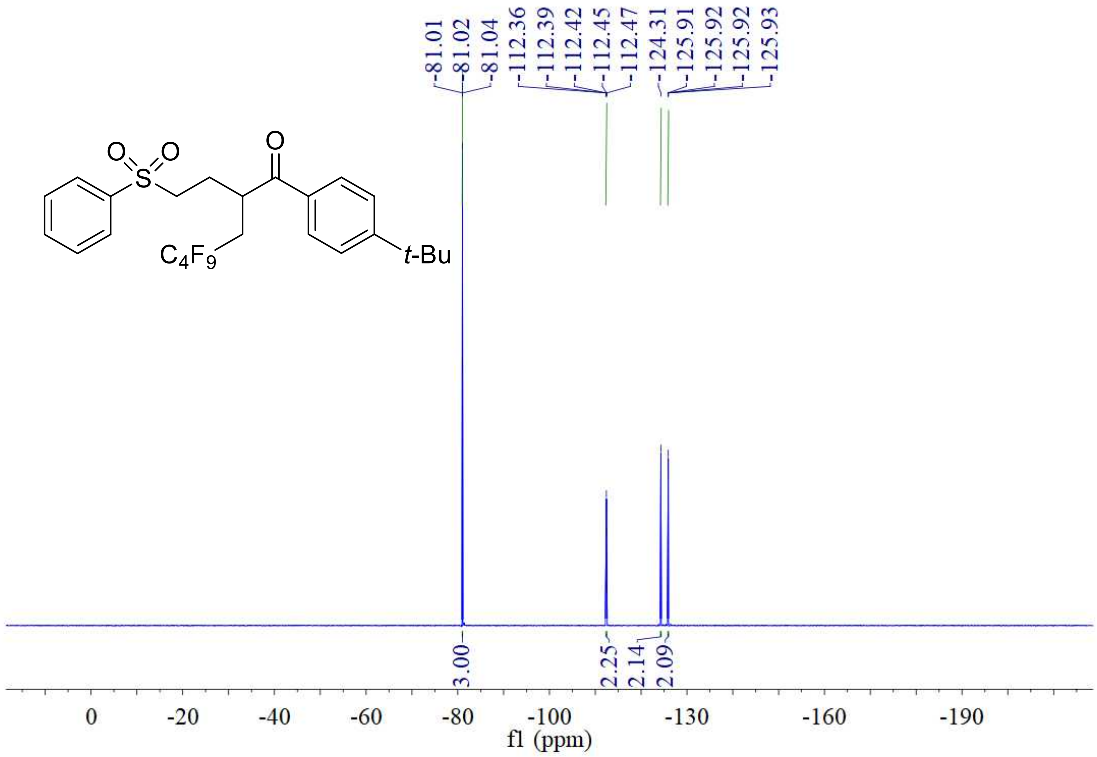

^{13}C NMR (150 MHz, $CDCl_3$)

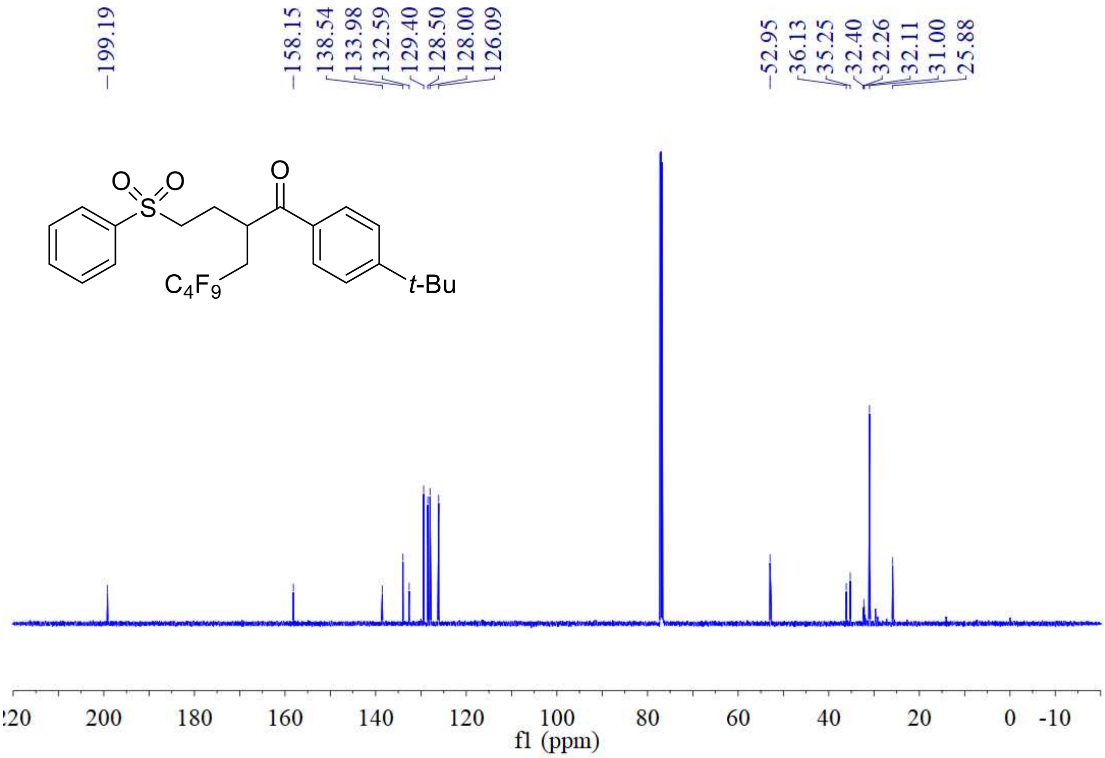

Supplementary Figure 40: NMR Spectra of 1-(4-(*tert*-Butyl)phenyl)-4,4,5,5,6,6,7,7,7-nonafluoro-2-(2-(phenylsulfonyl)ethyl)heptan-1-one (9)

^{1}H NMR (600 MHz, $CDCl_3$)

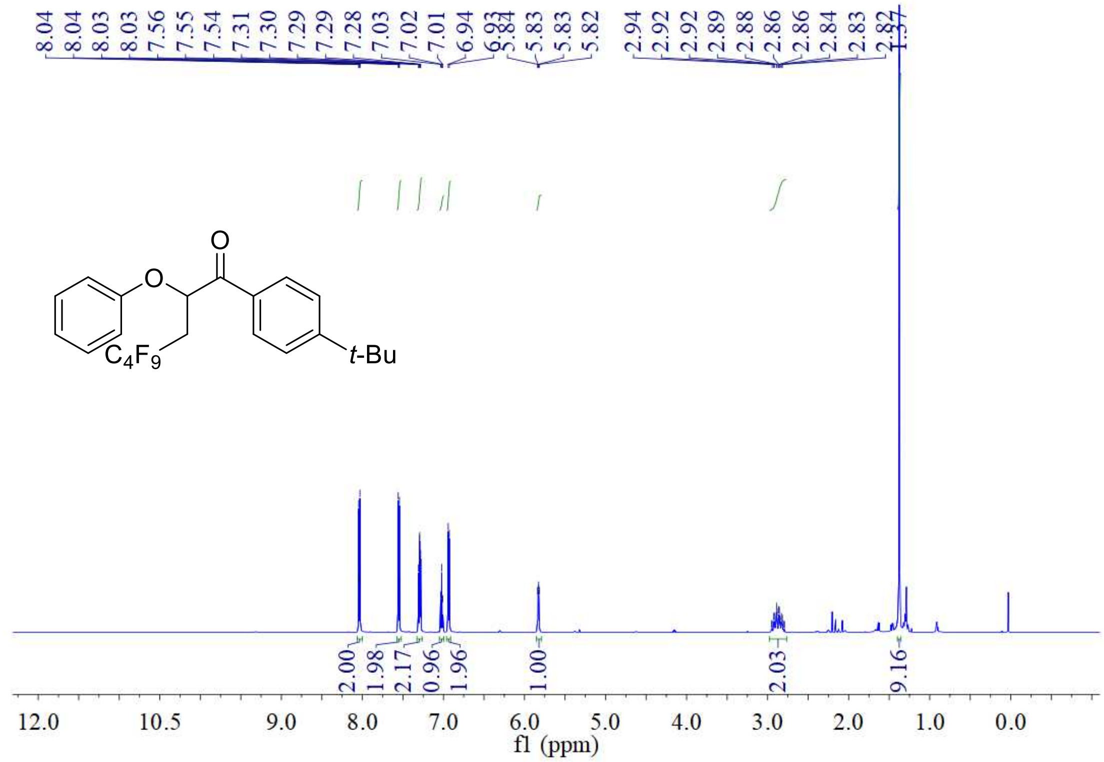

^{19}F NMR (564 MHz, $CDCl_3$)

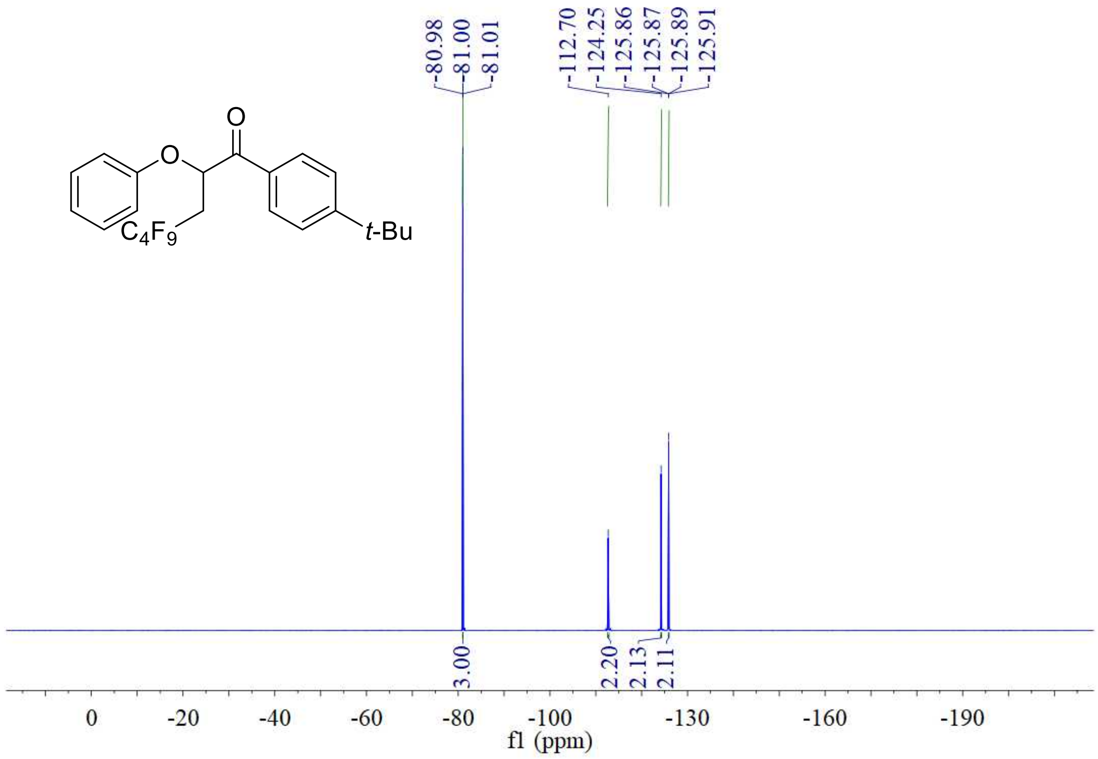

^{13}C NMR (150 MHz, $CDCl_3$)

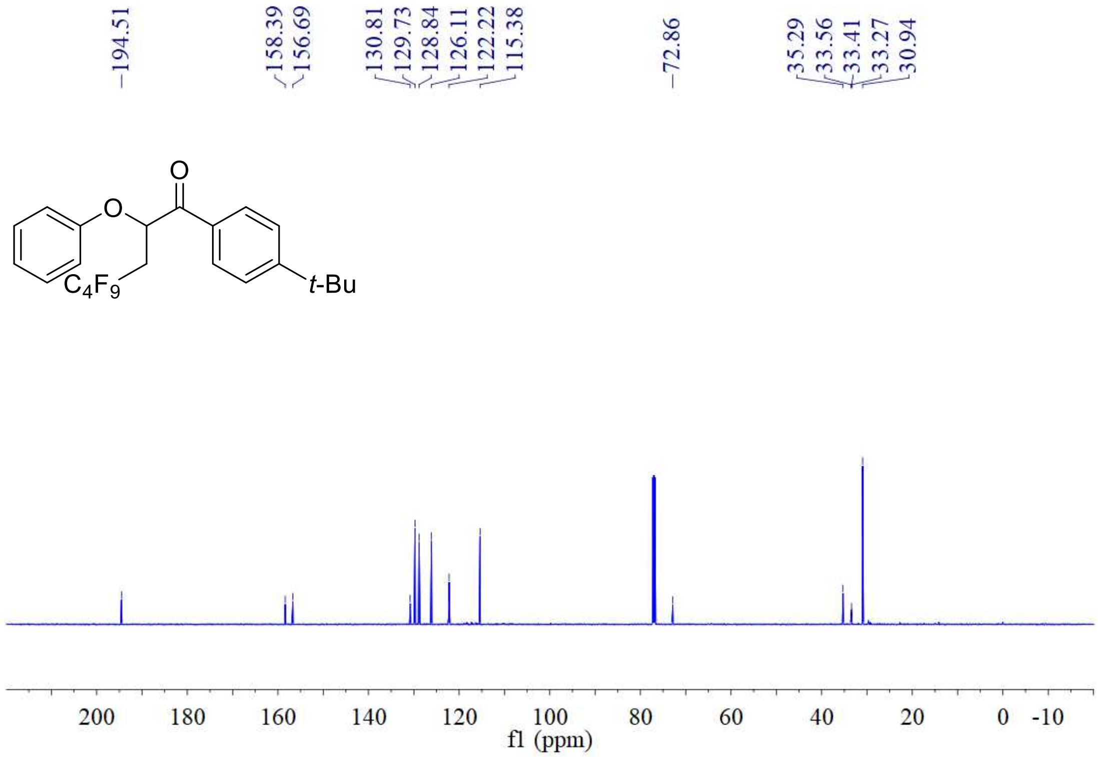

Supplementary Figure 41: NMR Spectra of 1-(4-(*tert*-Butyl)phenyl)-4,4,5,5,6,6,7,7,7-nonafluoro-2-phenoxyheptan-1-one (10)

^{1}H NMR (600 MHz, $CDCl_3$)

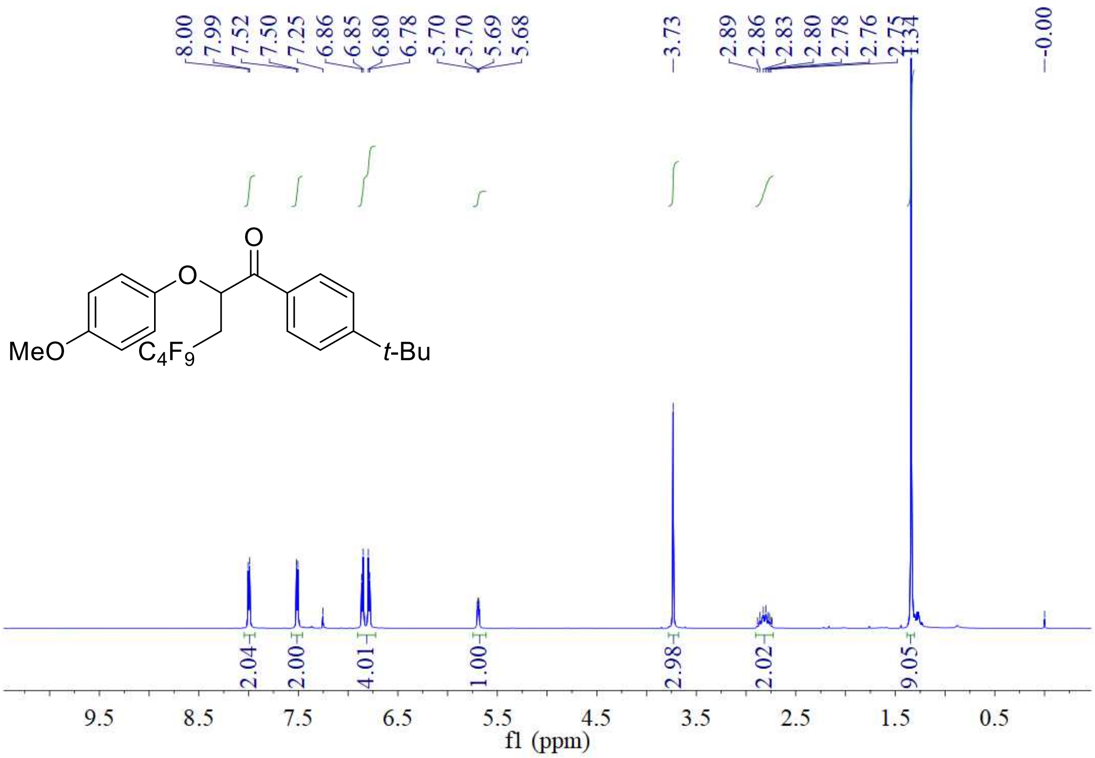

^{19}F NMR (564 MHz, $CDCl_3$)

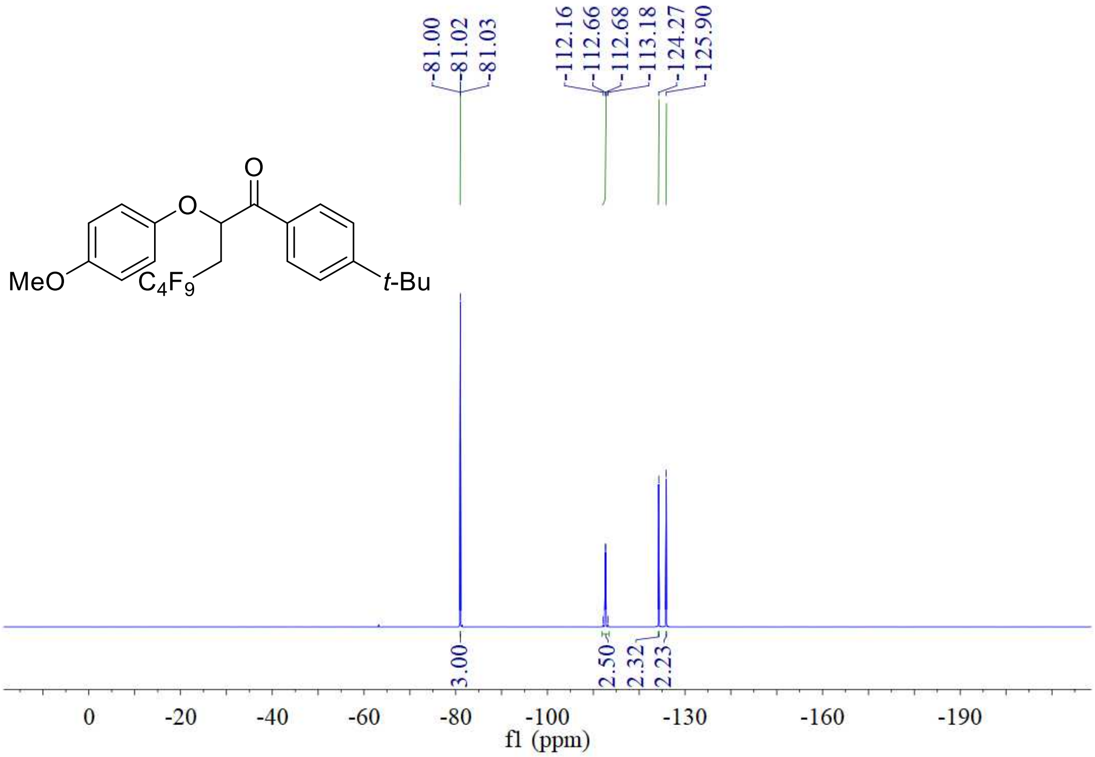

^{13}C NMR (150 MHz, $CDCl_3$)

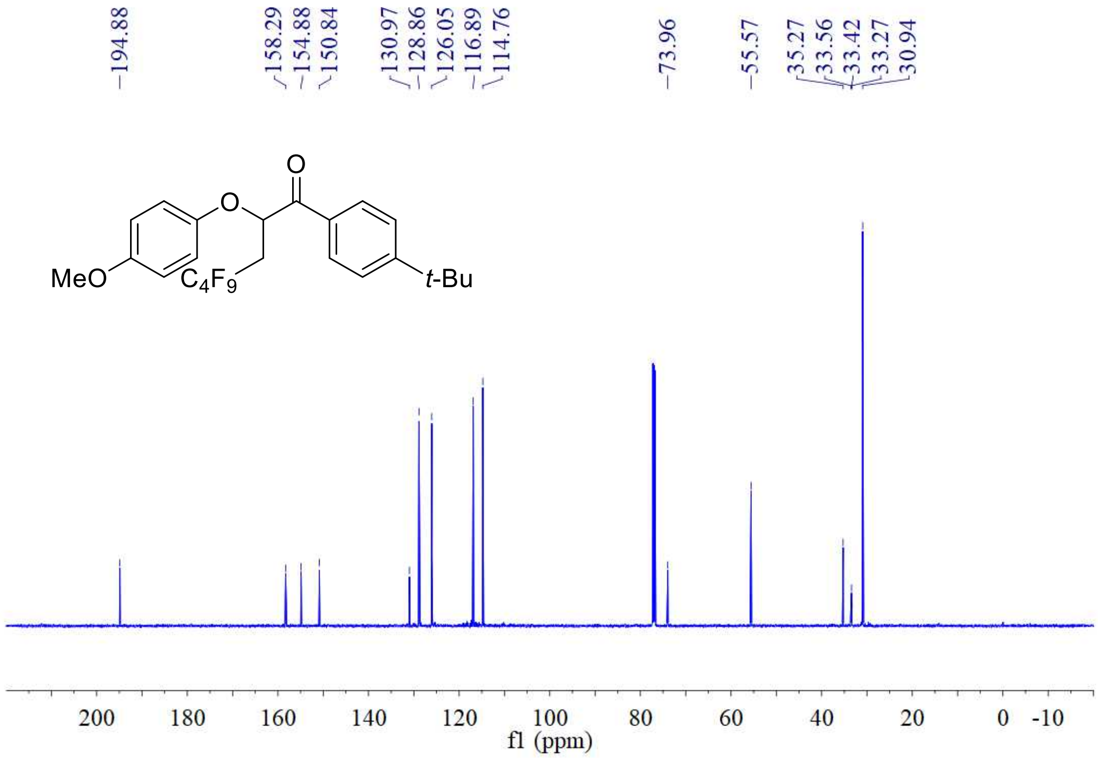

Supplementary Figure 42: NMR Spectra of 1-(4-(*tert*-Butyl)phenyl)-4,4,5,5,6,6,7,7,7-nonafluoro-2-(4-methoxyphenoxy)heptan-1-one (11)

^{1}H NMR (600 MHz, $CDCl_3$)

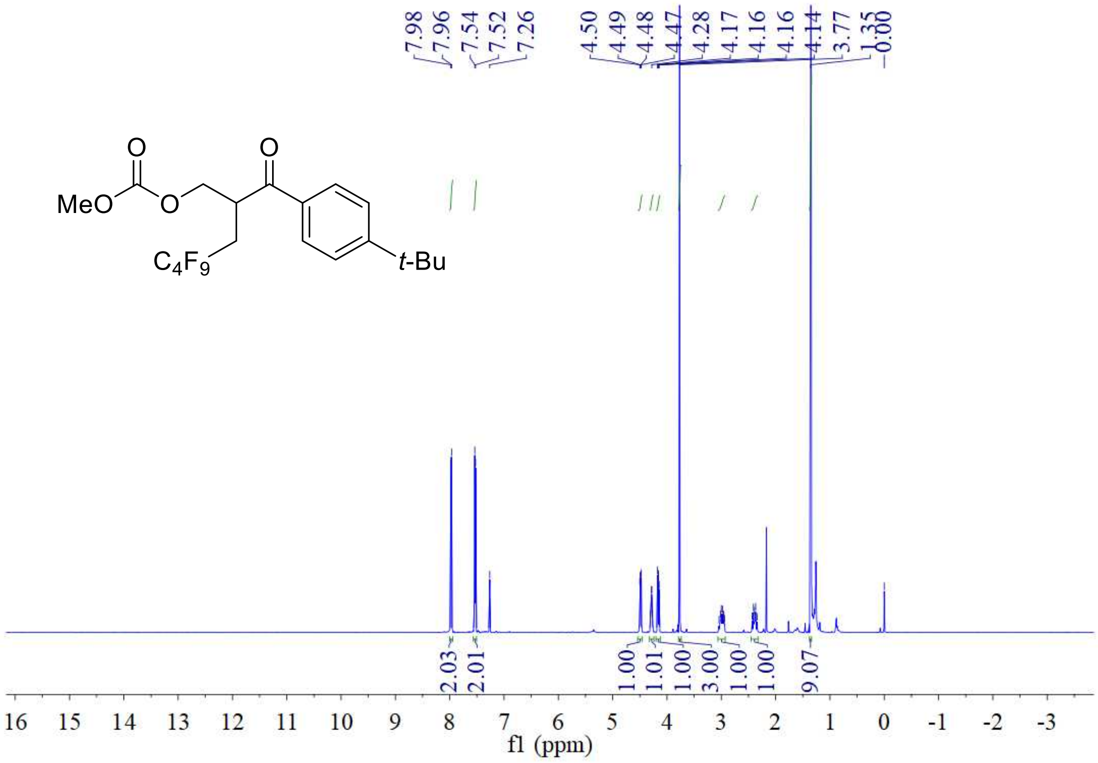

^{19}F NMR (564 MHz, $CDCl_3$)

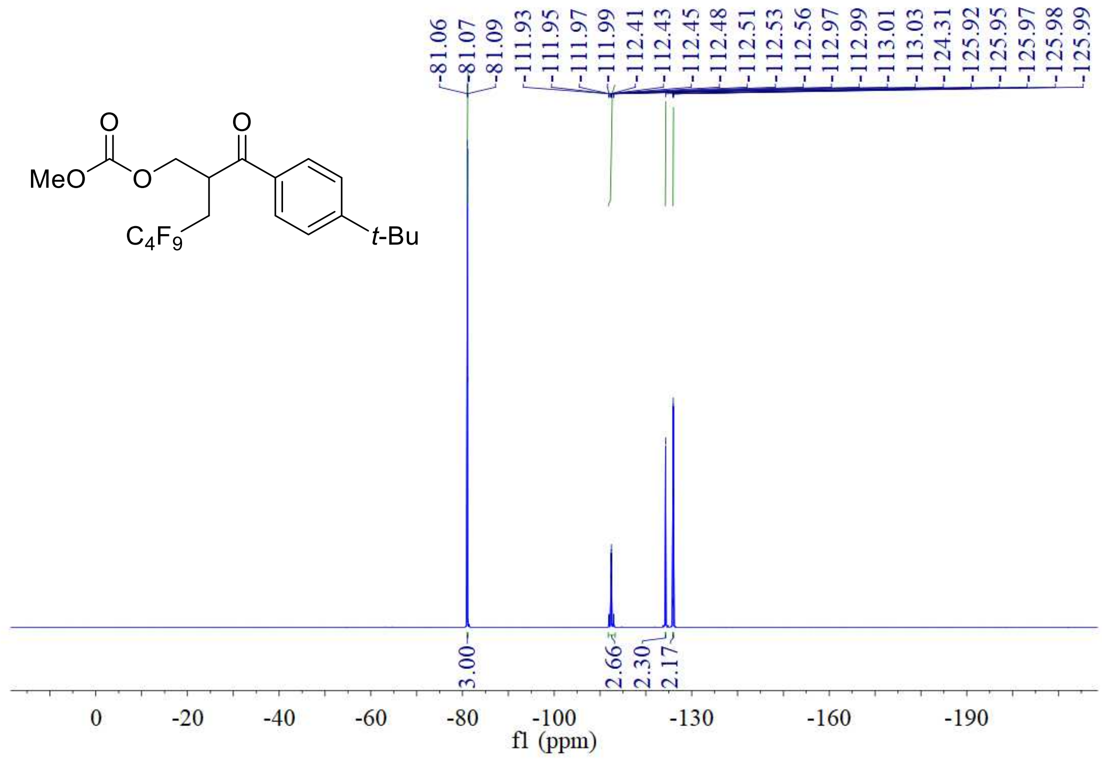

^{13}C NMR (100 MHz, $CDCl_3$)

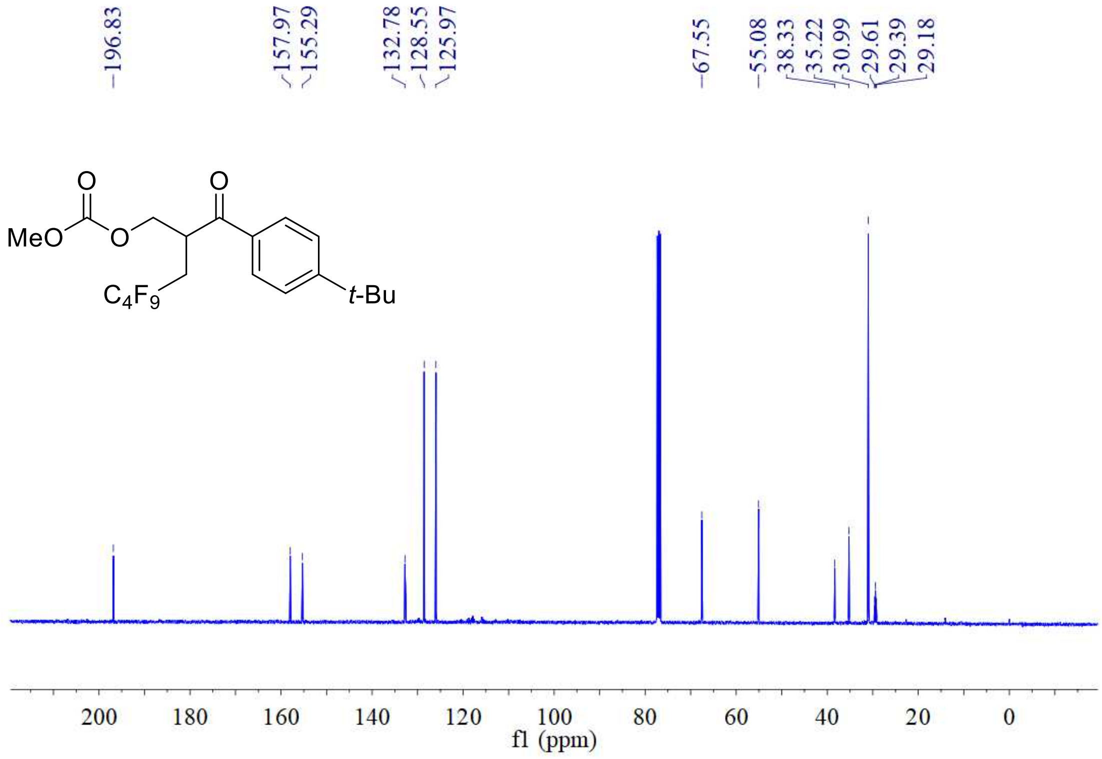

Supplementary Figure 43: NMR Spectra of 2-(4-(*tert*-Butyl)benzoyl)-4,4,5,5,6,6,7,7,7-nonafluoroheptyl methyl carbonate (12)

^{1}H NMR (600 MHz, $CDCl_3$)

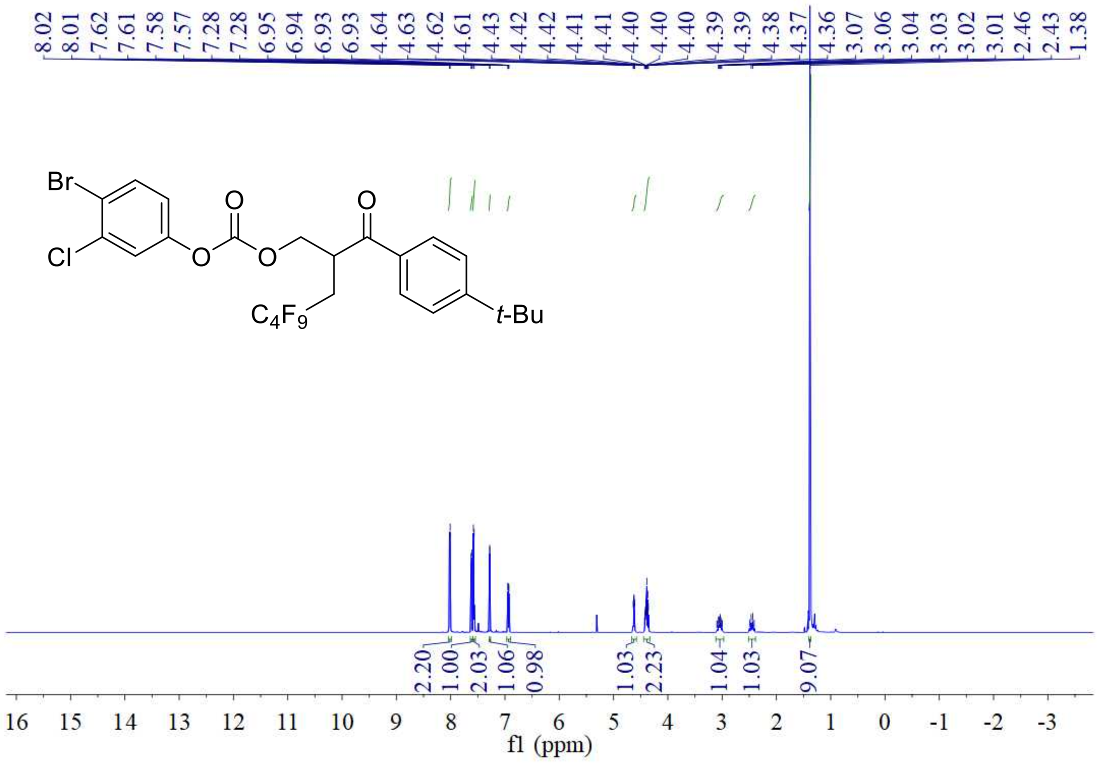

^{19}F NMR (564 MHz, $CDCl_3$)

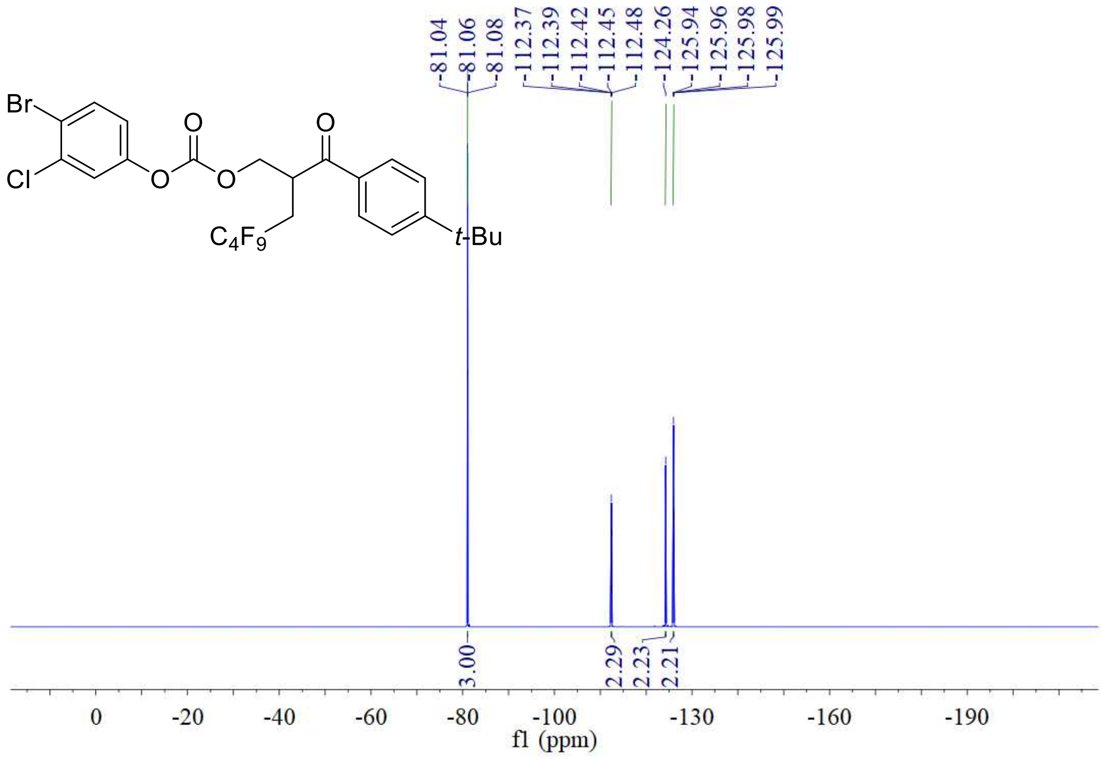

^{13}C NMR (150 MHz, $CDCl_3$)

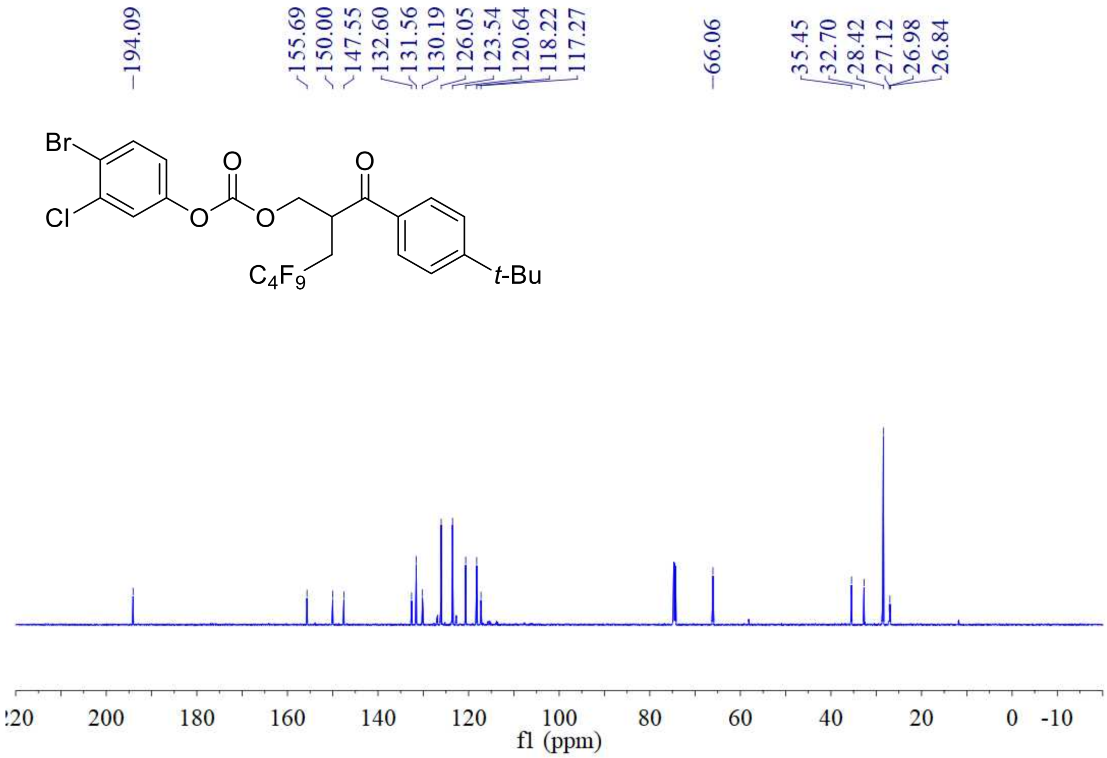

Supplementary Figure 44: NMR Spectra of 4-Bromo-3-chlorophenyl (2-(4-(*tert*-butyl)benzoyl)-4,4,5,5,6,6,7,7,7-nonafluoroheptyl) carbonate (13)

^{1}H NMR (600 MHz, $CDCl_3$)

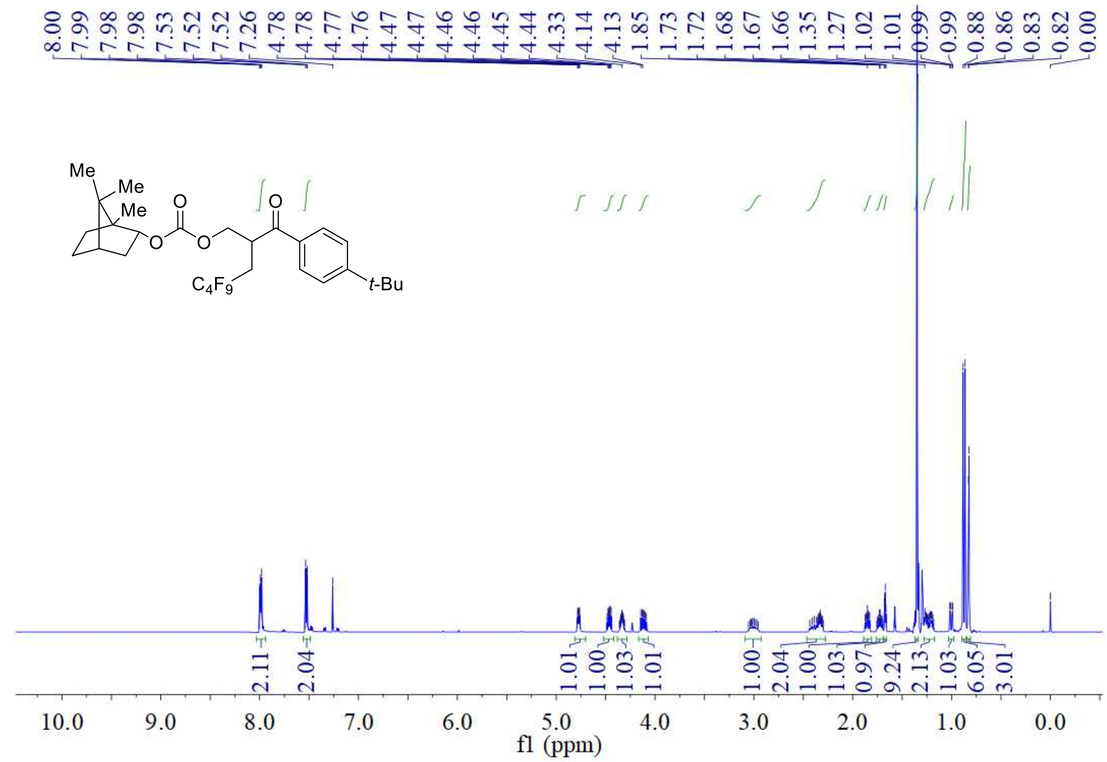

^{19}F NMR (564 MHz, $CDCl_3$)

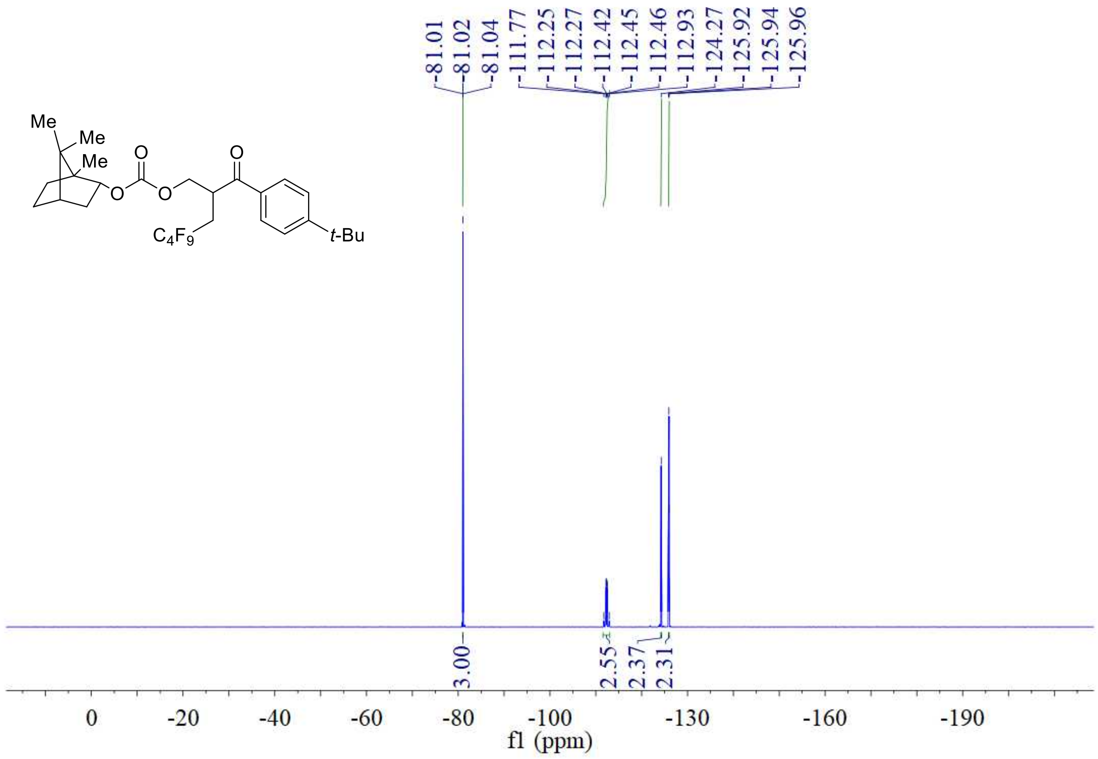

^{13}C NMR (150 MHz, $CDCl_3$)

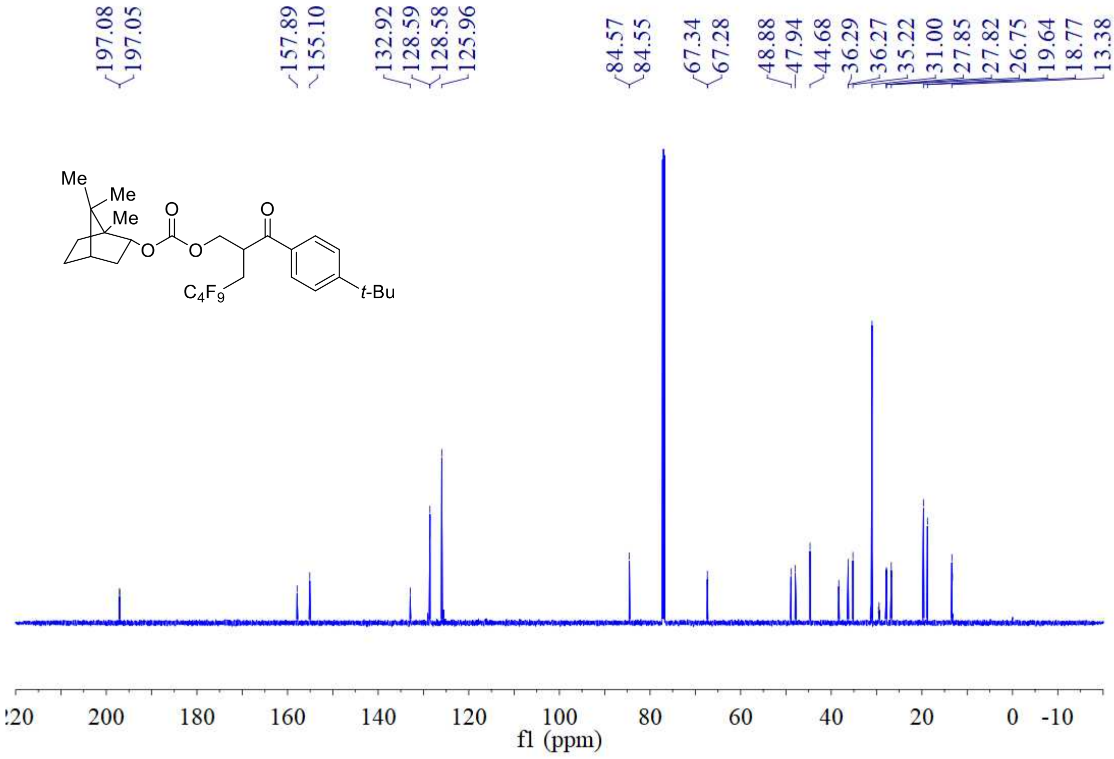

Supplementary Figure 45: NMR Spectra of 2-(4-(*tert*-Butyl)benzoyl)-4,4,5,5,6,6,7,7,7-nonafluoroheptyl (1,7,7-trimethylbicycle[2.2.1]heptan-2-yl) carbonate (14)

^{1}H NMR (600 MHz, $CDCl_3$)

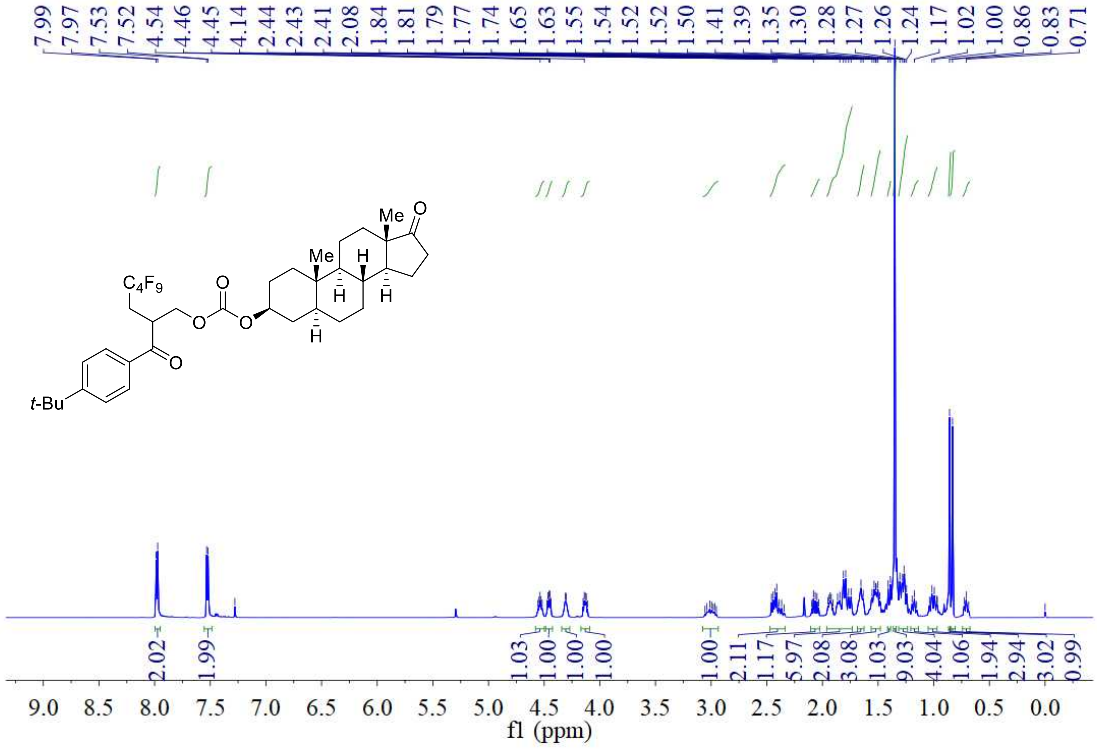

^{19}F NMR (564 MHz, $CDCl_3$)

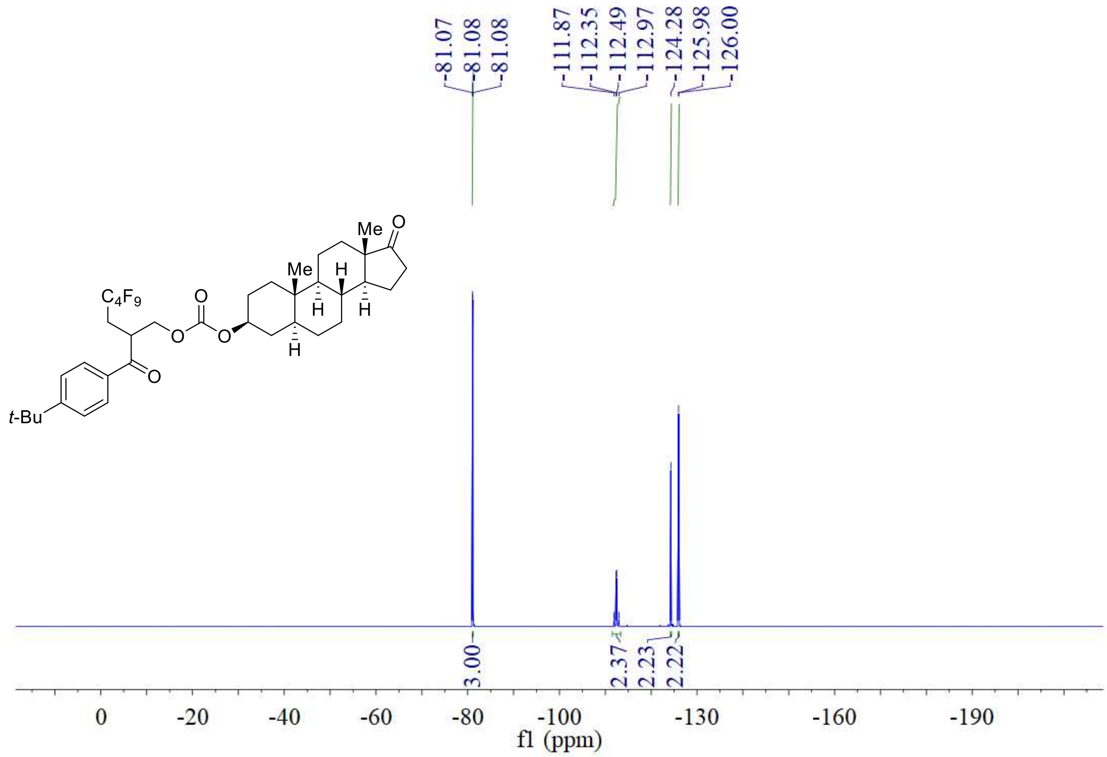

^{13}C NMR (150 MHz, $CDCl_3$)

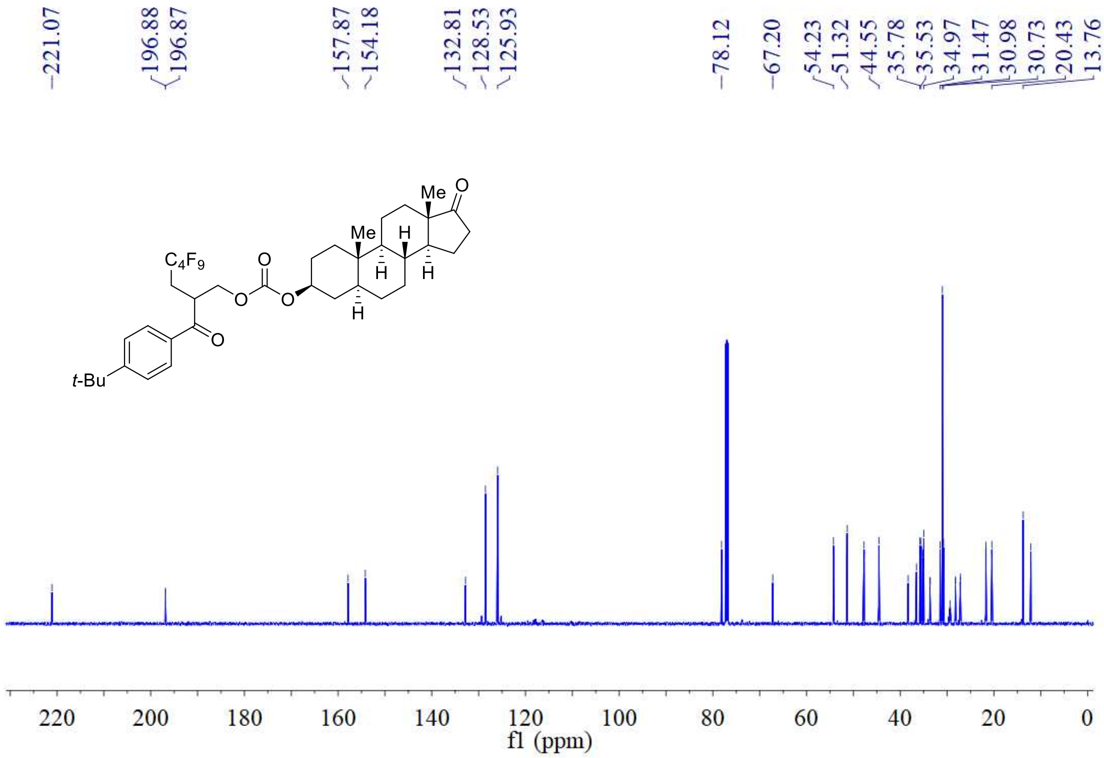

Supplementary Figure 46: NMR Spectra of 2-(4-(*tert*-Butyl)benzoyl)-4,4,5,5,6,6,7,7,7-nonafluoroheptyl (10,13-dimethyl-17-oxohexadecahydro-1H-cyclopenta[a]phenanthren-3-yl) carbonate (15)

^{1}H NMR (600 MHz, $CDCl_3$)

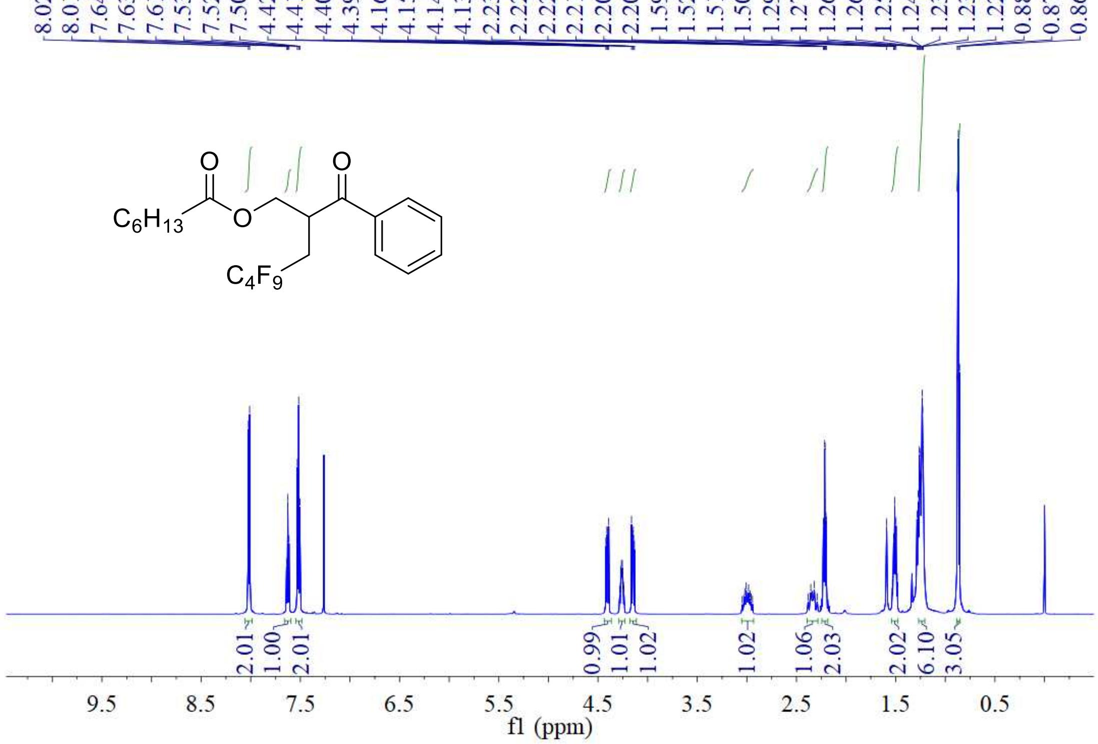

^{19}F NMR (564 MHz, $CDCl_3$)

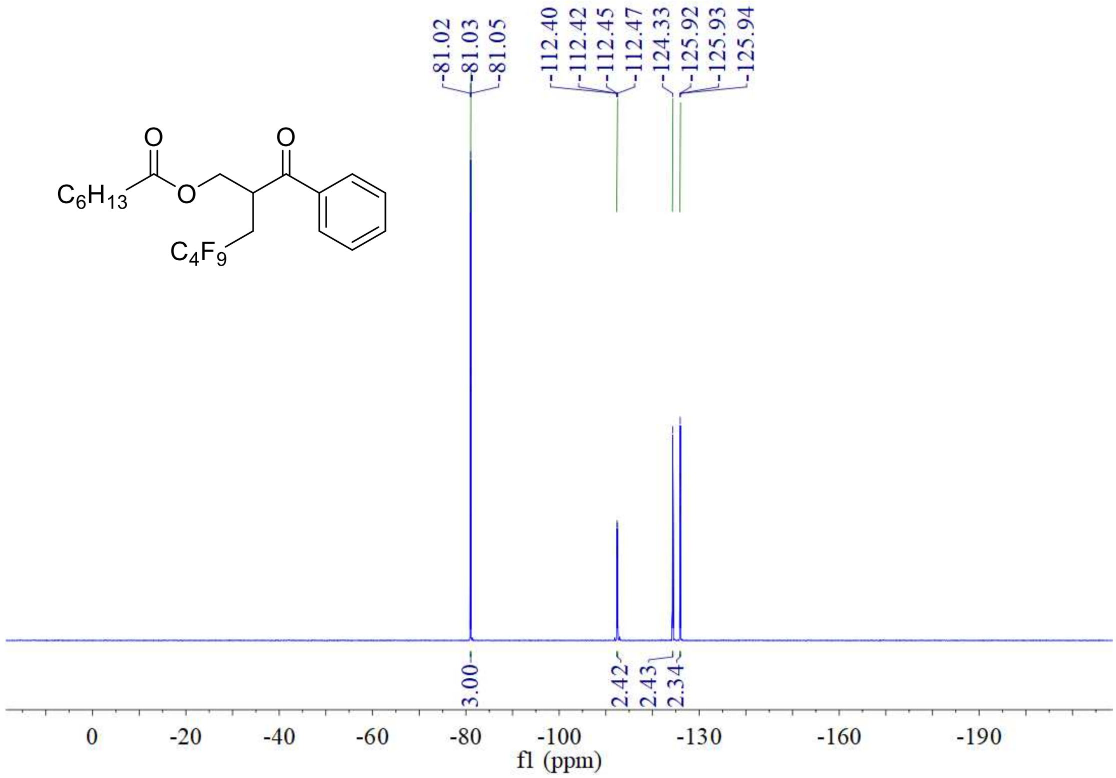

^{13}C NMR (150 MHz, $CDCl_3$)

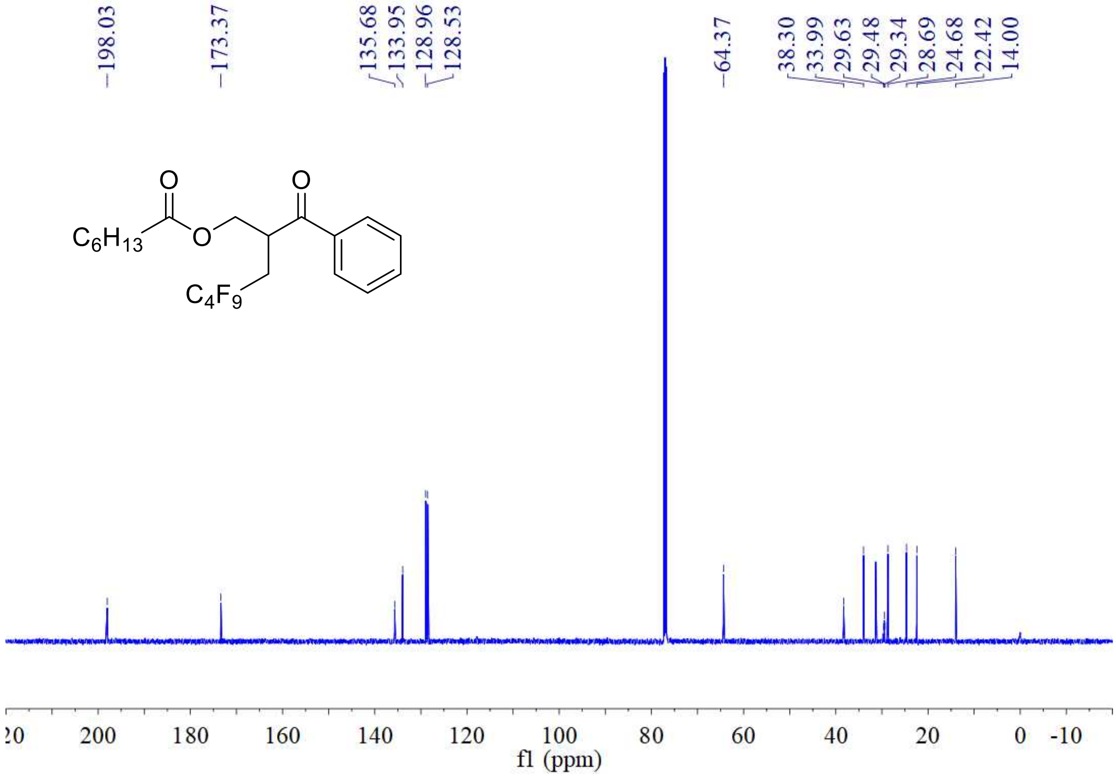

Supplementary Figure 47: NMR Spectra of 2-Benzoyl-4,4,5,5,6,6,7,7,7-nonafluoroheptyl heptanoate (16)

^{1}H NMR (400 MHz, CDCl$_3$)

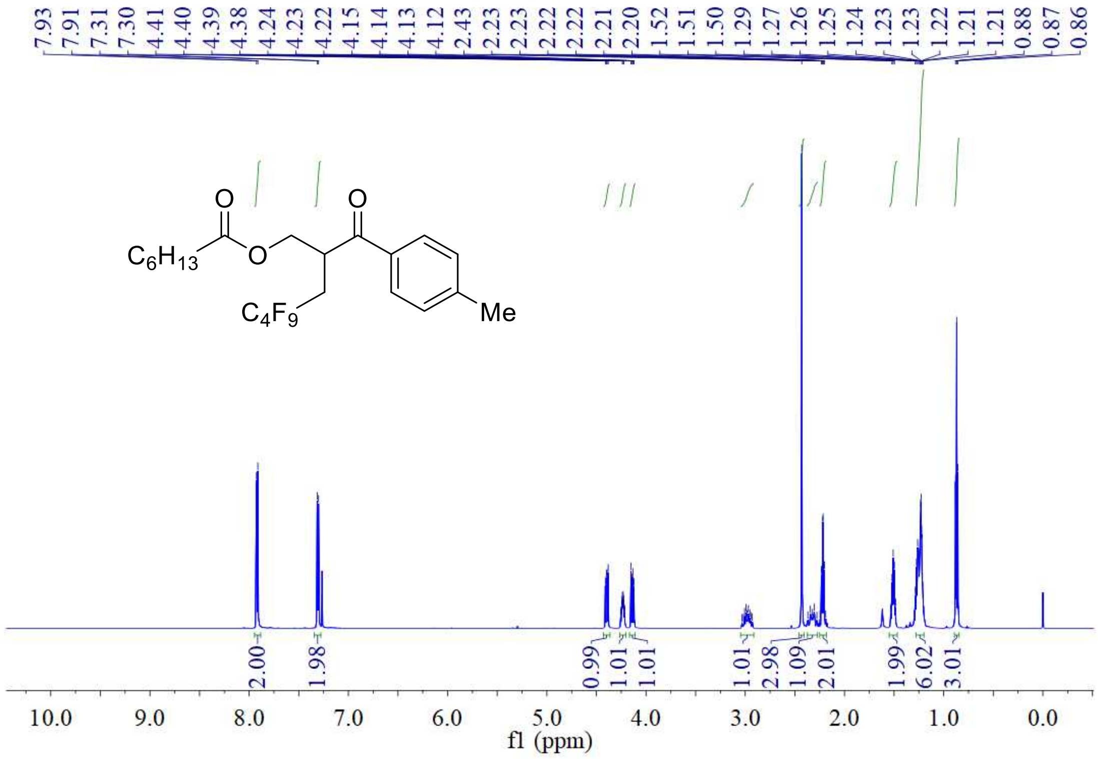

^{19}F NMR (376 MHz, CDCl$_3$)

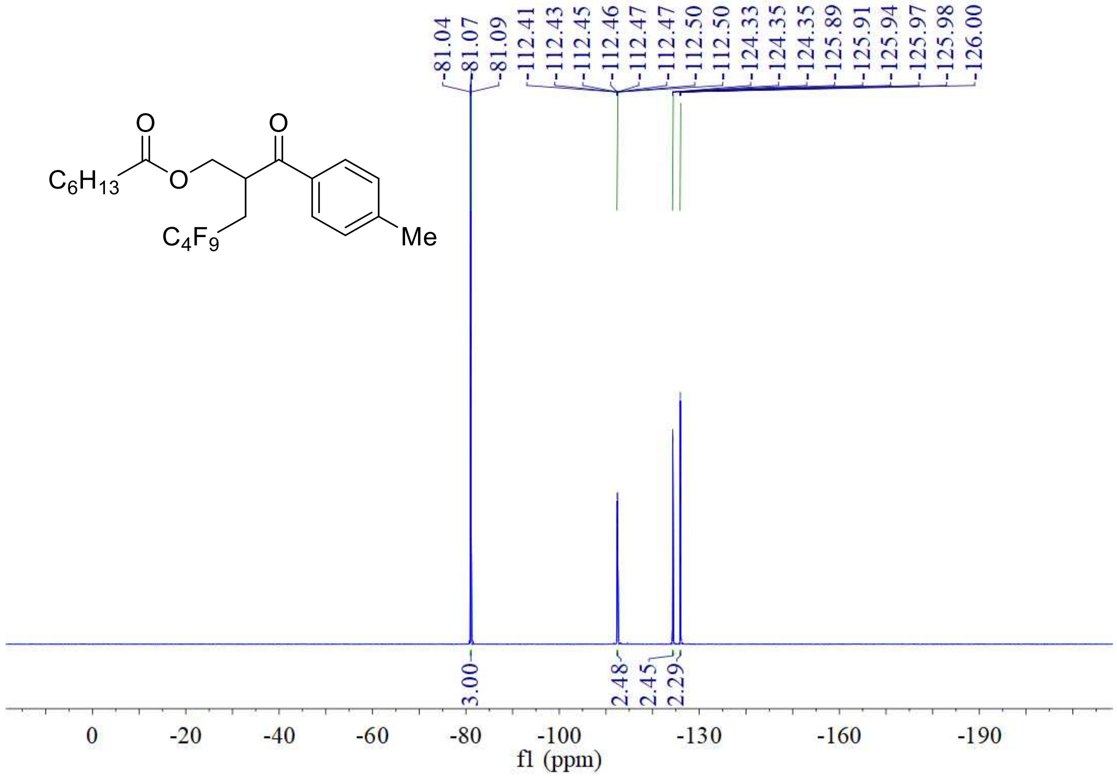

^{13}C NMR (150 MHz, CDCl$_3$)

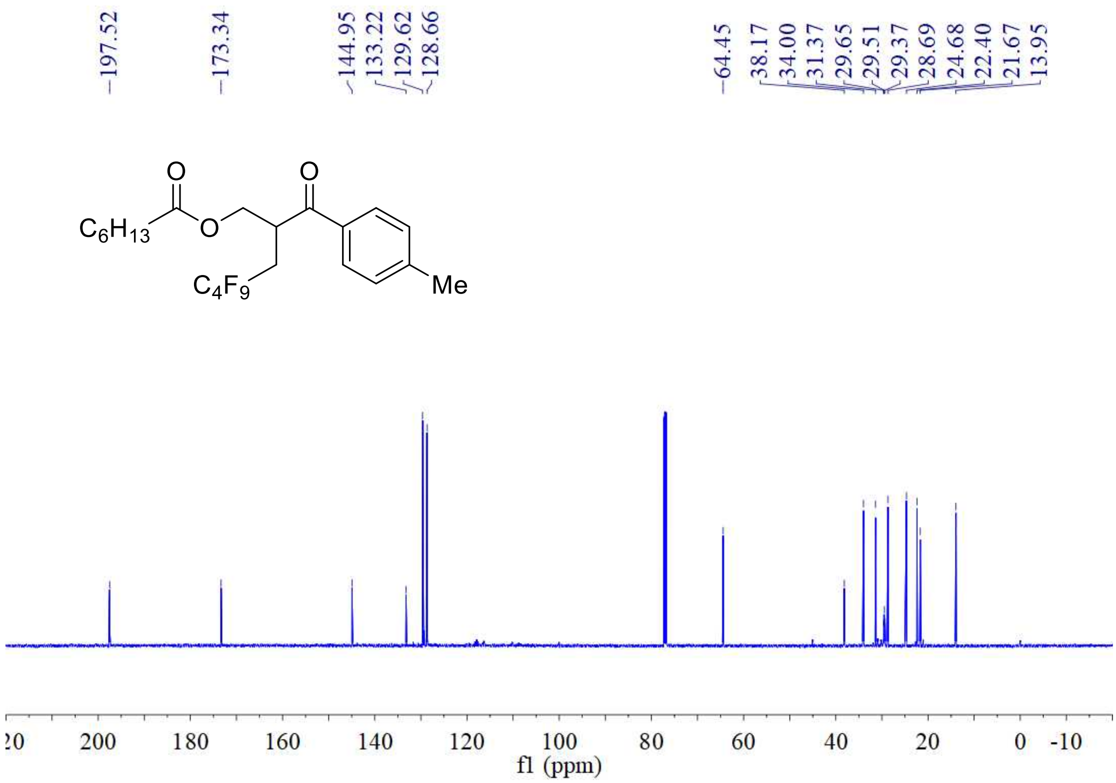

Supplementary Figure 48: NMR Spectra of 4,4,5,5,6,6,7,7,7-Nonafluoro-2-(4-methylbenzoyl)heptyl heptanoate (17)

^{1}H NMR (600 MHz, $CDCl_3$)

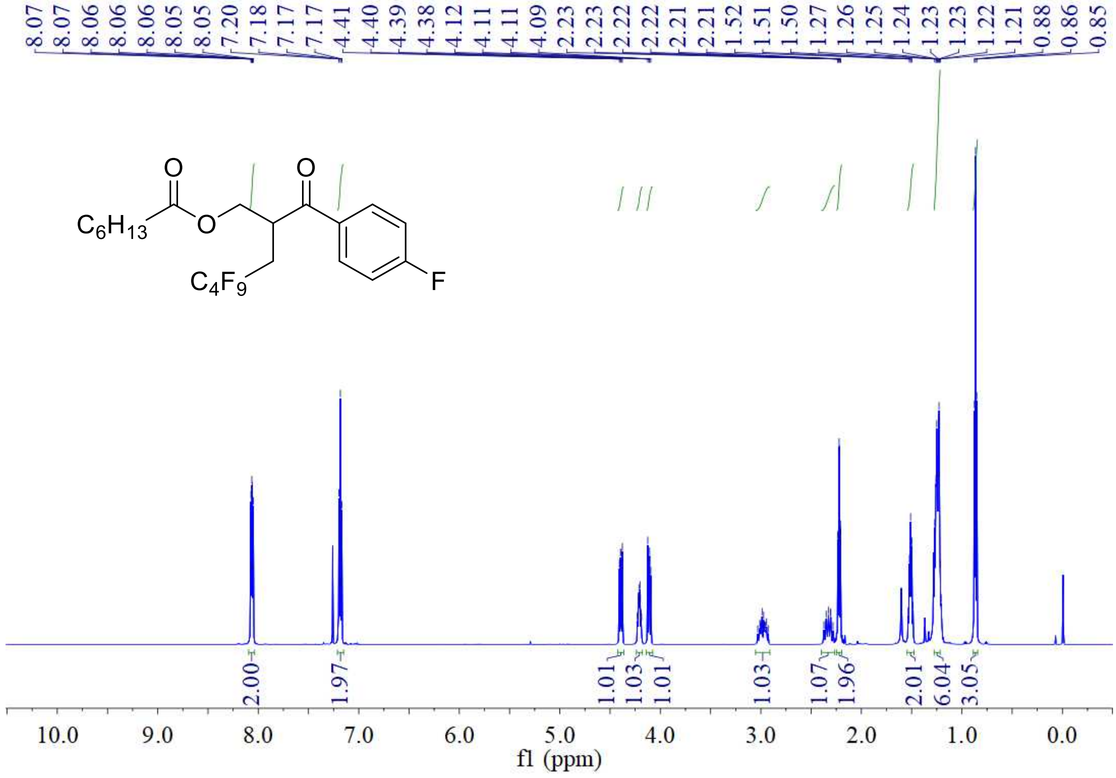

^{19}F NMR (376 MHz, $CDCl_3$)

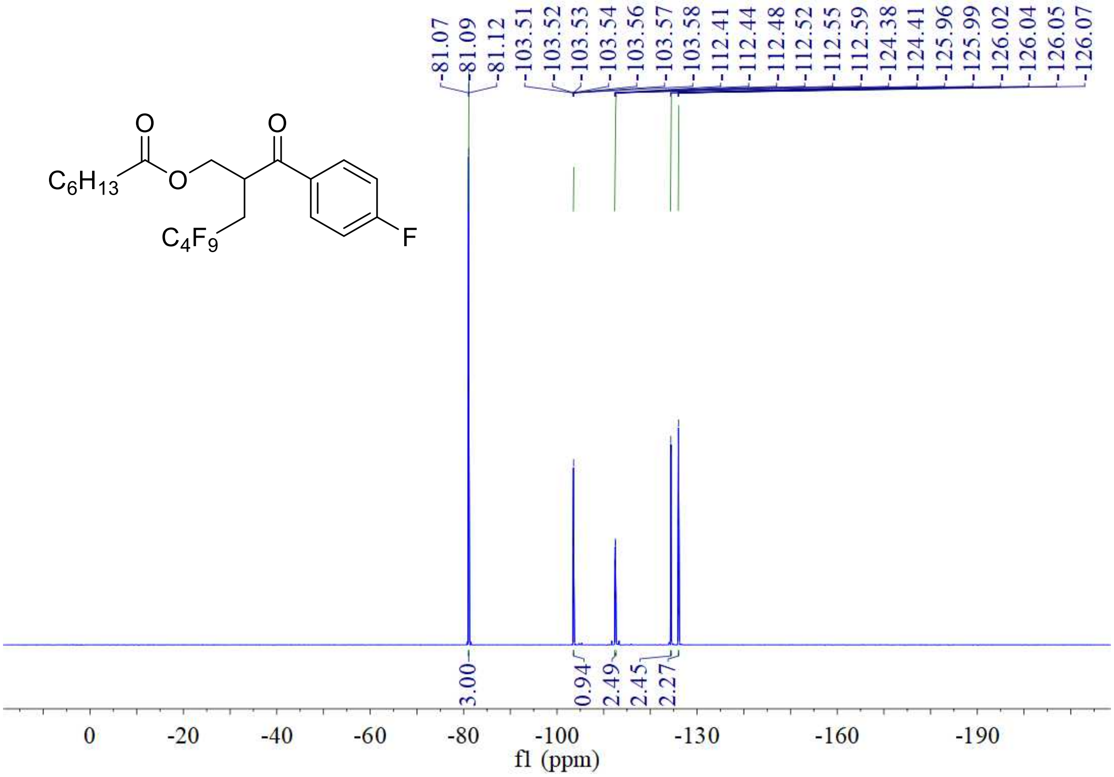

^{13}C NMR (150 MHz, $CDCl_3$)

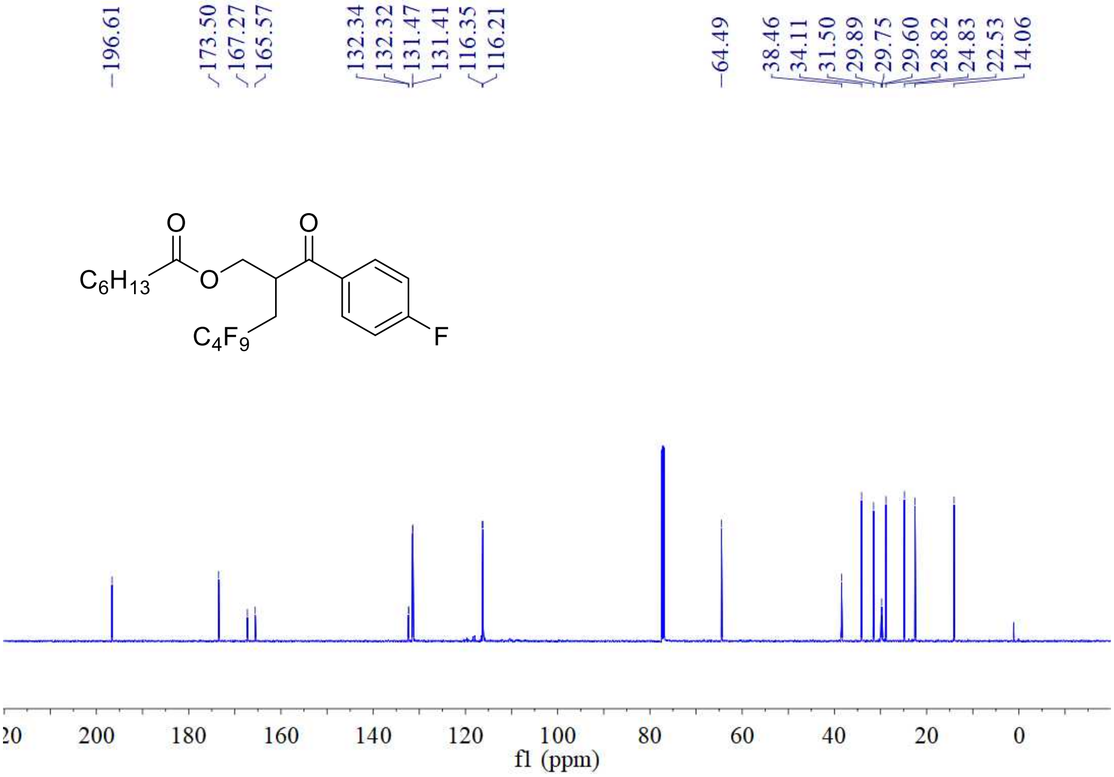

Supplementary Figure 49: NMR Spectra of 4,4,5,5,6,6,7,7,7-Nonafluoro

-2-(4-fluorobenzoyl)heptyl heptanoate (18)

^{1}H NMR (600 MHz, $CDCl_3$)

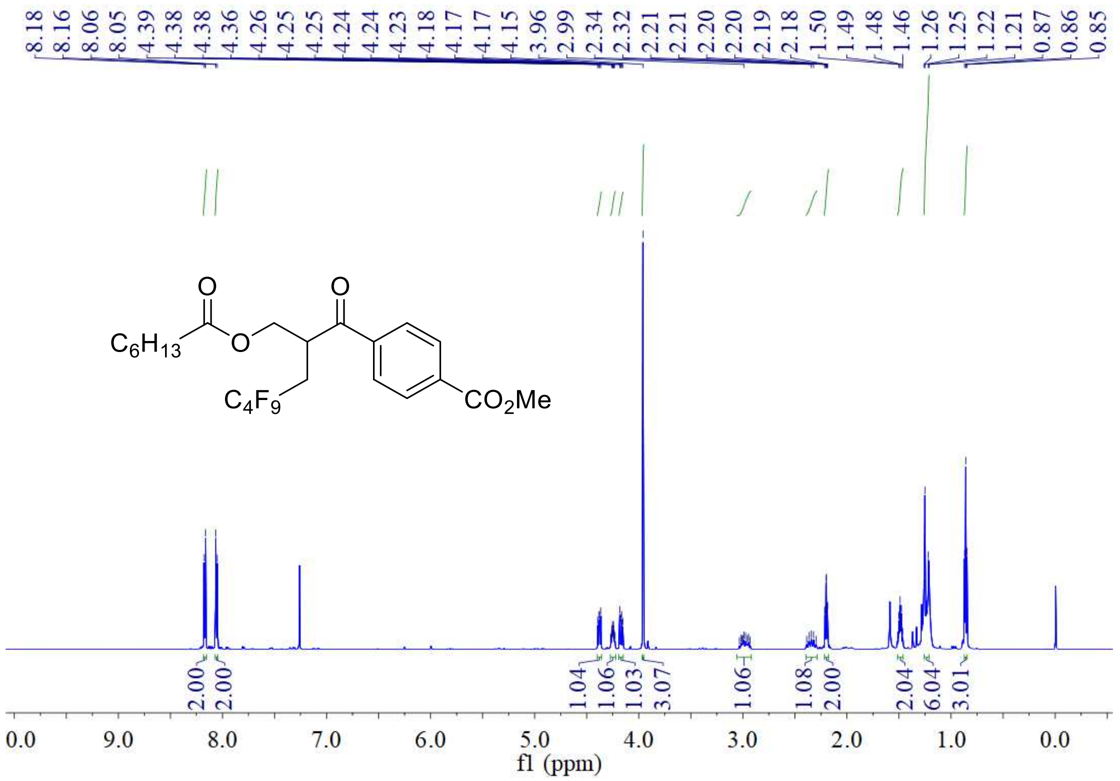

^{19}F NMR (564 MHz, $CDCl_3$)

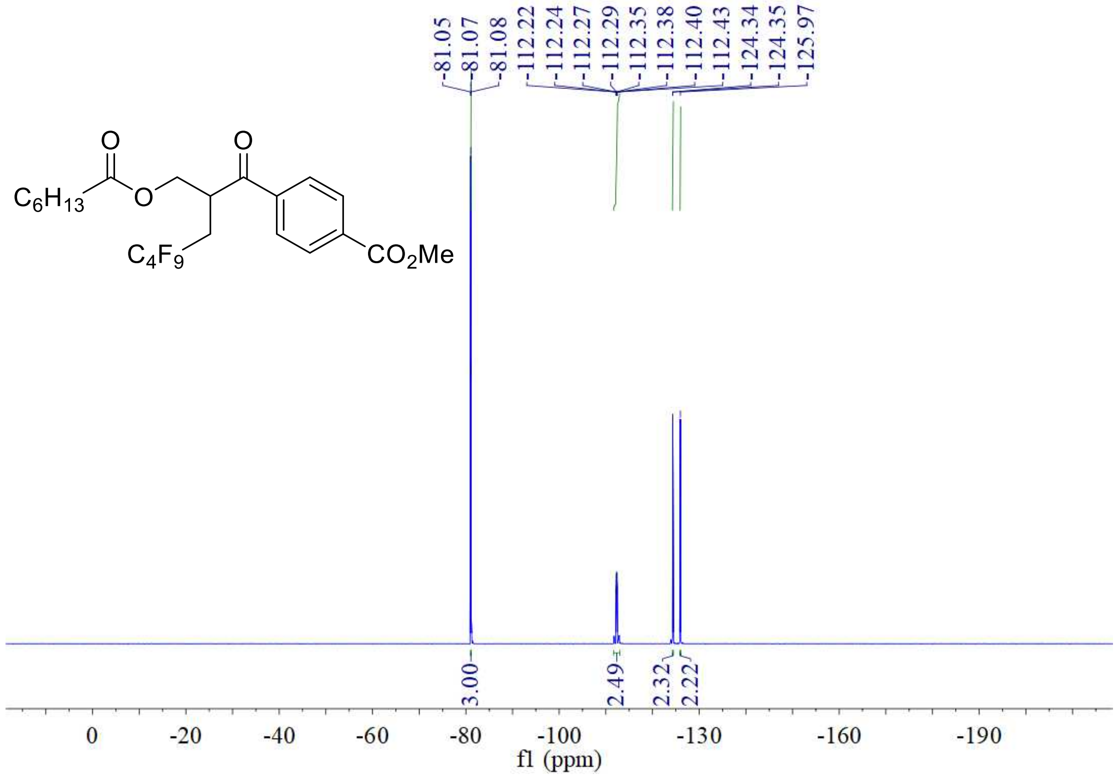

^{13}C NMR (150 MHz, $CDCl_3$)

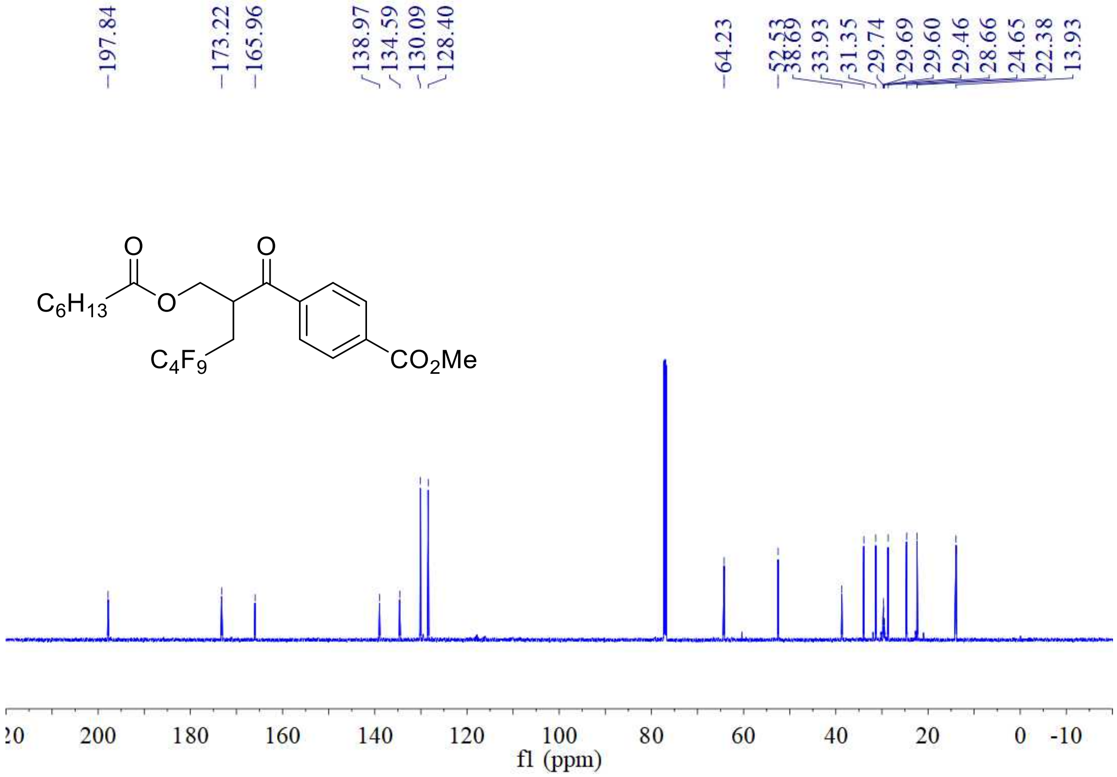

Supplementary Figure 50: NMR Spectra of Methyl 4-(4,4,5,5,6,6,7,7,7-nonafluoro-2-((heptanoyloxy)methyl)heptanoyl) Benzoate (19)

^{1}H NMR (600 MHz, $CDCl_3$)

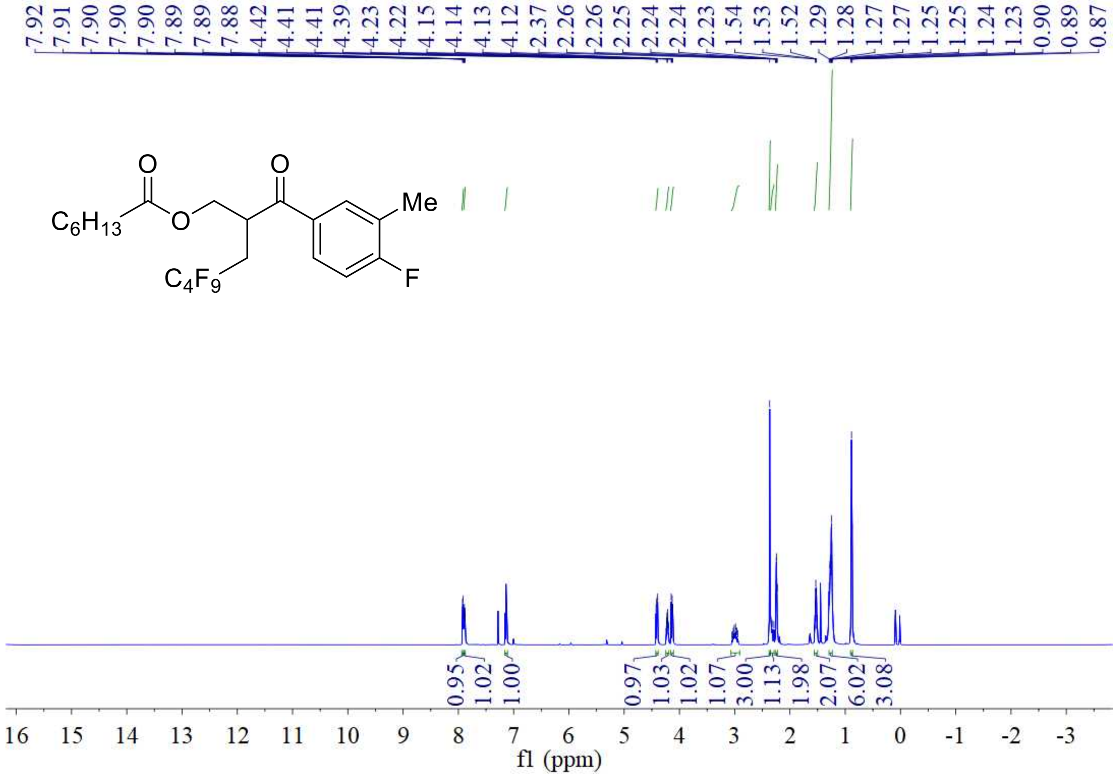

^{19}F NMR (564 MHz, $CDCl_3$)

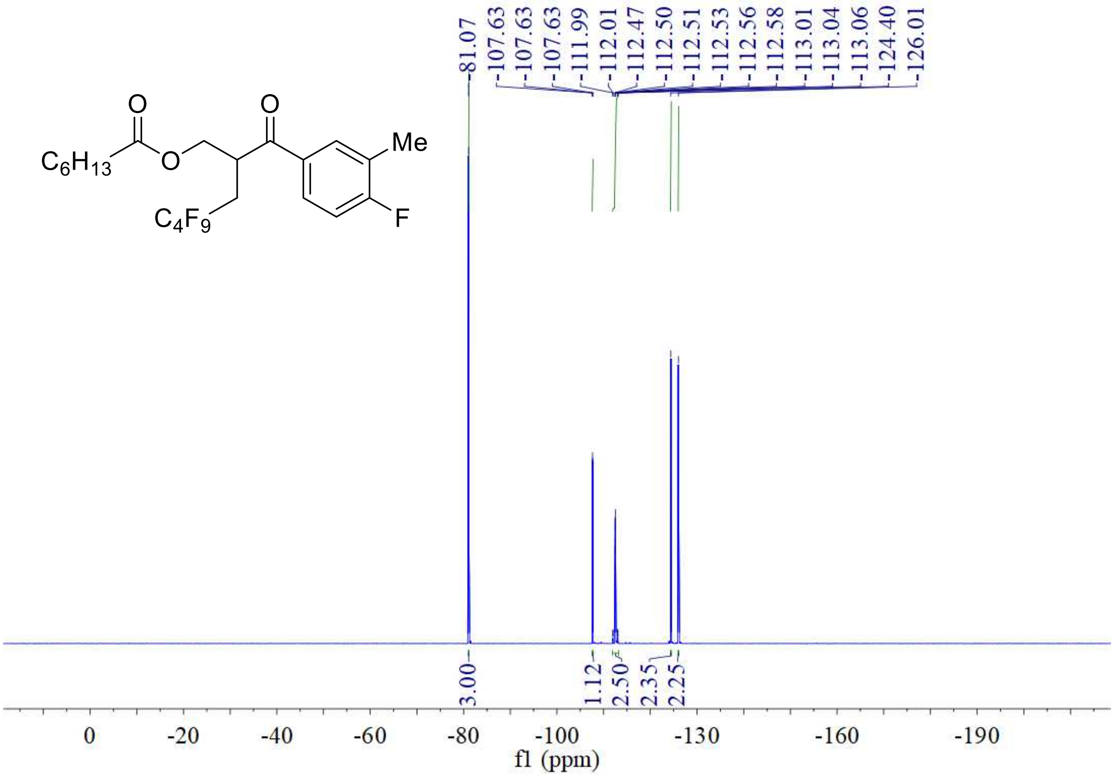

^{13}C NMR (150 MHz, $CDCl_3$)

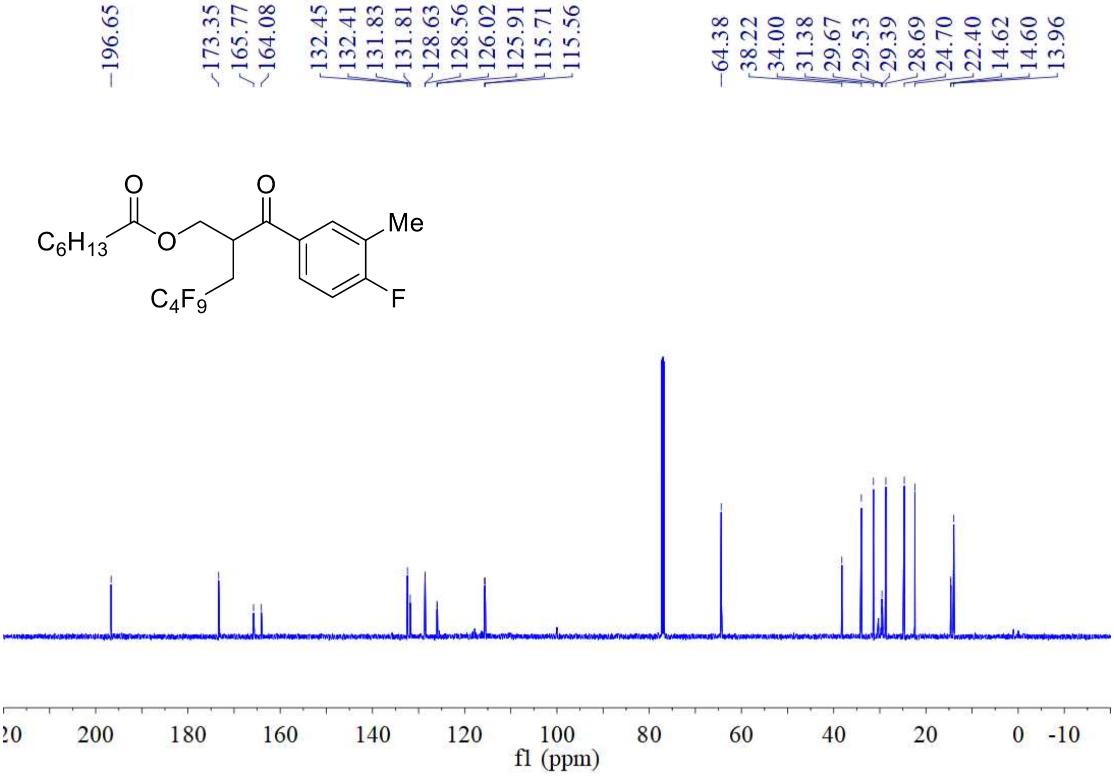

Supplementary Figure 51: NMR Spectra of 4,4,5,5,6,6,7,7,7

-Nonafluoro-2-(4-fluoro-3-methylbenzoyl)heptyl heptanoate (20)

^{1}H NMR (600 MHz, $CDCl_3$)

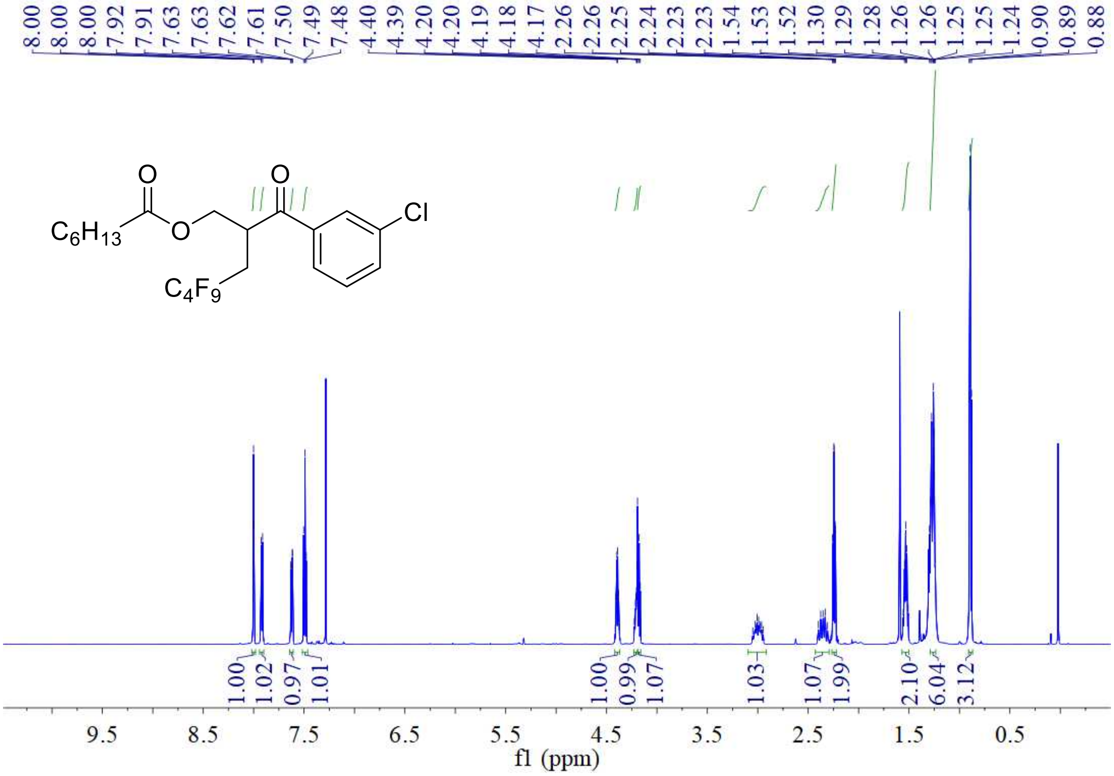

^{19}F NMR (376 MHz, $CDCl_3$)

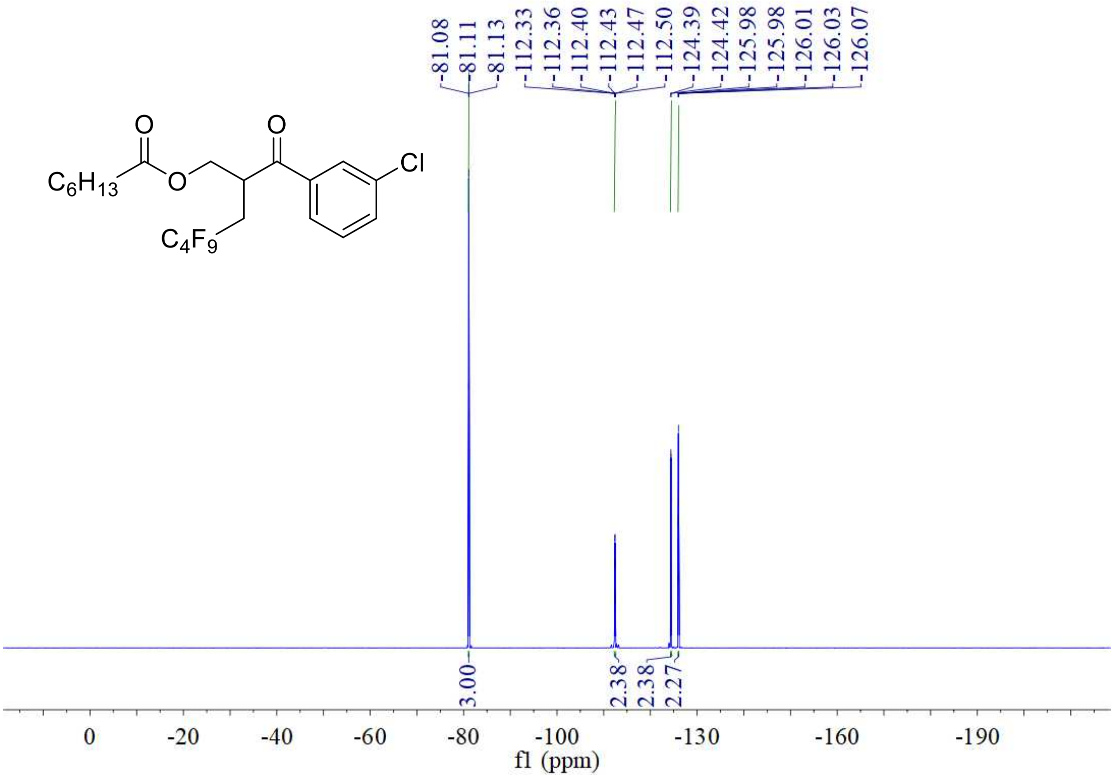

^{13}C NMR (150 MHz, $CDCl_3$)

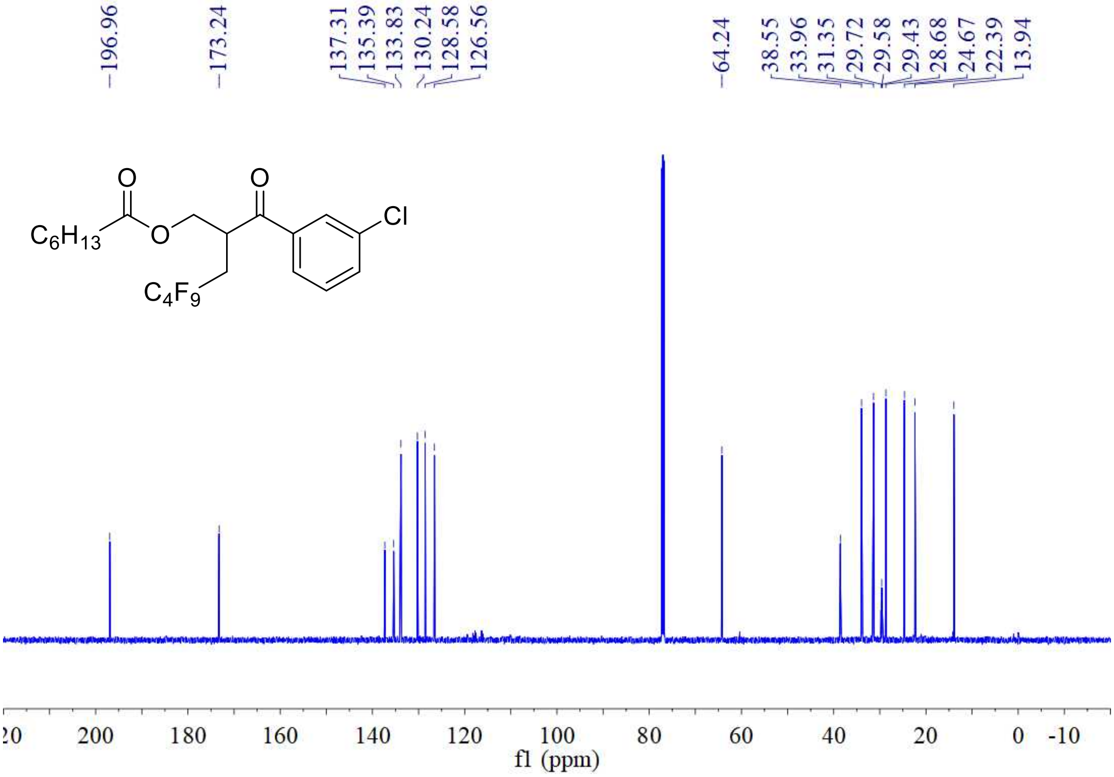

Supplementary Figure 52: NMR Spectra of 2-(3-Chlorobenzoyl)-4,4,5,5,6,6,7,7,7-nonafluoroheptyl heptanoate (21)

^{1}H NMR (600 MHz, $CDCl_3$)

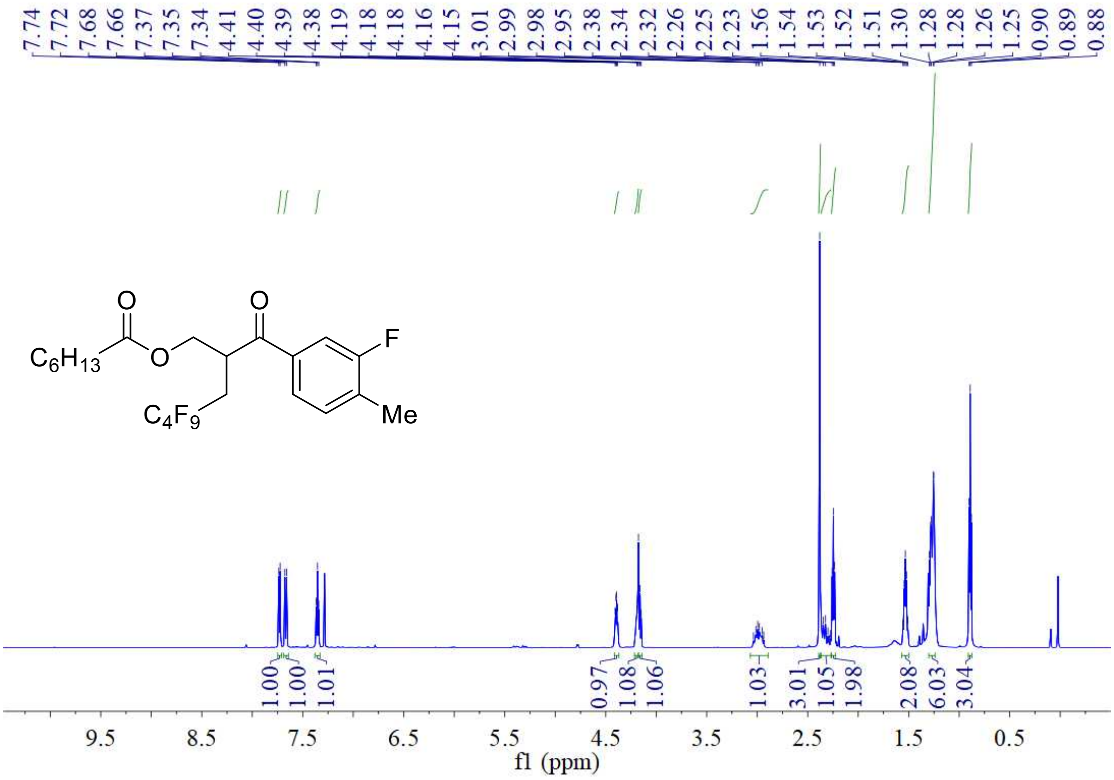

^{19}F NMR (564 MHz, $CDCl_3$)

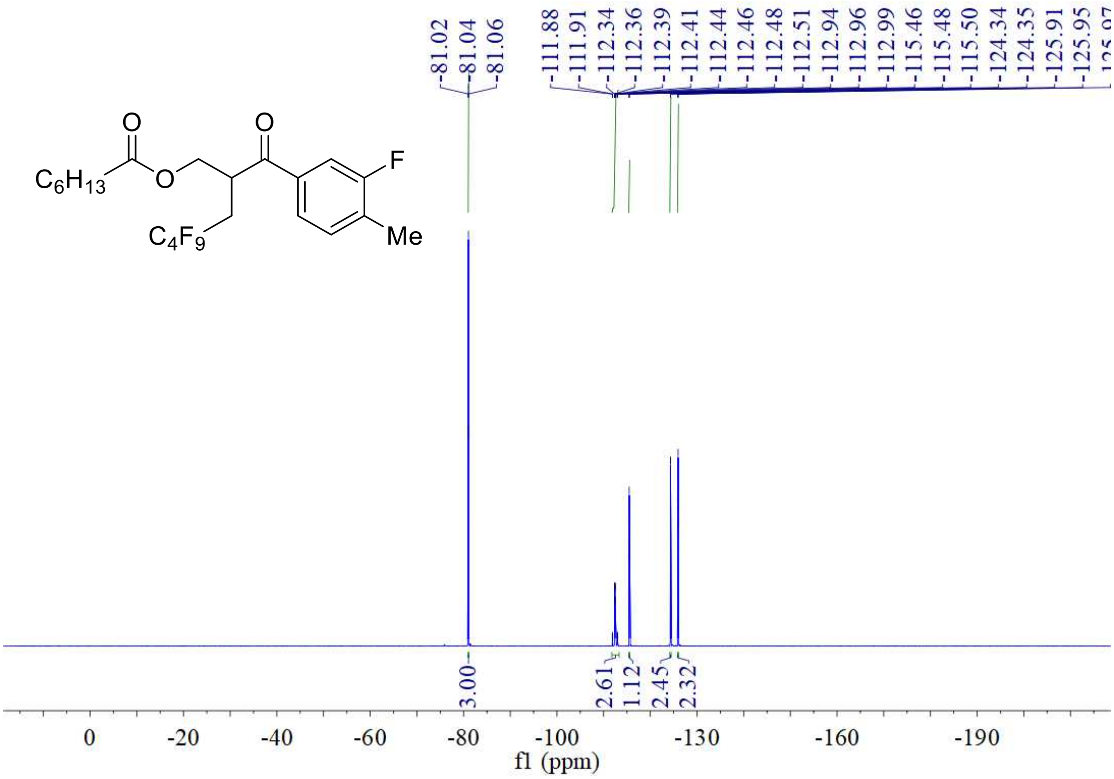

^{13}C NMR (150 MHz, $CDCl_3$)

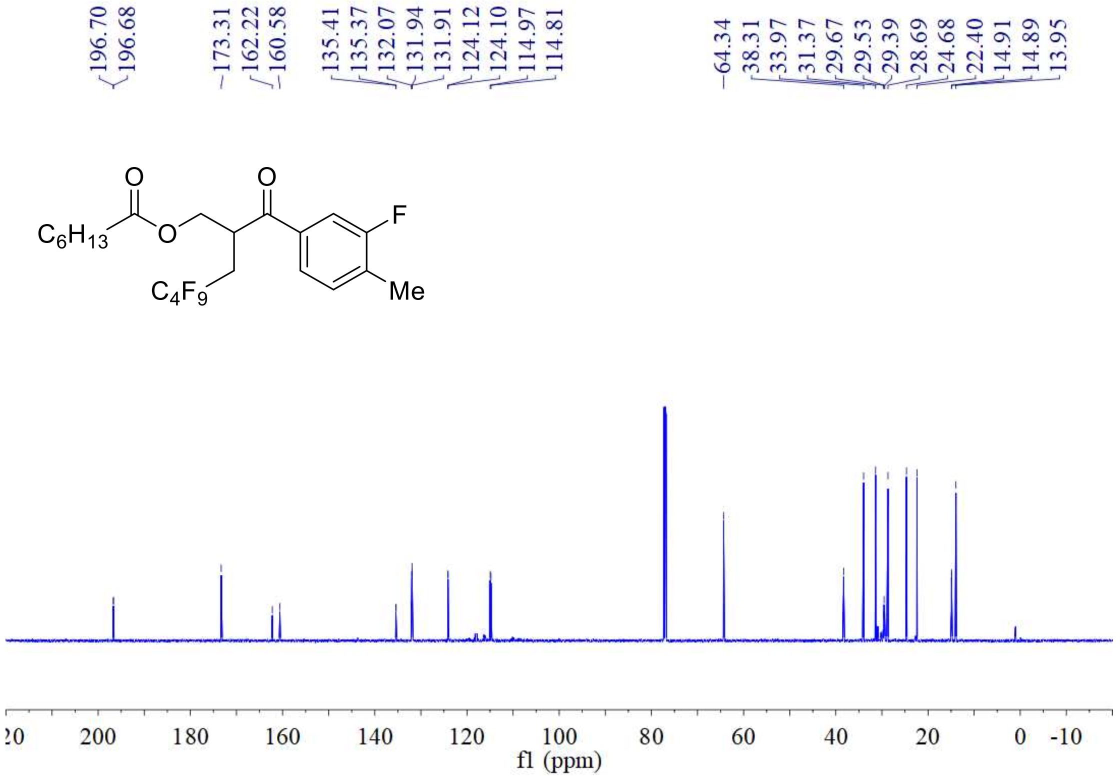

Supplementary Figure 53: NMR Spectra of 4,4,5,5,6,6,7,7,7-Nonafluoro -2-(3-fluoro-4-methylbenzoyl)heptyl heptanoate (22)

^{1}H NMR (600 MHz, $CDCl_3$)

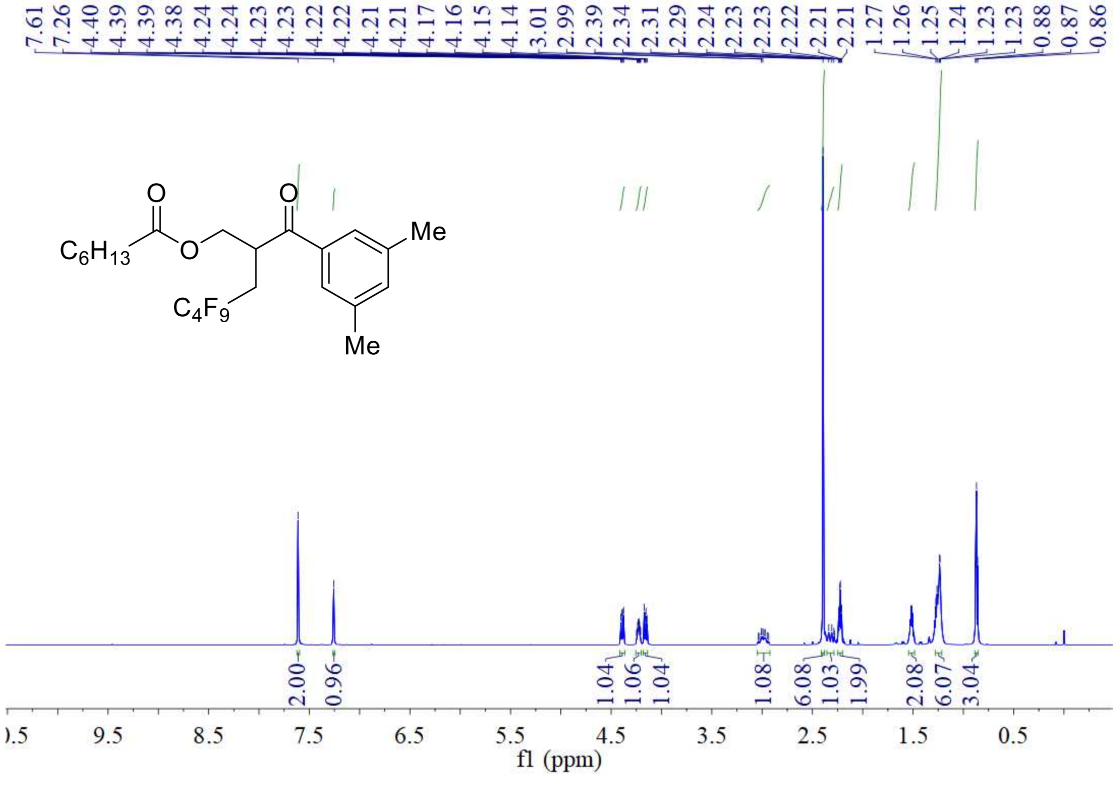

^{19}F NMR (564 MHz, $CDCl_3$)

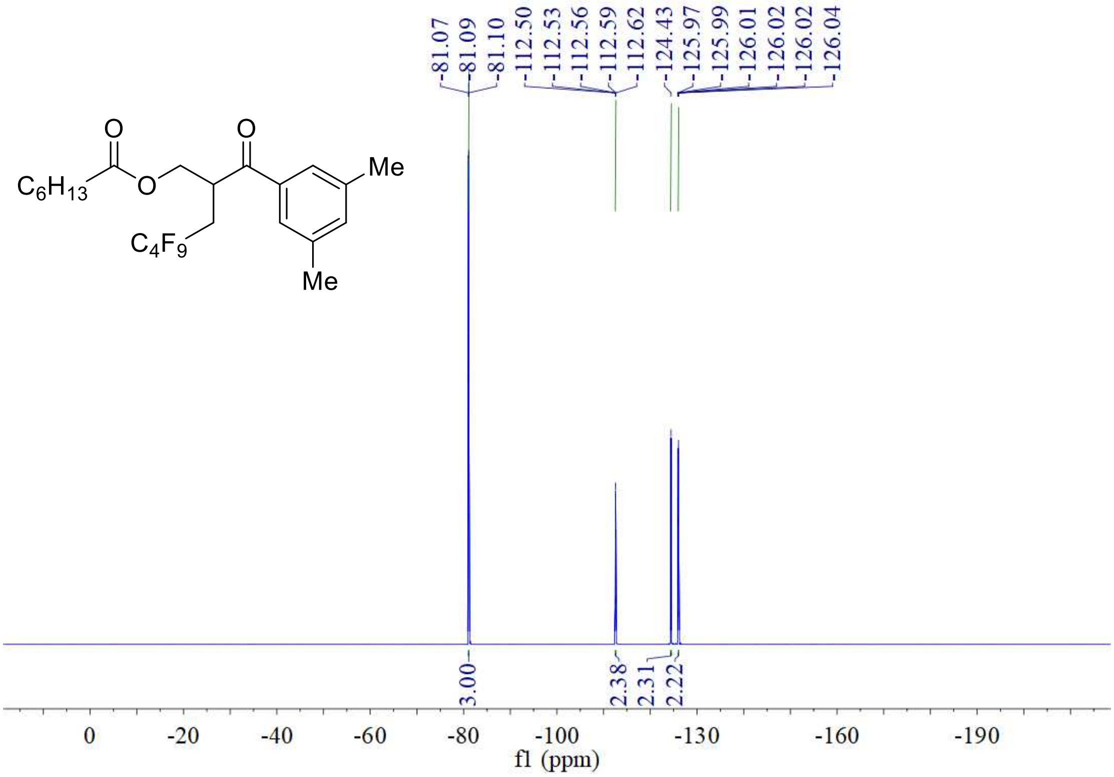

^{13}C NMR (150 MHz, $CDCl_3$)

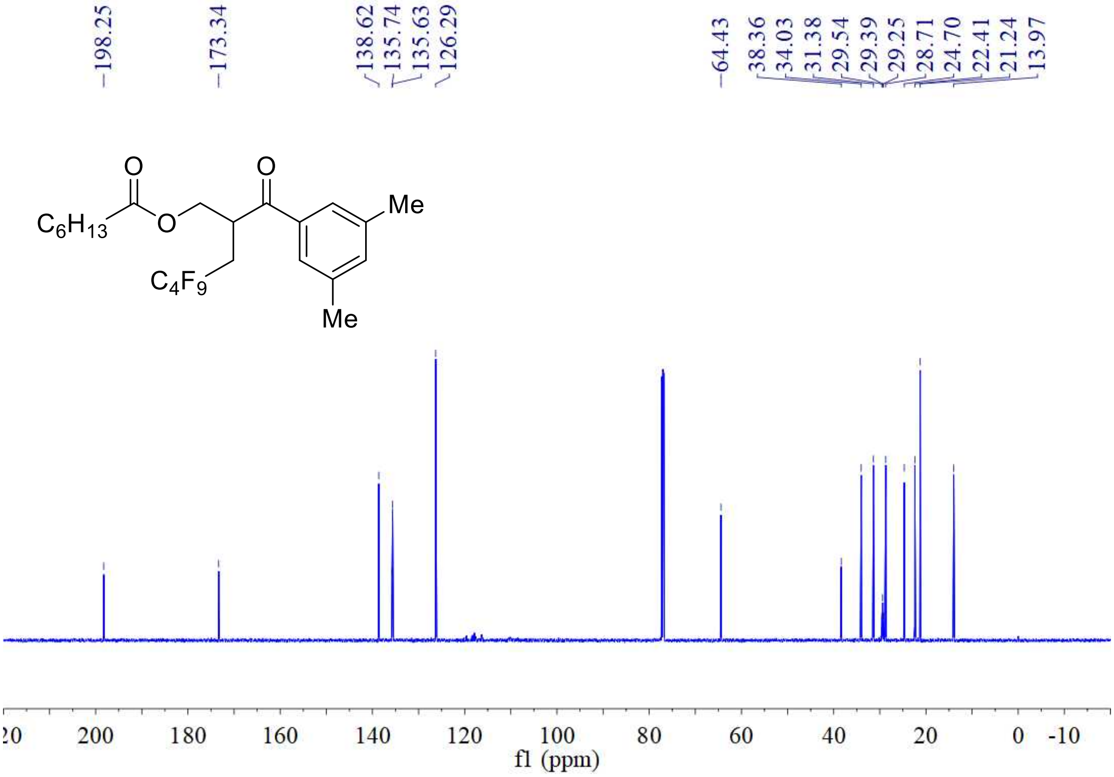

Supplementary Figure 54: NMR Spectra of 2-(3,5-Dimethylbenzoyl)-

4,4,5,5,6,6,7,7,7-nonafluoroheptyl heptanoate (23)

^{1}H NMR (600 MHz, $CDCl_3$)

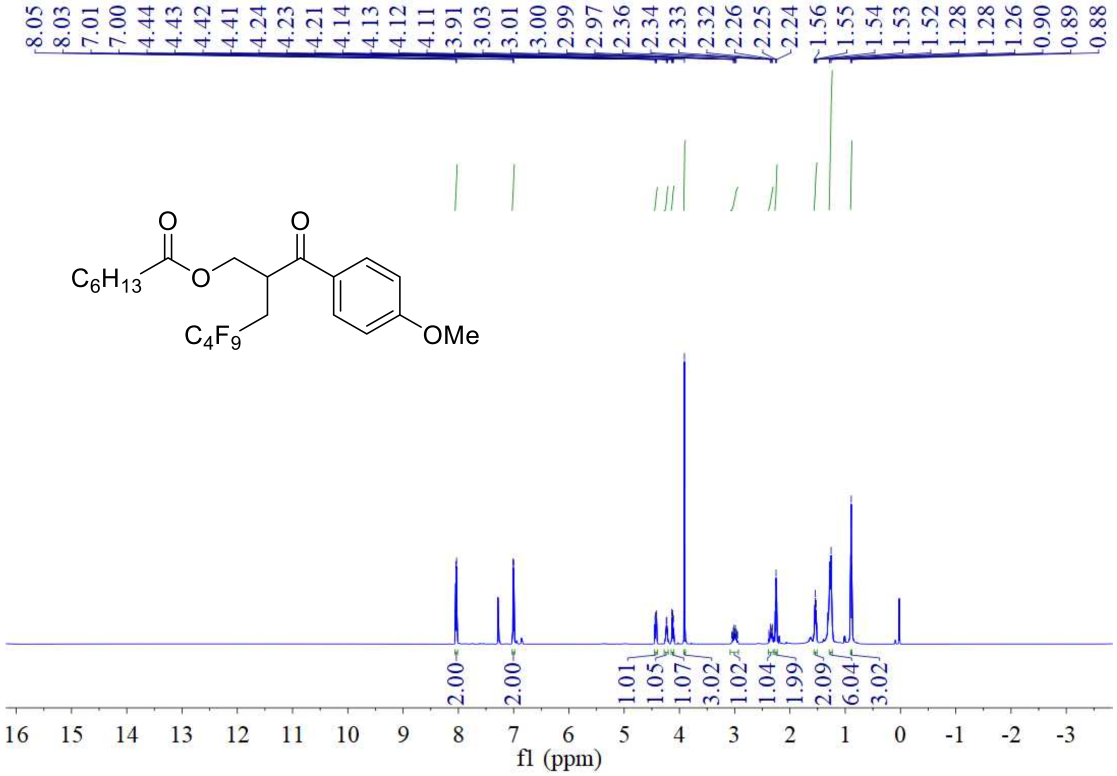

^{19}F NMR (564 MHz, $CDCl_3$)

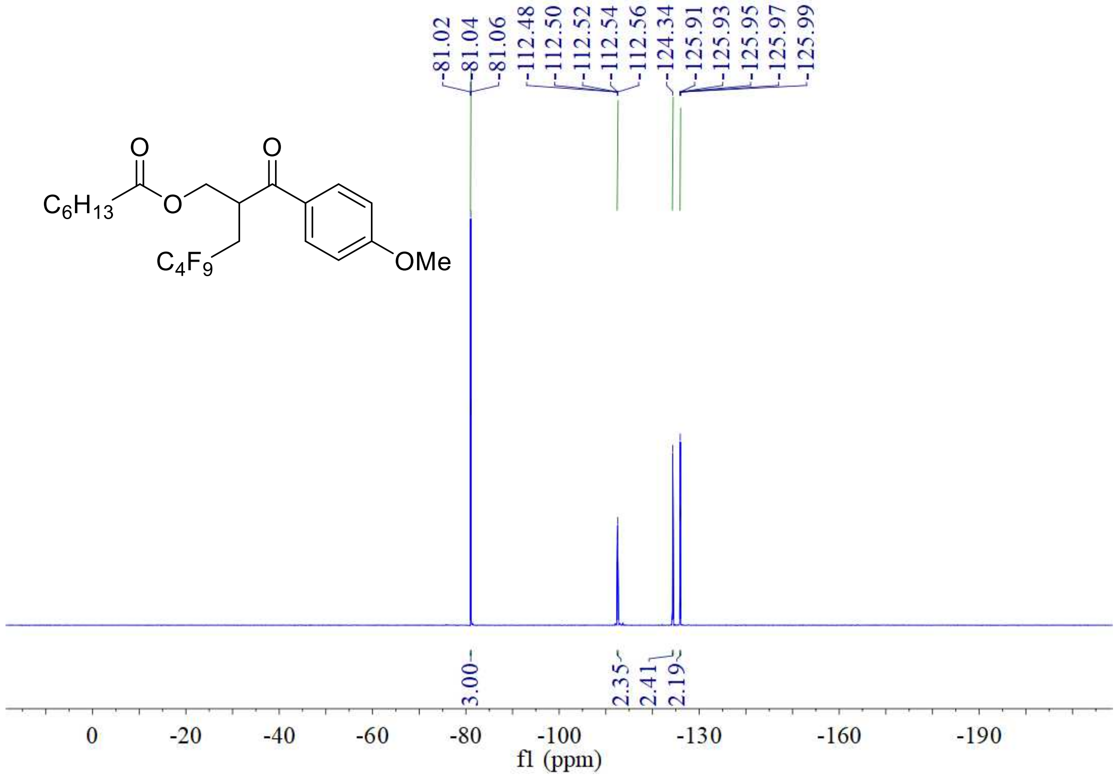

^{13}C NMR (150 MHz, $CDCl_3$)

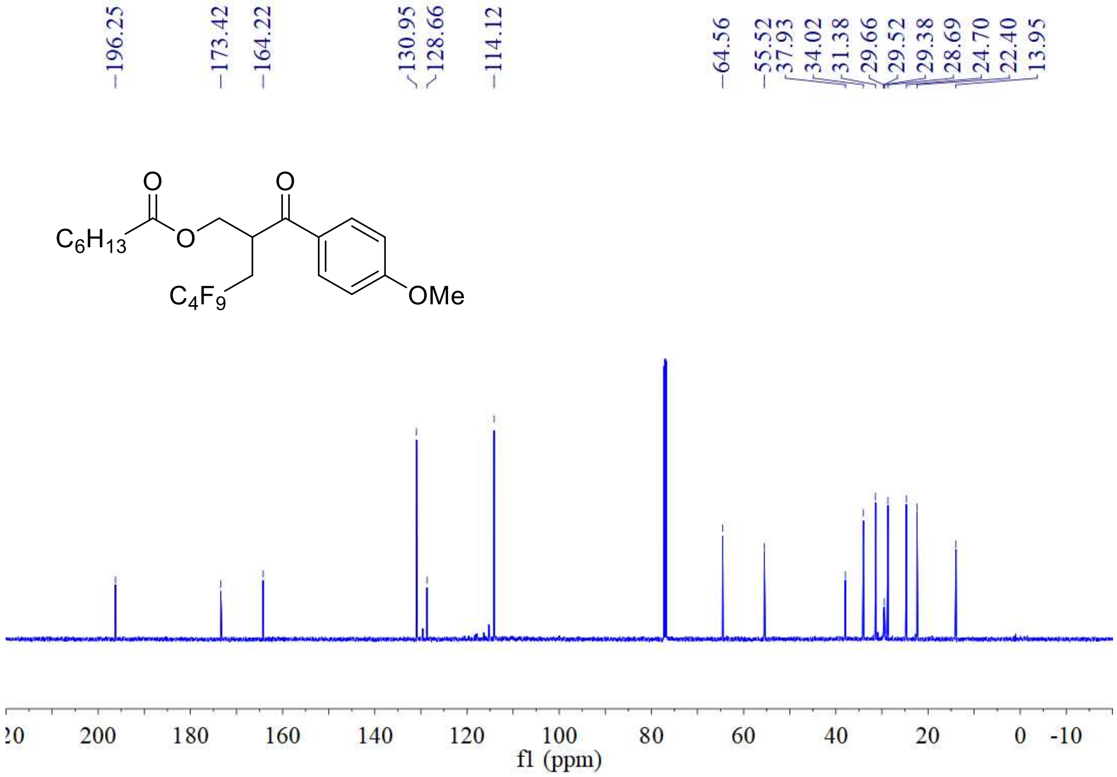

Supplementary Figure 55: NMR Spectra of 4,4,5,5,6,6,7,7,7-Nonafluoro -2-(4-methoxybenzoyl)heptyl heptanoate (24)

^{1}H NMR (600 MHz, $CDCl_3$)

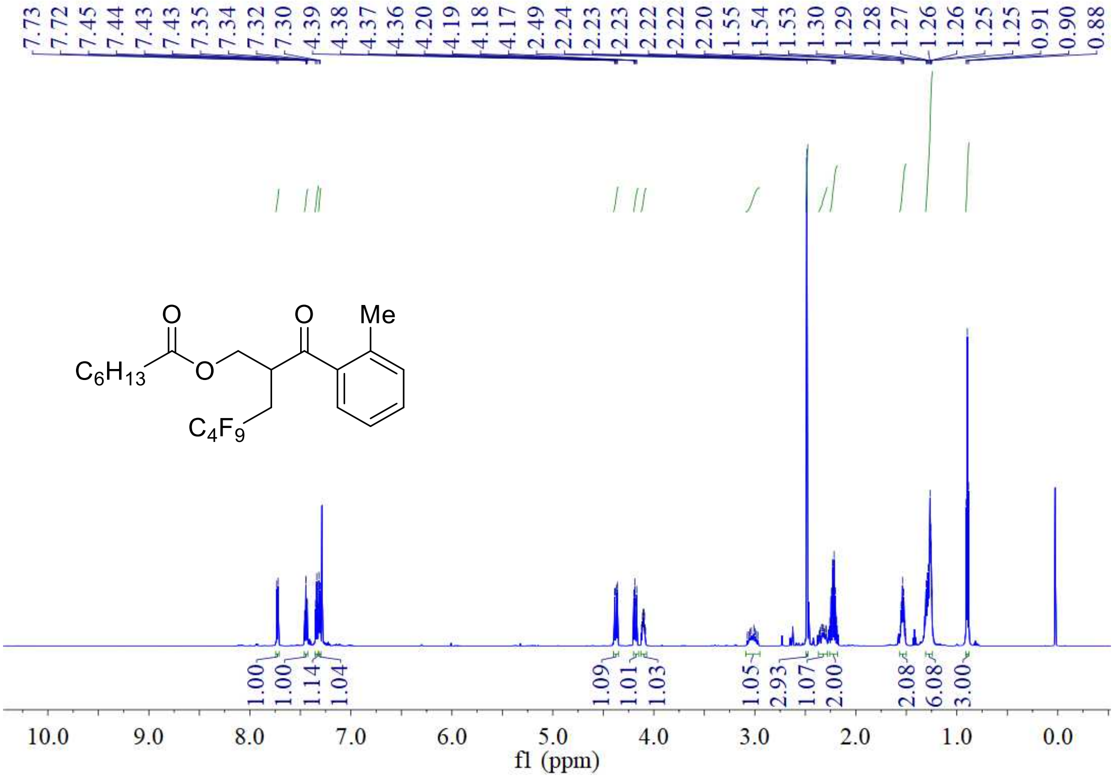

^{19}F NMR (564 MHz, $CDCl_3$)

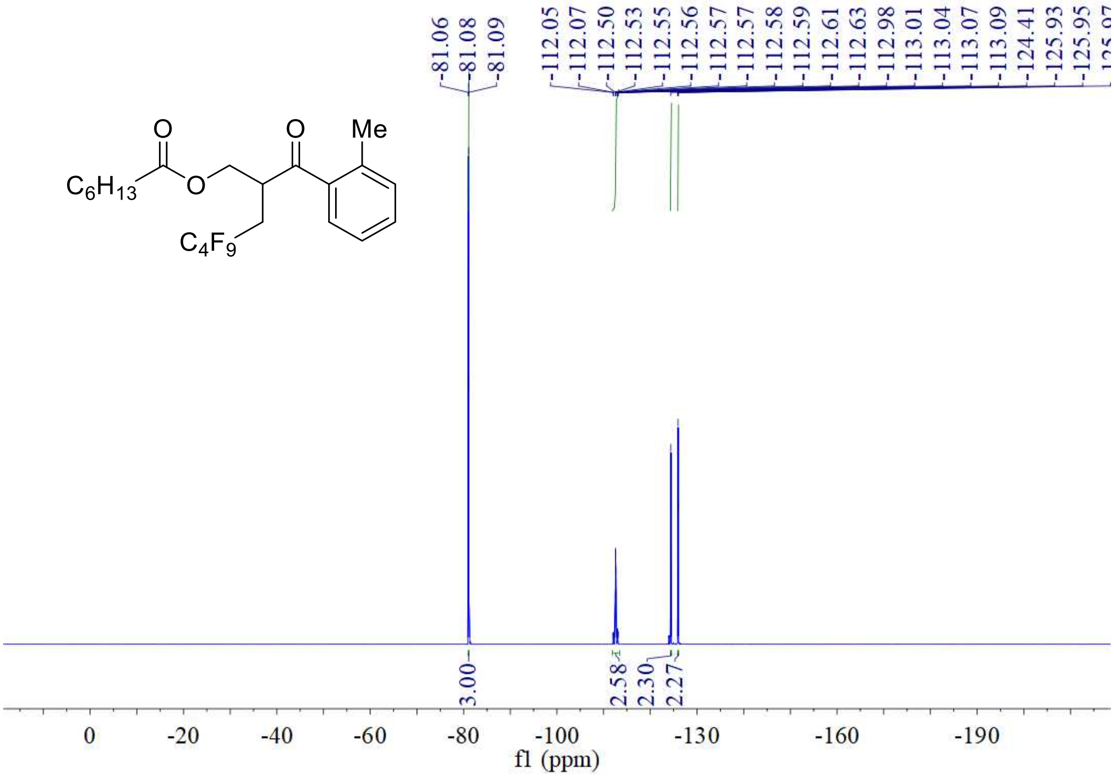

^{13}C NMR (150 MHz, $CDCl_3$)

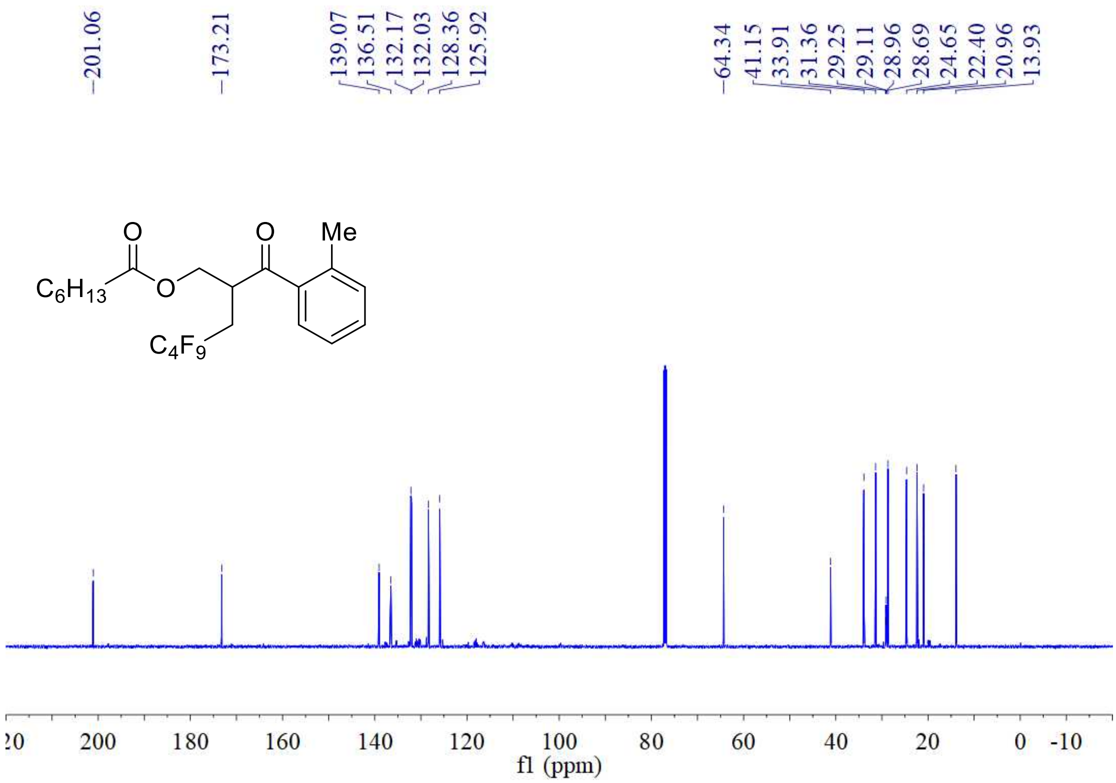

Supplementary Figure 56: NMR Spectra of 4,4,5,5,6,6,7,7,7-Nonafluoro-2-(2-methylbenzoyl)heptyl heptanoate (25)

^{1}H NMR (600 MHz, $CDCl_3$)

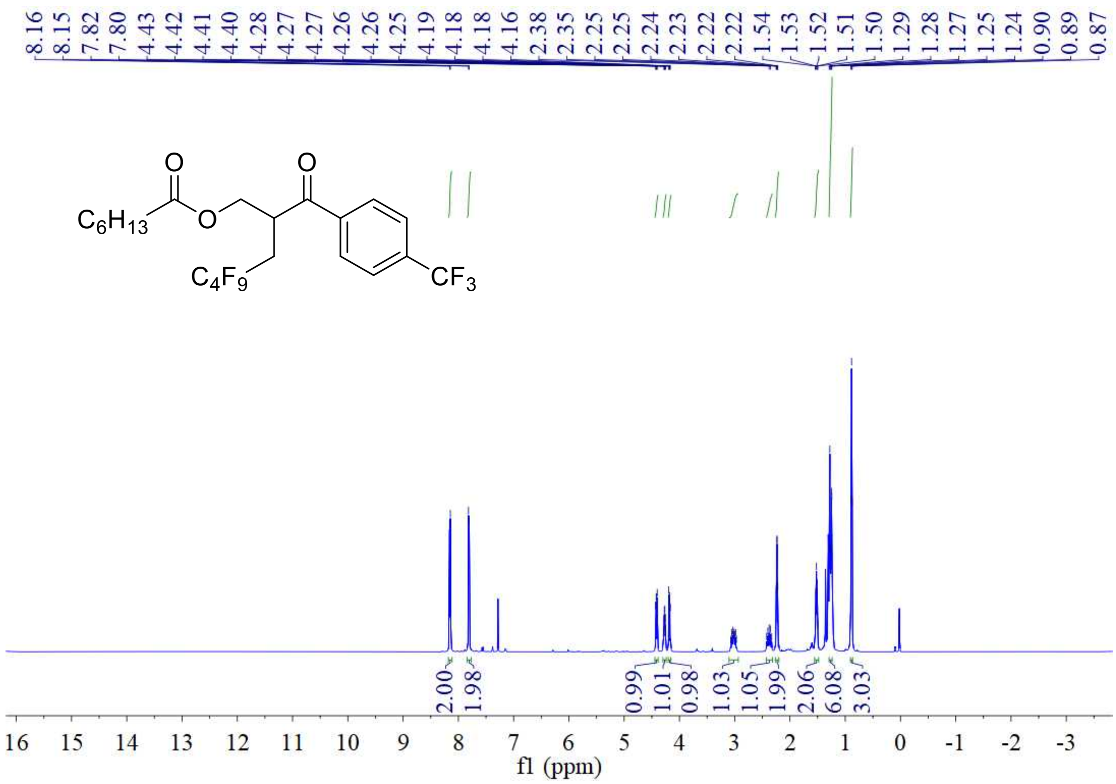

^{19}F NMR (564 MHz, $CDCl_3$)

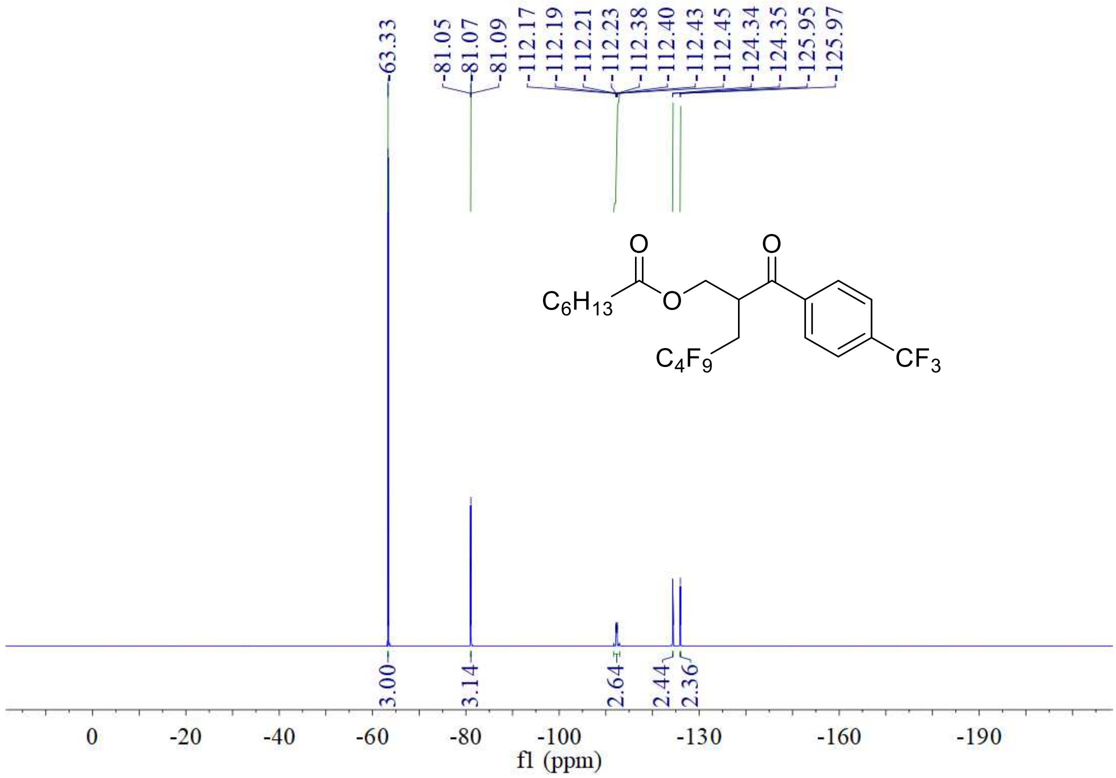

^{13}C NMR (150 MHz, $CDCl_3$)

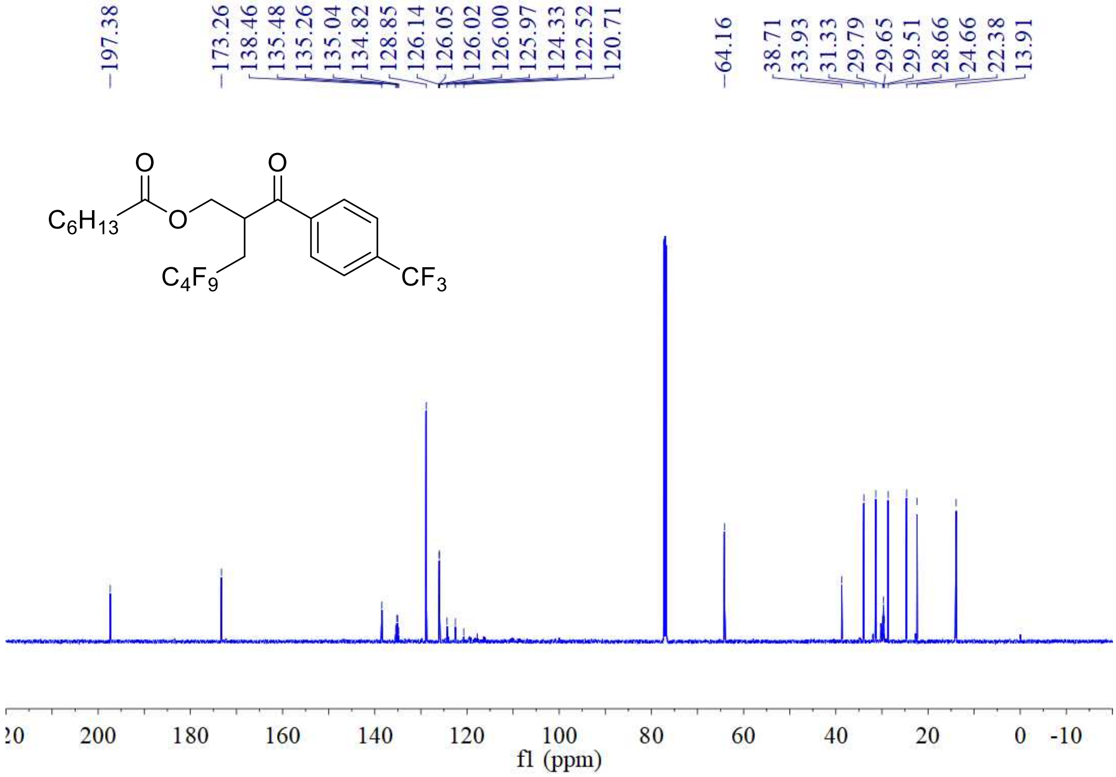

Supplementary Figure 57: NMR Spectra of 4,4,5,5,6,6,7,7,7-Nonafluoro-2-(4-(trifluoromethyl)benzoyl)heptyl heptanoate (26)

^{1}H NMR (600 MHz, $CDCl_3$)

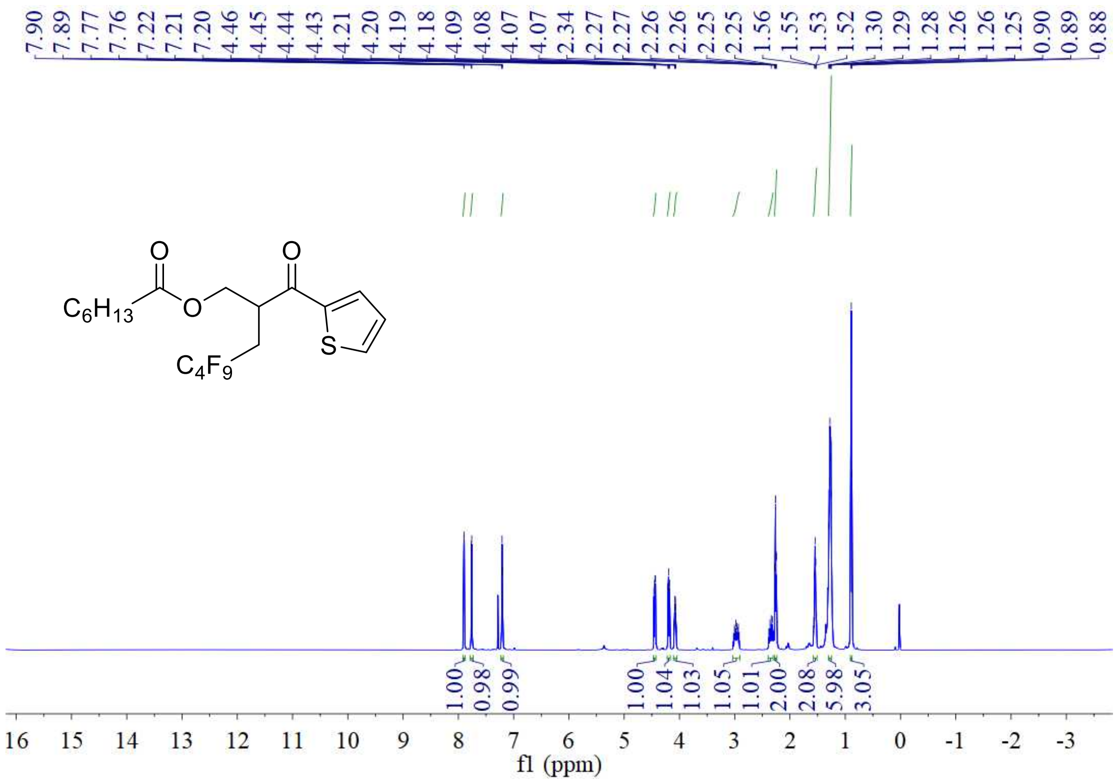

^{19}F NMR (564 MHz, $CDCl_3$)

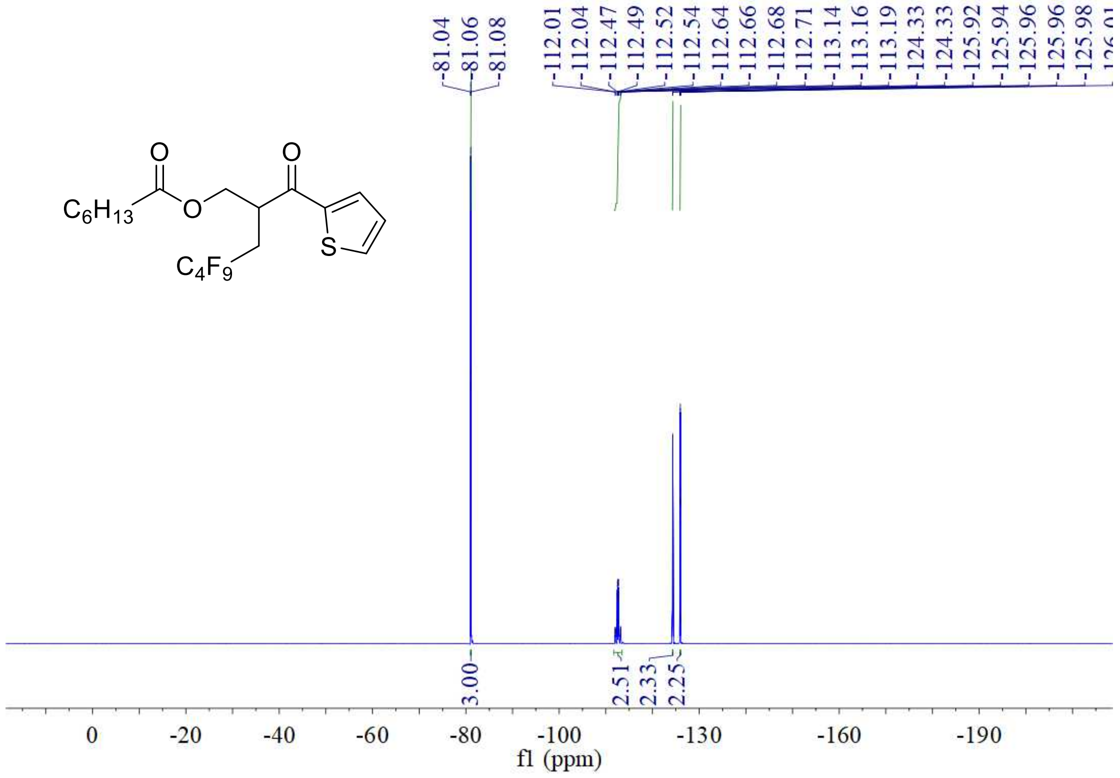

^{13}C NMR (150 MHz, $CDCl_3$)

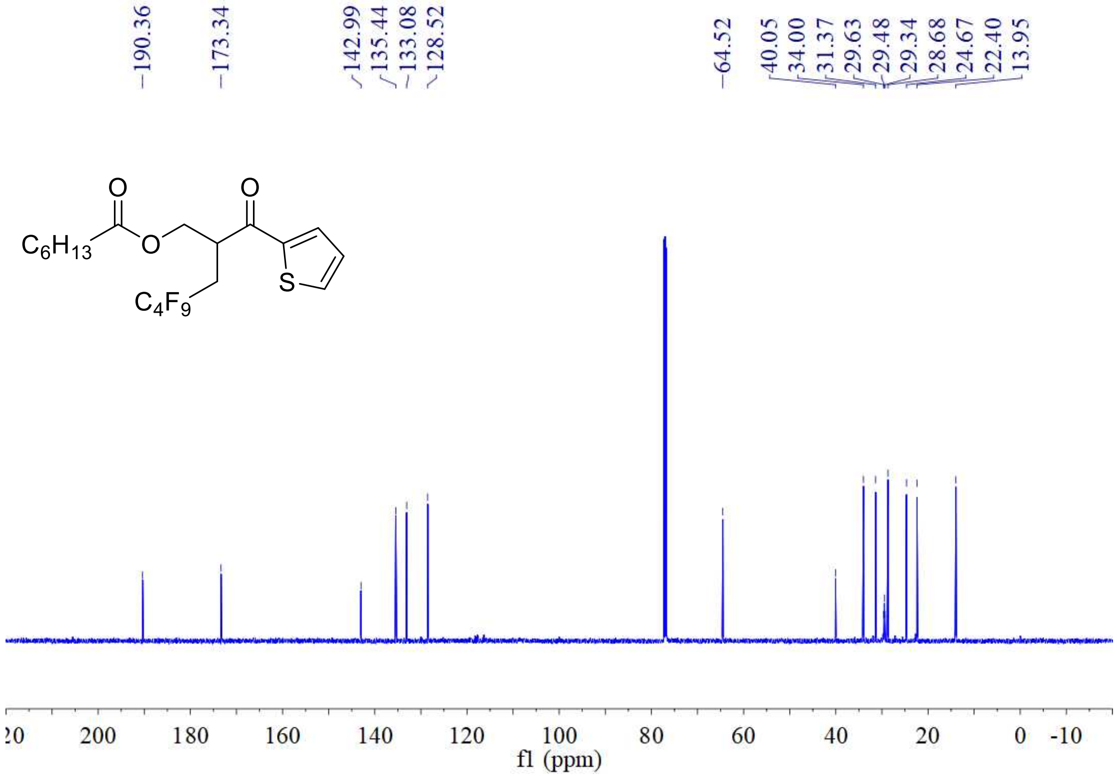

Supplementary Figure 58: NMR Spectra of 4,4,5,5,6,6,7,7,7-Nonafluoro-2-(thiophene-2-carbonyl)heptyl heptanoate (27)

^{1}H NMR (600 MHz, $CDCl_3$)

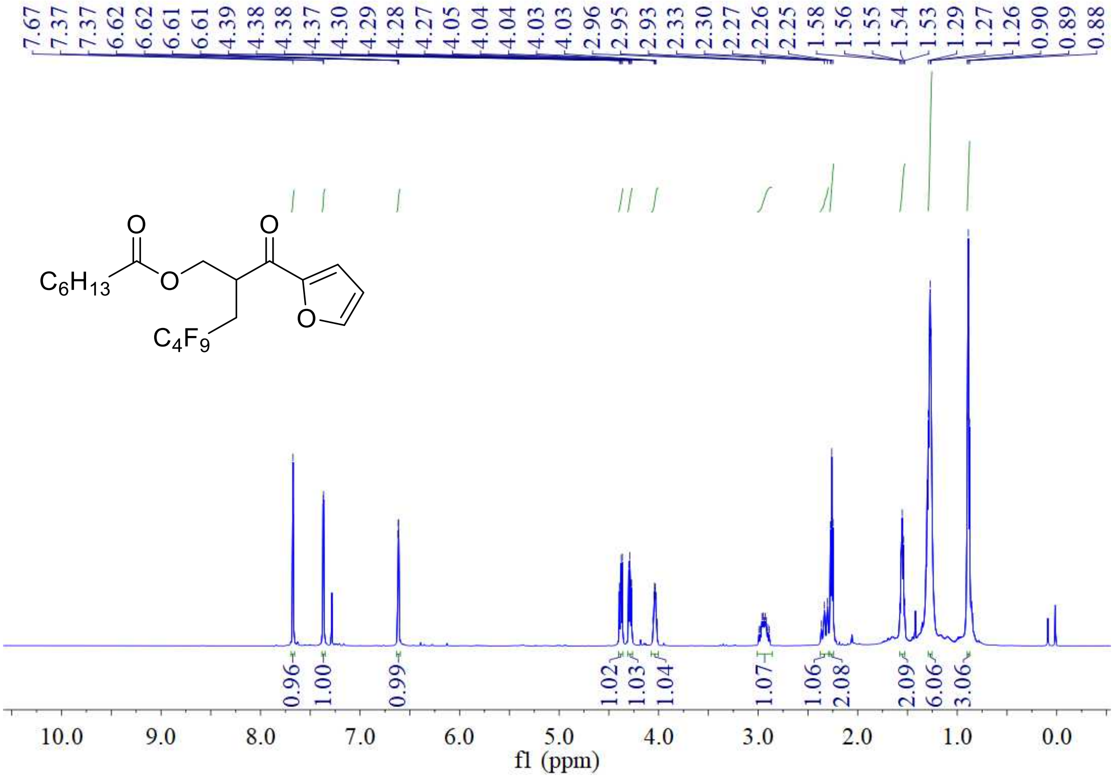

^{19}F NMR (564 MHz, $CDCl_3$)

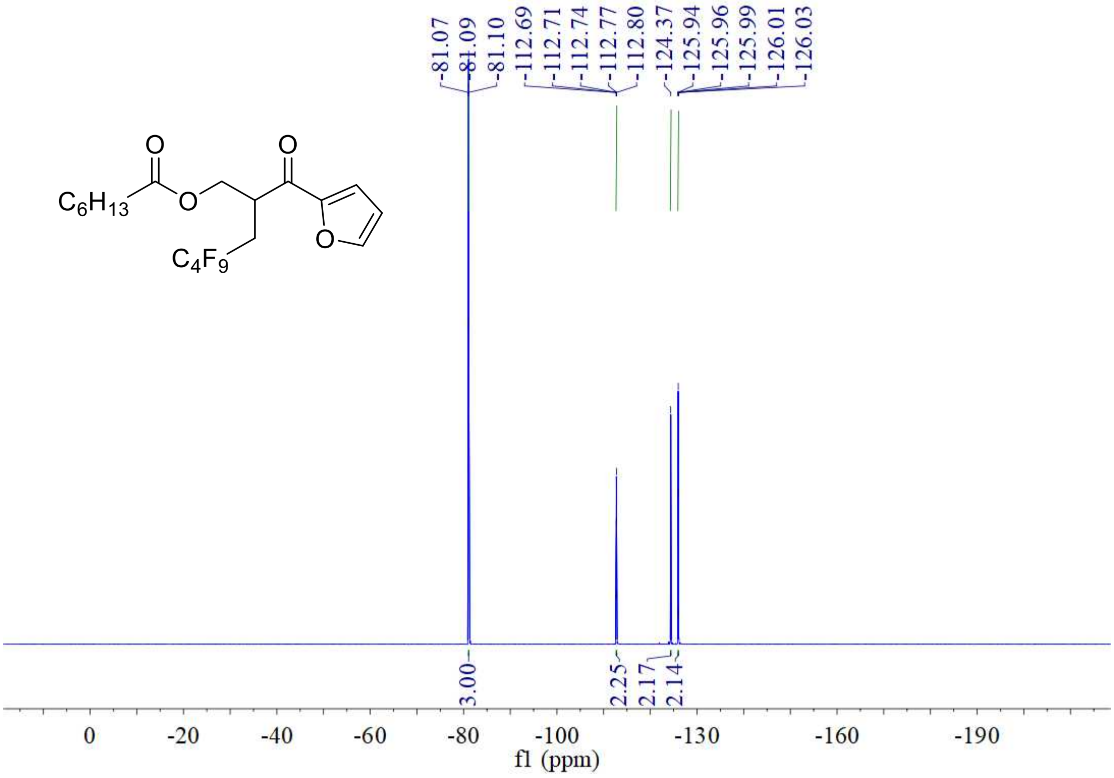

^{13}C NMR (150 MHz, $CDCl_3$)

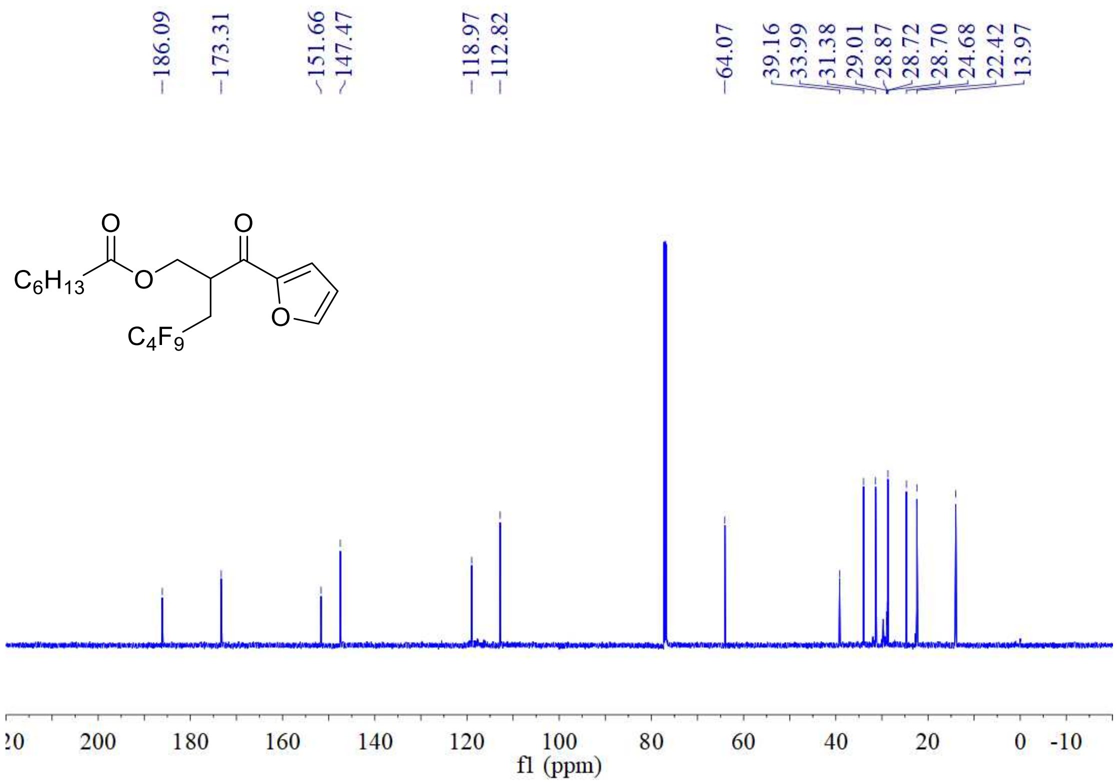

Supplementary Figure 59: NMR Spectra of 4,4,5,5,6,6,7,7,7-Nonafluoro-2-(furan-2-carbonyl)heptyl heptanoate (28)

^{1}H NMR (600 MHz, $CDCl_3$)

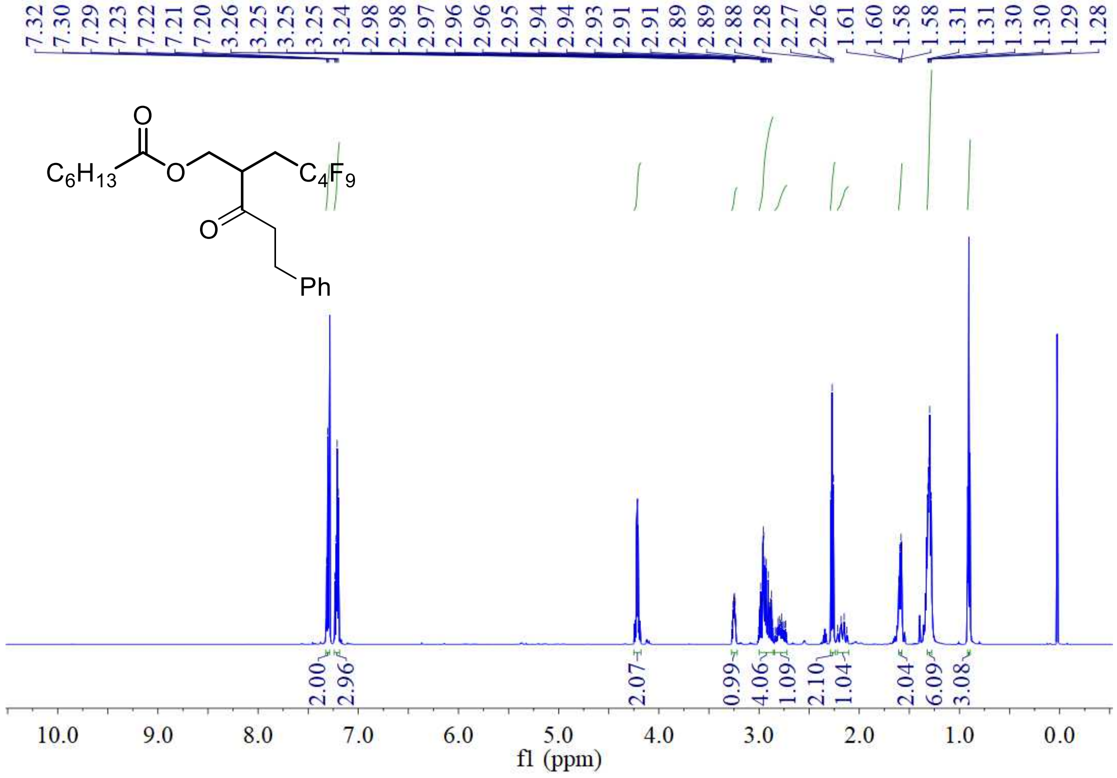

^{19}F NMR (564 MHz, $CDCl_3$)

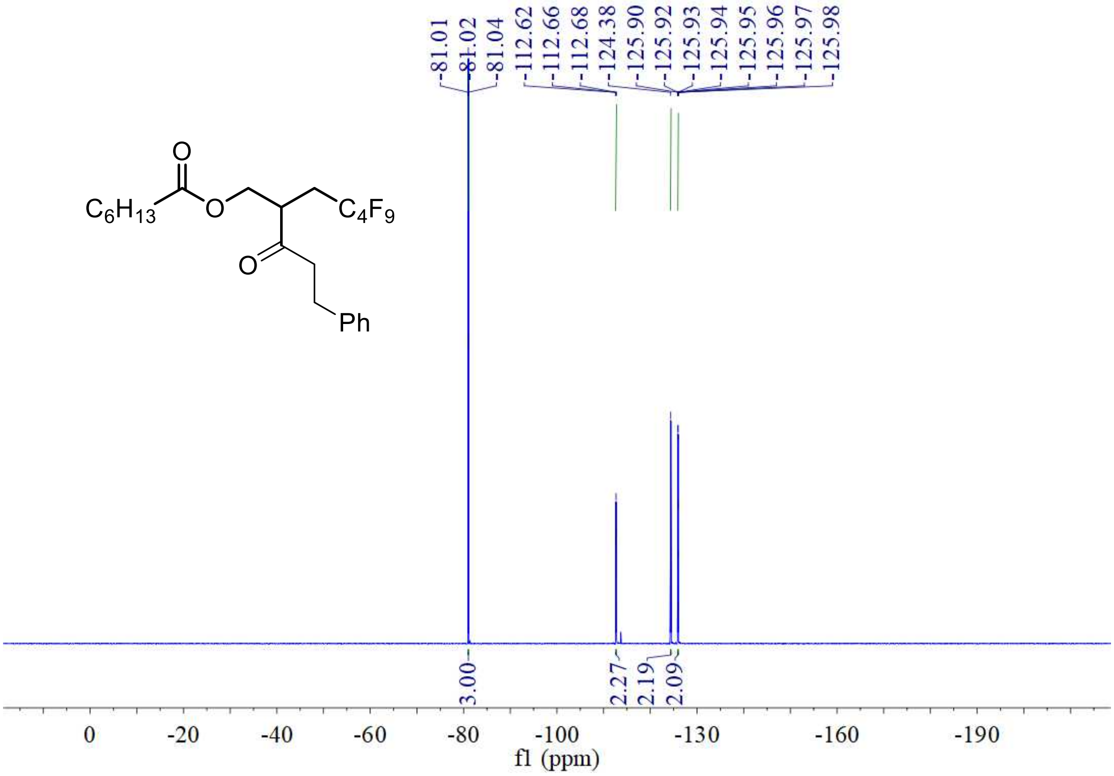

^{13}C NMR (150 MHz, $CDCl_3$)

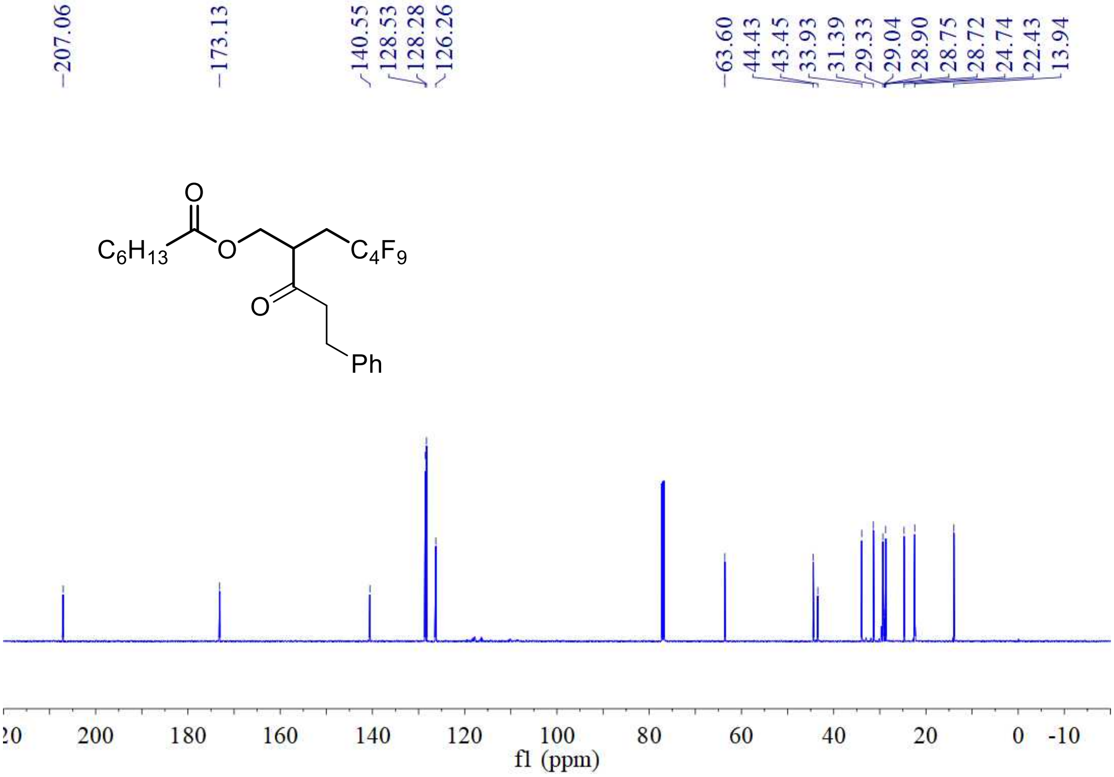

Supplementary Figure 60: NMR Spectra of 4,4,5,5,6,6,7,7,7-Nonafluoro-2-(3-phenylpropanoyl)heptyl heptanoate (29)

^{1}H NMR (600 MHz, $CDCl_3$)

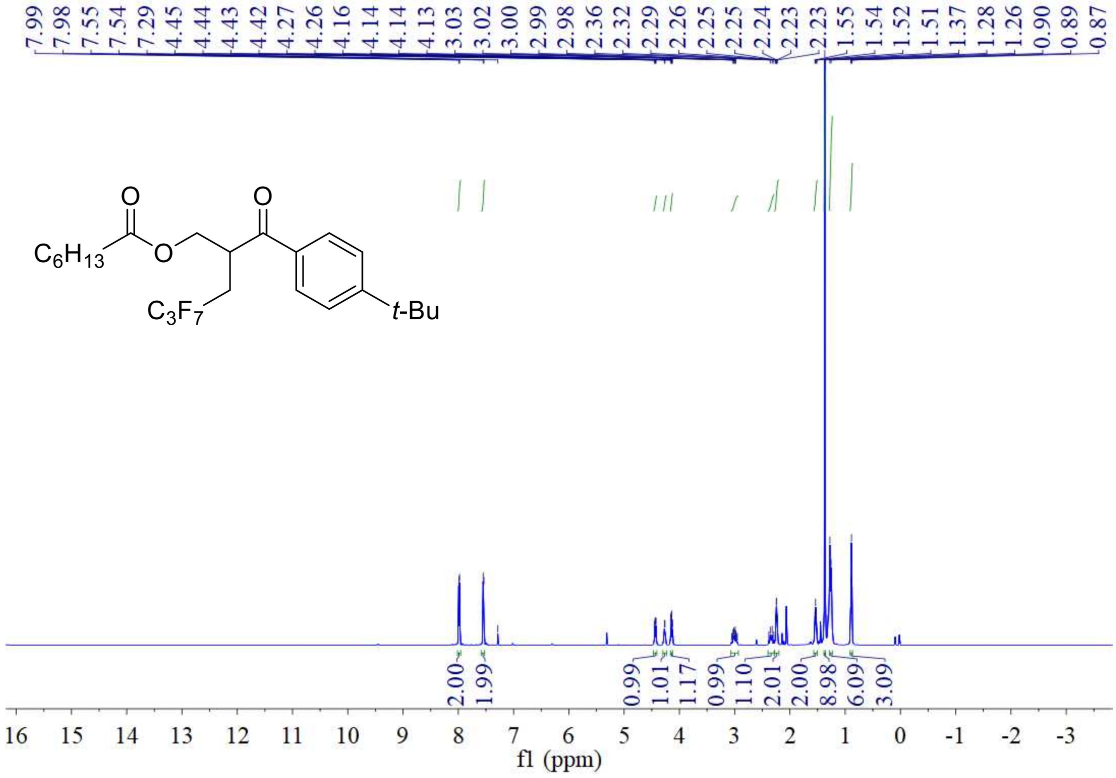

^{19}F NMR (376 MHz, $CDCl_3$)

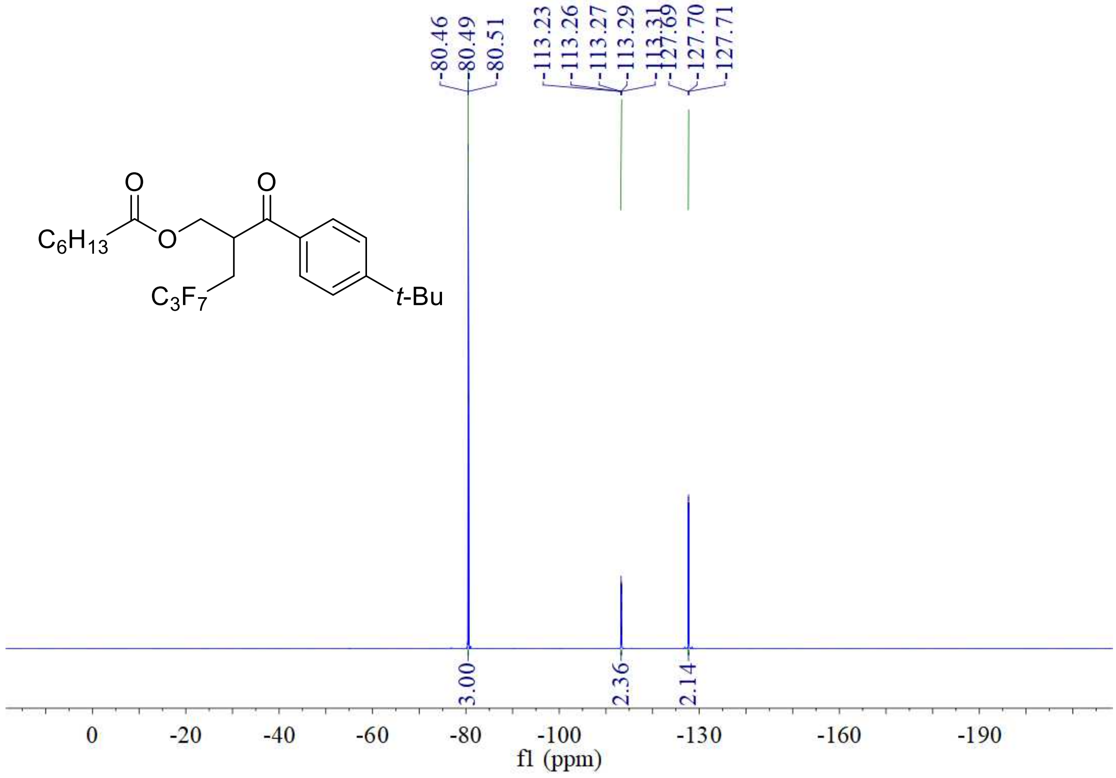

^{13}C NMR (150 MHz, $CDCl_3$)

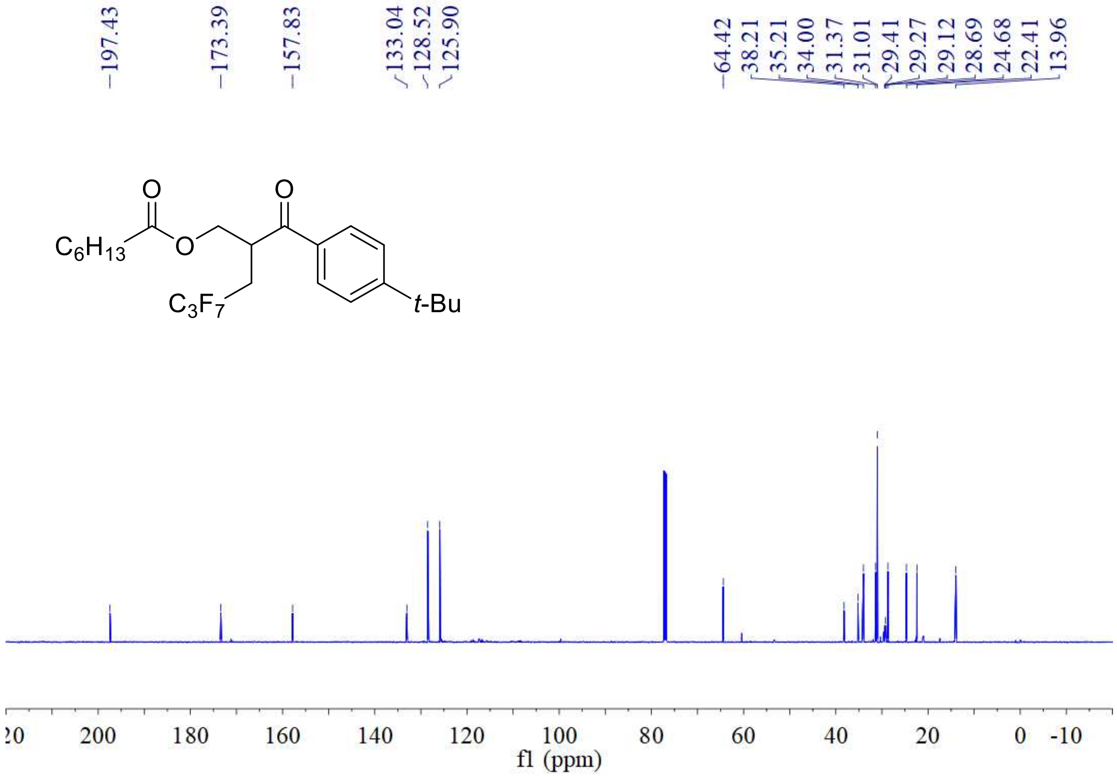

Supplementary Figure 61: NMR Spectra of 2-(4-(*tert*-Butyl)benzoyl)-4,4,5,5,6,6,6-heptafluorohexyl heptanoate (30)

^{1}H NMR (600 MHz, $CDCl_3$)

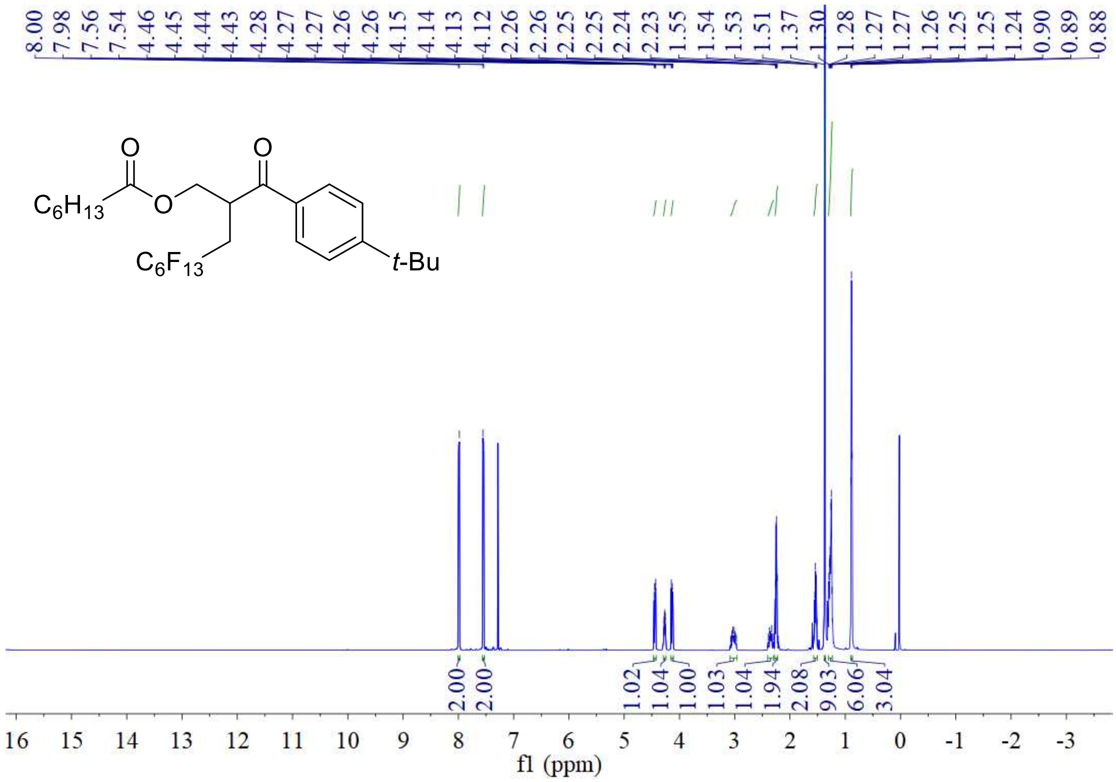

^{19}F NMR (564 MHz, $CDCl_3$)

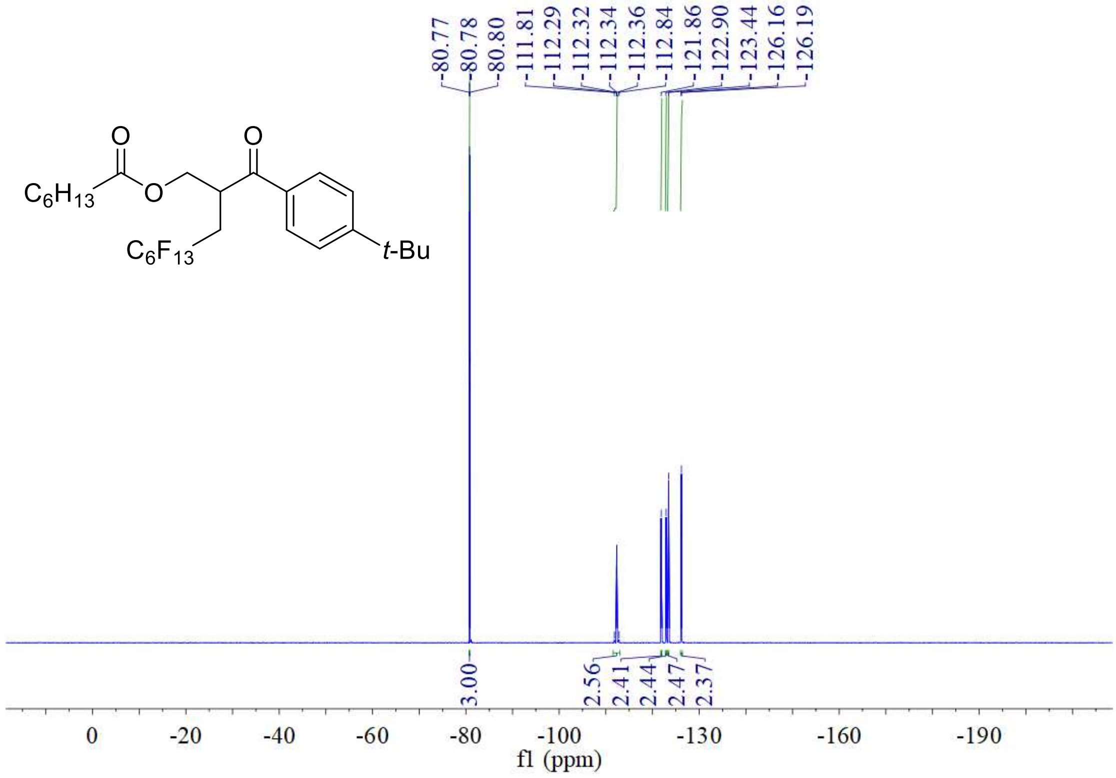

^{13}C NMR (150 MHz, $CDCl_3$)

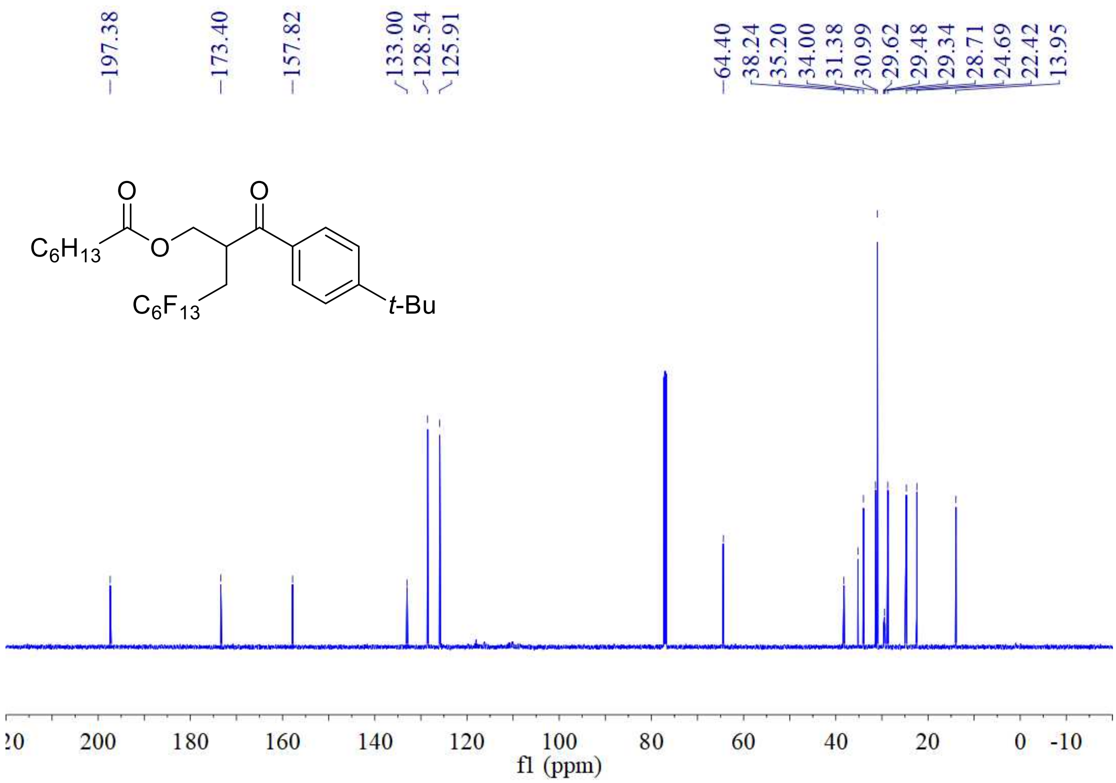

Supplementary Figure 62: NMR Spectra of 2-(4-(*tert*-Butyl)benzoyl)-4,4,5,5,6,6,7,7,8,8,9,9,9-tridecafluorononyl heptanoate (31)

^{1}H NMR (600 MHz, $CDCl_3$)

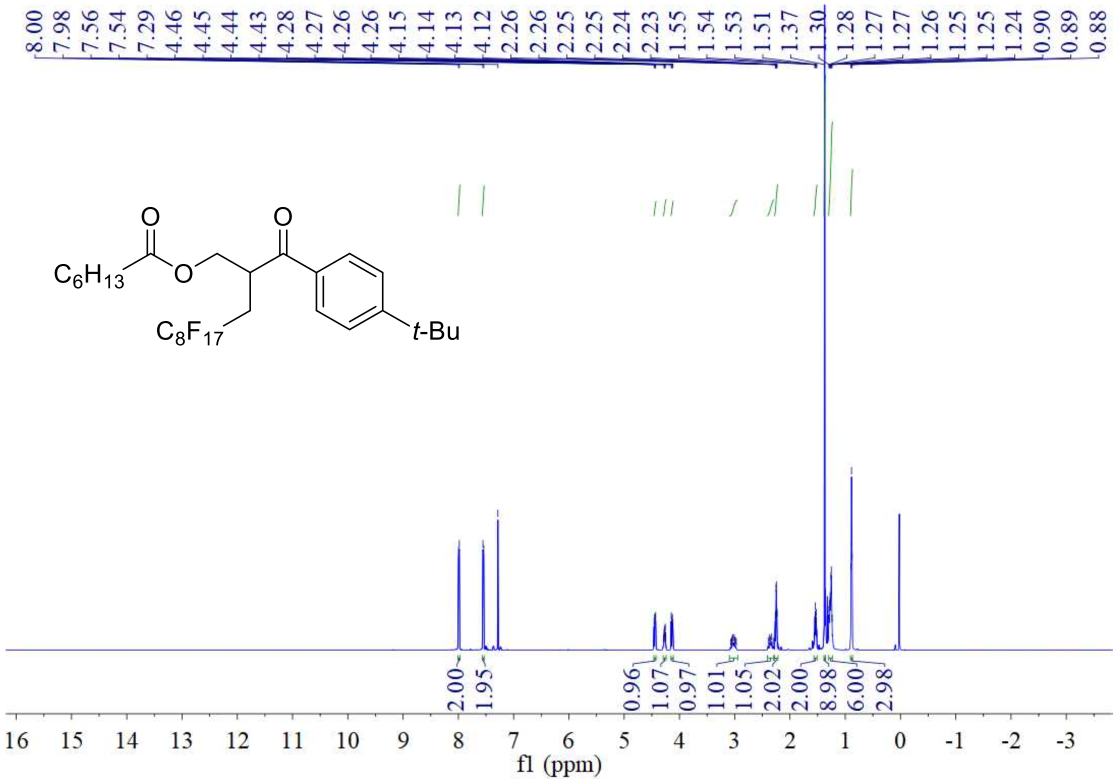

^{19}F NMR (564 MHz, $CDCl_3$)

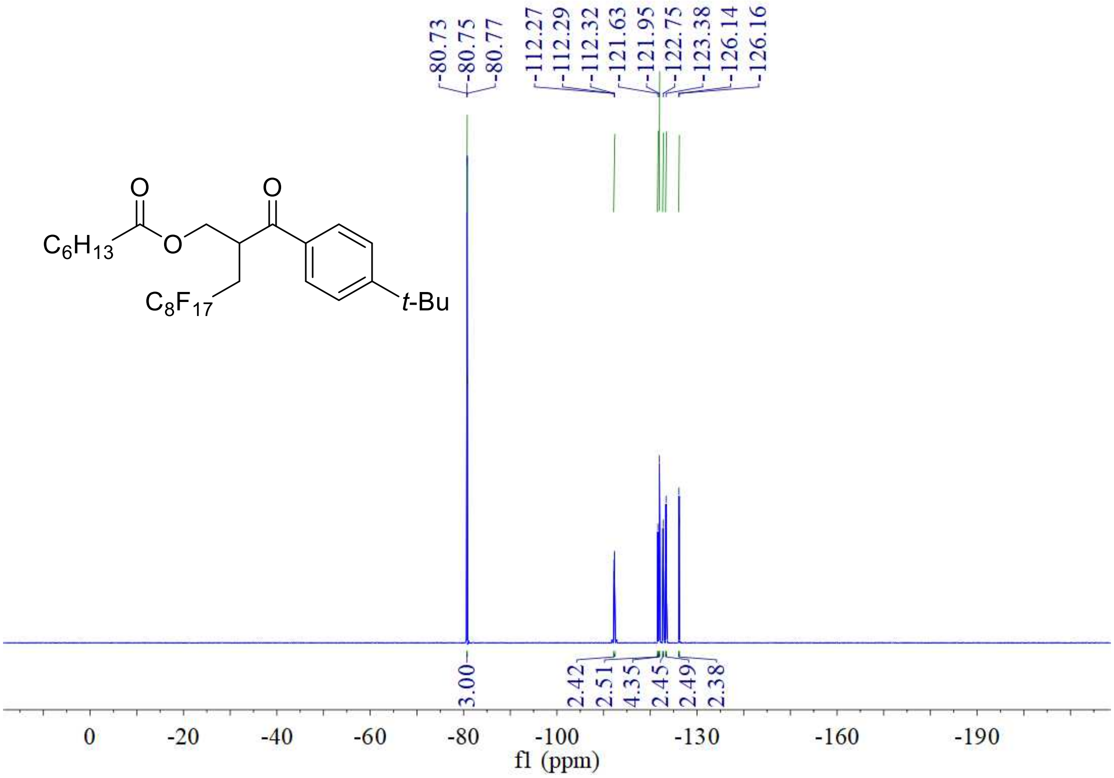

^{13}C NMR (150 MHz, $CDCl_3$)

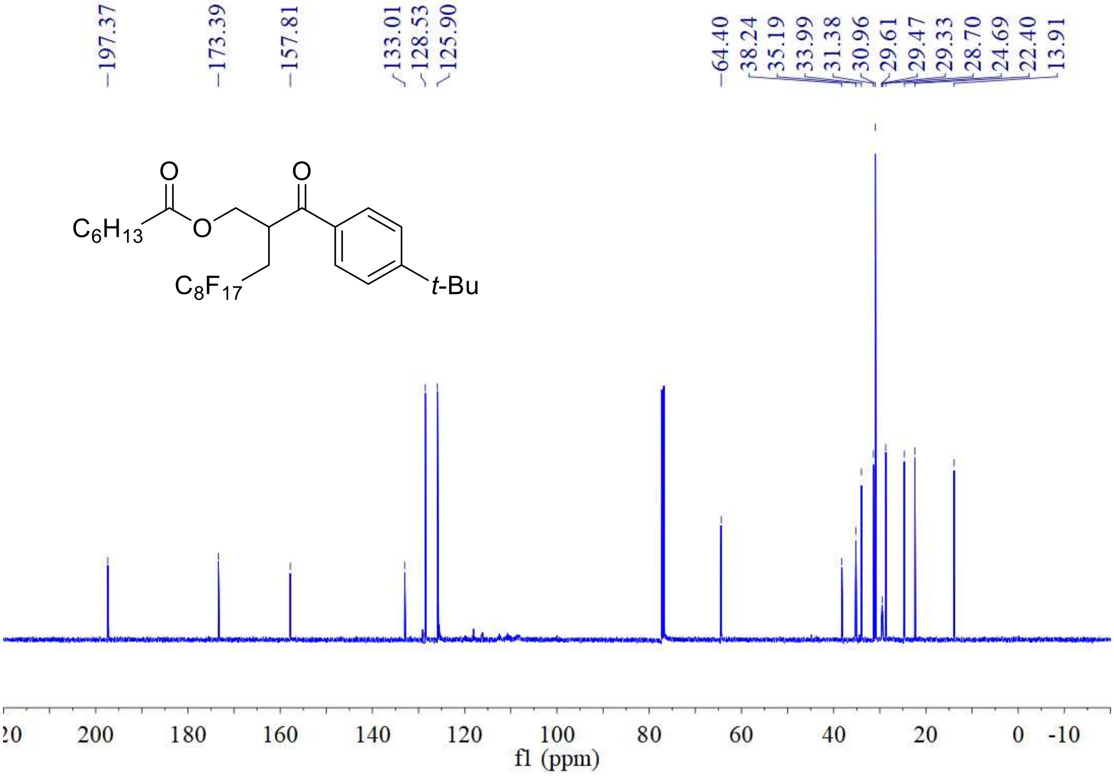

Supplementary Figure 63: NMR Spectra of 2-(4-(*tert*-Butyl)benzoyl)-4,4,5,5,6,6,7,7,8,8,9,9,10,10,11,11,11-heptadecafluoroundecyl heptanoate (32)

^{1}H NMR (600 MHz, $CDCl_3$)

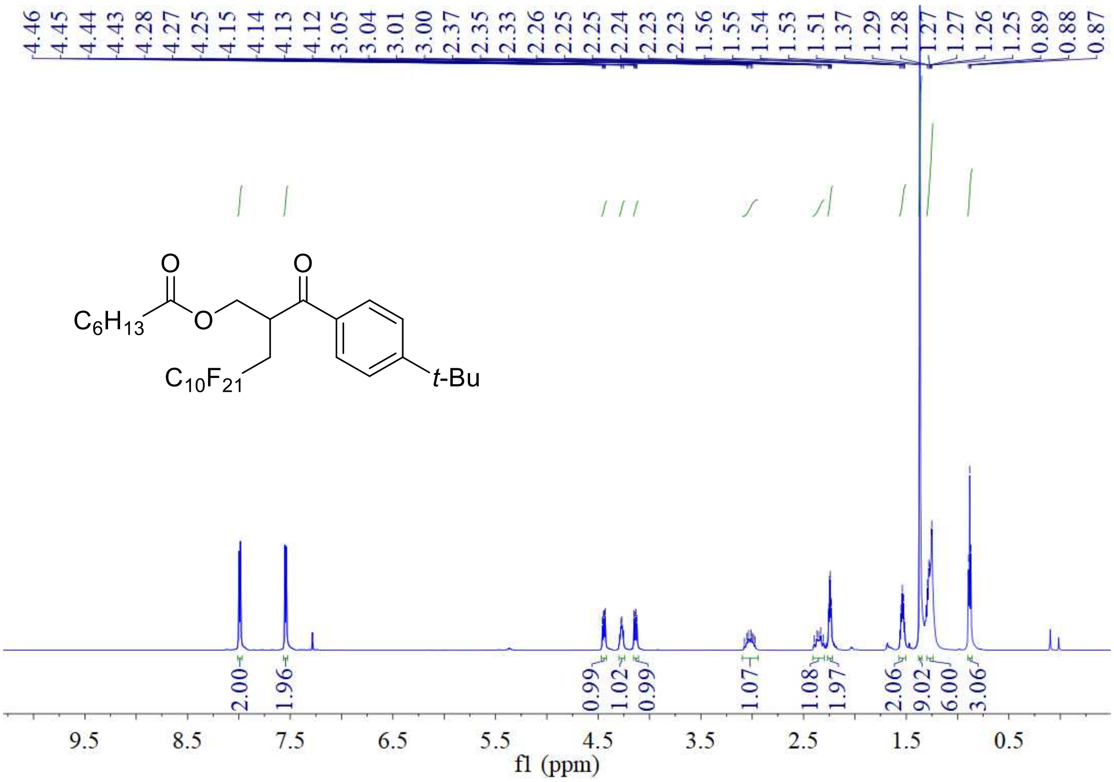

^{19}F NMR (564 MHz, $CDCl_3$)

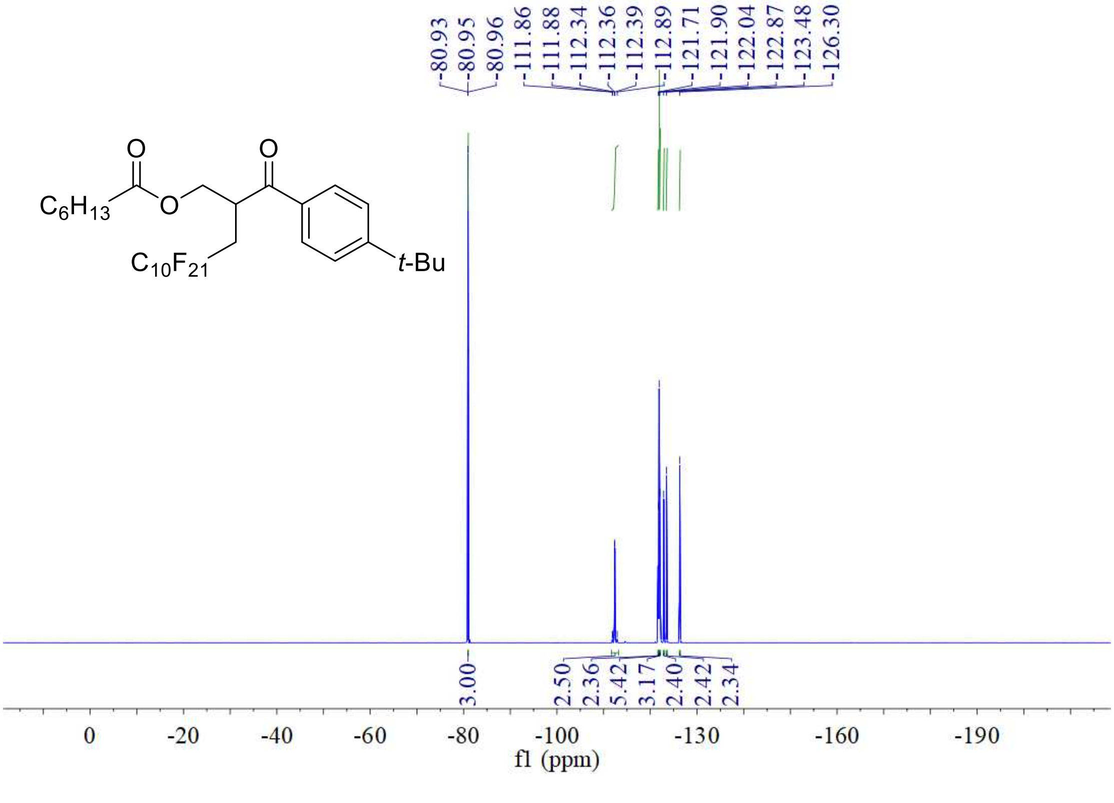

^{13}C NMR (150 MHz, $CDCl_3$)

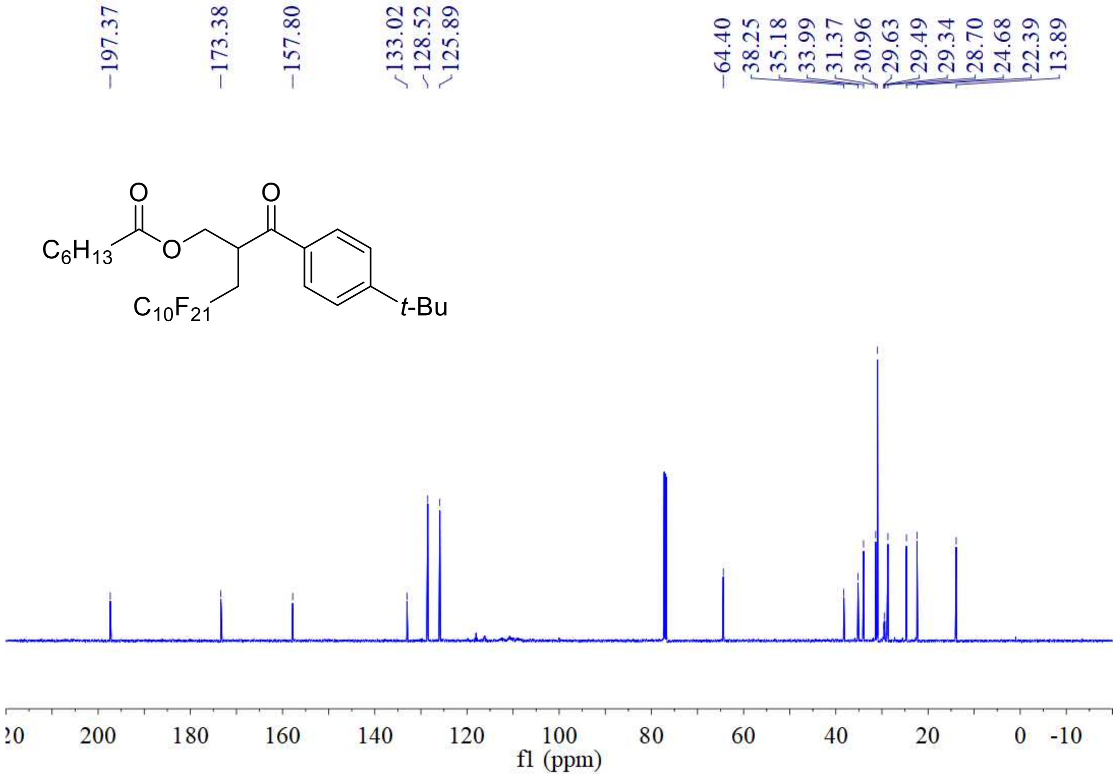

Supplementary Figure 64: NMR Spectra of 2-(4-(*tert*-Butyl)benzoyl)-4,4,5,5,6,6,7,7,8,8,9,9,10,10,11,11,12,12,13,13,13-henicosafluorotridecyl heptanoate (33)

^{1}H NMR (600 MHz, $CDCl_3$)

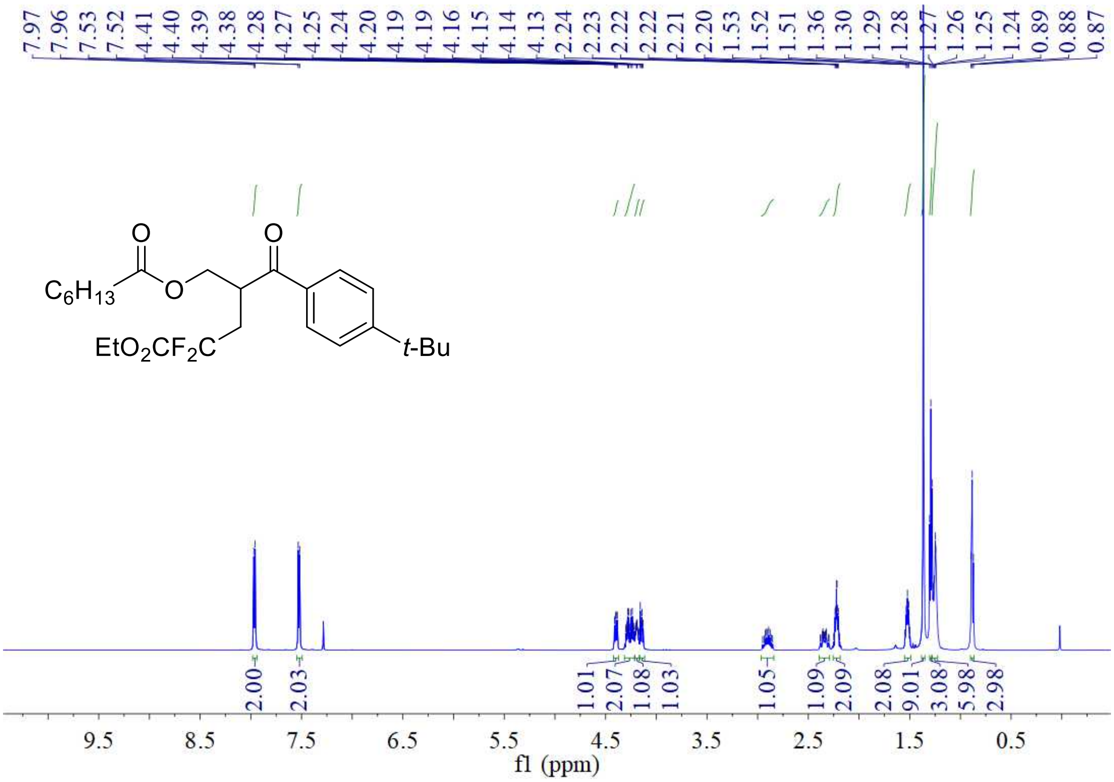

^{19}F NMR (564 MHz, $CDCl_3$)

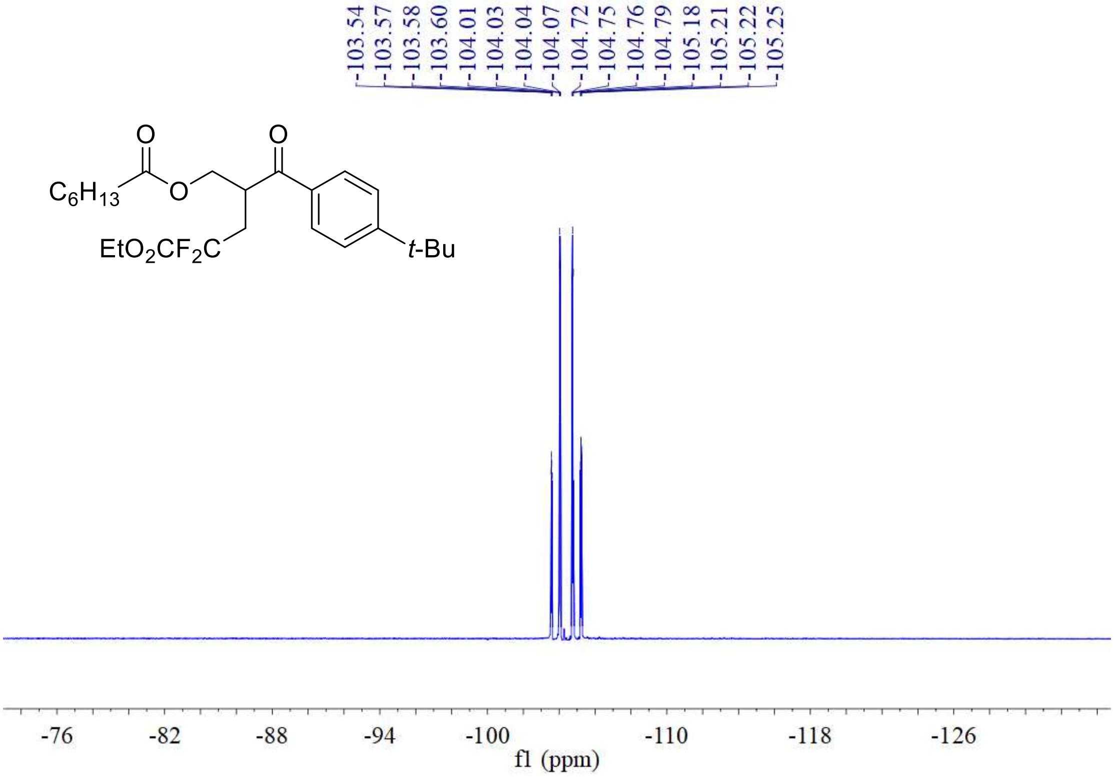

^{13}C NMR (150 MHz, $CDCl_3$)

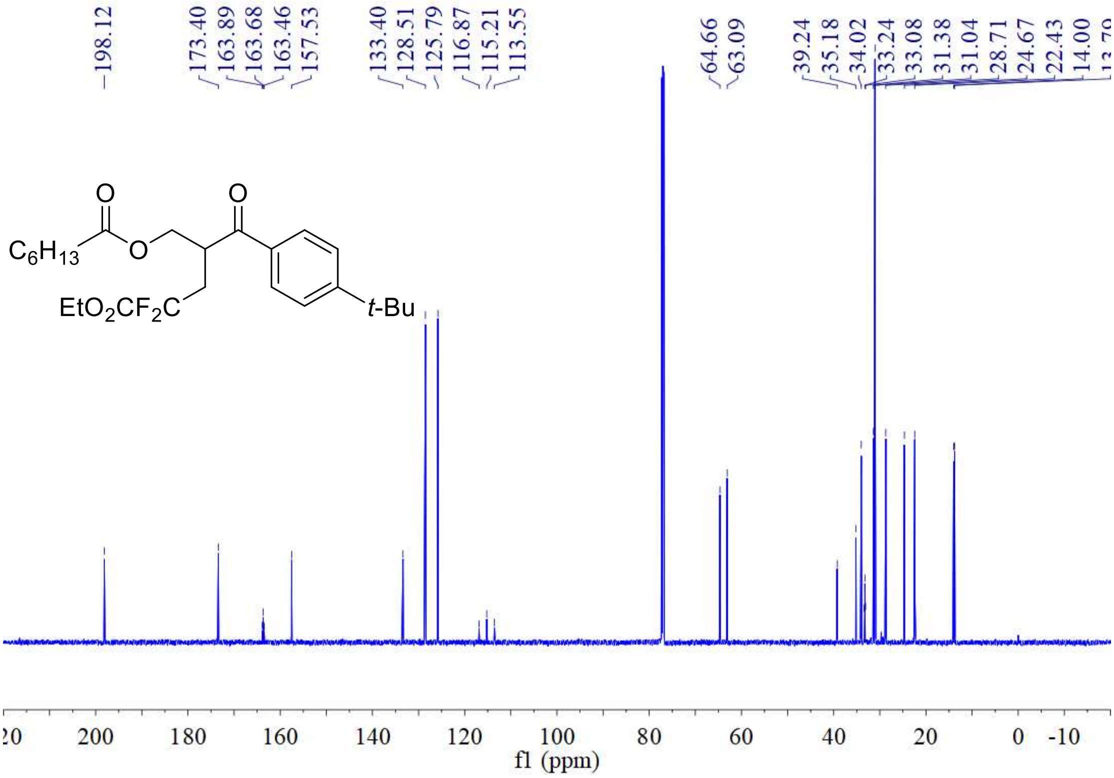

Supplementary Figure 65: NMR Spectra of 2-(4-(*tert*-Butyl)benzoyl)-5-ethoxy-4,4-difluoro-5-oxopentyl heptanoate (34)

^{1}H NMR (600 MHz, $CDCl_3$)

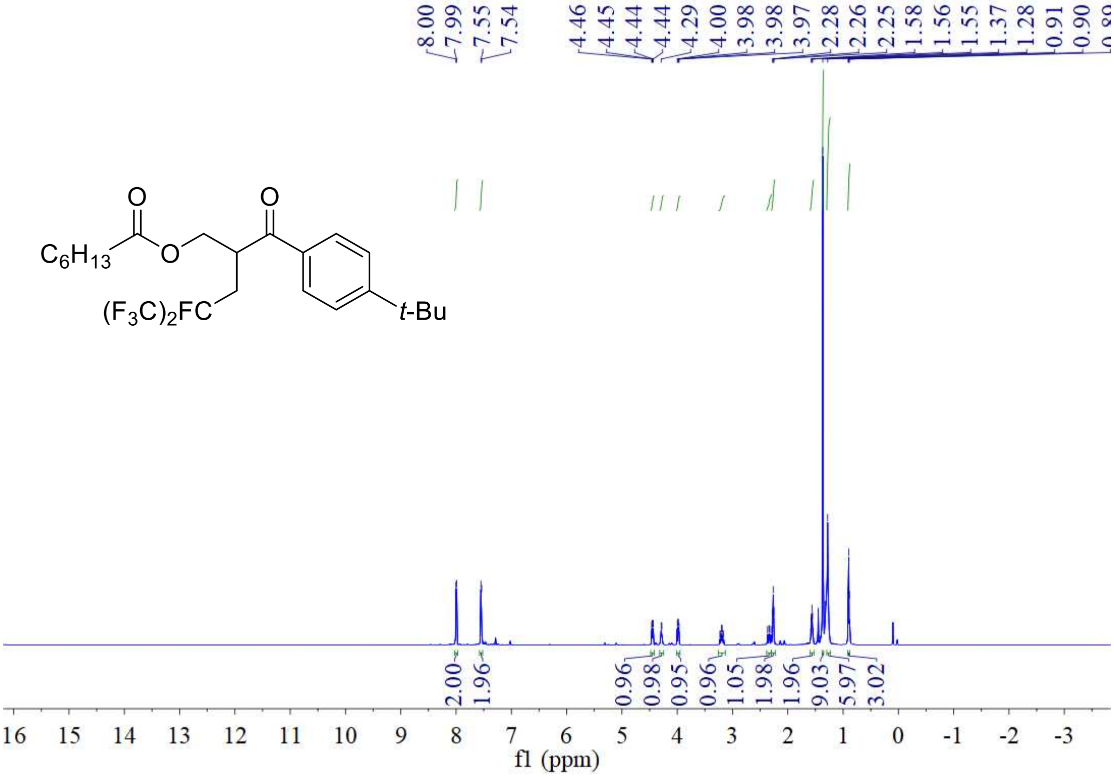

^{19}F NMR (564 MHz, $CDCl_3$)

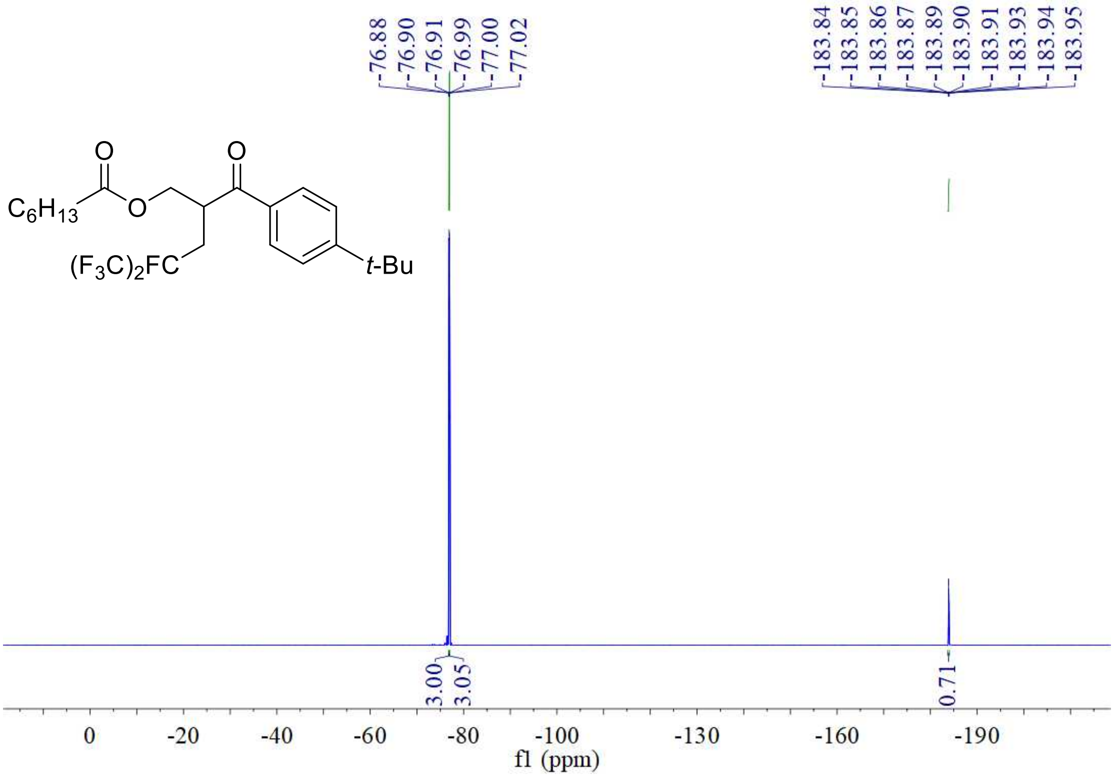

^{13}C NMR (150 MHz, $CDCl_3$)

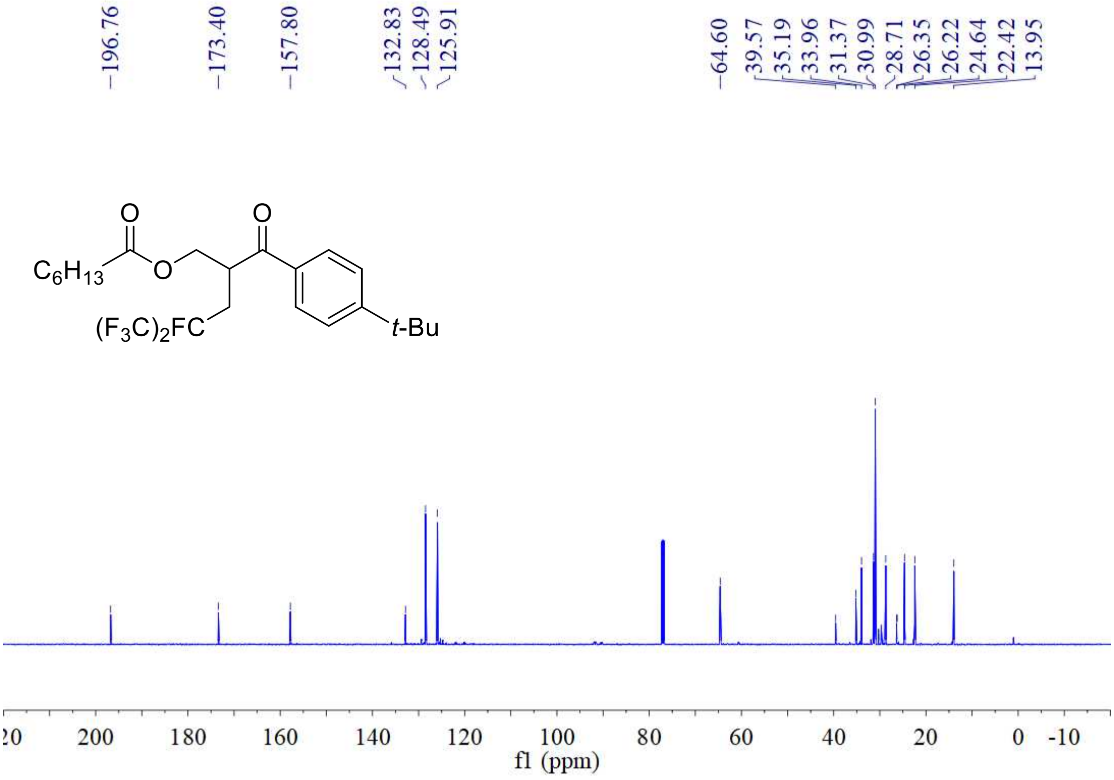

Supplementary Figure 66: NMR Spectra of 2-(4-(*tert*-Butyl)benzoyl

)-4,5,5,5-tetrafluoro-4-(trifluoromethyl)pentyl heptanoate (35)

^{1}H NMR (600 MHz, $CDCl_3$)

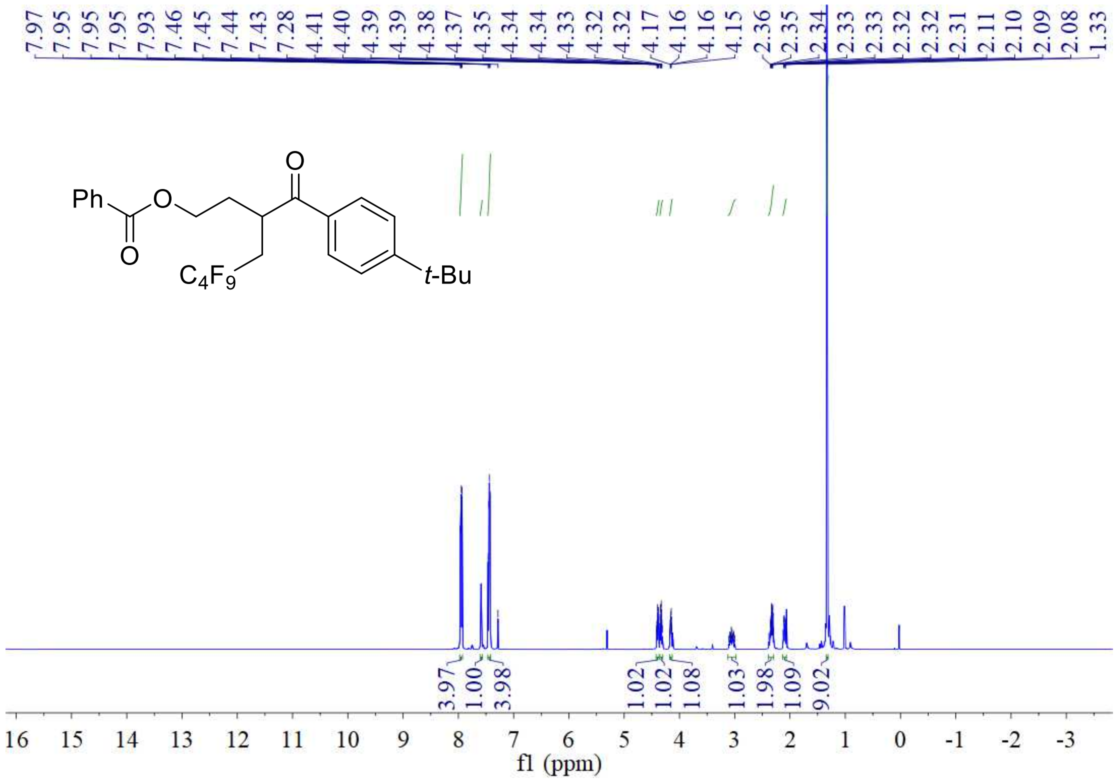

^{19}F NMR (564 MHz, $CDCl_3$)

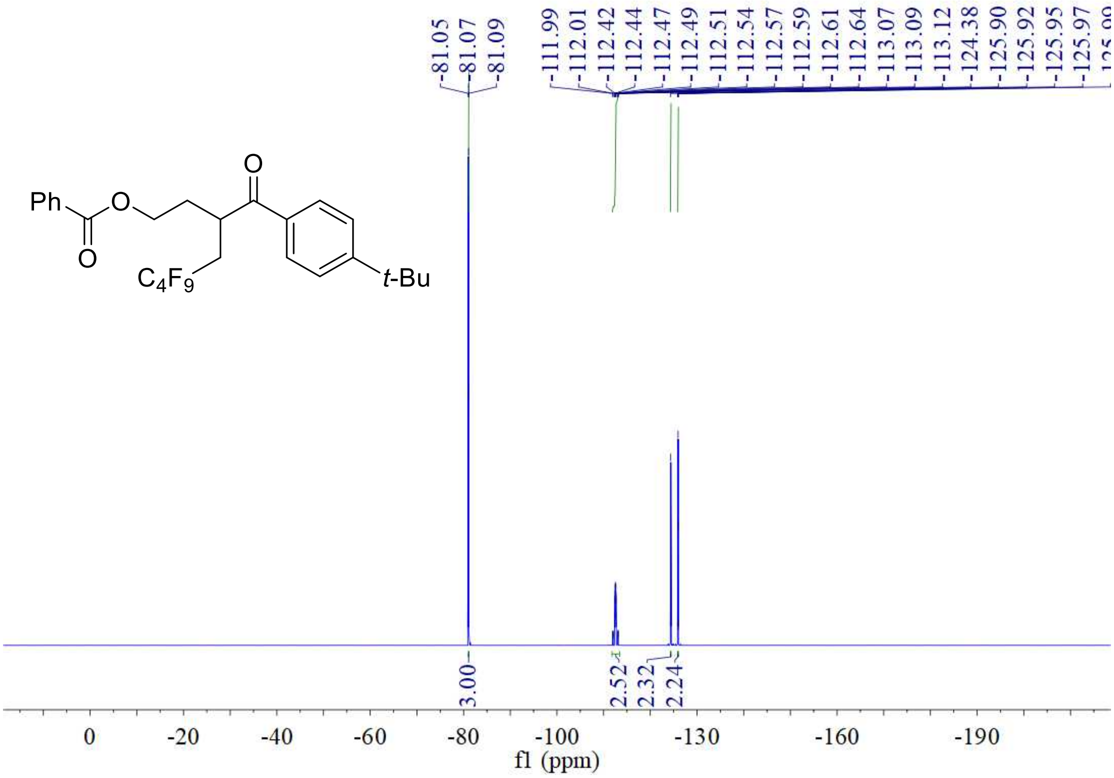

^{13}C NMR (150 MHz, $CDCl_3$)

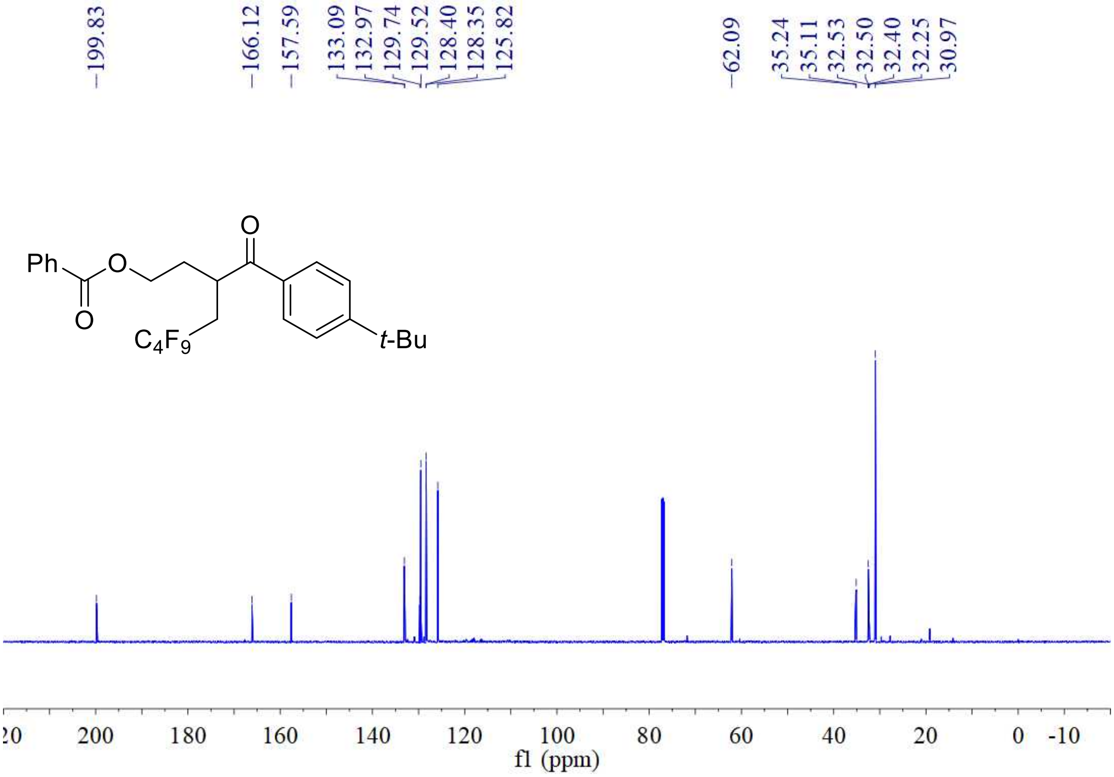

Supplementary Figure 67: NMR Spectra of 3-(4-(*tert*-Butyl)benzoyl)-5,5,6,6,7,7,8,8,8-nonafluorooctyl benzoate (36)

^{1}H NMR (600 MHz, $CDCl_3$)

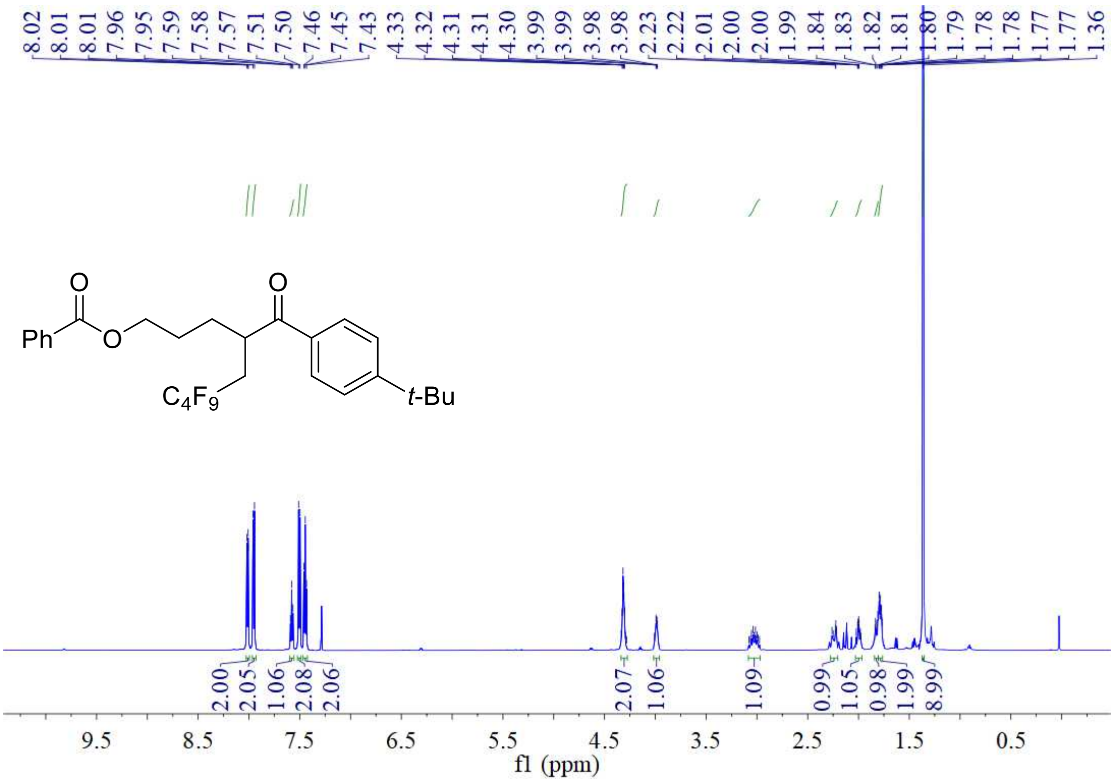

^{19}F NMR (564 MHz, $CDCl_3$)

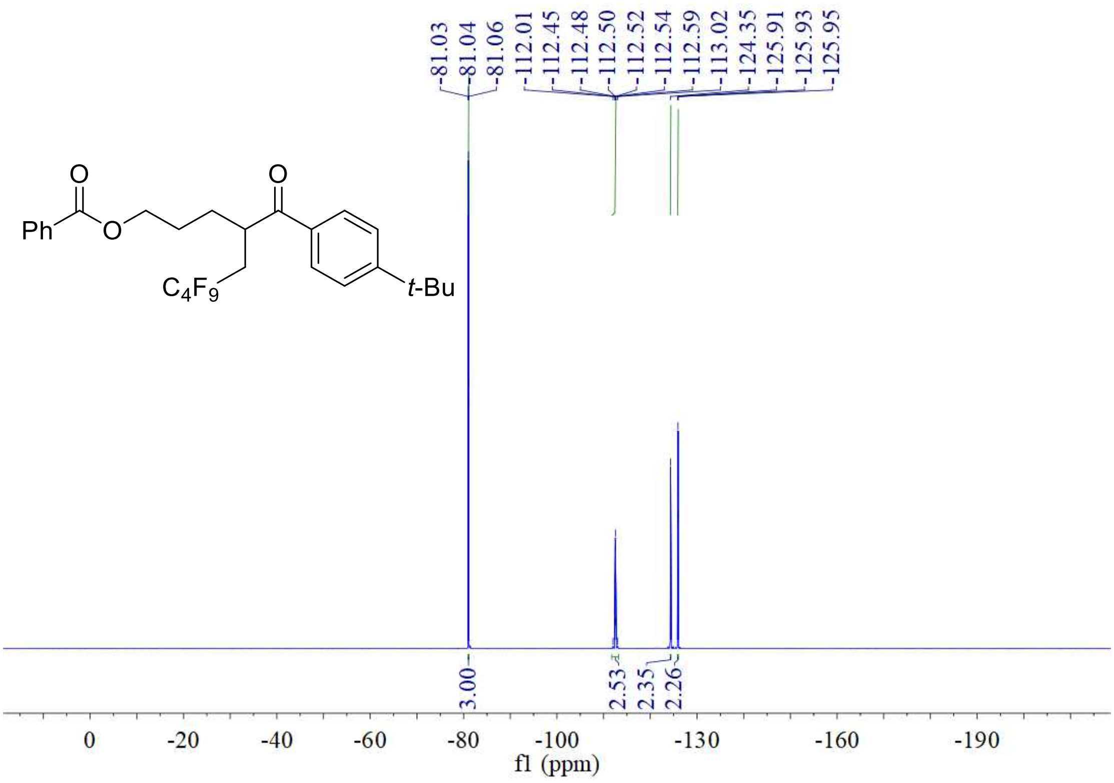

^{13}C NMR (150 MHz, $CDCl_3$)

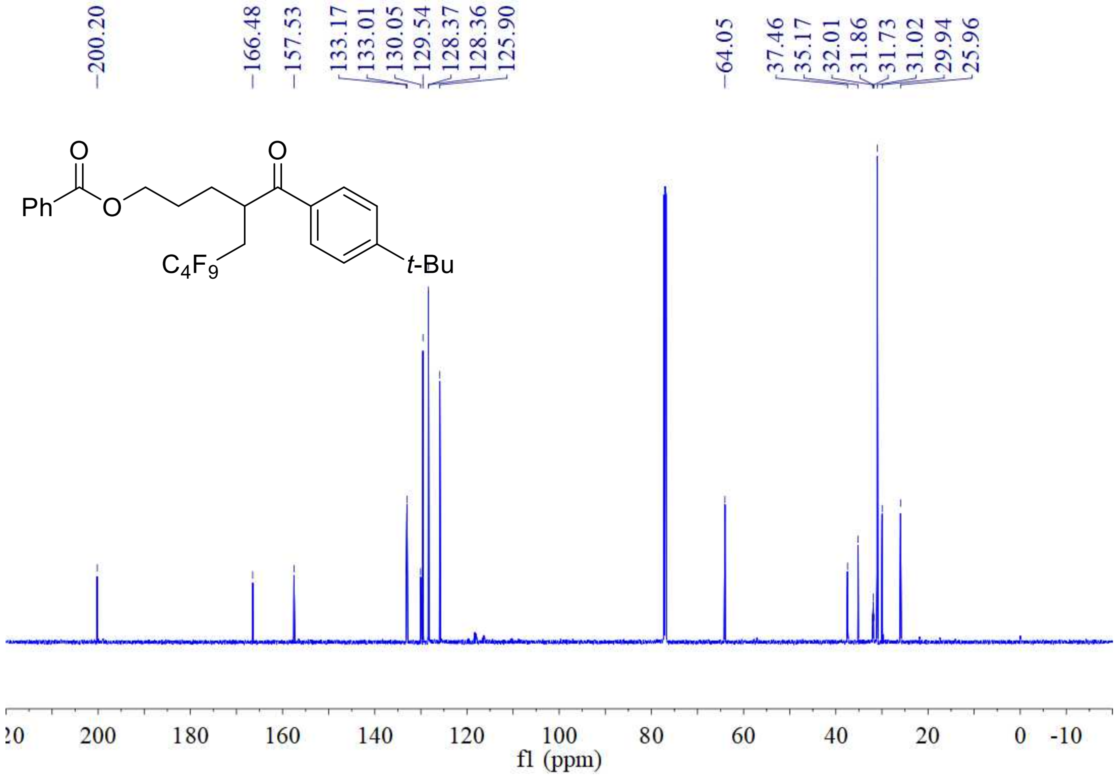

Supplementary Figure 68: NMR Spectra of 4-(4-(*tert*-Butyl)benzoyl)-6,6,7,7, 8,8,9,9,9-nonafluorononyl benzoate (37)

^{1}H NMR (600 MHz, $CDCl_3$)

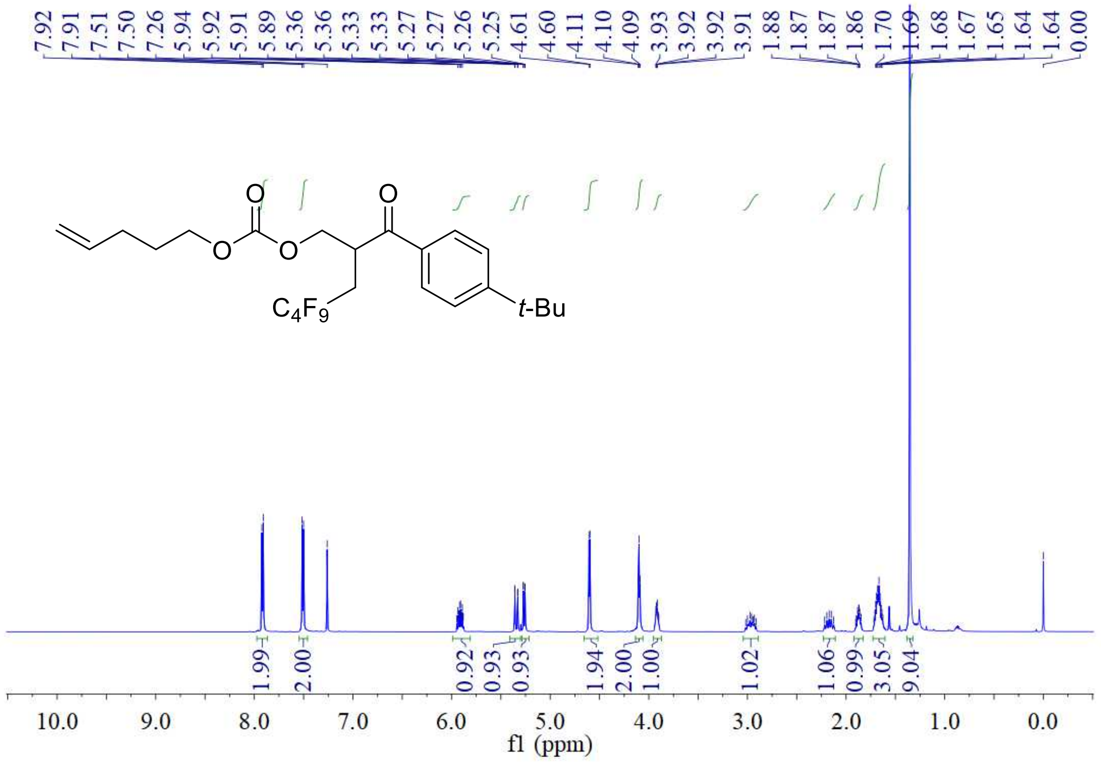

^{19}F NMR (564 MHz, $CDCl_3$)

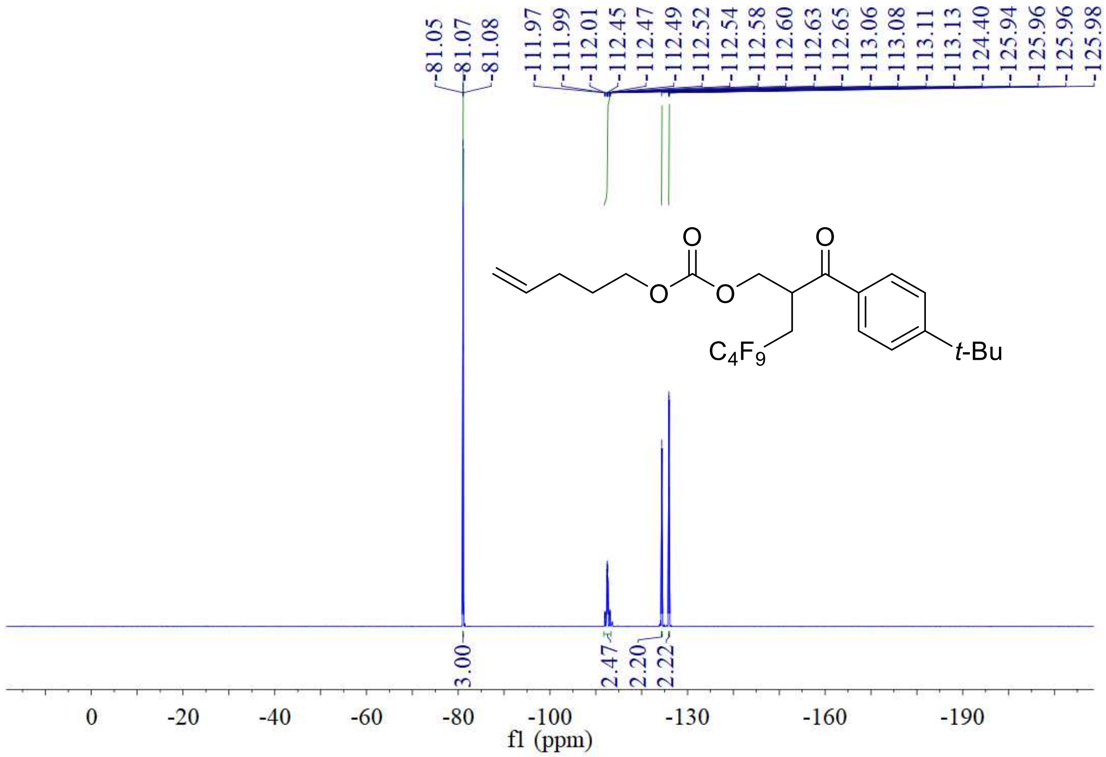

^{13}C NMR (150 MHz, $CDCl_3$)

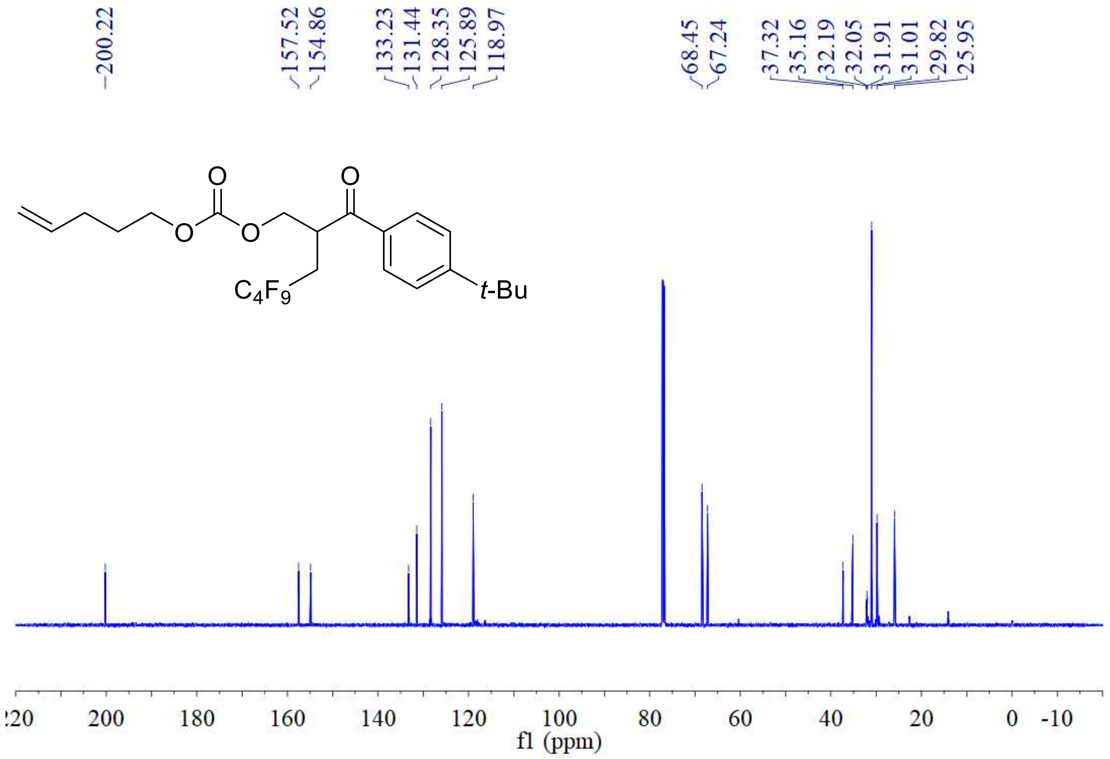

Supplementary Figure 69: NMR Spectra of 2-(4-(*tert*-Butyl)benzoyl) -4,4,5,5,6,6,7,7,7-nonafluoroheptyl pent-4-en-1-yl carbonate (41)

^{1}H NMR (600 MHz, $CDCl_3$)

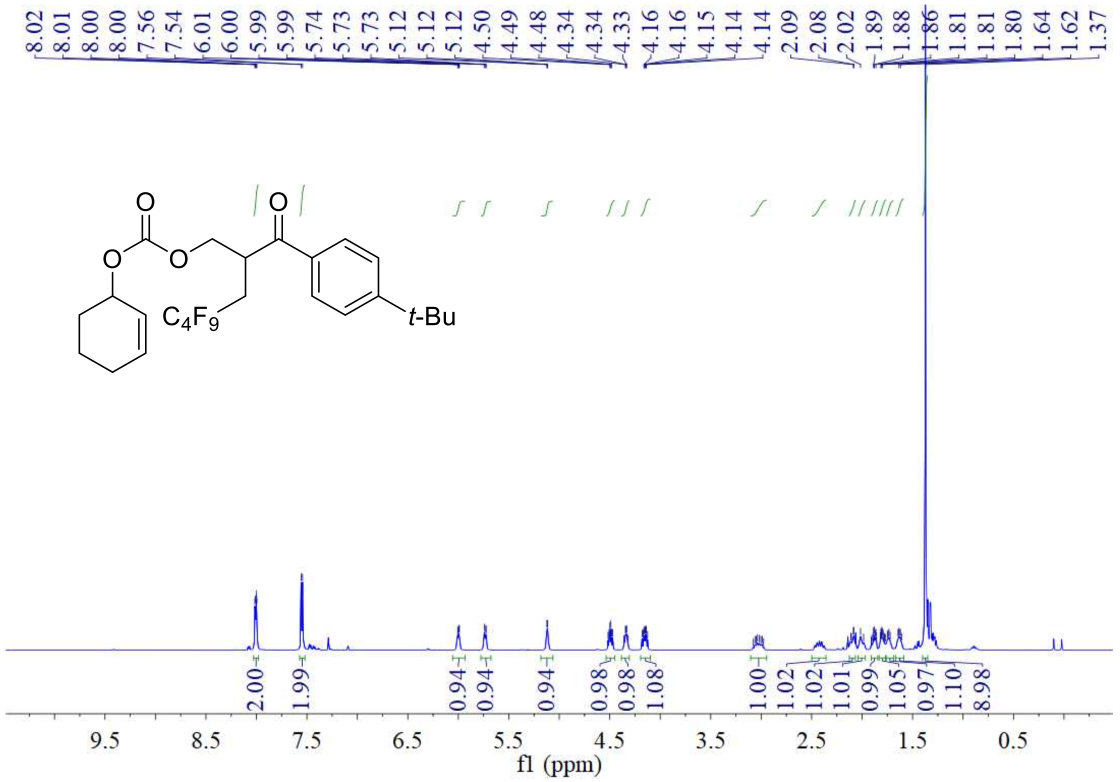

^{19}F NMR (564 MHz, $CDCl_3$)

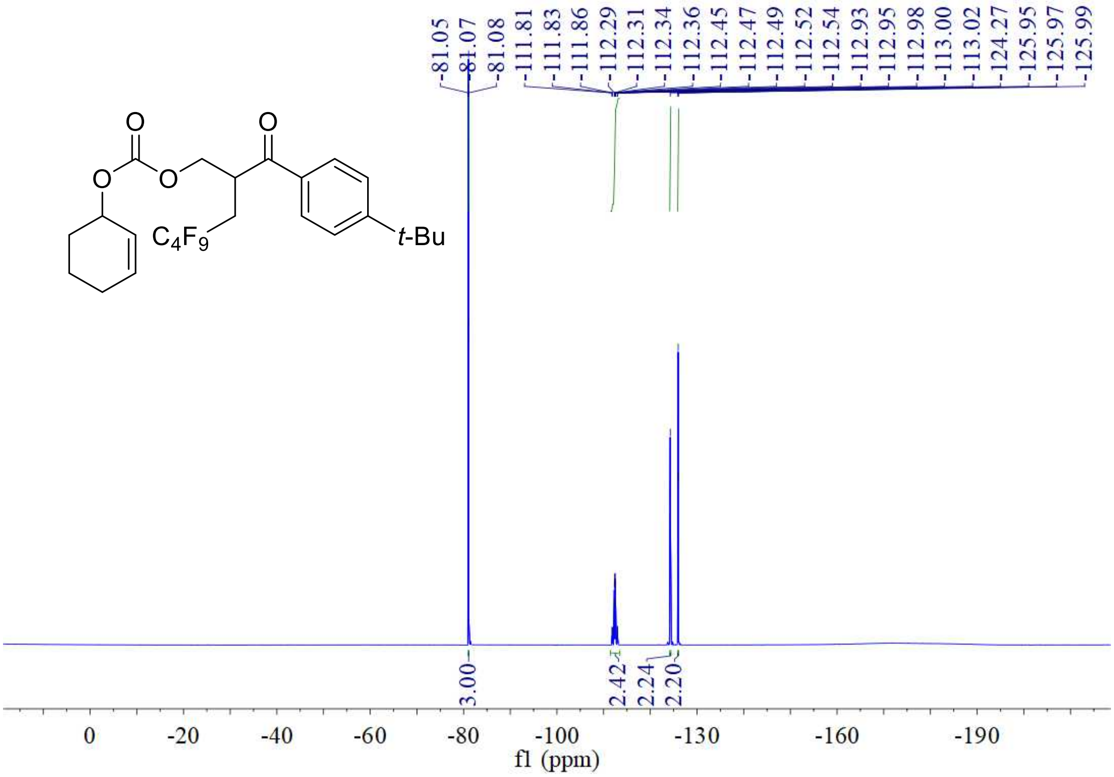

^{13}C NMR (150 MHz, $CDCl_3$)

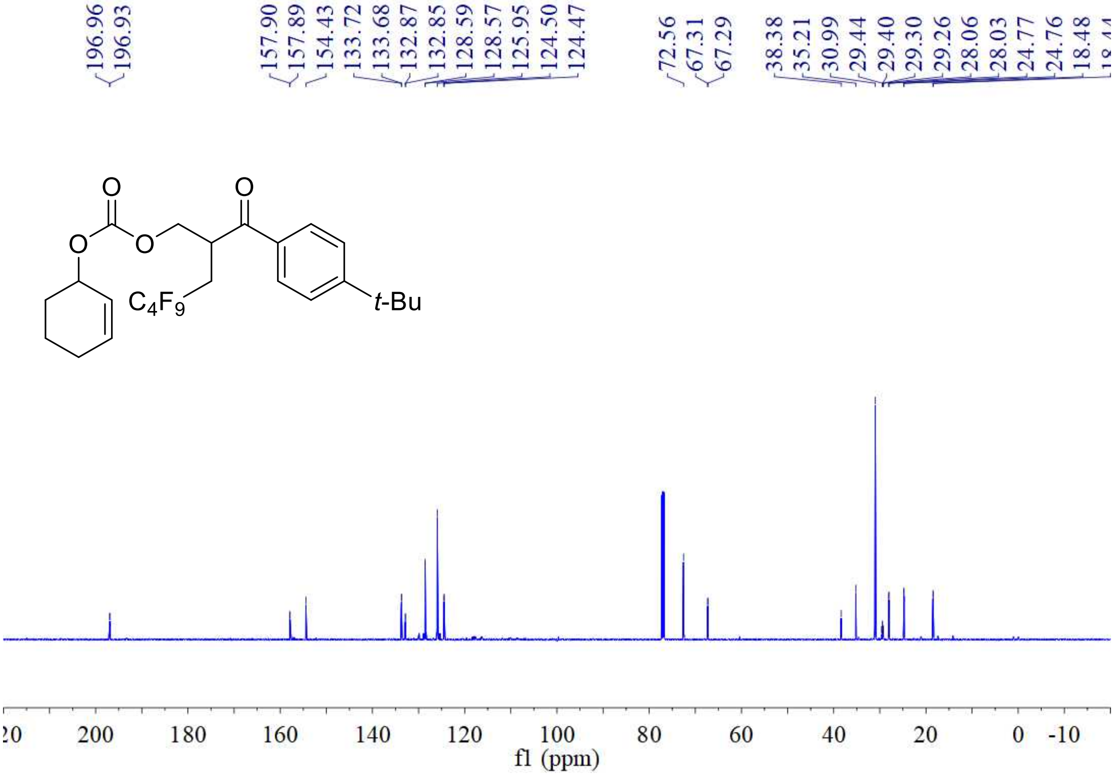

Supplementary Figure 70: NMR Spectra of 2-(4-(*tert*-Butyl)benzoyl)-4,4,

5,5,6,6,7,7,7-nonafluoroheptyl cyclohex-2-en-1-yl carbonate (42)

^{1}H NMR (600 MHz, $CDCl_3$)

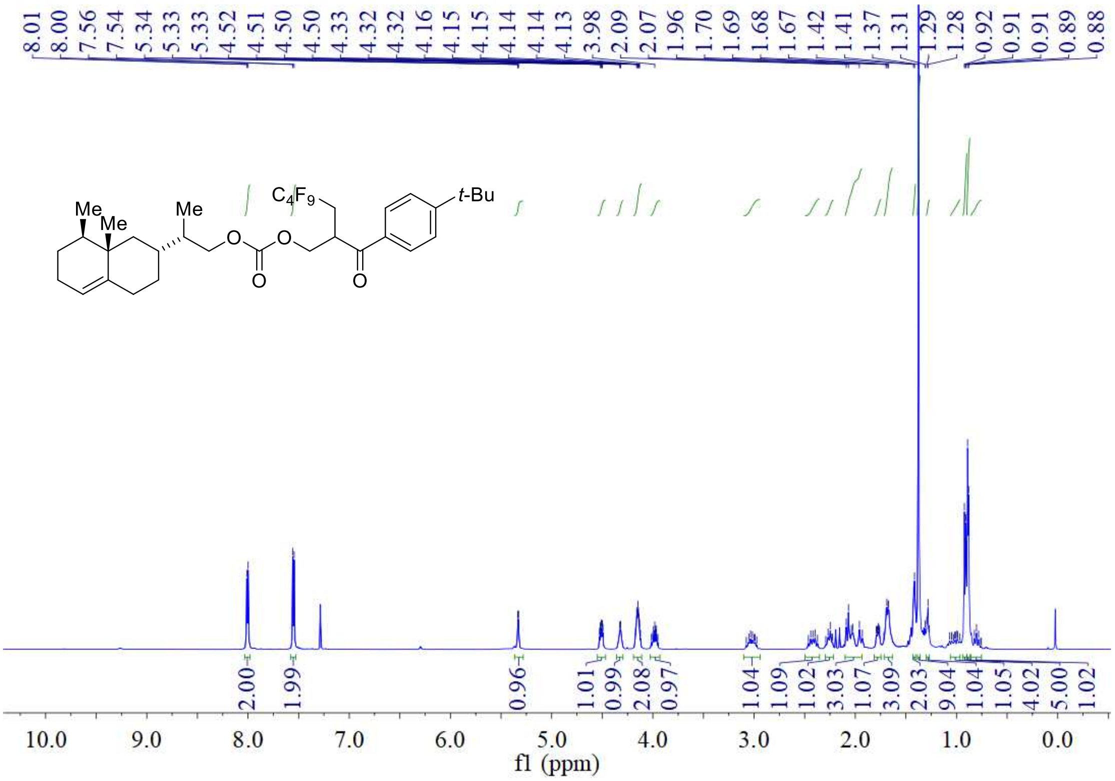

^{19}F NMR (564 MHz, $CDCl_3$)

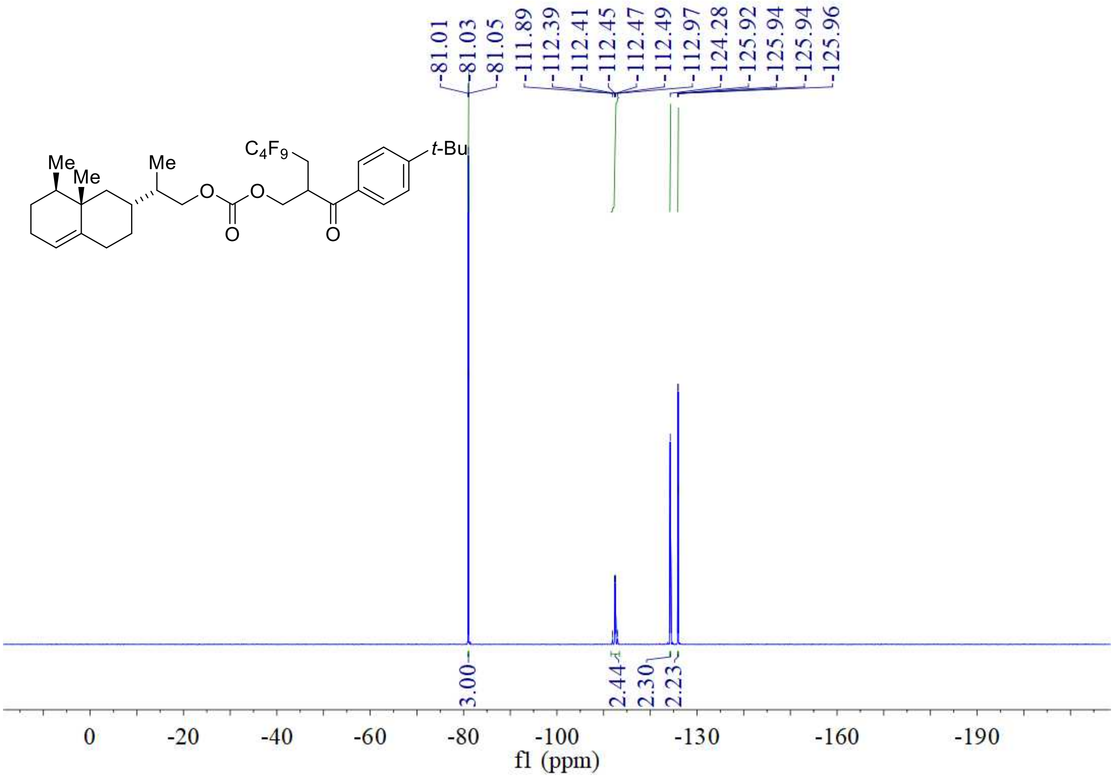

^{13}C NMR (150 MHz, $CDCl_3$)

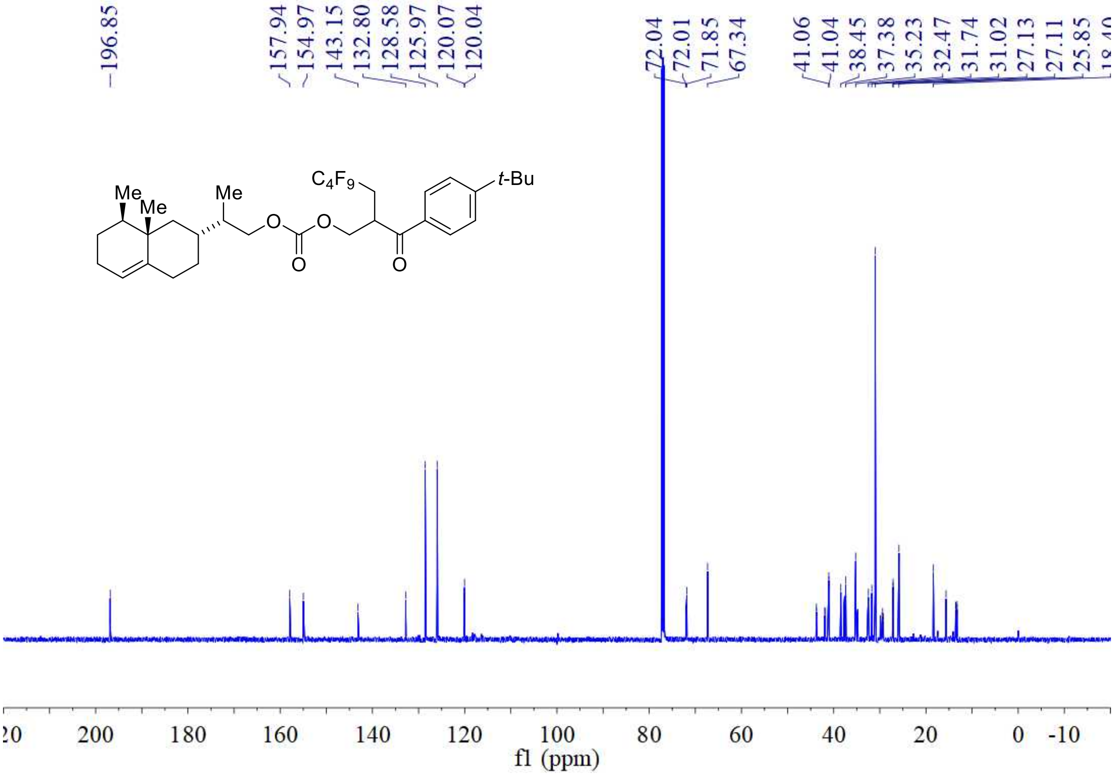

Supplementary Figure 71: NMR Spectra of 2-(4-(*tert*-Butyl)benzoyl)-3,3,4,4,5,5,6,6,6-nonafluorohexyl (2-8,8a-dimethyl -1,2,3,4,6,7,8,8a-octahydronaphthalen-2-yl)propyl) carbonate (43)

^{1}H NMR (600 MHz, $CDCl_3$)

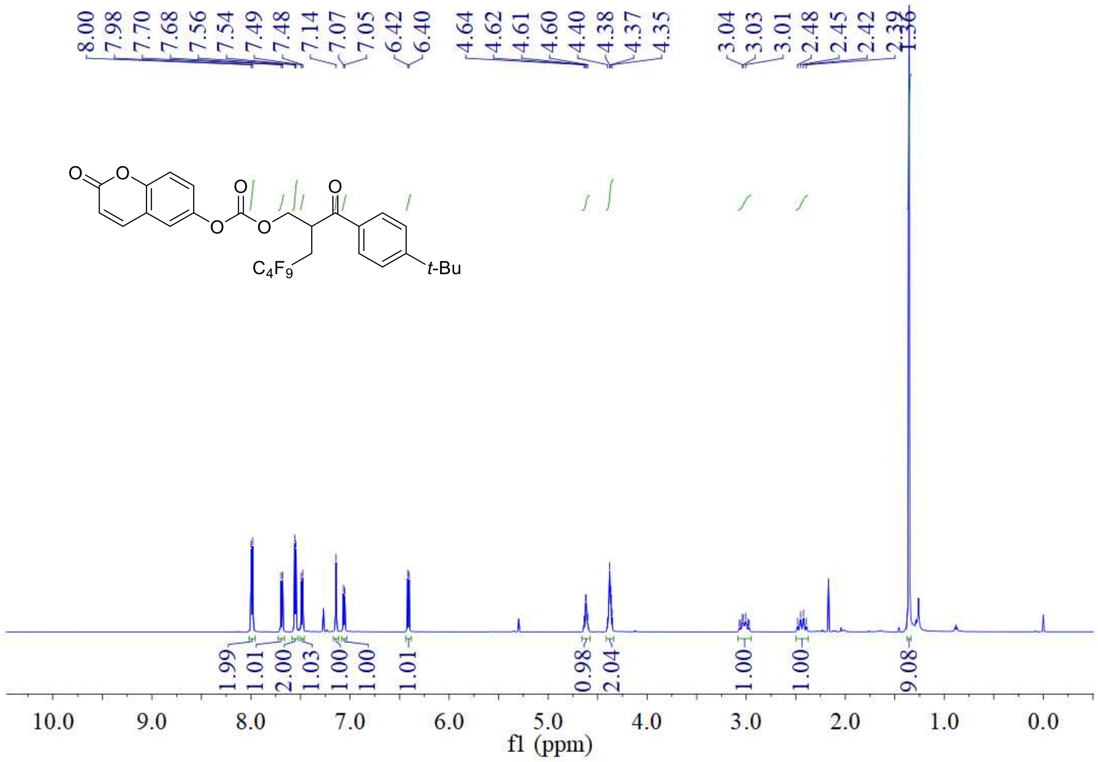

^{19}F NMR (564 MHz, $CDCl_3$)

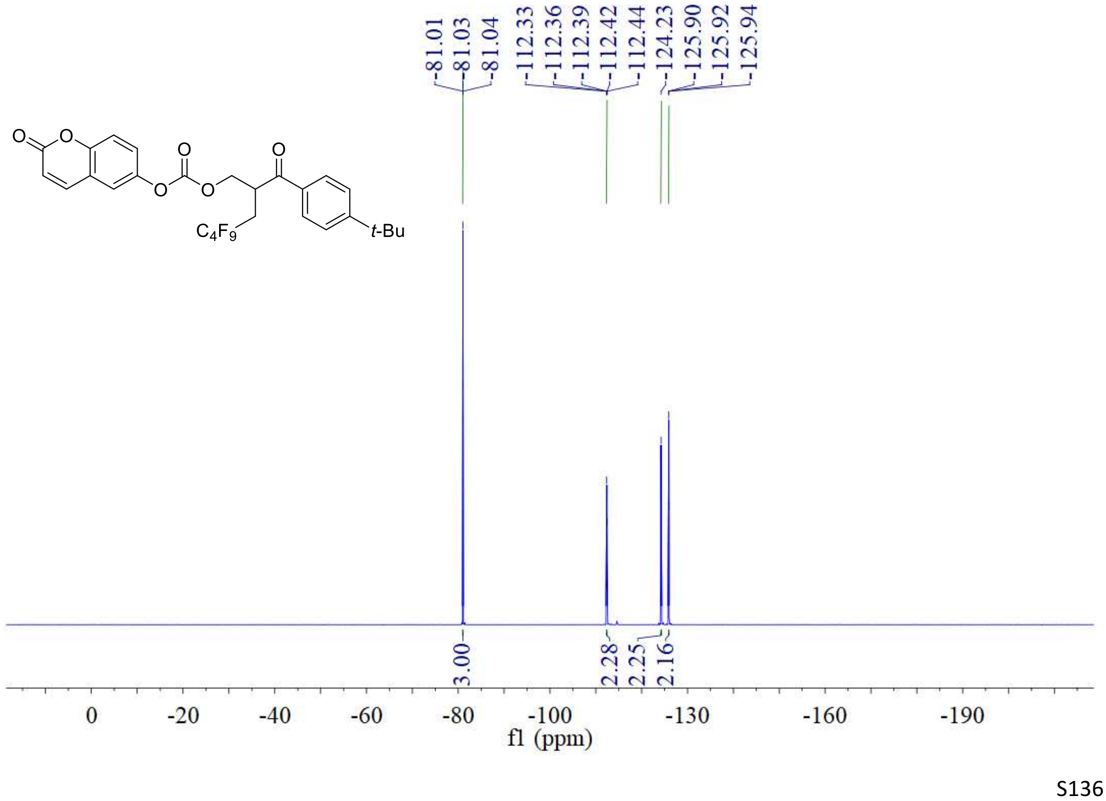

^{13}C NMR (100 MHz, $CDCl_3$)

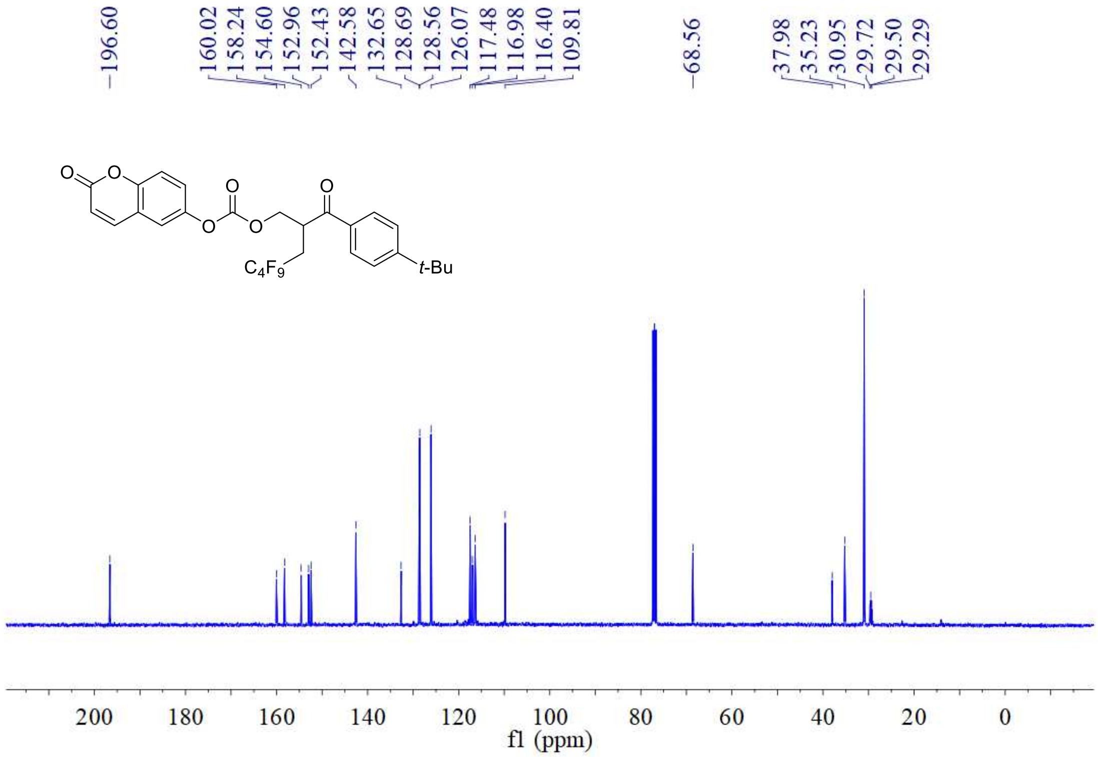

Supplementary Figure 72: NMR Spectra of 2-(4-(*tert*-Butyl)benzoyl)-4,4,5,5,6,6,7,7,7-nonafluoroheptyl(2-oxo-2H-chromen-6-yl) carbonate (44)

^{1}H NMR (600 MHz, $CDCl_3$)

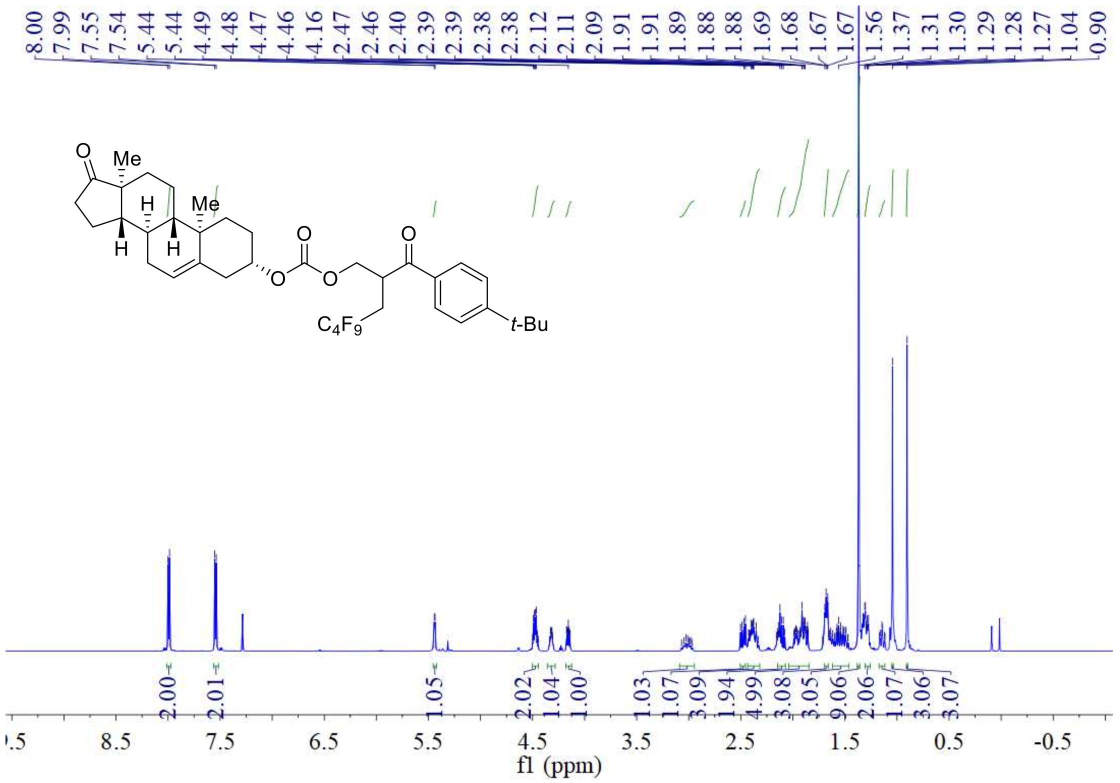

^{19}F NMR (564 MHz, $CDCl_3$)

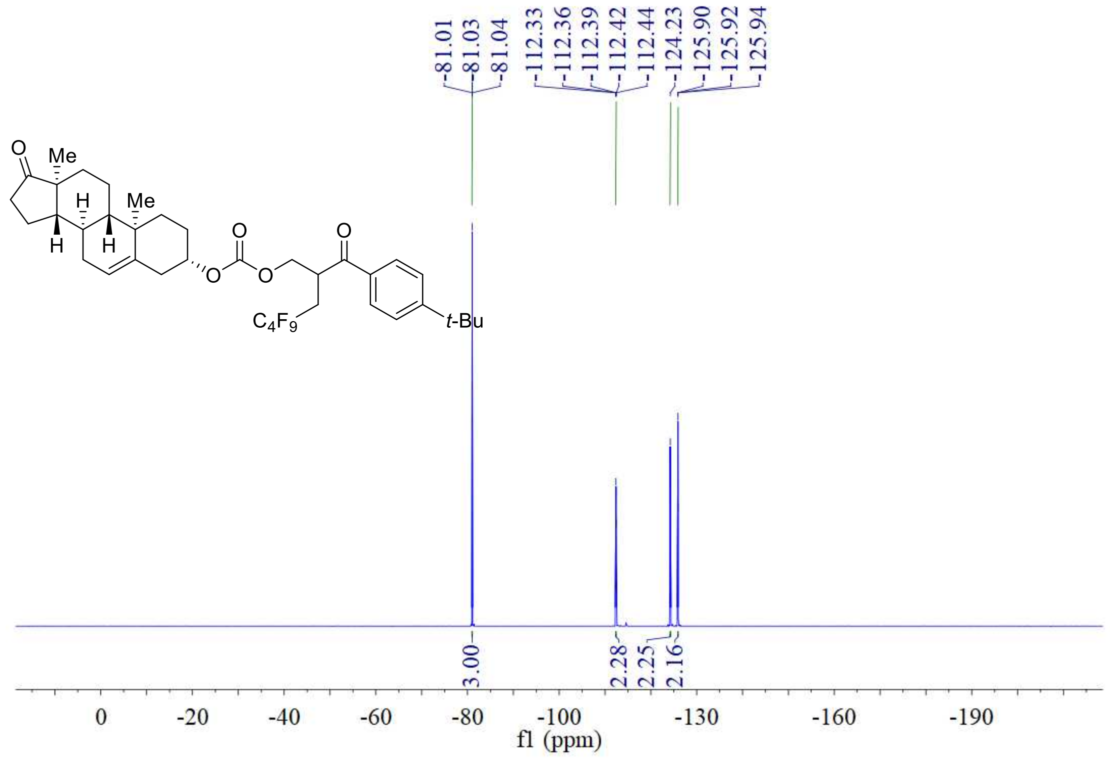

^{13}C NMR (150 MHz, $CDCl_3$)

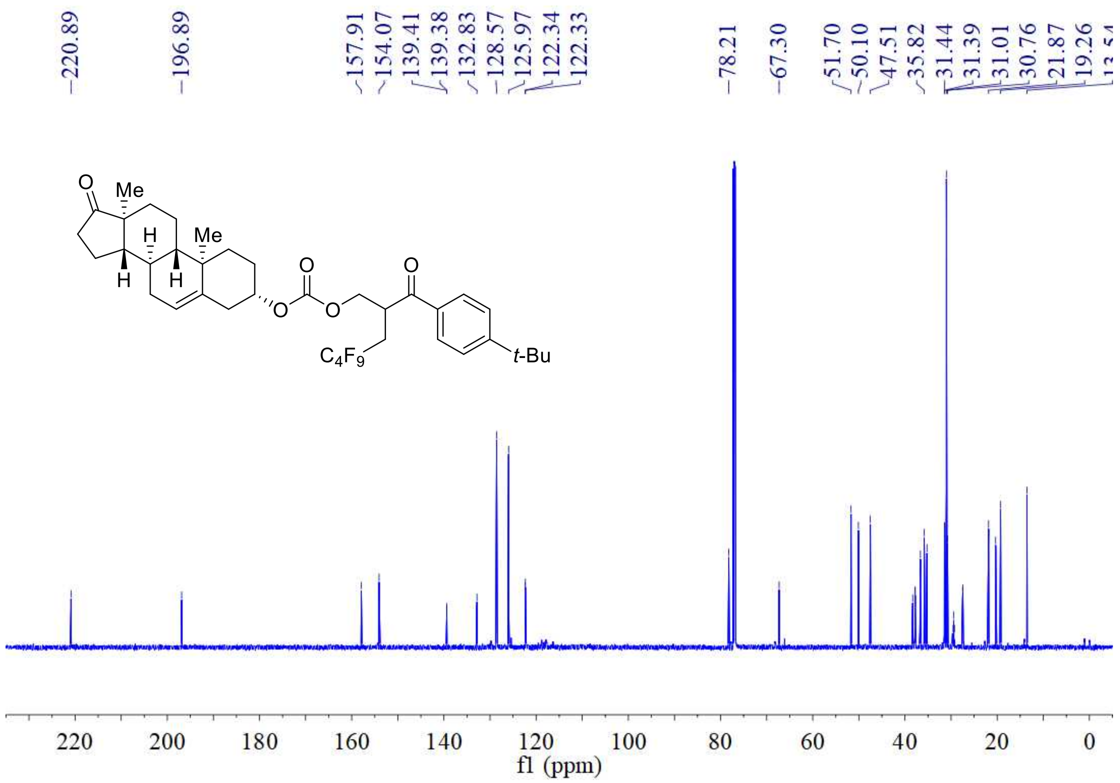

Supplementary Figure 73: NMR Spectra of 2-(4-(*tert*-butyl)benzoyl)-4,4,5,5,6,6,7,7,7-nonafluoroheptyl (9,13,14-trimethyl-17-oxo-2,3,4,7,8,9,10,11,12,13,14,15,16,17-tetradecahydro-1H-cyclopenta[a]phenanthren-3-yl) carbonate (45)

^{1}H NMR (600 MHz, $CDCl_3$)

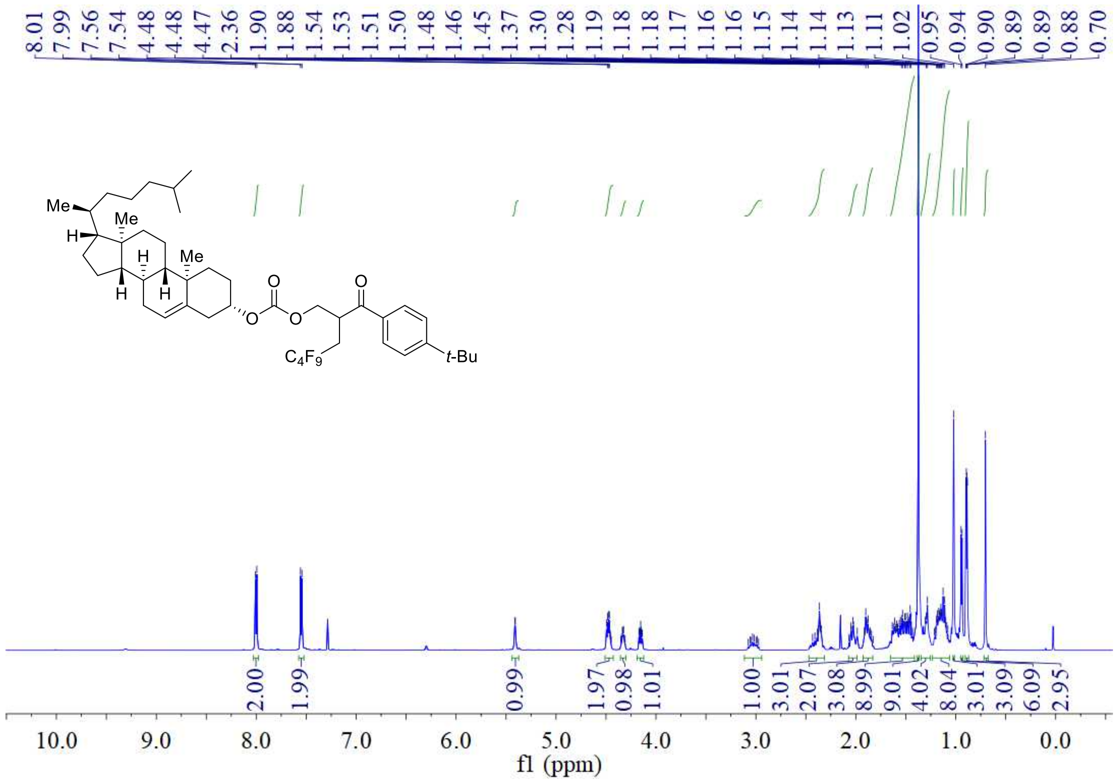

^{19}F NMR (564 MHz, $CDCl_3$)

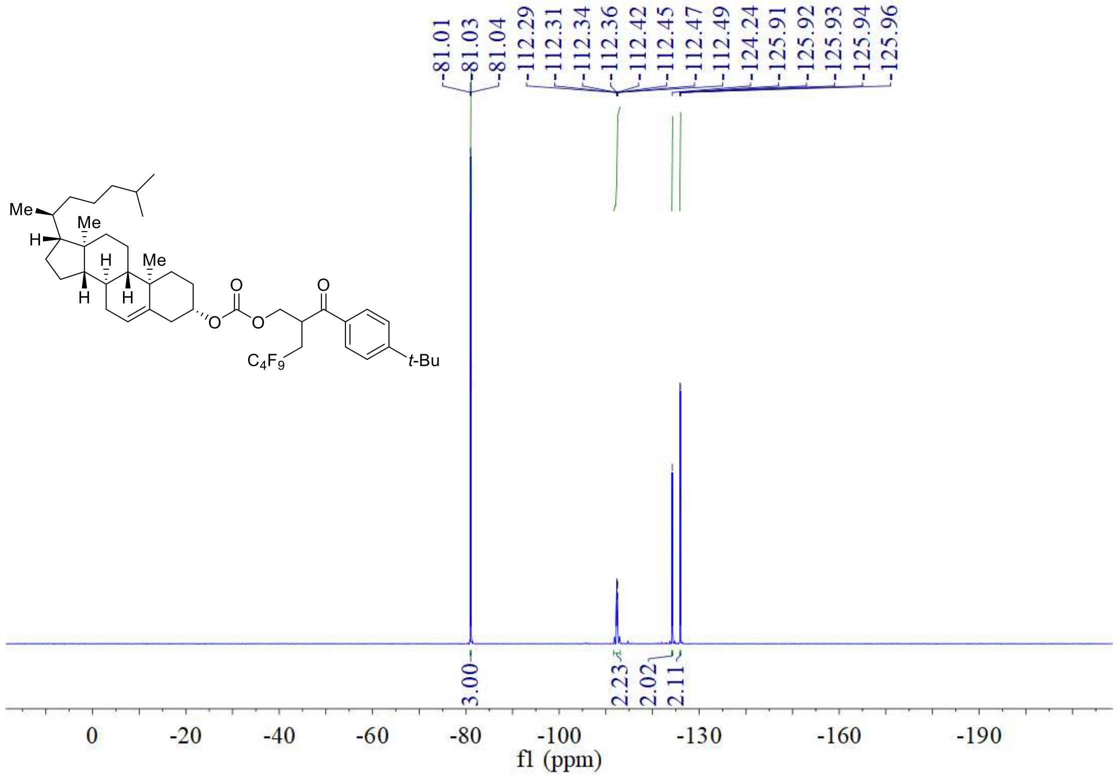

^{13}C NMR (150 MHz, $CDCl_3$)

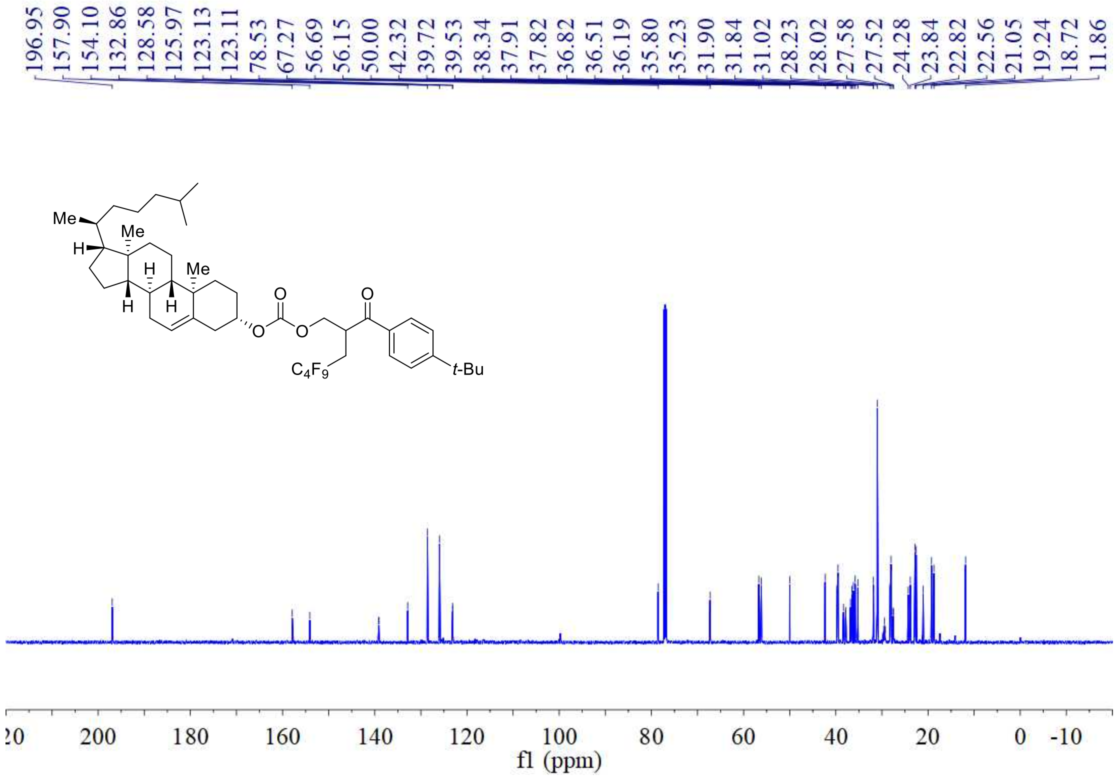

Supplementary Figure 74: NMR Spectra of 2-(4-(*tert*-butyl)benzoyl)-4,4,5,5,6,6,7,7,7-nonafluoroheptyl (10,13-dimethyl-17-(6-methylheptan-2-yl)-2,3,4,7,8,9,10,11,12,13,14,15,16,17-tetradecahydro-1H-cyclopenta[a]phenanthren-3-yl) carbonate (46)

^{1}H NMR (600 MHz, $CDCl_3$)

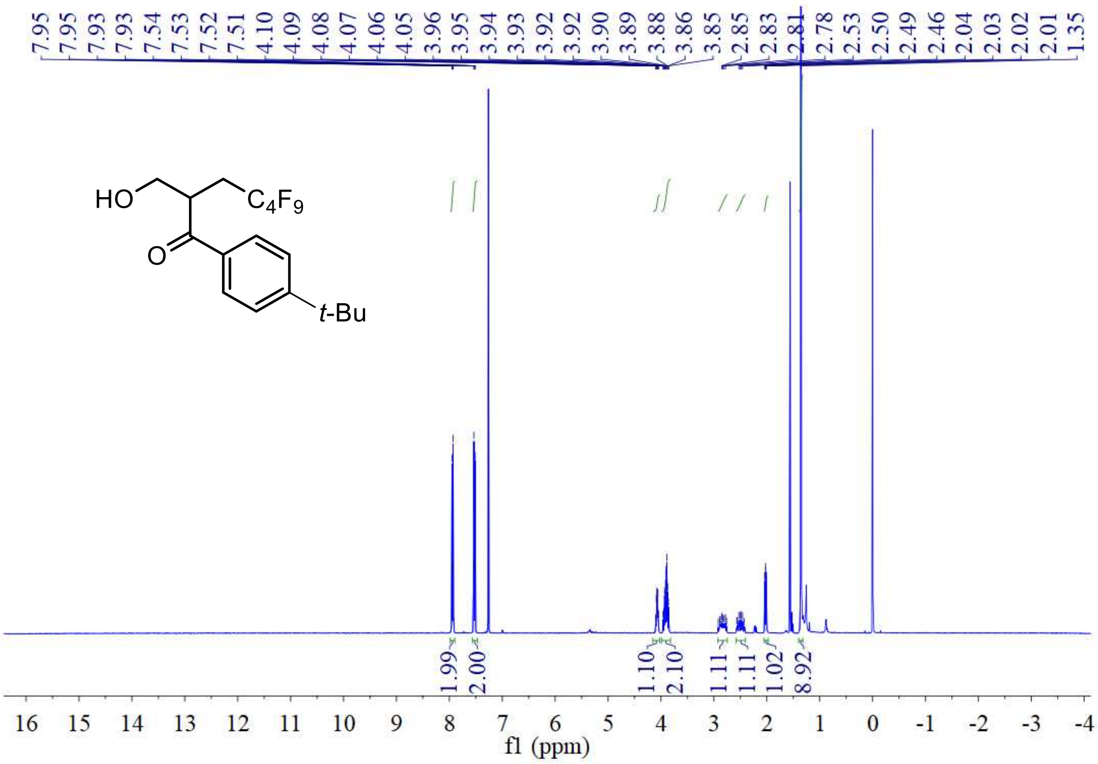

^{19}F NMR (564 MHz, $CDCl_3$)

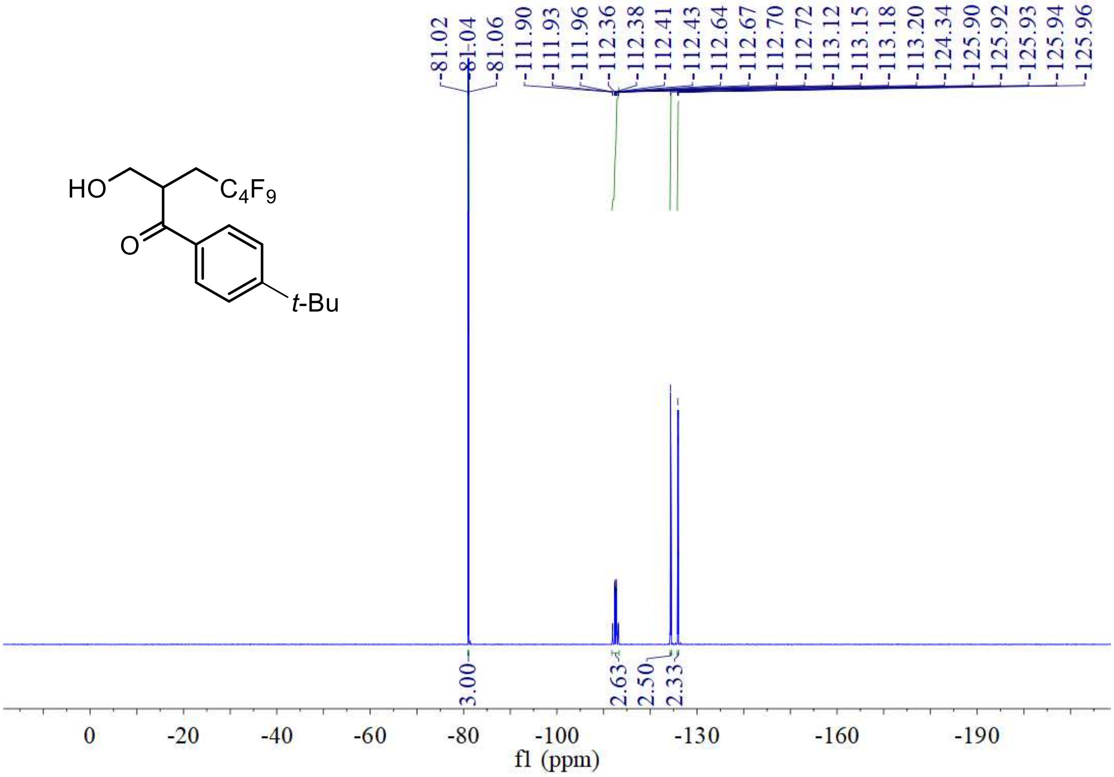

^{13}C NMR (150 MHz, $CDCl_3$)

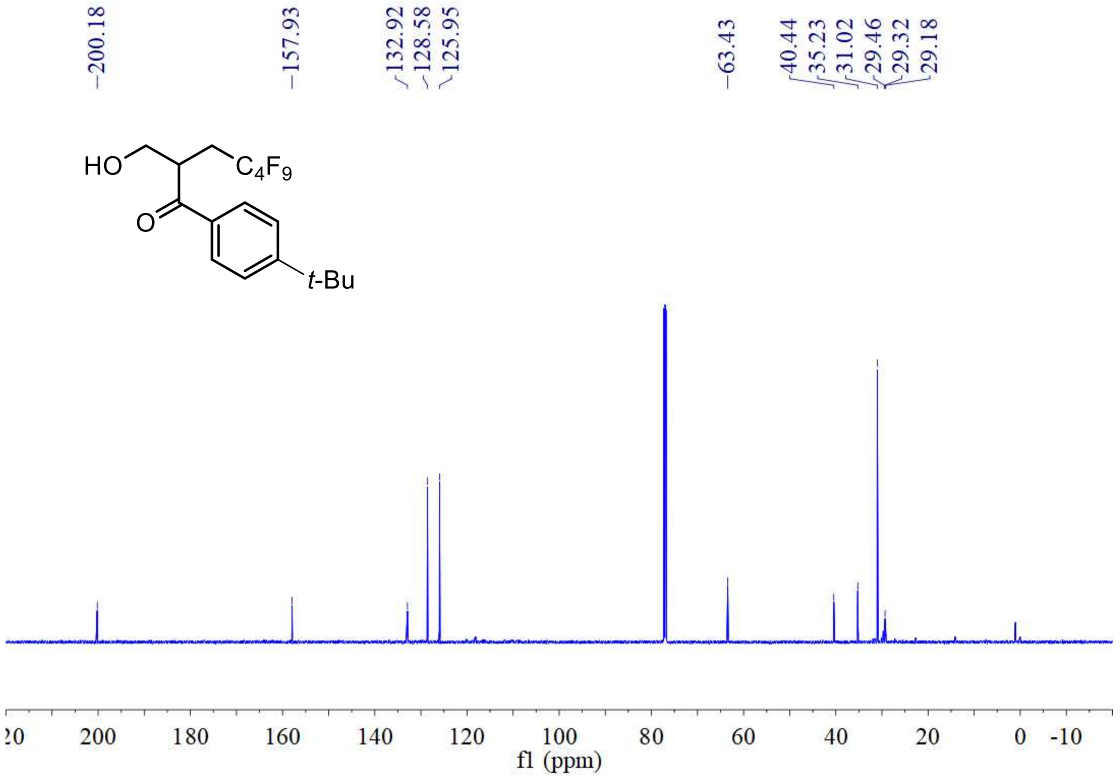

Supplementary Figure 75: NMR Spectra of 1-(4-(tert-Butyl)phenyl)-4,4,5, 5,6,6,7,7,7-nonafluoro-2-(hydroxymethyl)heptan-1-one (47)

^{1}H NMR (400 MHz, $CDCl_3$)

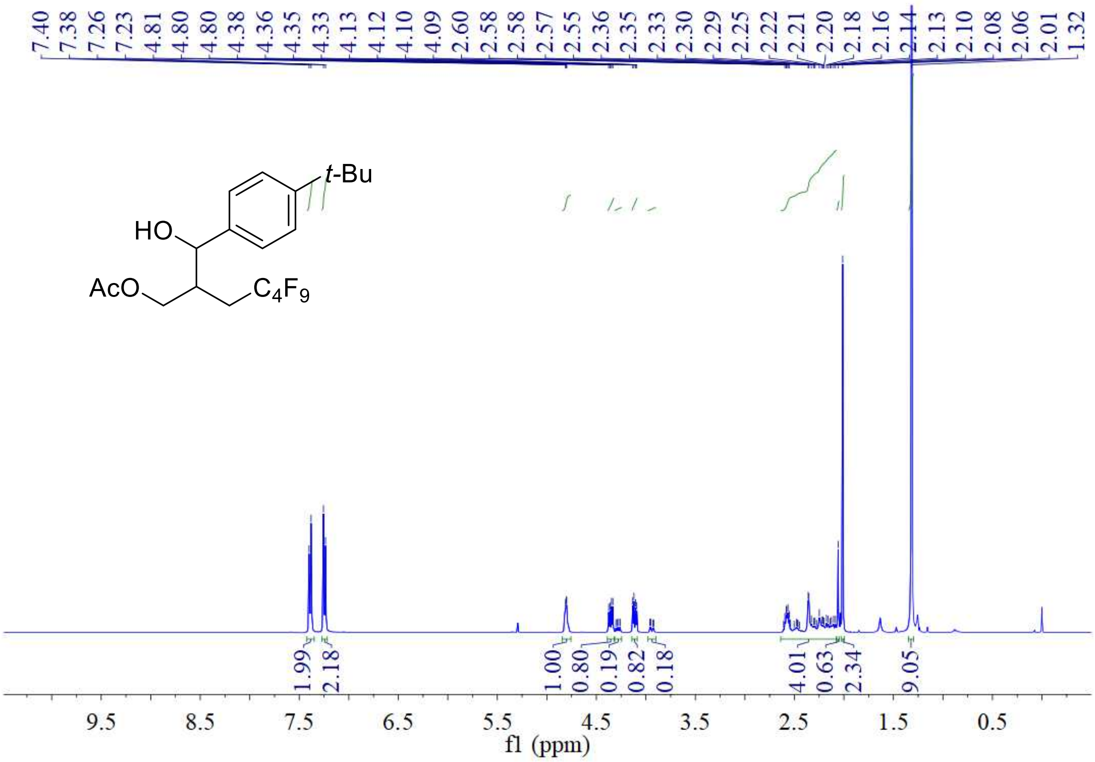

^{19}F NMR (375 MHz, $CDCl_3$)

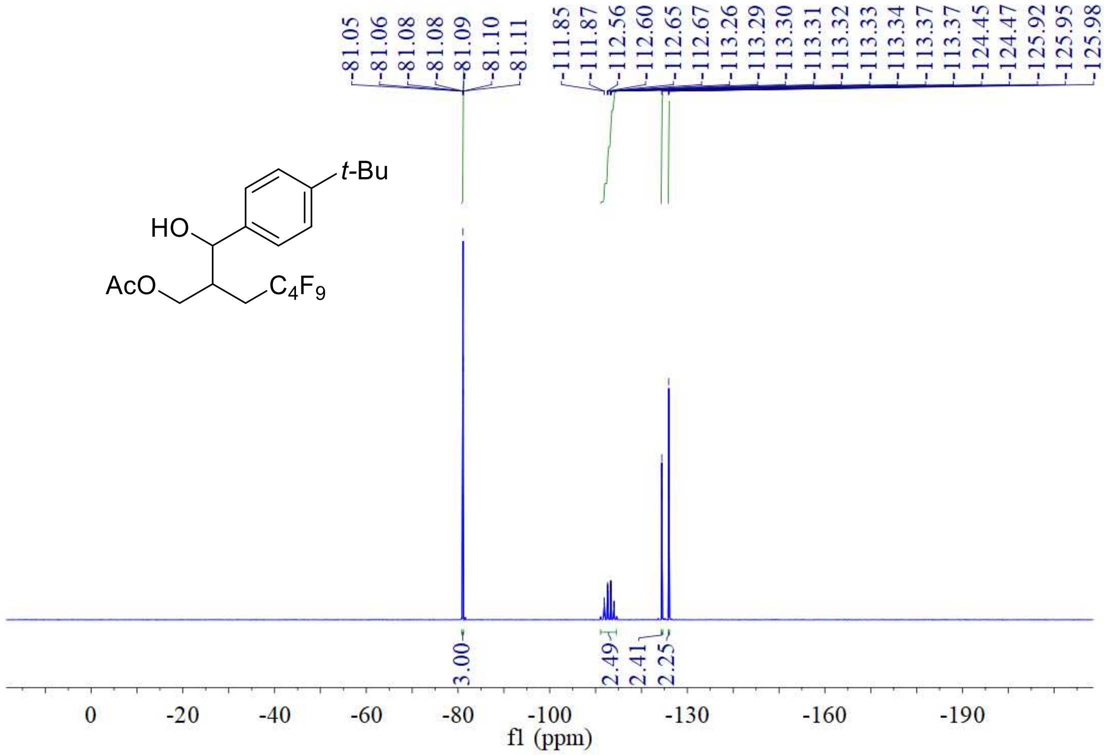

^{13}C NMR (100 MHz, $CDCl_3$)

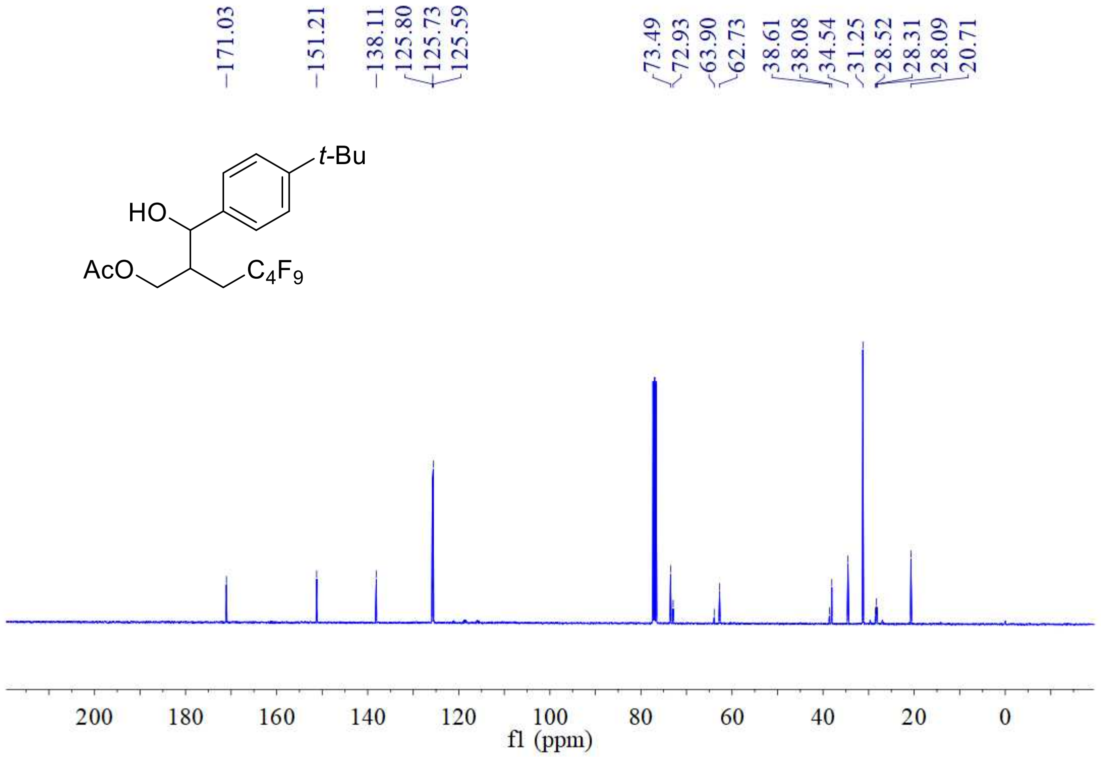

Supplementary Figure 76: NMR Spectra of 2-((4-(tert-Butyl)phenyl)(hydroxy)methyl)-4,4,5,5,6,6,7,7,7-nonafluoroheptyl acetate (48)

^{1}H NMR (400 MHz, $CDCl_3$)

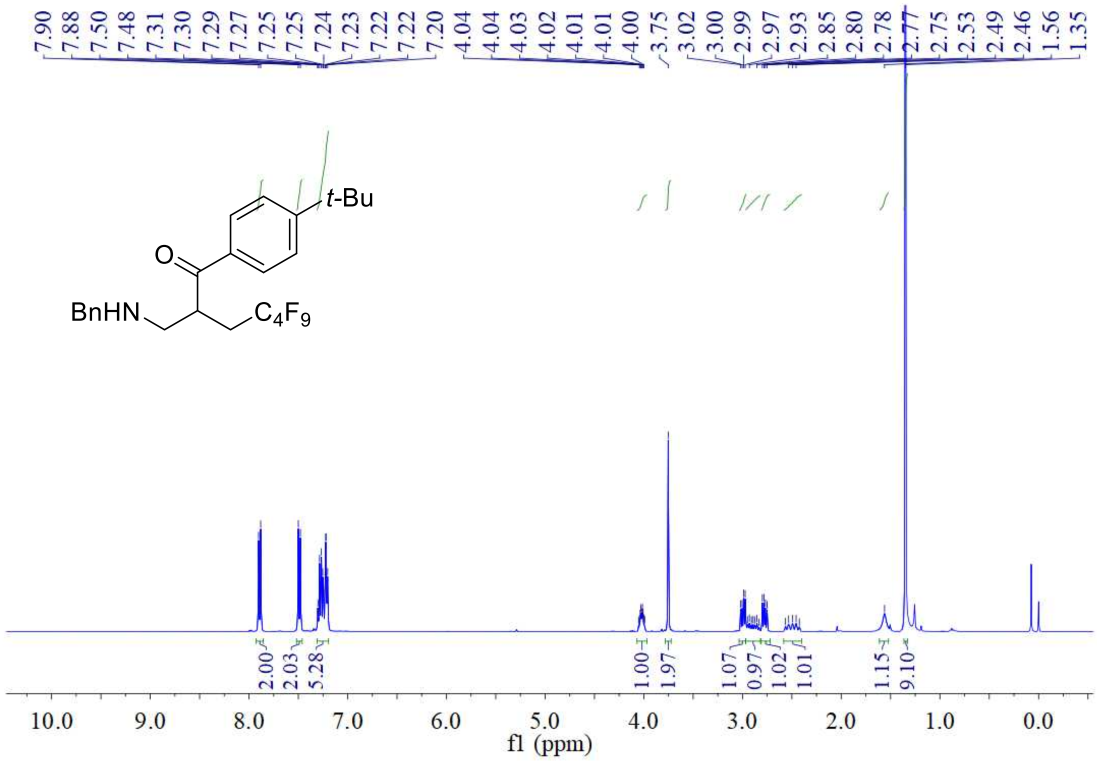

^{19}F NMR (375 MHz, $CDCl_3$)

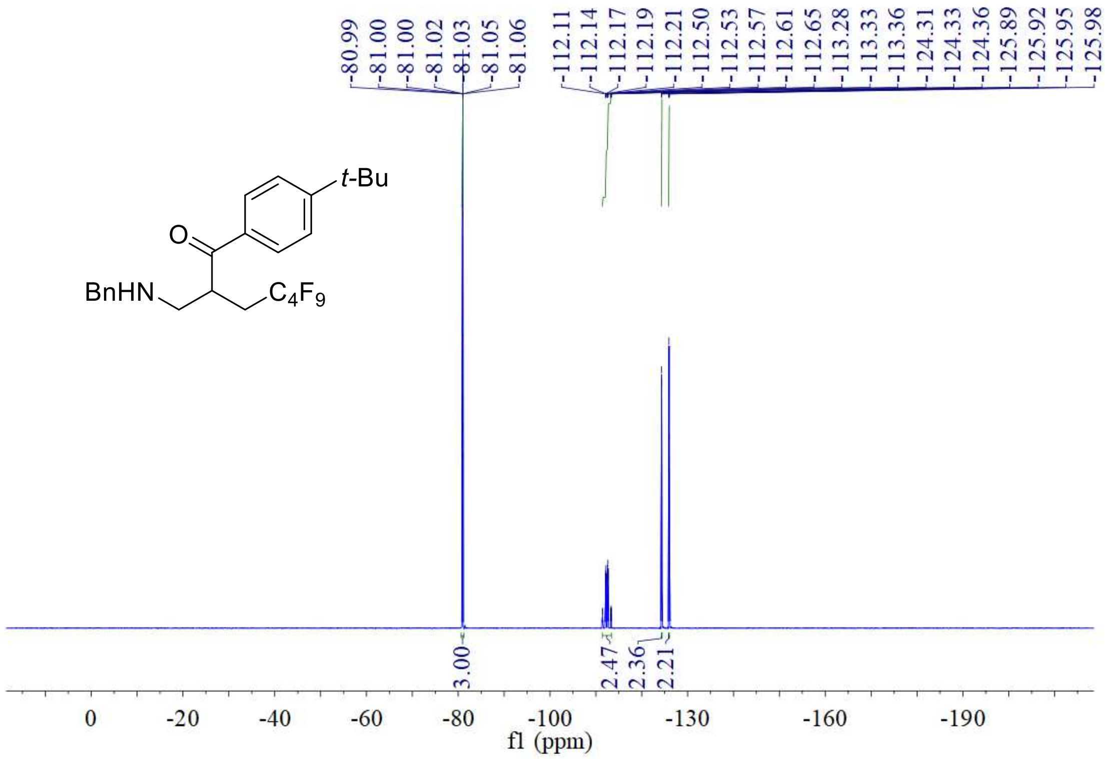

^{13}C NMR (100 MHz, $CDCl_3$)

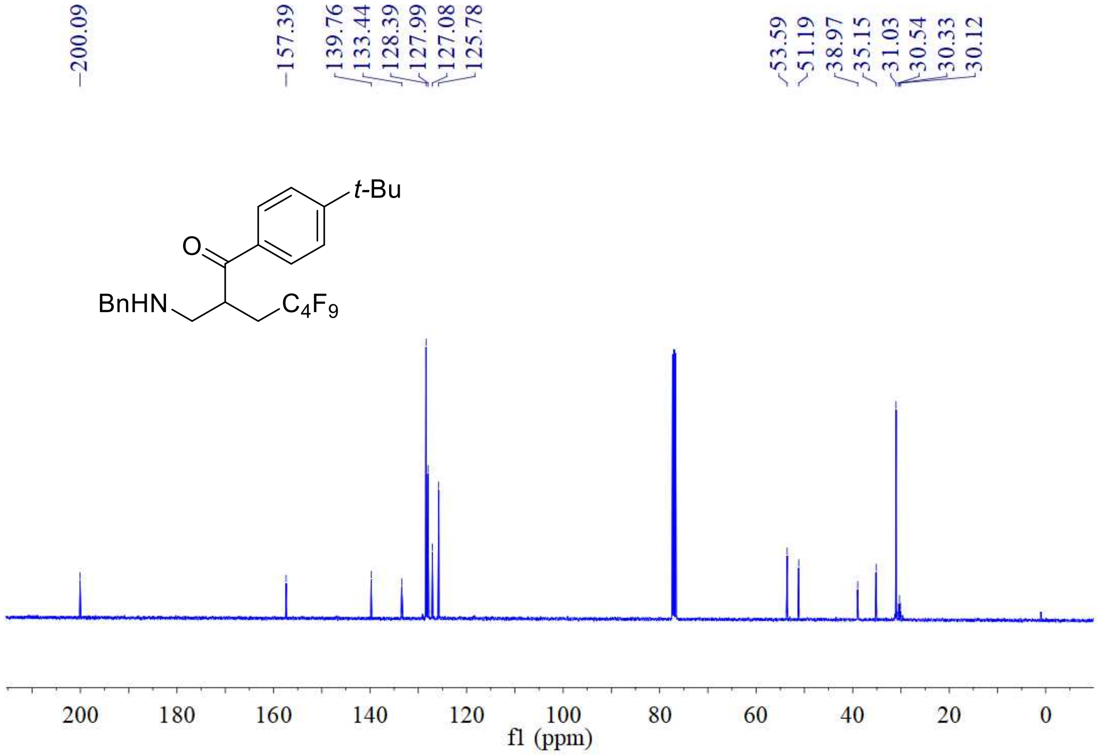

Supplementary Figure 77: NMR Spectra of 2-((Benzylamino)methyl)-1-(4-(tert-butyl)phenyl)-4,4,5,5,6,6,7,7,7-nonafluoroheptan-1-one (49)

^{1}H NMR (400 MHz, $CDCl_3$)

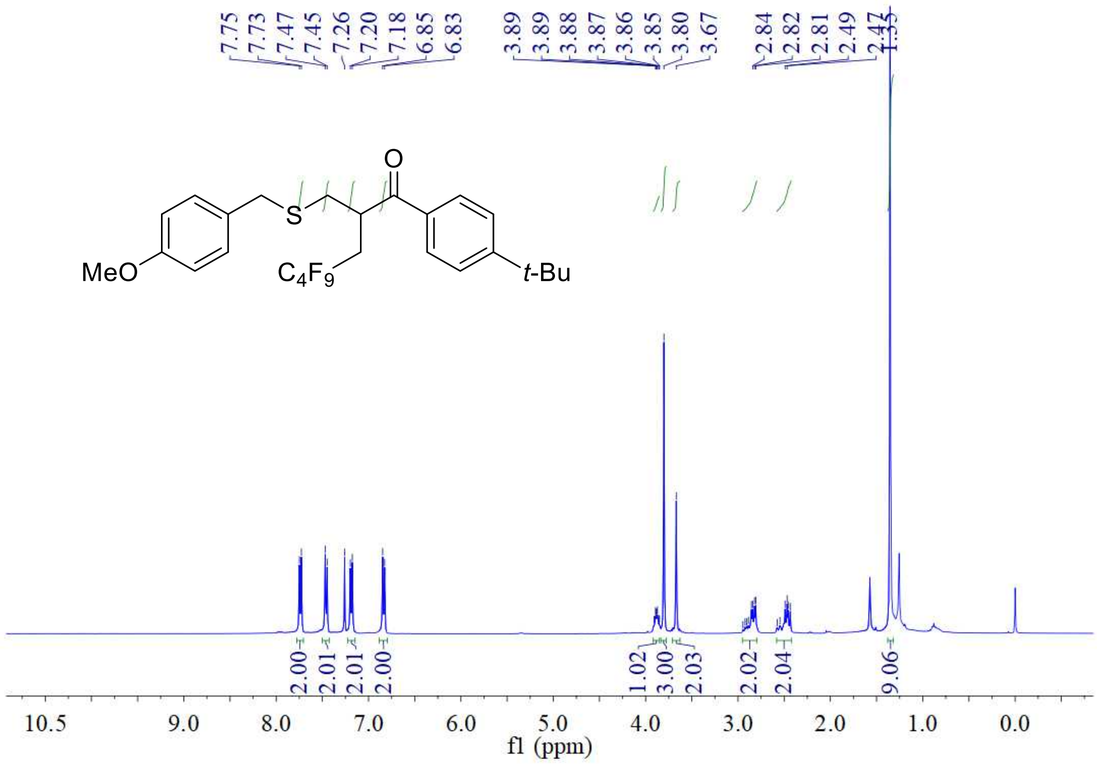

^{19}F NMR (375 MHz, $CDCl_3$)

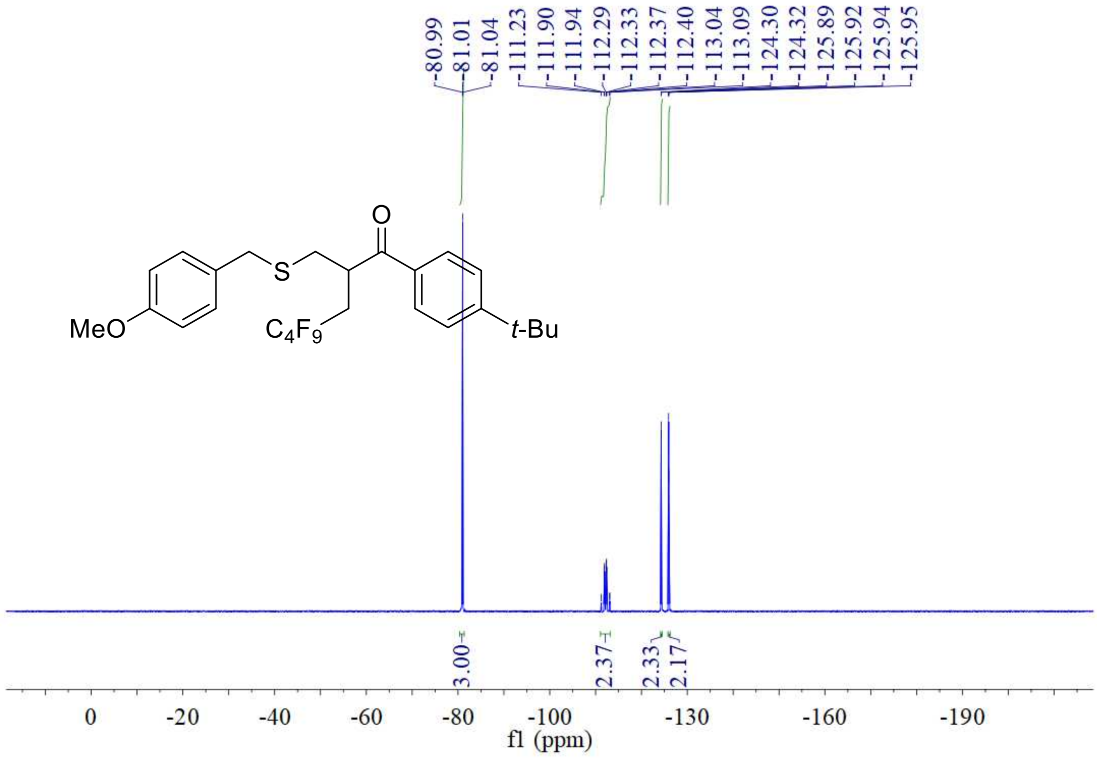

^{13}C NMR (150 MHz, $CDCl_3$)

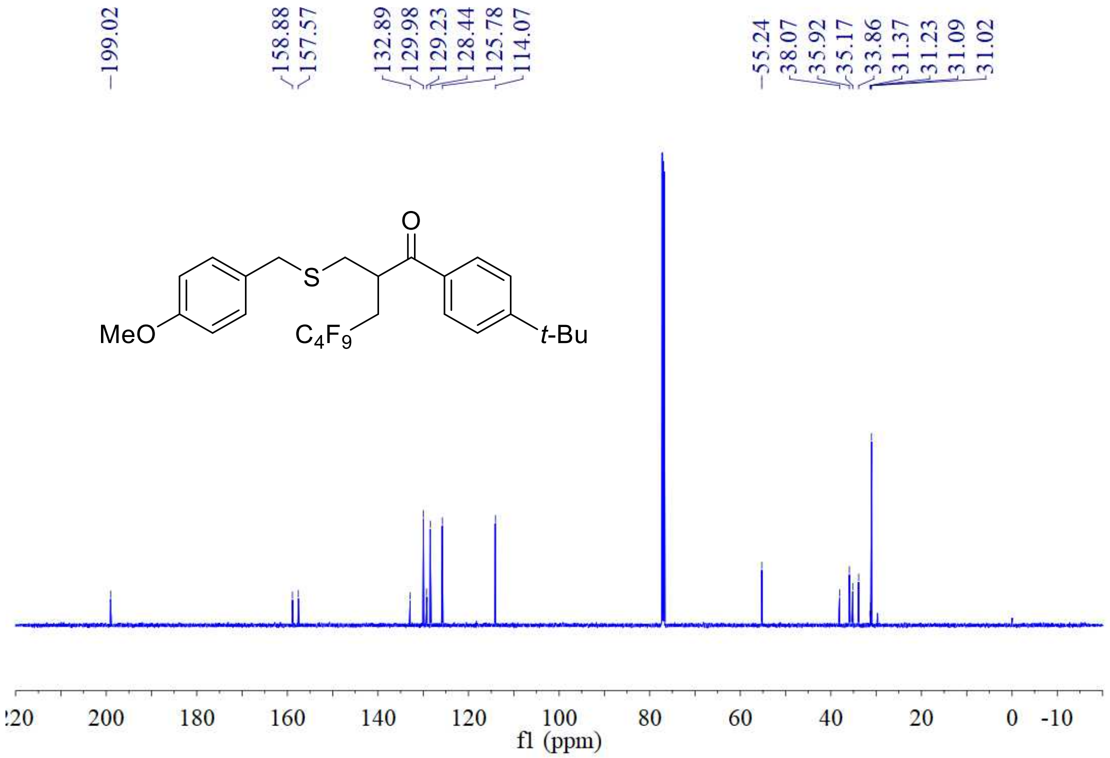

Supplementary Figure 78: NMR Spectra of 1-(4-(tert-Butyl)phenyl)-4, 4,5,5,6,6,7,7,7-nonafluoro-2-(((4-methoxybenzyl)thio)methyl)heptan-1-one (50)

^{1}H NMR (600 MHz, $CDCl_3$)

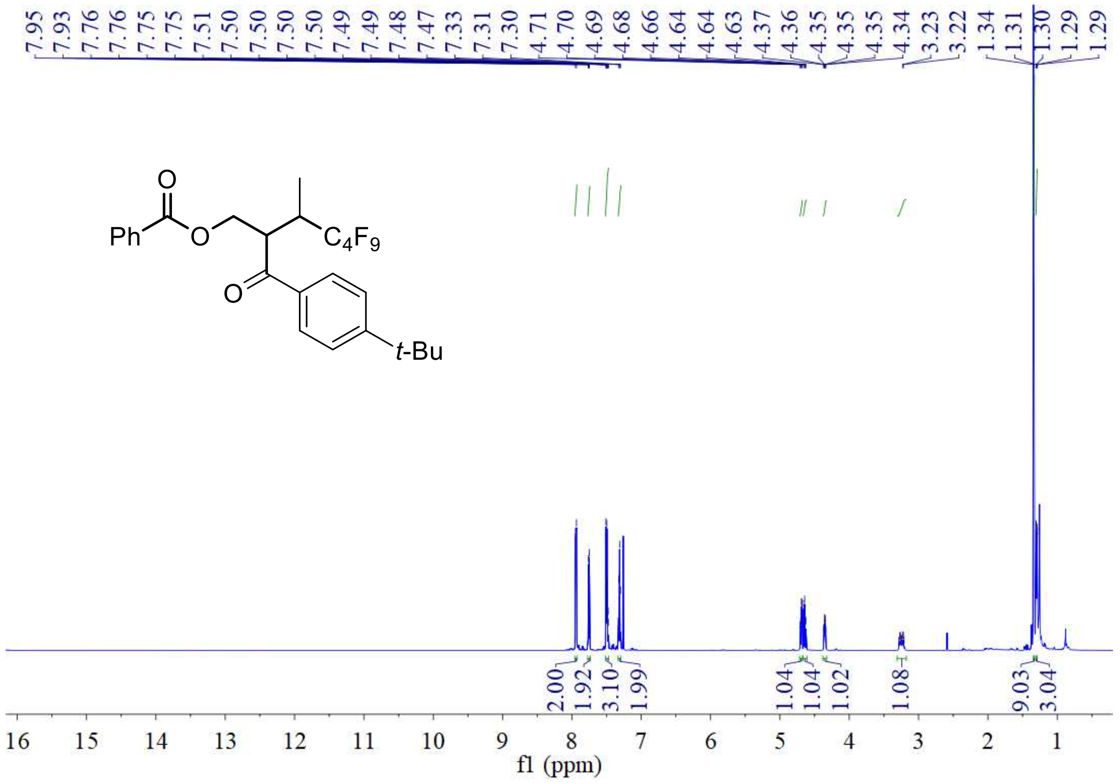

^{19}F NMR (564 MHz, $CDCl_3$)

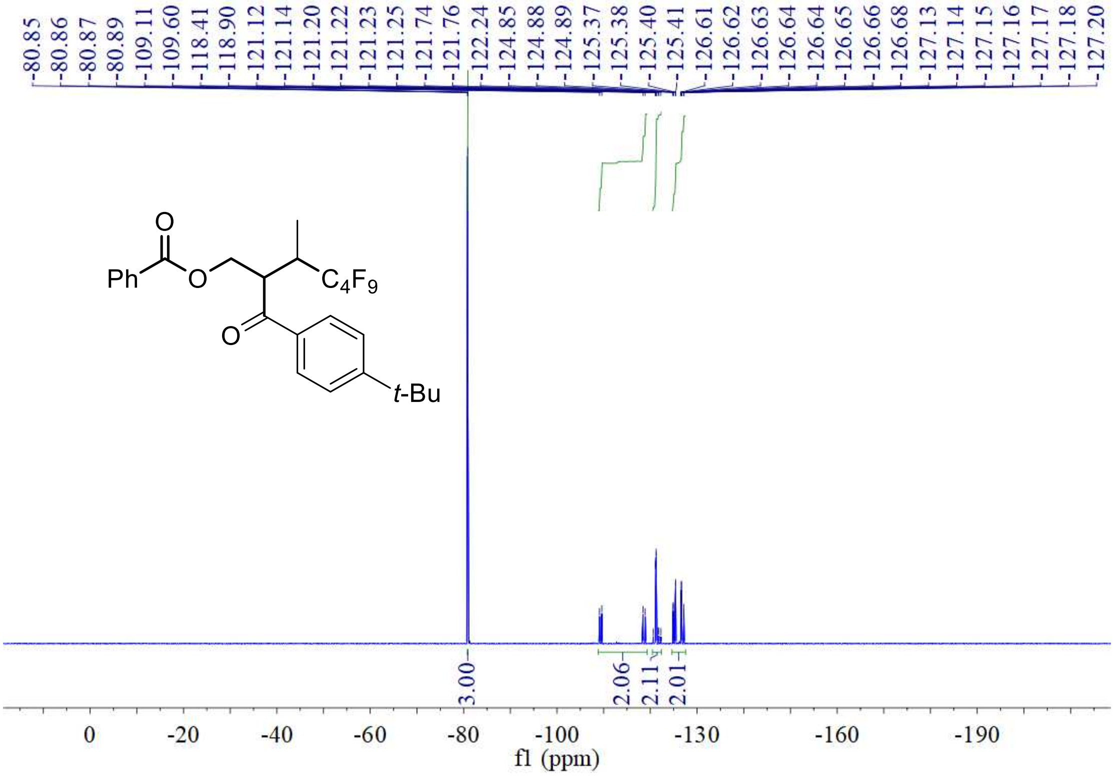

^{13}C NMR (150 MHz, $CDCl_3$)

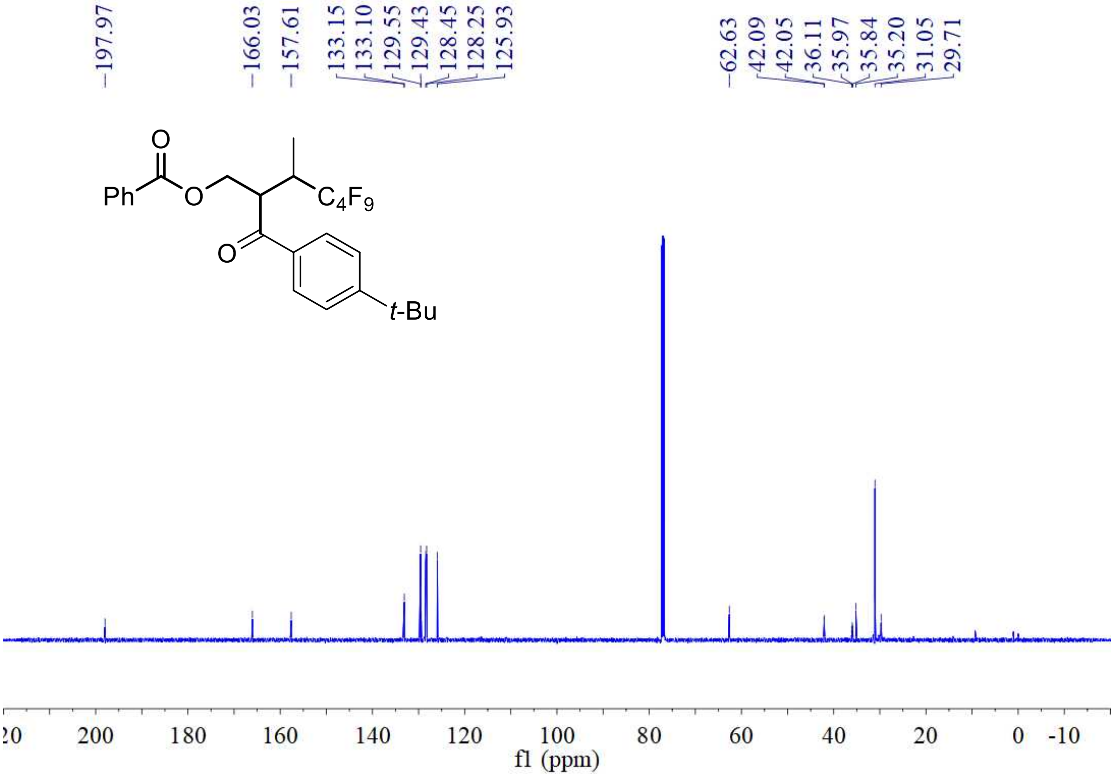

Supplementary Figure 79: NMR Spectra of 2-(4-(tert-Butyl)benzoyl) -4,4,5,5,6,6,7,7,7-nonafluoro-3-methylheptyl benzoate (S9)

^{1}H NMR (600 MHz, $CDCl_3$)

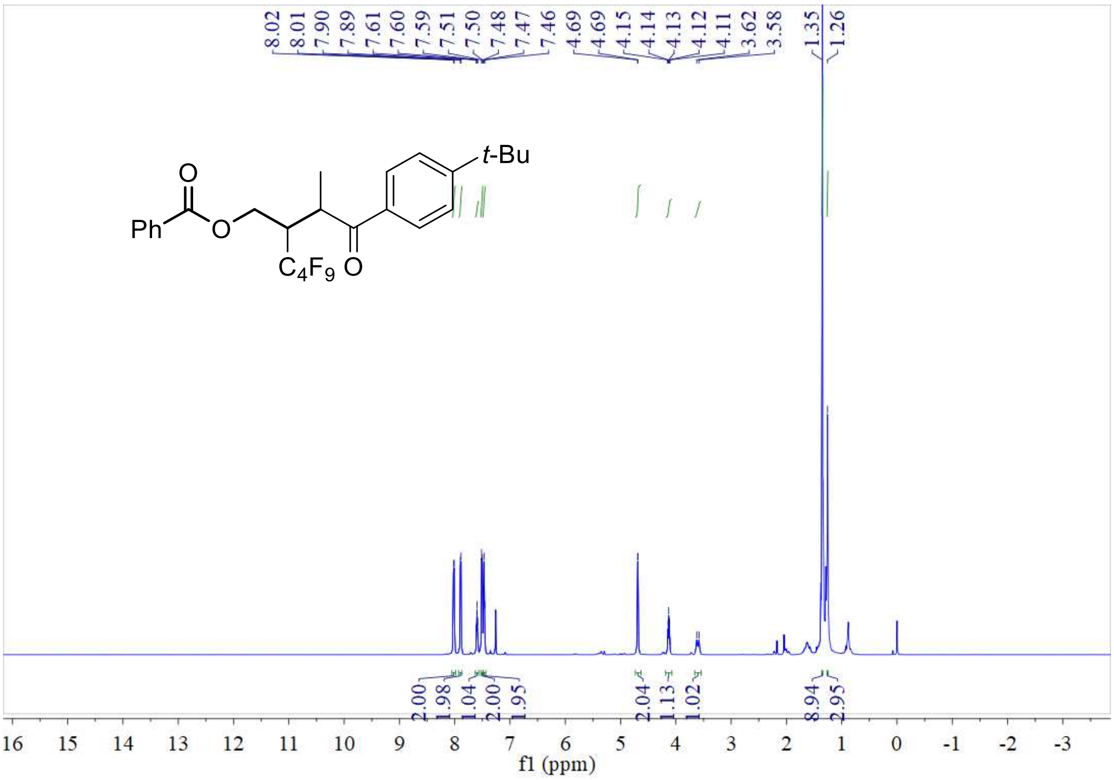

^{19}F NMR (564 MHz, $CDCl_3$)

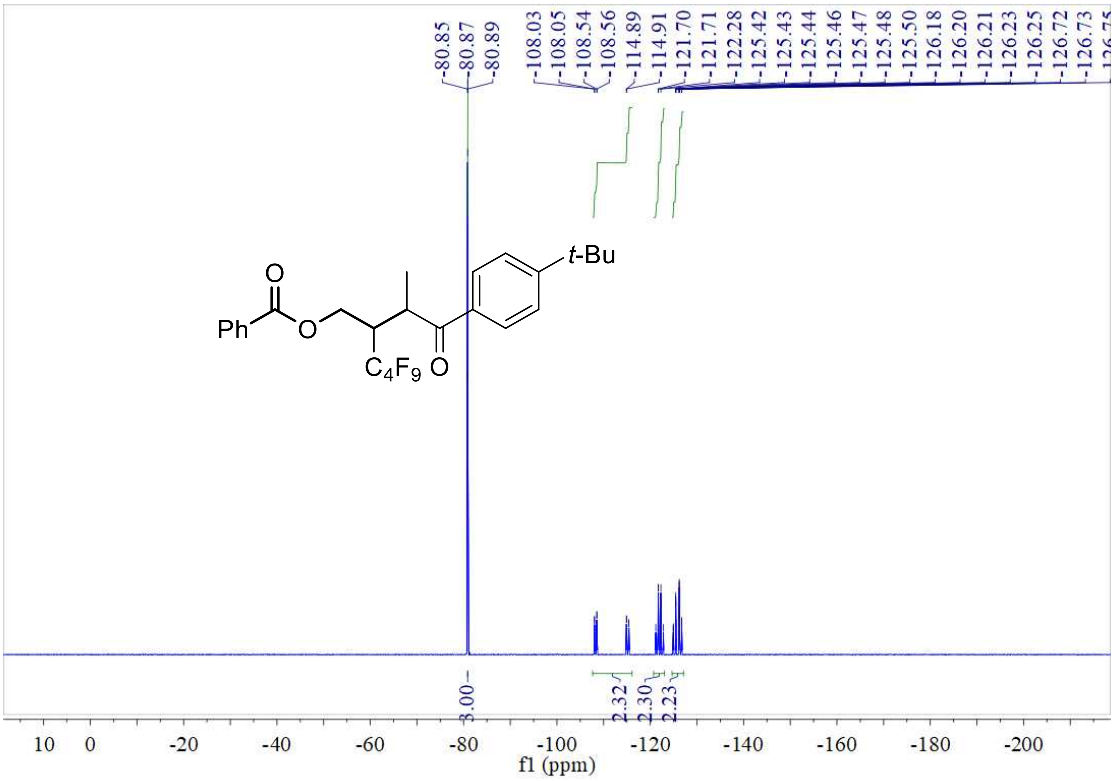

^{13}C NMR (150 MHz, $CDCl_3$)

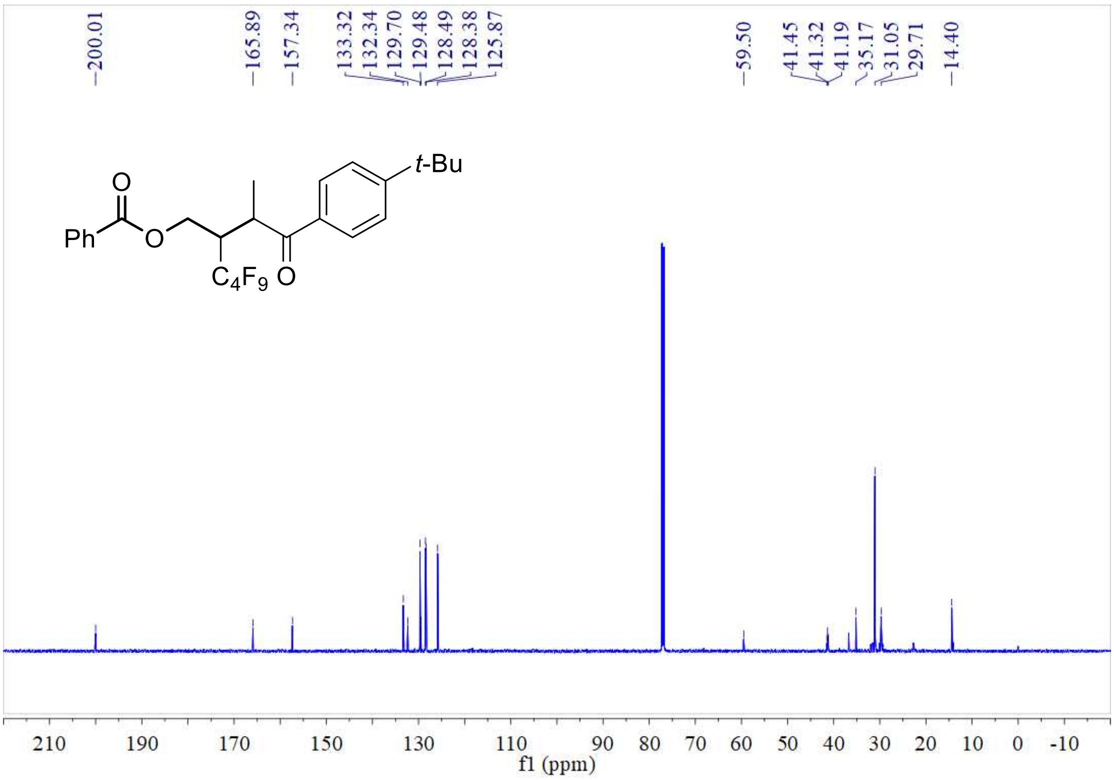

Supplementary Figure 80: NMR Spectra of 2-(1-(4-(tert-Butyl)phenyl) -1-oxopropan-2-yl)-3,3,4,4,5,5,6,6,6-nonafluorohexyl benzoate (S10)

^{1}H NMR (600 MHz, $CDCl_3$)

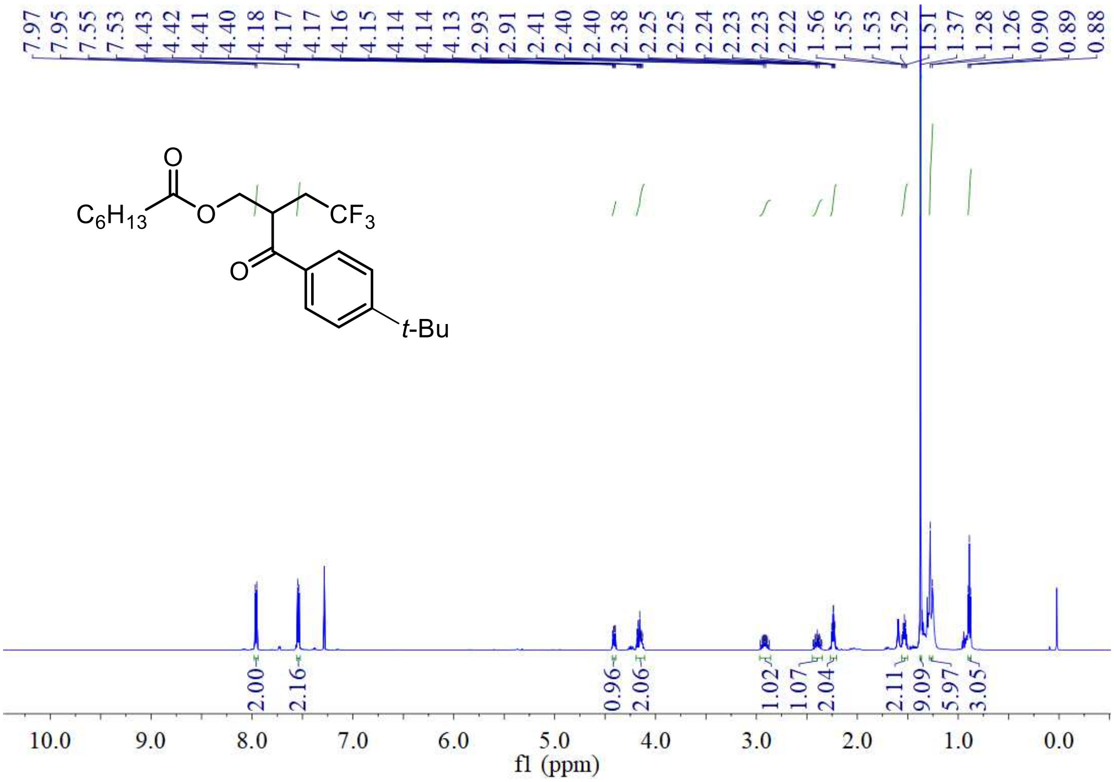

^{19}F NMR (564 MHz, $CDCl_3$)

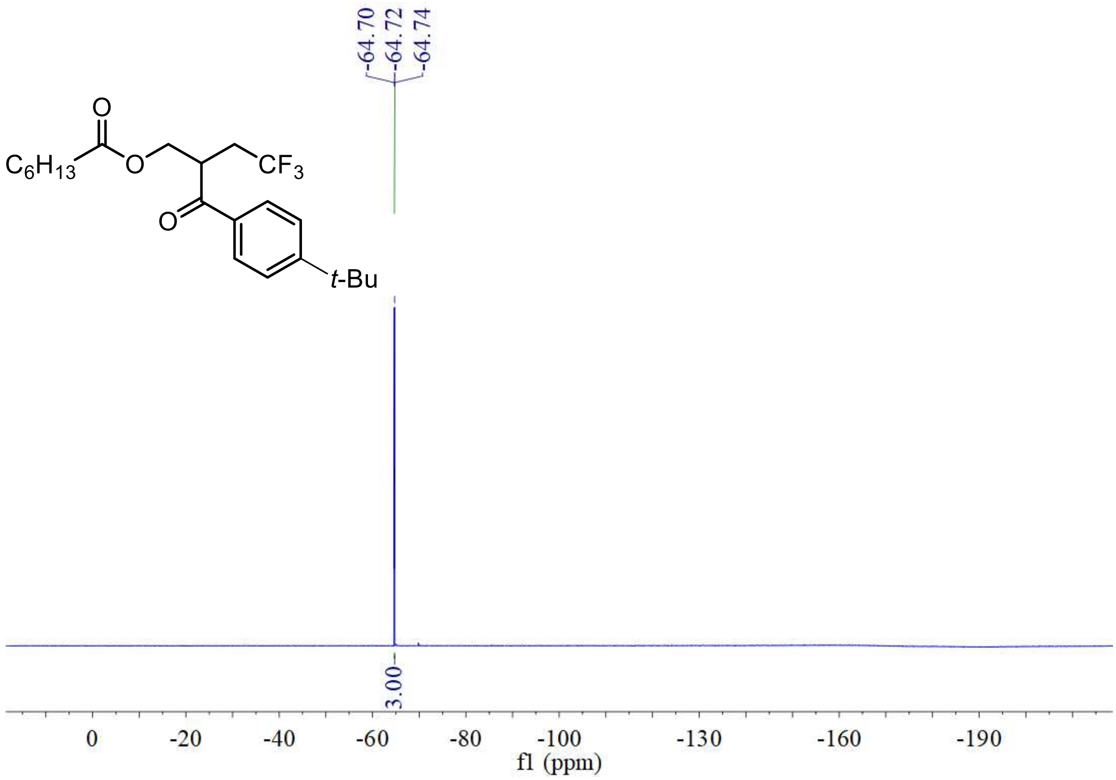

^{13}C NMR (150 MHz, $CDCl_3$)

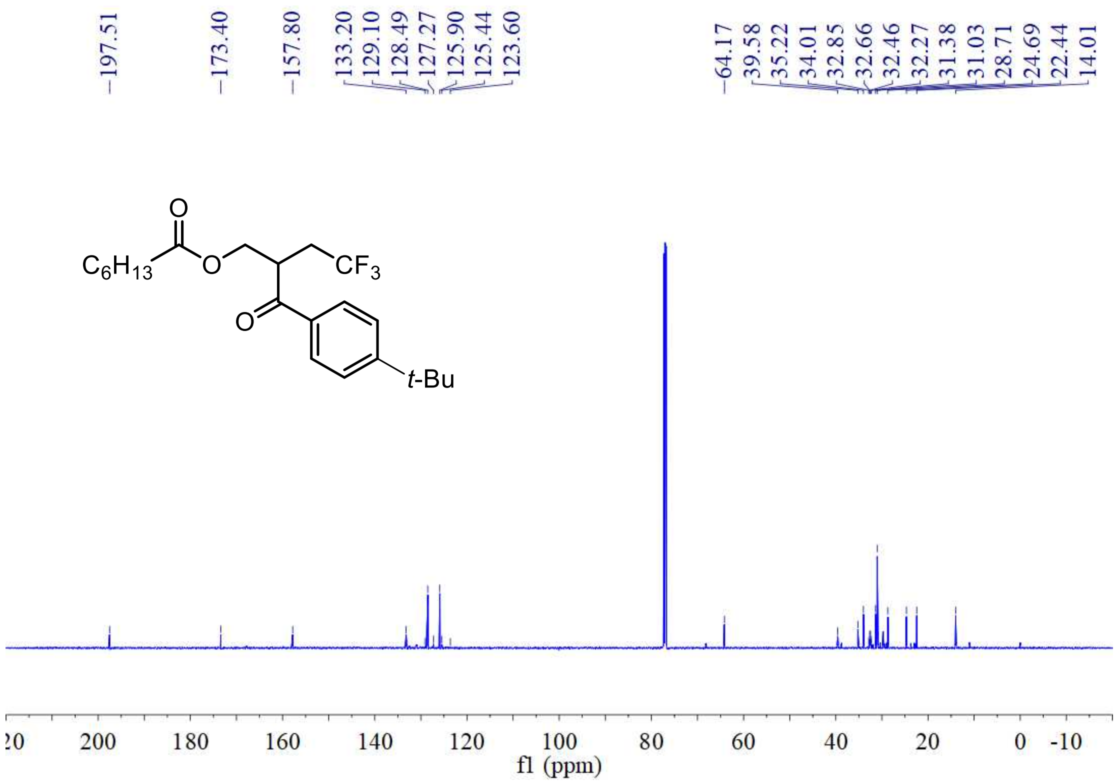

Supplementary Figure 81: NMR Spectra of 2-(4-(tert-Butyl)benzoyl) -4,4,4-trifluorobutyl heptanoate (S11)

^{1}H NMR (600 MHz, $CDCl_3$)

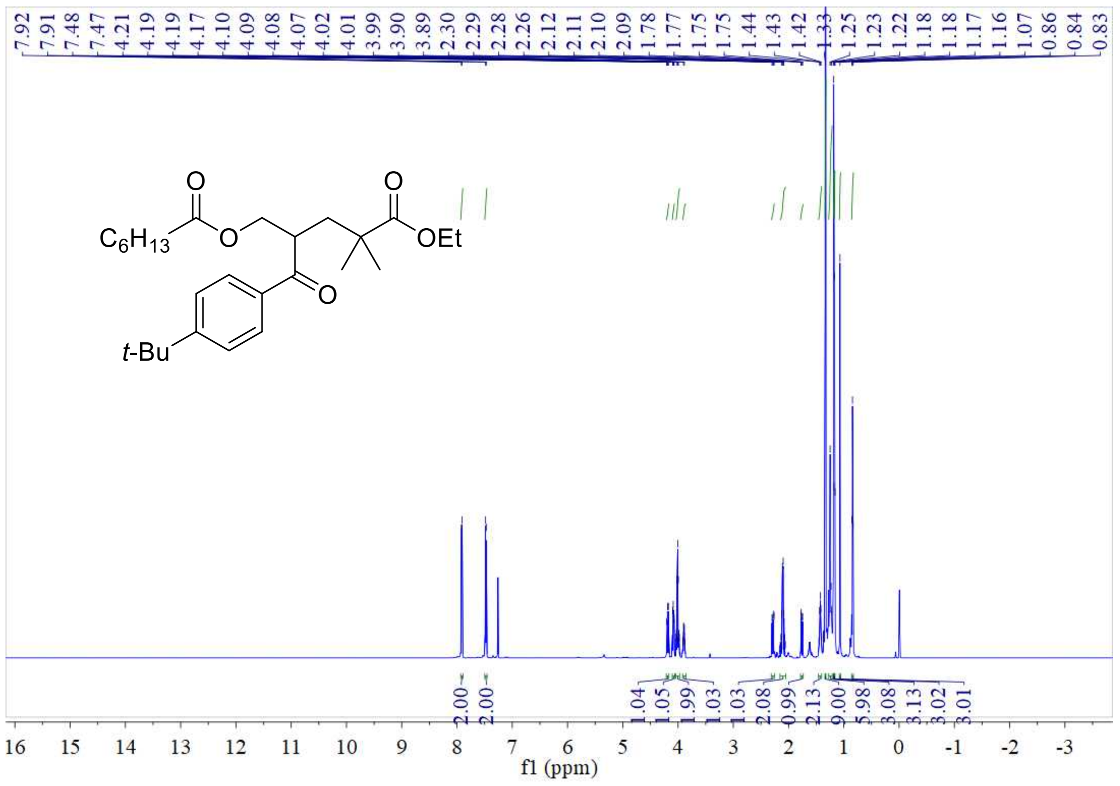

^{13}C NMR (150 MHz, $CDCl_3$)

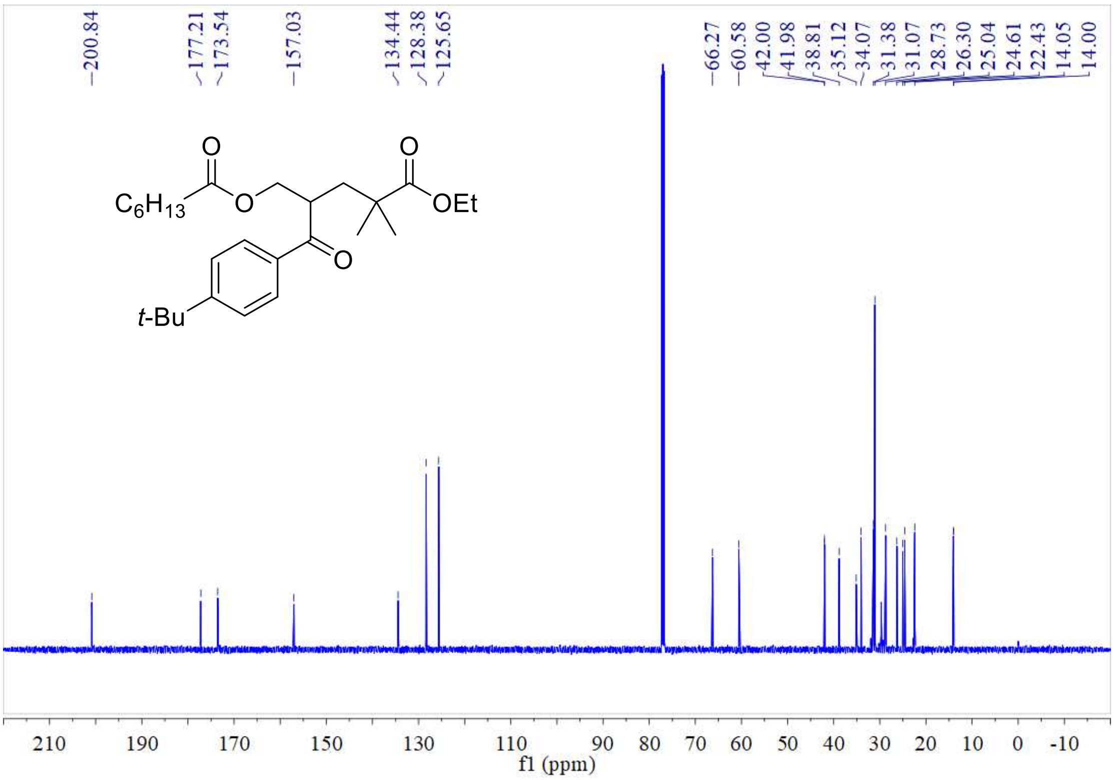

Supplementary Figure 82: NMR Spectra of 2-(4-(tert-Butyl)benzoyl)-5-ethoxy-4,4-dimethyl-5-oxopentyl heptanoate (S12)

^{1}H NMR (600 MHz, $CDCl_3$)

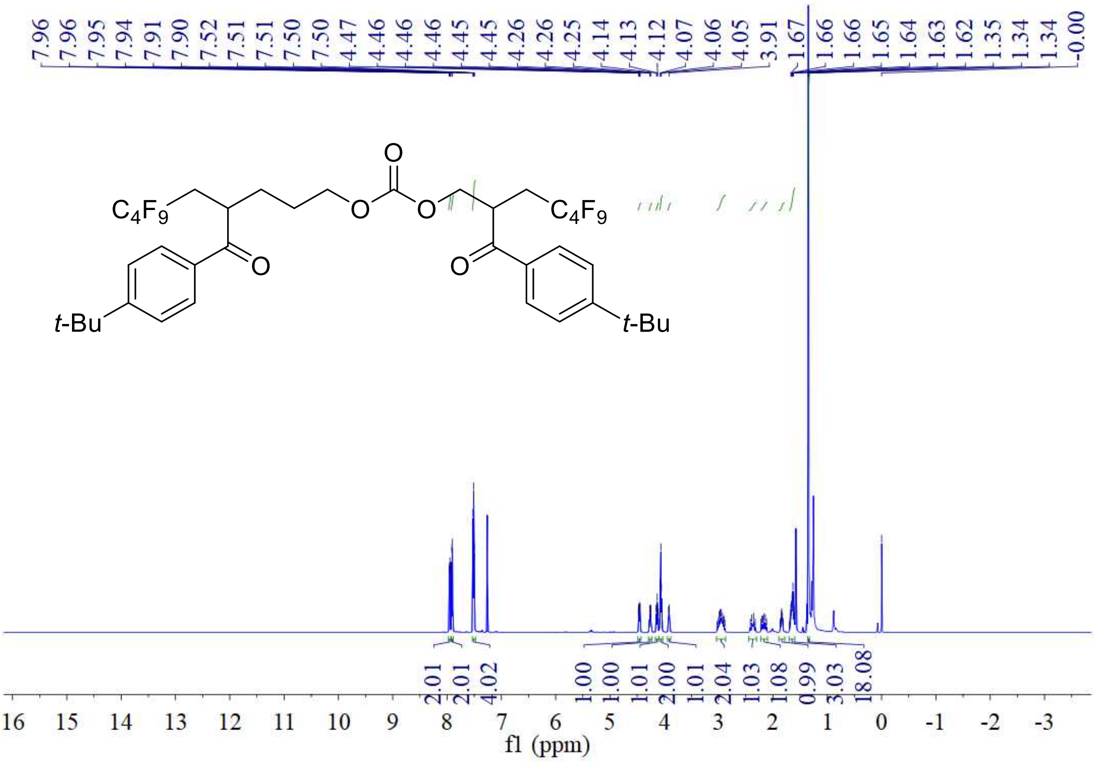

^{19}F NMR (564 MHz, $CDCl_3$)

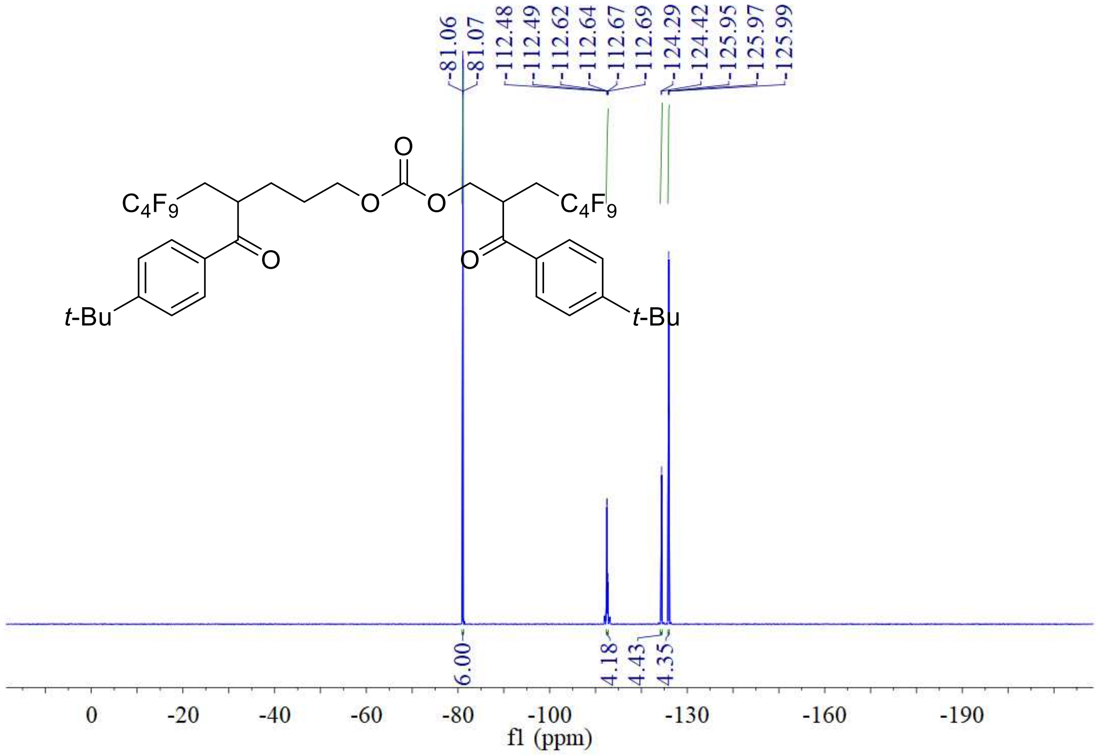

^{13}C NMR (150 MHz, $CDCl_3$)

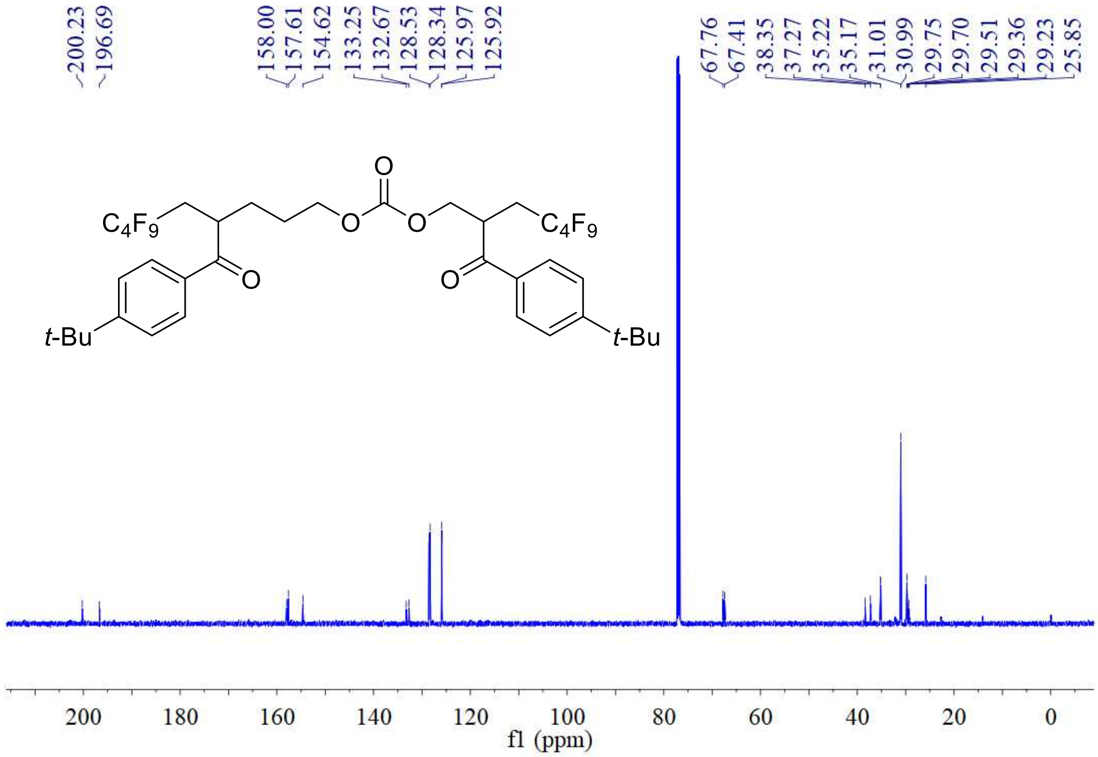

Supplementary Figure 83: NMR Spectra of 2-(4-(tert-Butyl)benzoyl)-4,4,5,5,6,6,7,7,7-nonafluoroheptyl (4-(4-(tert-butyl) benzoyl)-6,6,7,7,8,8,9,9,9-nonafluorononyl) carbonate (S13)

^{1}H NMR (600 MHz, $CDCl_3$)

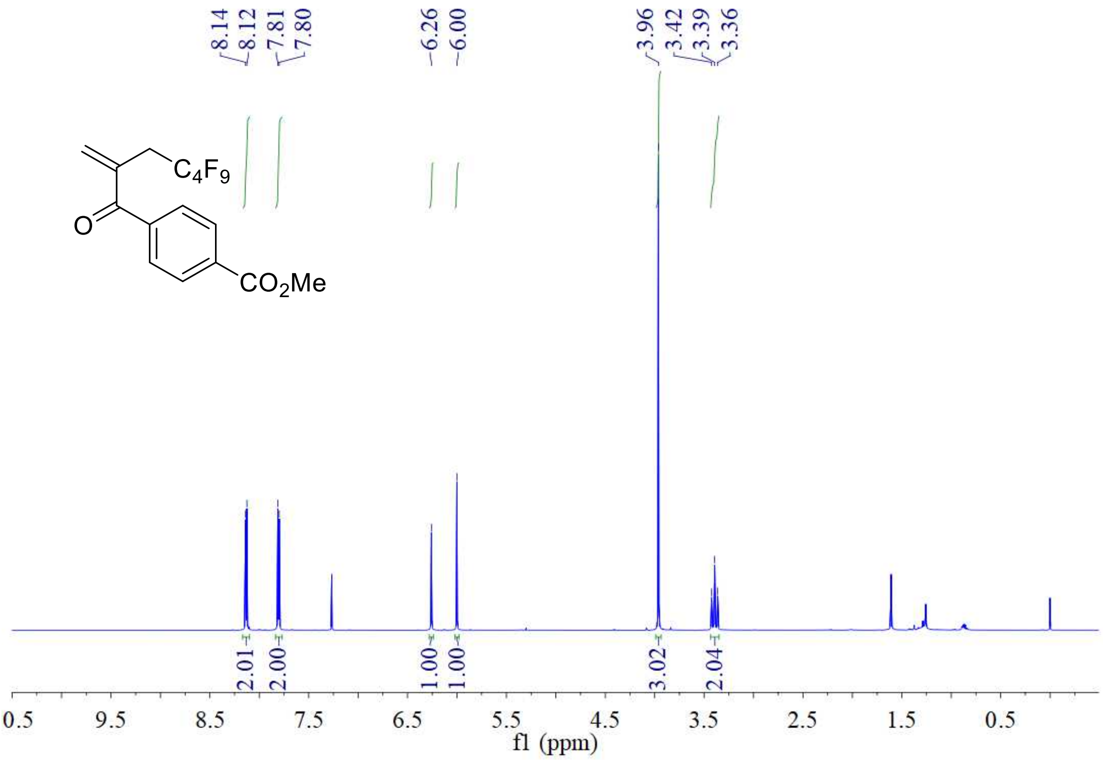

^{19}F NMR (564 MHz, $CDCl_3$)

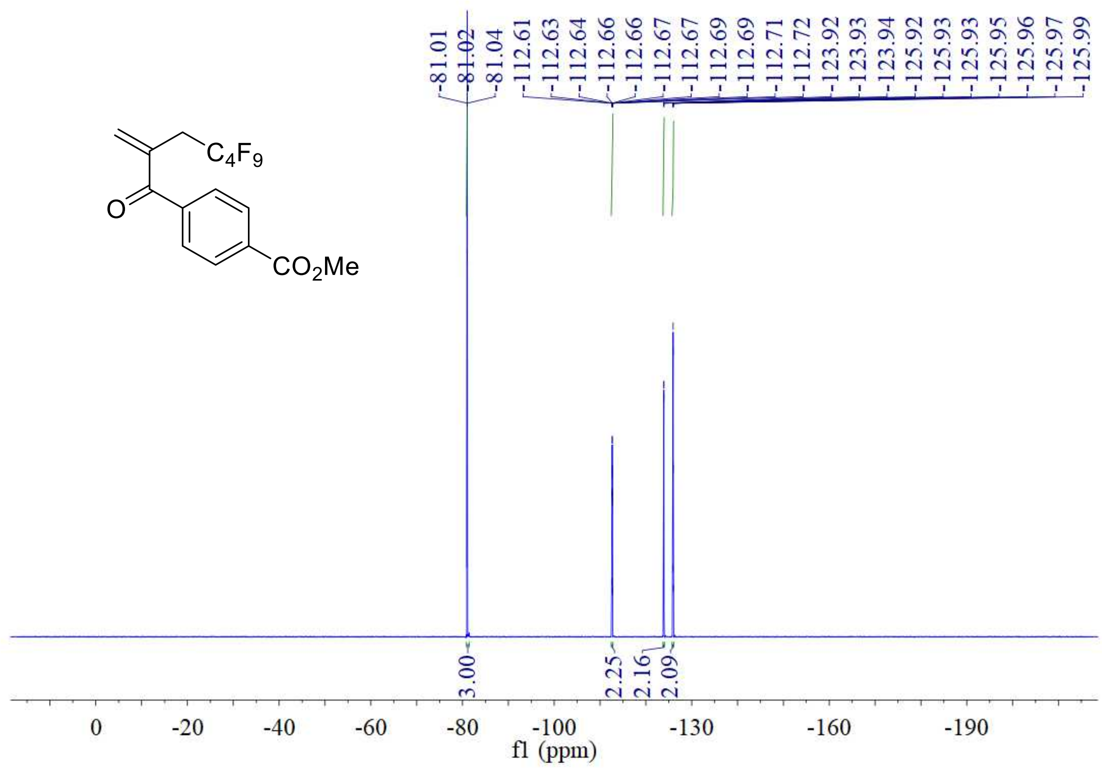

^{13}C NMR (150 MHz, $CDCl_3$)

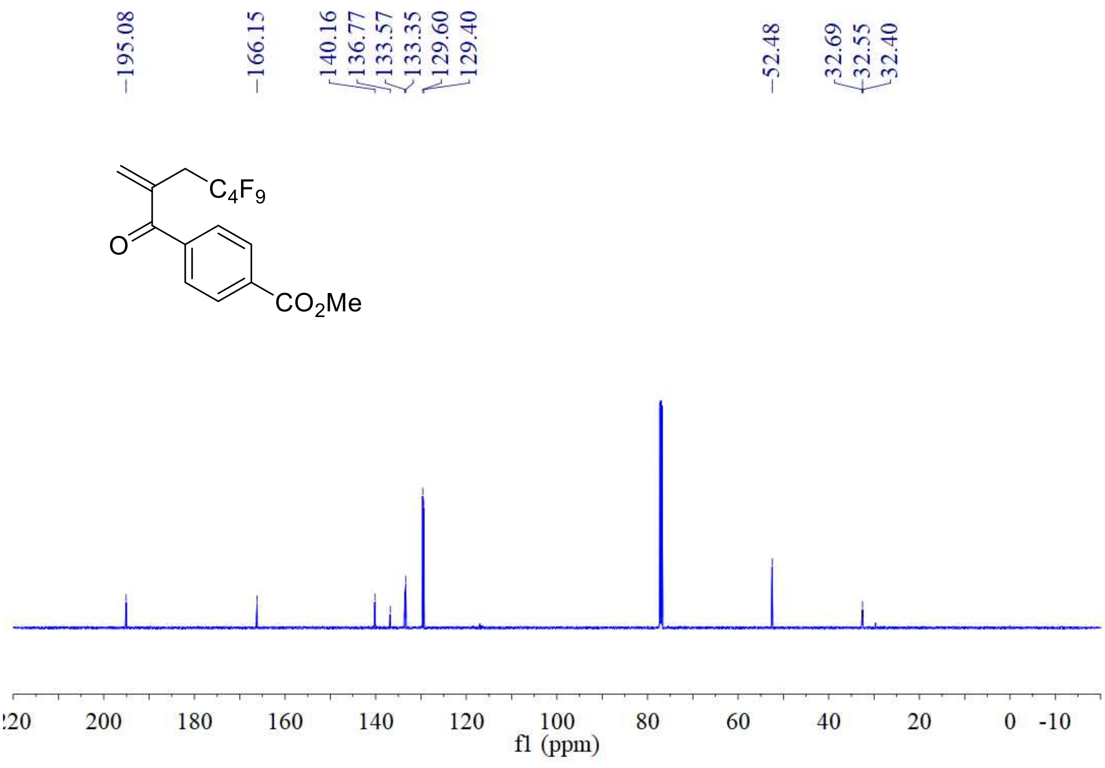

Supplementary Figure 84: NMR Spectra of Methyl 4-(4,4,5,5,6,6,7,7,7-nonafluoro-2-methyleneheptanoyl)benzoate (S14)

^{1}H NMR (600 MHz, $CDCl_3$)

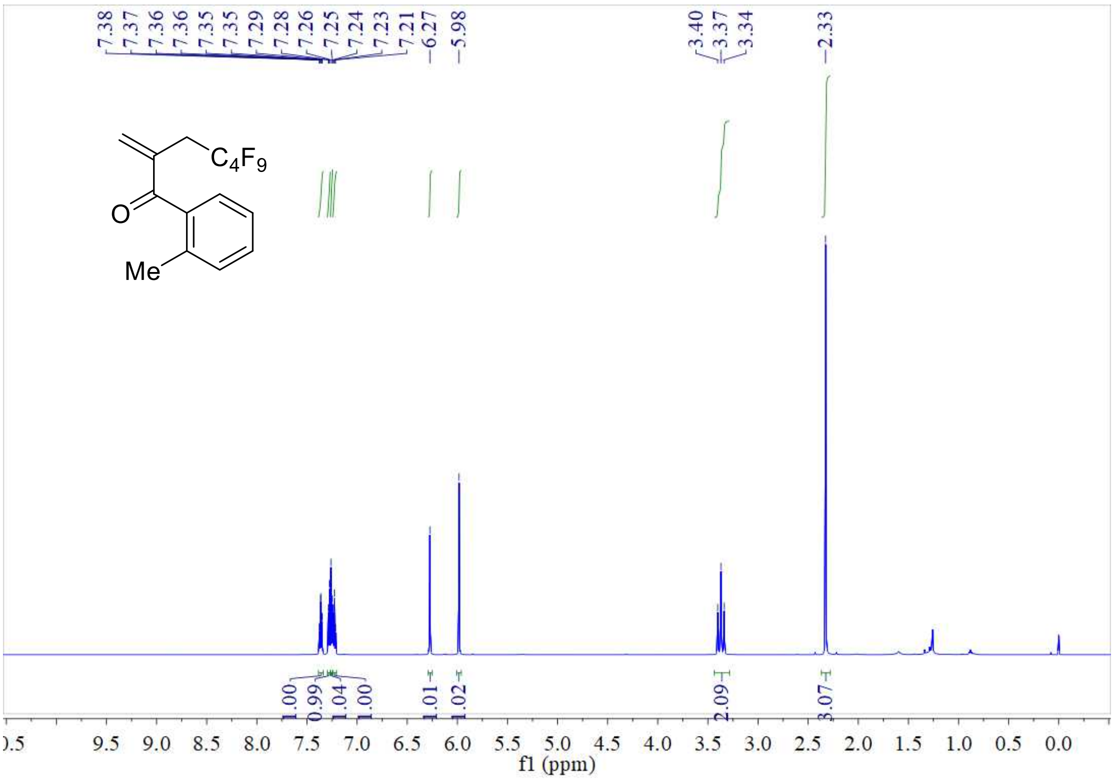

^{19}F NMR (564 MHz, $CDCl_3$)

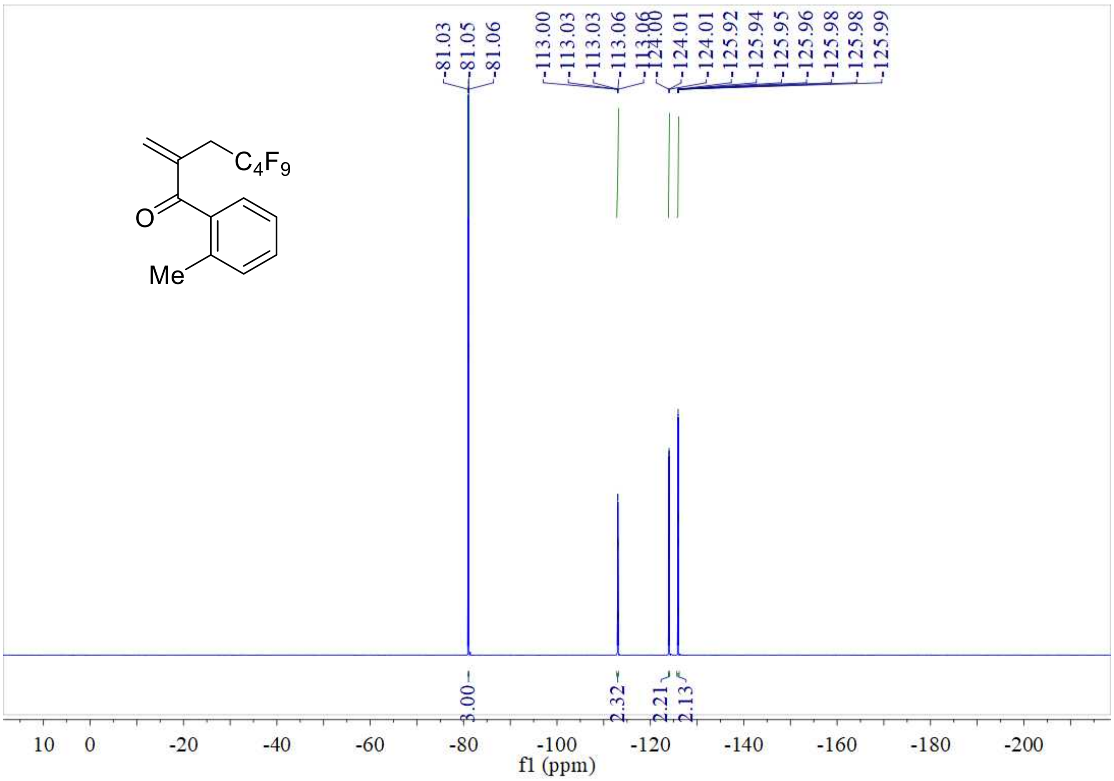

^{13}C NMR (150 MHz, $CDCl_3$)

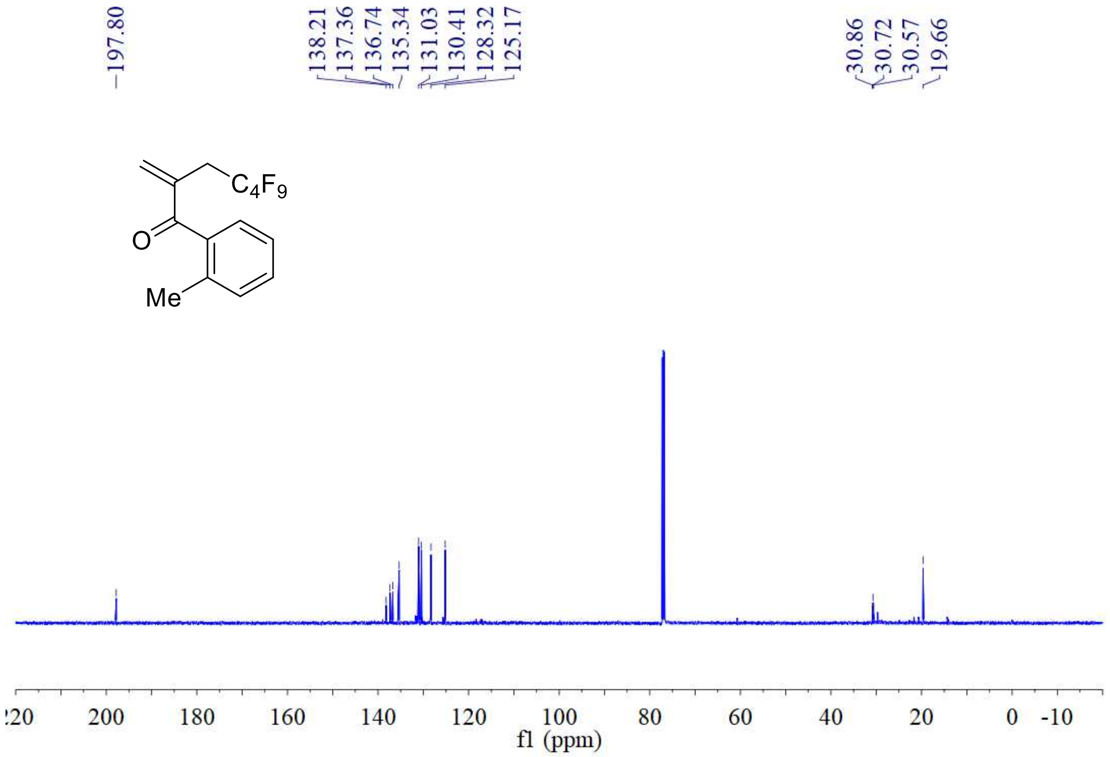

Supplementary Figure 85: NMR Spectra of 4,4,5,5,6,6,7,7,7-Nonafluoro-2-methylene-1-(o-tolyl)heptan-1-one (S15)

^{1}H NMR (400 MHz, $CDCl_3$)

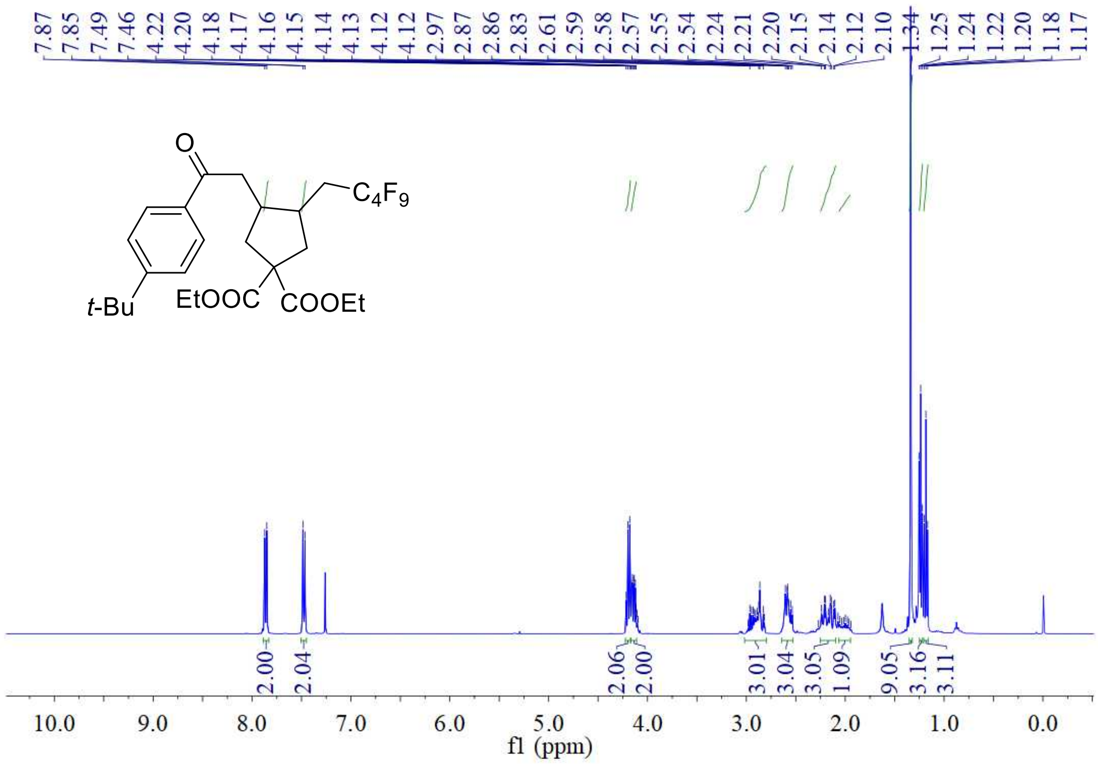

^{19}F NMR (564 MHz, $CDCl_3$)

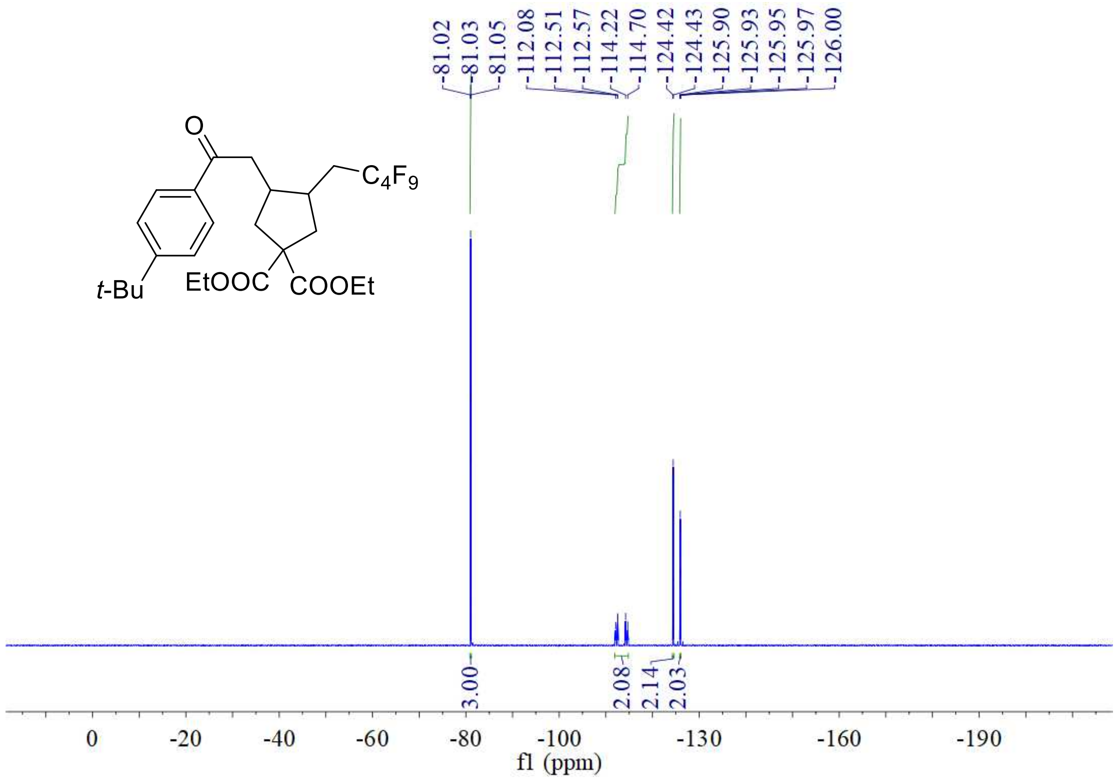

^{13}C NMR (100 MHz, $CDCl_3$)

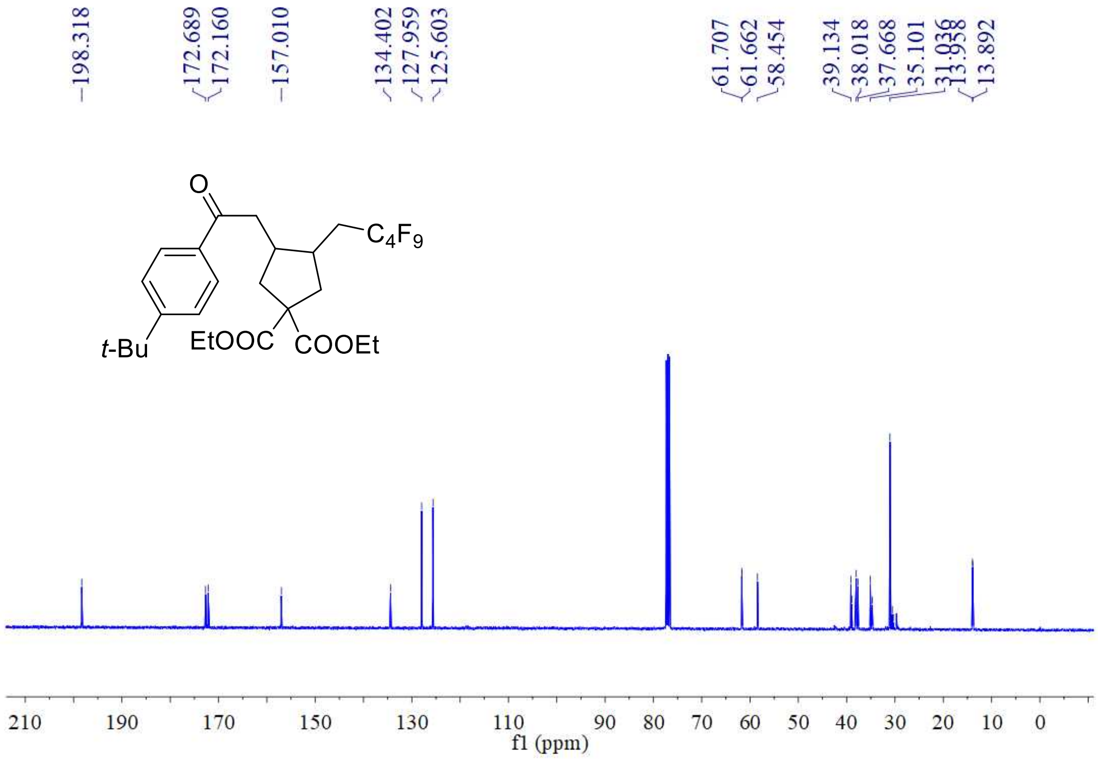

Supplementary Figure 86: NMR Spectra of Diethyl 3-(2-(4-(tert-butyl)phenyl)-2-oxoethyl)-4-(2,2,3,3,4,4,5,5,5-nonafluoropen-tyl)cyclopentane-1,1-dicarboxylate (53)

^{1}H NMR (600 MHz, $CDCl_3$)

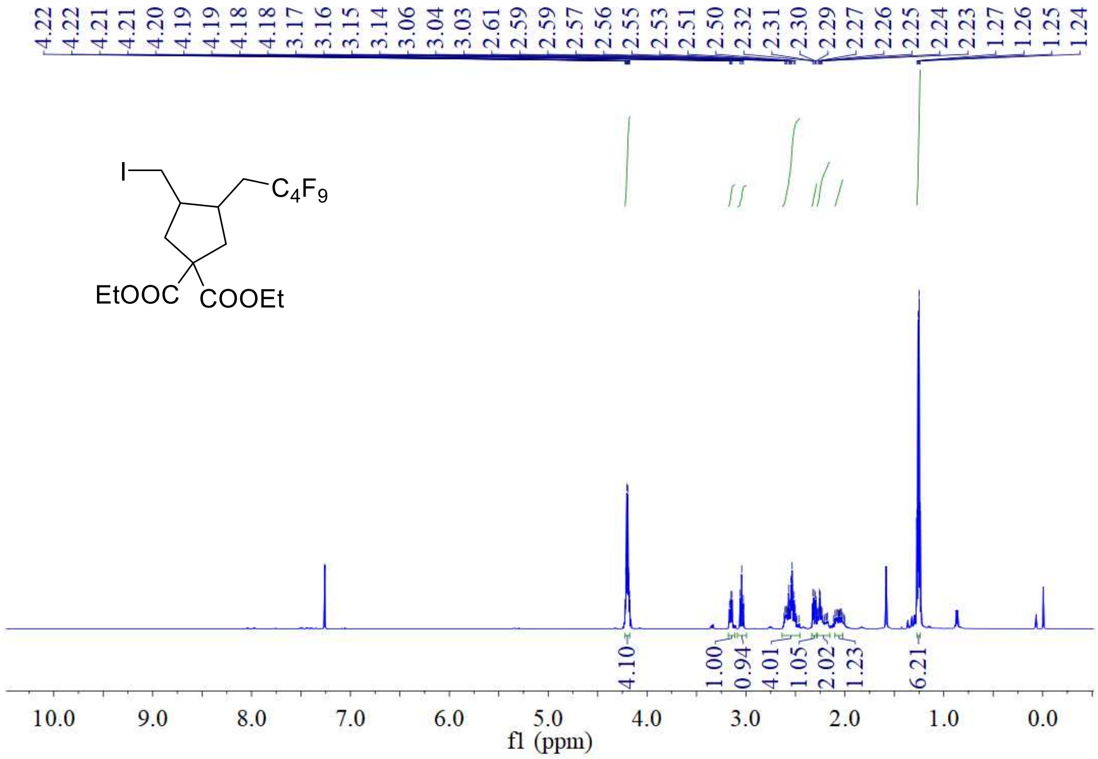

^{19}F NMR (375 MHz, $CDCl_3$)

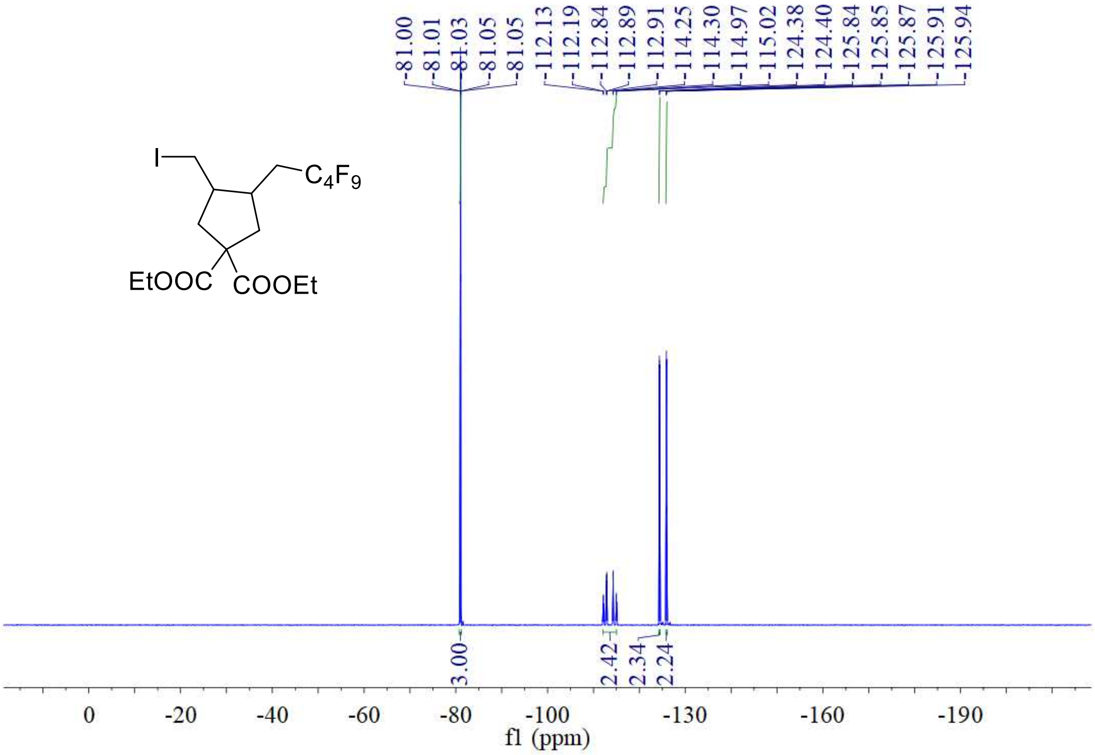

^{13}C NMR (150 MHz, $CDCl_3$)

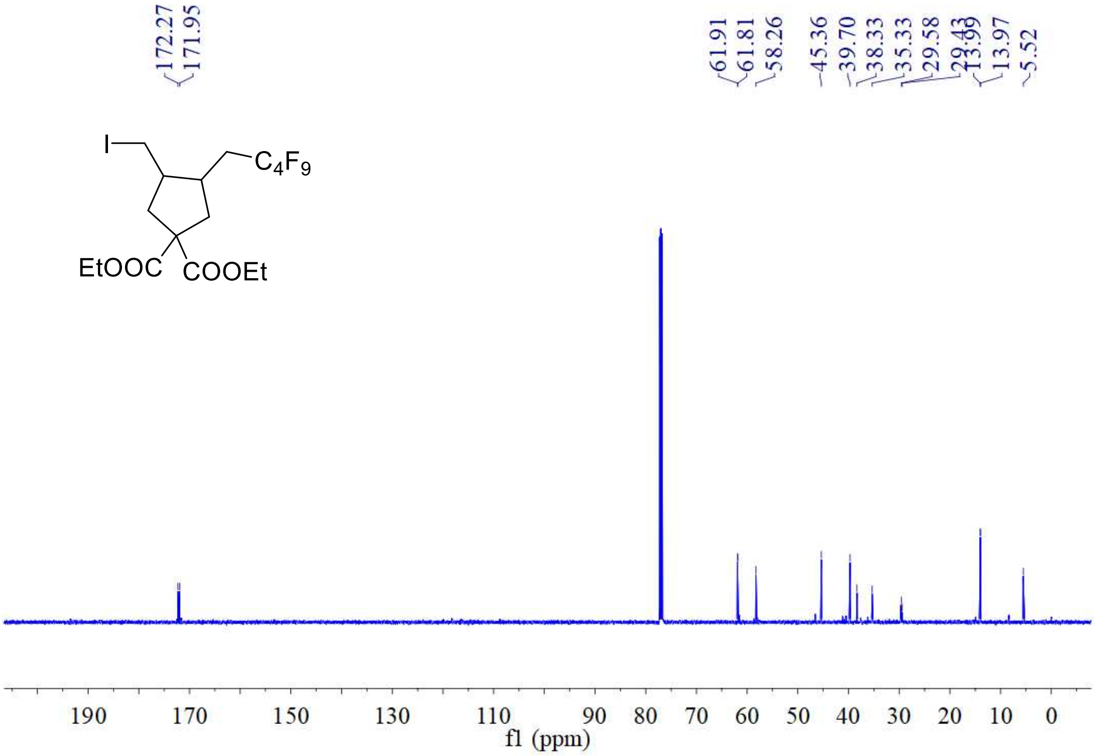

Supplementary Figure 87: NMR Spectra of Diethyl 3-(iodomethyl)-4-(2,2,3,3,4,4,5,5,5-nonafluoropentyl)cyclopentane-1,1-Dicarboxylate (54)

3. Supplementary References

(1) Dieskau, A. P., Plietker, B. A Mild Ligand-Free Iron-Catalyzed Liberation of Alcohols from Allylcarbonates. *Org. Lett.* **13**, 5544-5547 (2010).

(2) Durandetti, M., Devaud, M. & Perichon, J. Investigation of the reductive coupling of aryl halides and/or ethyl chloroacetate electrocatalyzed by the precursor NiX_2(bpy) with X^- =Cl^-, Br^-or $MeSO_3^-$and bpy =2,2'-dipyridyl. *New J. Chem.* **20**, 659–667 (1996).

(3) Sun, Z., Kumagai, N., Shibasaki, M. Photocatalytic α-Acylation of Ethers. *Org Lett.* **19**, 3727-3730 (2017).

(4) Wu, F., Lu, W., Qian, Q., Ren, Q., Gong, H. Ketone Formation via Mild Nickel-Catalyzed Reductive Coupling of Alkyl Halides with Aryl Acid Chlorides. *Org. Lett.* **14,** 3044-3047 (2012).

(5) Cherney, A. H.; Kadunce, N. T.; Reisman, S. E. Catalytic Asymmetric Reductive Acyl Cross-Coupling: Synthesis of Enantioenriched Acyclic α,α-Disubstituted Ketones. *J. Am. Chem. Soc.* **135**, 7442-7445 (2013).
